# Atmospheric‐pressure scanning microprobe matrix‐assisted laser desorption/ionization mass spectrometry imaging of *Neospora caninum*‐infected cell monolayers

**DOI:** 10.1002/ansa.202200016

**Published:** 2022-08-30

**Authors:** Nils H. Anschütz, Stefanie Gerbig, Alejandra M. Peter Ventura, Liliana M. R. Silva, Camilo Larrazabal, Carlos Hermosilla, Anja Taubert, Bernhard Spengler

**Affiliations:** ^1^ Institute of Inorganic and Analytical Chemistry Justus Liebig University Giessen Giessen Germany; ^2^ Institute of Parasitology Justus Liebig University Giessen Giessen Germany

## Abstract

*Neospora caninum* is an obligate intracellular protozoan parasite of the phylum Alveolata (subphylum Apicomplexa) which has not been studied extensively in a biochemical context. *N. caninum* is a primary cause of reproductive disorders causing mummification and abortion not only in cattle but also in other small ruminant species resulting in a substantial economic impact on the livestock industry. In canids, which are the final hosts of *N. caninum*, clinical disease includes neuromuscular symptoms, ataxia, and ascending paralysis. Fatal outcomes of neosporosis have also been reported depending on the host species, age and immune status, however, its zoonotic potential is still uncertain. Therefore, *N. caninum* should be thoroughly investigated. Matrix‐assisted laser desorption/ionisation (MALDI) mass spectrometry (MS) and MS imaging (MSI) were used, combined with high‐performance liquid chromatography (HPLC) to investigate these intracellular parasites. The aim of this study was to identify molecular biomarkers for *N. caninum* tachyzoite‐infected host cells and to further clarify their functions. By atmospheric‐pressure scanning microprobe MALDI MS(I), sections of *N. caninum*‐infected and non‐infected host cell pellets were examined in order to determine potential markers. In vivo, *N. caninum* infects different types of nucleated cells, such as endothelial cells which represent a highly immunoreactive cell type. Therefore, primary bovine umbilical vein endothelial cells were here used as a suitable infection system. For comparison, the permanent MARC‐145 cell line was used as an additional, simplified in vitro cell culture model. HPLC‐tandem MS (HPLC‐MS/MS) experiments combined with database search were employed for structural verification of markers. The statistically relevant biomarkers found by MS and identified by HPLC‐MS/MS measurements were partly also found in infected monolayers. Marker signals were imaged in cell layers of *N. caninum*‐infected and non‐infected host cells at 5 µm lateral resolution.

**Abbreviations**
AP SMALDI: atmospheric‐pressure scanning microprobe matrix‐assisted laser desorption/ionization
*B. besnoiti*
: *Besnoitia besnoiti*
BUVEC: bovine umbilical vein endothelial cells
DHB: 2,5 dihydroxybenzoic acid
DMEM: Dulbecco's Modified Eagle's Medium
e. g.: *exempli gratia*
EGGM: endothelial cell growth medium
ESI: electrospray ionization
FCS: foetal calf serum
FDR: false‐discovery‐rate
h p. i.: hours post infection
HPLC: high performance liquid chromatography
i. e.: *id est*
IgG: Immunoglobulin G
LC: liquid chromatography
*m/z*
: mass‐to‐charge ratio
MALDI: matrix‐assisted laser desorption/ionisation
MARC‐145: african green monkey kidney epithelial cells
MS: mass spectrometry
MS/MS: tandem mass spectrometry
MSI: MS imaging
MTBE: 2‐methoxy‐2‐methylpropane
*N. caninum*
: *Neospora caninum*
PA: phosphatidic acids
PBS: phosphate‐buffered saline
PC: phosphatidylcholines
PCR: polymerase chain reaction
PE: phosphatidylethanolamine
PI: phosphatidylinositol
ppm: parts per million
PS: phosphatidylserine
rpm: revolutions per minute
RT: room temperature
spp.: *species pluralis*
*T. gondii*
: *Toxoplasma gondii*
TFA: trifluoroacetic acid
TIC: total ion count

## 1 INTRODUCTION

*Neospora caninum* is an obligate intracellular parasite (phylum Alveaolata and subphylum Apicomplexa) involving canids as definitive hosts and a wide range of intermediate hosts including cattle, sheep, goats, wild cervids and new world camelids.^1^ Like the closely related parasite *Toxoplasma gondii*, *N. caninum* is considered as tissue‐cyst forming coccidia.^2^ As such, both parasites show strong similarities in their life cycles, morphology, genome and transcriptome.^3^, ^4^, ^5^, ^6^, ^7^ These common features led to *N. caninum* being misidentified as *T. gondii* before 1984.^8^ Canid species [i.e. dogs (*Canis familiaris*), wolves (*Canis lupus*) and coyotes (*Canis latrans*)] can as well act as intermediate hosts for *N. caninum* infections. In ruminant intermediate host species, neosporosis is considered a major cause of reproductive disorders thereby causing significant economic losses mainly in the cattle, goat, sheep and alpaca/llama industry. Neosporosis is considered a globally spreading disease with a focus on the United States, South America, Central America, Australia and Europe.^9^, ^10^ In most cases, diagnosis of *N. caninum* infection is carried out serologically using blood tests for either parasite‐specific antibodies or antigens.^11^, ^12^, ^13^ Detection can also be performed by immunohistochemistry or by molecular diagnostic tools, such as polymerase chain reaction, both options being more complex and expensive.^14^ As stated above, neosporosis is an infectious disease, especially detrimental for canids and cattle. In canids, *N. caninum* is responsible for multisystemic lesions resulting in severe dermal and neuromuscular symptoms in offspring of infected bitches.^1^ Within the life cycle, three different stages are known: fast replicating tachyzoites, slowly proliferating bradyzoites within tissue cysts and sporozoites present in sporulated oocysts. Only two of these stages ‐ tachyzoites and tissue cysts ‐ occur in intermediate hosts, and they show obligate intracellular development.^9^ In general, transmission can take place in several ways, for example, canids can acquire infection by ingesting infected host tissue containing cysts with thousands of bradyzoites, congenitally by transfer of tachyzoite stages or via ingestion of sporulated oocysts from contaminated environments. Conversely, in cattle transplacental transmission via tachyzoites seems to be the most common route of infection when compared to oocyst ingestion,^15^ thus vertical transmission is the major route involved in the spread of bovine neosporosis.^16^ Initial infection of a cattle herd is driven by purchasing infected cattle or by oocyst‐excreting farm dogs.^17^ The symptoms of this disease strongly depend on the host type. In bovines, *N. caninum* can infect the reproductive system of male and female hosts, which implies a major threat to the cattle industry. In bulls, the sperm concentration, viability and motility are significantly lower if infected with *N. caninum*.^18^ In pregnant cows, neosporosis often leads to foetus mummification and/or abortion, and therefore is considered one of the most common infectious causes of abortion worldwide.^19^, ^20^, ^21^ The spread of bovine neosporosis and its consequences lead to significant economic impact with financial losses in the multi‐billion dollar range.^10^ Vaccines against *N. caninum* are rare and inefficient in preventing abortion in cattle.^22^ Since clinically manifested neosporosis has also been reported in two rhesus monkeys,^23^ concerns are rising that *N. caninum* might eventually become a threat to humans.^24^ Moreover, there are reports on the presence of IgG anti‐*N. caninum* antibodies in pregnant women^25^ and in human immunodeficiency virus carriers.^26^ Therefore, this parasite and the disease should be thoroughly investigated.

A comprehensive investigation of *N. caninum*‐infected host cells in a biochemical context has not yet been carried out. Therefore, no lipidomic or metabolomic data of *N. caninum* at a molecular level is available. Available studies are mostly focusing on proteome‐ or genome‐based comparisons to *T. gondii*.^7^, ^27^ In this context, mass spectrometry (MS) was used in a supportive or independent manner to identify molecules or to validate results obtained with other methods. Since the beginning of the millennium, MS instrumentation has improved considerably, allowing a detailed mass analysis that can be used to calculate elemental formulae of compounds on the basis of highly accurate molecular mass values.^28^, ^29^, ^30^ MS imaging (MSI) provides the visualization of analyte distribution in tissues and cells, and parasites can be visualized by MSI if specific markers are determined. Tachyzoites are approximately 6 × 2 µm in size.^9^ Due to the improvement in MSI instrumentation, lateral resolutions of 1–2 µm became accessible, which implies that assembled or even individual cells can be examined by this method.^31^

In this work, high‐performance liquid chromatography (HPLC) and matrix‐assisted laser desorption/ionisation (MALDI), coupled with MS and MSI^32^ were used to investigate the cellular metabolism of parasites in infected host cells. Model systems for cattle were used with tachyzoites as the corresponding stages. Characteristic m/z signals for the infection of host cells with *N. caninum* were identified using the combination of MSI, HPLC‐MS and statistical data analysis.

## 2 EXPERIMENTAL SECTION

### 2.1 Parasites

*N. caninum* (strain Nc1) tachyzoites were maintained by serial passages *in Mycoplasma* spp.‐free primary bovine umbilical vein endothelial cells (BUVEC) or permanent African green monkey kidney epithelial cells (MARC‐145). Vital *N. caninum* tachyzoites were collected from supernatants of infected host cell monolayers, filtered through 5 µm sterile syringe filters (Sartorius, Goettingen, Germany) to remove cell debris, pelleted (400 × *g*, 12 min), resuspended in cell culture medium, counted (Neubauer haemocyte chamber using the inverted microscope IX81, Olympus, Shinjuku City, Tokyo, Japan) and used for infection of BUVEC. For pure parasite pellets, freshly released tachyzoites were collected from cell culture supernatants, filtered with a 5 µm syringe filter (Sartorius), pelleted (400 × *g*, 12 min), and washed twice with sterile phosphate‐buffered saline (PBS) 1X (Sigma‐Aldrich, Steinheim, Germany). After fixation with 2.5% glutaraldehyde (Merck, Darmstadt, Germany) for 10 min at room temperature (RT), fixed pellets were immediately frozen in liquid nitrogen and stored at −80°C until further use.^33^

### 2.2 Cell culture

MARC‐145 cell layers were maintained in Dulbecco's Modified Eagle Medium (Sigma‐Aldrich) cell culture medium supplemented with 1% penicillin (500 U/ml; Sigma‐Aldrich), streptomycin (500 mg/ml; Sigma‐Aldrich) and 5% foetal calf serum (FCS; Gibco, part of Thermo Fisher Scientific, Dreieich, Germany) and incubated at 37°C and 5% CO_2_ until confluency. Primary BUVEC were maintained in modified ECGM [endothelial cell growth medium (PromoCell, Heidelberg, Germany); 30% (v/v) ECMG and 70% (v/v) M199, supplemented with 1% penicillin and streptomycin and 5% FCS] at 37°C in 5% CO_2_ atmosphere until confluency.^34^

### 2.3 Preparation of cell pellets and cell monolayers

For cell pellet preparation, confluent (*n* = 3) cell layers of BUVEC or MARC cultured in 75 cm^2^ flasks were infected with freshly released *N. caninum* tachyzoites at a multiplicity of infection (MOI) = 5:1. At 24 h post‐infection, infected and non‐infected host cell monolayers were washed with sterile PBS 1X (Sigma‐Aldrich) prior to fixation with 2.5% glutaraldehyde (10 min, RT). Monolayers were detached from flasks using cell scrapers (Greiner Bio‐One, Kremsmünster, Austria) and flasks were washed with sterile PBS 1X (Sigma‐Aldrich) for cell collection. After centrifugation (400 × *g*, 10 min), cell pellets were transferred to microcentrifuge tubes (1.5 ml, Eppendorf, Hamburg, Germany) and washed twice with sterile PBS 1X to remove any traces of fixative (1150 × *g*, 5 min). Fixed pellets were immediately frozen in liquid nitrogen and stored at −80°C until further use.^33^ Also, BUVEC were seeded on glass coverslips (15 mm; Thermo Fisher Scientific) and allowed to grow until confluency. Then, cell layers were infected as described above. Following fixation (2.5% glutaraldehyde; 10 min, RT), cell layers were washed carefully twice with PBS 1X (Sigma‐Aldrich) and were allowed to dry before being stored at −80°C until further use.

### 2.4 Preparation of cryo‐sections

To produce comparable samples for MALDI‐MS, three technical replicates of sections were prepared from each cell pellet of each cell type. In total, three different biological replicates were used for primary bovine endothelial cells. In all cases, sections with a thickness of 30 µm were cut by a microcryotome (Microm HM 525, Microm International GmbH, part of Thermo Fisher Scientific, Walldorf, Germany) at −25°C with a cutting angle of 11° using a Microm Sec35p^®^ blade.

### 2.5 MALDI‐MS sample preparation

Note that, 2,5‐Dihydroxybenzoic acid (DHB, Merck, Darmstadt, Germany) was used as a matrix for MALDI measurements in positive‐ion mode. The matrix solution was prepared by dissolving DHB (30 mg/ml) in 1:1 acetone–water, adding 0.1% of trifluoroacetic acid (Sigma Aldrich). The cell pellet‐derived sections and the sections of the host tissue were covered by spraying 100 µl of the matrix solution at a constant flow rate of 10 µl/min using a dedicated pneumatic sprayer (SMALDIPrep; TransMIT GmbH, Giessen, Germany). For cell layer samples, the volume of the matrix solution was increased by 10 µl to reduce the reflectivity of the sample surface during autofocusing operation.

### 2.6 Metabolite extraction

Metabolite extraction was performed following literature.^35^ The metabolite yield is strongly dependent on the completeness of disruption of assessed cells during extraction. The sample and 25 µl of 0.1% ammonium acetate (Honeywell, Riedel‐de Haen, LC‐MS Chromasolv) were transferred to a potter homogenizer for cell lysis. To this lysate, 100 µl methanol (Sigma Aldrich) and 400 µl 2‐methoxy‐2‐methylpropane (MTBE, Sigma Aldrich) were added. The mixture was incubated at 4°C and 900 rpm for 1 h. Afterwards, 200 µl of ice‐cold MS‐grade water was added in order to initiate phase separation. The sample was centrifuged for 10 min at 1000 g. The upper organic phase was removed, and the lower aqueous phase was reextracted. For this purpose, 200 µl MTBE/methanol/water (4:1.2:1; v/v/v) were added. After incubation at 4°C and 900 rpm for 1 h and centrifugation for 10 min at 1000 g, the organic phase was again removed. The two organic phases were combined and dried under nitrogen gas flow. The sample was resuspended in 500 µl acetonitrile/water (60:40; v/v).

### 2.7 UHPLC‐MS/MS analysis

Liquid chromatographic separation was performed on a Dionex UltiMate 3000 RSLC‐System (Thermo Fisher Scientific), using a 2.6 µm C18 (100 × 2.1 mm) UHPLC column (Kinetex Phenomenex), coupled online to a Q Exactive HF‐X (Thermo Fisher Scientific, Dreieich, Germany) orbital trapping mass spectrometer. The binary gradient was modified from the literature.^36^ The solvent systems used were as follows: solvent A (60:40 acetonitrile: water, 0.1% formic acid, 10 mM ammonium formate [Sigma Aldrich]) and solvent B (90:8:2 isopropanol: acetonitrile: water, 0.1% formic acid, 10 mM ammonium formate [Sigma Aldrich, Germany]). The elution was performed with a gradient over 32 min. Starting condition was 32% of solvent B for 1.5 min. Until 4 min, solvent B was increased to 45%. From 4 to 5 min, solvent B was increased to 52%, from 5 to 8 min to 58%, from 8 to 11 min to 66%, from 11 to 14 min to 70%, from 14 to 18 min to 75% and from 18 to 21 min to 97%. From 21 to 25 min, the gradient was held constant. By the 26th minute, solvent B was reduced to 32% again. Subsequently, the gradient was kept unchanged for 7 min. The flow rate was kept constant at 260 µl/min throughout the whole measurement.

### 2.8 MALDI‐mass spectrometry (I)

MALDI‐MS and MALDI‐MSI experiments were carried out using an AP‐SMALDI5 AF (TransMIT GmbH) imaging system (pixel size: ≥ 5 µm) coupled to a Q Exactive HF (Thermo Fisher Scientific) orbital trapping mass spectrometer (mass resolution R = 240000 @ *m/z* 200). In order to achieve the best possible signal intensities and to ensure optimal comparability of the different measurements, the non‐flat monolayers were measured with the help of the pixelwise autofocus (3D‐surface imaging) system. Due to the small size of the parasites, the highest possible lateral resolution of 5 µm was chosen. In the case of the cell pellet sections, a pattern of 50 × 50 pixels was measured with a lateral resolution (step size) of 10 µm and a slightly defocused laser beam. Due to the larger (defocused) laser spot area at 10 µm, higher signal intensities were obtained. This approach with its constant number of 2500 spectra for each sample led to consistently reliable data. The major advantage here lies in the higher signal intensities due to the larger sampled area compared to the monolayers.

### 2.9 Data processing

To find potential biomarkers within the cell pellets, the software Mirion (TransMIT GmbH) in combination with the Perseus software platform (MPI of Biochemistry, Martinsried, Germany) was used.^37^ The employed procedure was based on published literature.^33^ With the help of Mirion, all MALDI measurements were stitched together. A list of all *m/z* values with an image coverage above or equal to 0.5% was exported. The corresponding deviations (±5 ppm) were calculated, and this new list was applied to all the single cell‐section measurements. The results were then imported into Perseus. With Perseus, the data sets were categorized into infected and non‐infected host cells. In the second and third steps, a normalization was carried out, first by dividing the intensity values by their sum, then by using the Z‐score (median). In the fourth step, multiple‐sample tests were carried out (ANOVA; permutation‐based false‐discovery‐rate, FDR = 0.05, number of randomizations = 250). In the fifth step, the corresponding values from step four were filtered. In the sixth step, post hoc tests were performed with the remaining values (visualized using hierarchical clusters). Afterwards, data were prepared for HPLC‐MS/MS. Lipid Maps was used to generate annotations from the *m/z* values found by Perseus. A mass list was then generated with these exact masses and their deviations (±5 ppm). For identification of the detected molecular markers, the software LipidMatch (SECIM, Gainesville, USA) in collaboration with ProteoWizard (MSConvertGUI) and MZmine were used.^38^, ^39^, ^40^

## 3 RESULTS AND DISCUSSION

### 3.1 Detection of infection markers in cell pellets and subsequent assessment of statistical relevance

**FIGURE 1:**
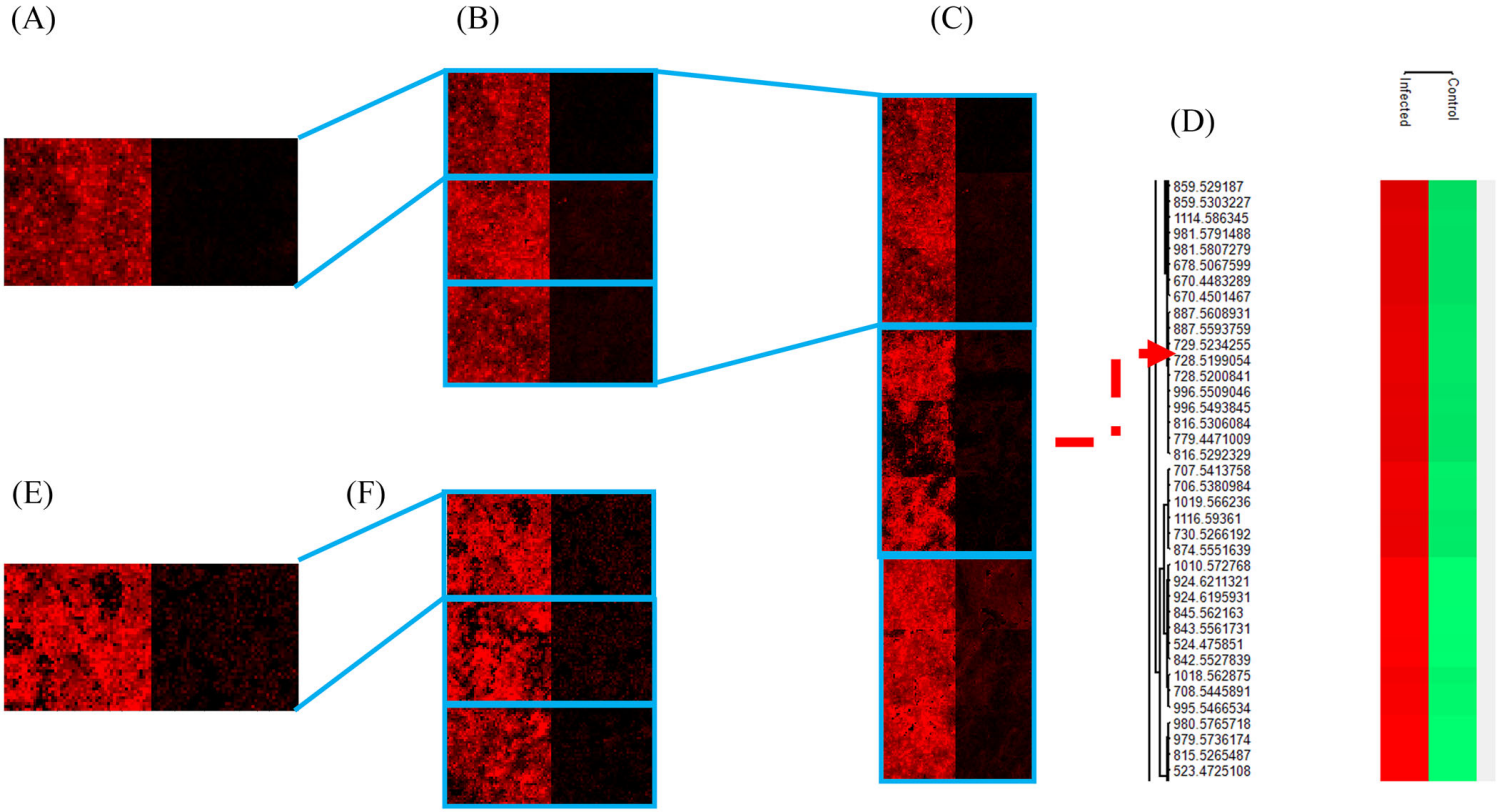
Detection of molecular markers in cell pellet sections of N. caninum‐infected cells. (A–C) bovine umbilical vein endothelial cells (BUVEC): phosphatidylcholines (PC) (14:0_16:0) as [M+Na]^+^, *m/z* 728.5164 ± 5 ppm; (D) Segment of a heat map generated with Perseus; (E,F) MARC: PC (30:0) as [M+H]^+^, *m/z* 678.5034 ± 5 ppm

BUVEC was used as a model system in order to enable the best possible representation of an in vivo scenario on fast replicating tachyzoites in vessel endothelium during the acute phase of bovine neosporosis. For comparative reasons, MARC‐145 cells were also used. This permanent cell line was mainly used to promote massive intracellular *N. caninum* replication, since large numbers of viable tachyzoites were obtained, which were later needed to infect primary BUVEC. Permanent cell lines are easy to handle and offer a pure cell population, thus enabling a consistent sample and reproducible results. Due to their fast and endless proliferation, a practically unlimited supply of material is provided. Nonetheless, since permanent cell lines are either genetically manipulated or of tumorous origin, they may not reflect physiological reactions like in primary cells and therefore may provide deviant results.^41^ Primary, as well as permanent cells, include a cycle of cell division which might affect cellular metabolism in vitro. In this paper, the two different cell lines were compared based on their different cell‐derived reactions after parasitic infection.^42^, ^43^, ^44^, ^45^ However, the focus of this work was on the more realistic primary host cell, namely highly immunoreactive endothelial cells.^34^, ^42^ In order to obtain a homogeneous sample pattern for the MALDI‐MS experiments, cell pellets were cut into consecutive thin sections. This methodology facilitates statistical evaluability. Figure 1 illustrates the chosen experimental approach. Different BUVEC (*n* = 3) cell sections were measured (Figure 1A–C) and the signal at *m*/*z* 728.5164 as a statistically relevant marker for the infection was visualized using the red colour channel. Figure 1A shows two measurements with 50 × 50 pixels. All measurements of the cell pellets were conducted with a step size of 10 µm. The larger step size, combined with a corresponding defocusing of the laser beam, led to larger spot size, resulting in more material being removed and ionized by the laser beam.

The left part of Figure 1A illustrates an infected section, and the right part the corresponding control sample. The two samples were placed next to each other on one sample holder, sprayed with matrix and measured subsequently thereby applying identical experimental conditions. After that, another two infected and control pairs of the same biological sample were measured in order to obtain triplicate measurements (Figure 1B). Technical replicas were measured to exclude potential errors in sample preparation (e.g. sectioning) or possible heterogeneities within the cell pellets. This procedure was repeated for two other biological replicates. Figure 1C shows the resulting images of the three biological replicates with three technical replicates. Biological replicates are needed to minimize variations of results due to varying individual metabolic responses of animals to infection. Preparation of samples and their previous storage were all carried out under the same conditions. Figure 1E and F show measurements of MARC‐145 cell pellet sections. Figure 1E is the equivalent to Figure 1A in this context. The signal *m*/*z* 678.5034, visualized in the red colour channel, is a statistically relevant marker for infection. Figure 1F shows the corresponding triplicates. Since MARC‐145 is a permanent cell line, biological replicates cannot be measured. To find potential biomarkers within those cell pellet sections, the software Mirion in combination with the Perseus software platform was used.^37^ The biomarkers found in these experiments were set as a basis for all subsequent experiments. With Perseus, the data sets were categorized into *N. caninum*‐infected and non‐infected ones. In the next step, two standardizations were carried out, followed by multiple‐sample tests (ANOVA; permutation‐based false‐discovery‐rate, FDR = 0.05, number of randomizations = 250). The corresponding values from the preceding step were filtered and non‐fitting values were rejected. In the last step, post hoc tests were performed with the remaining values (visualized using hierarchical clustering, see Figure 1D). For the BUVEC model system, 582 marker signals were found for infection in positive‐ion mode (Table S1) and 659 marker signals for infection in negative‐ion mode (Table S2). In the control samples, 48 (Table S3) and 411 marker signals (Table S4) were detected, respectively.

### 3.2 Annotation of markers

**FIGURE 2:**
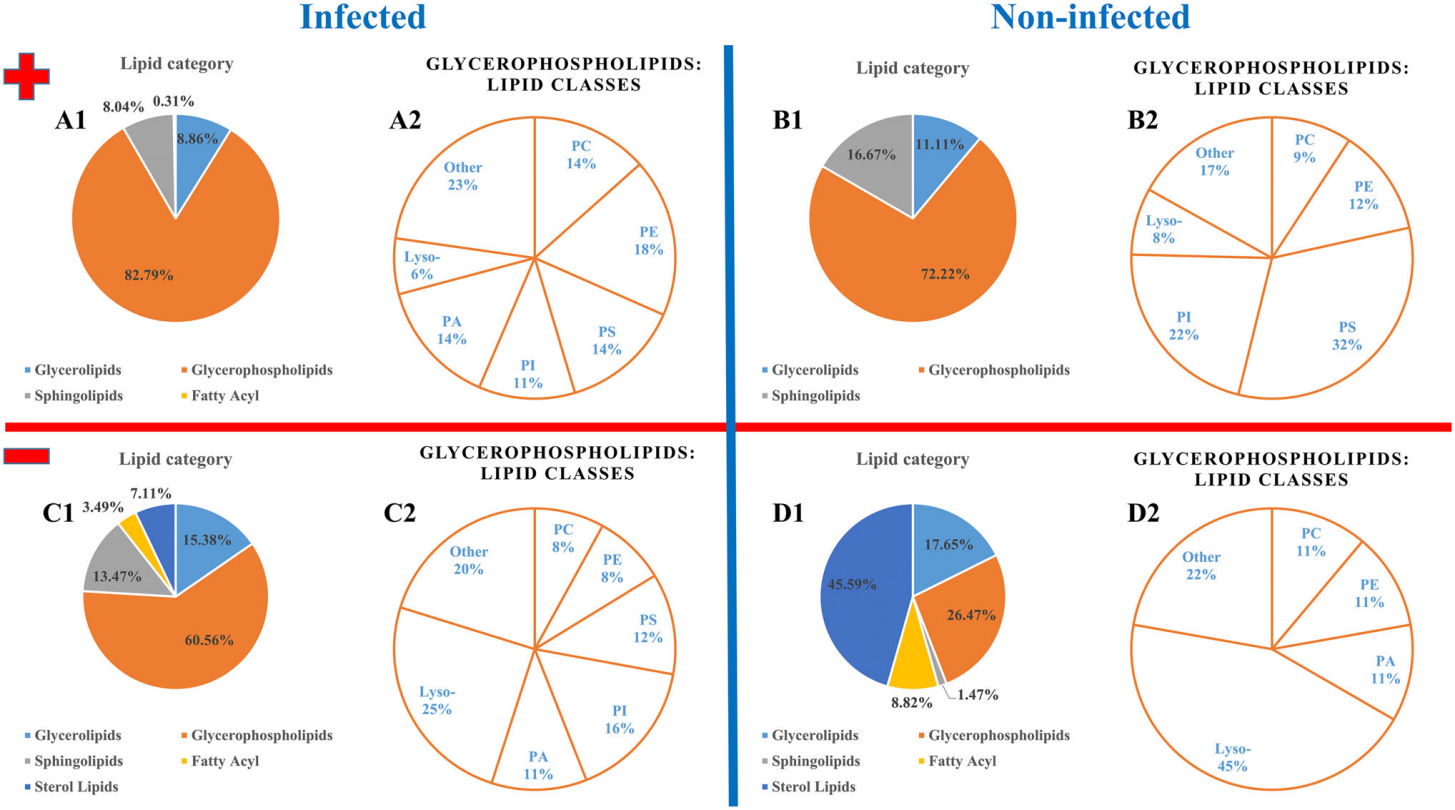
Abundances of categories of detected bovine umbilical vein endothelial cells (BUVEC) markers (fractions of signal numbers in [%]). (A,B) positive‐ion mode; (C,D) negative‐ion mode; (A,C) infected samples; (B,D) control samples; 1: lipid categories, 2: Detected lipid classes in the glycerophospholipids category

The LIPID MAPS database of computationally‐generated “bulk” lipid species, a virtual database composed of major classes of lipid species, was used to annotate previously determined markers.^46^ Lipids are basic components for structural and functional categories of cells. In cell membranes, lipids divide the functional areas and are involved in accomplishing various aspects of signal transmission. In the case of MALDI measurements of cell pellets and the following statistical evaluation, all *m/z* signals were taken into account. Subsequently, the focus was exclusively on lipids. Due to the restriction to lipids (database and subsequent extraction for HPLC‐MS/MS), not every signal recognized as a marker was annotated. In order to obtain reasonable annotations, expedient ion adducts with the respective polarity were selected as LIPID MAPS search criteria. Only single charged species were selected. In positive‐ion mode, ions of types [M+H]^+^, [M+H‐H_2_O]^+^, [M+Na]^+^, [M+NH_4_]^+^ and [M+K]^+^ and in negative‐ion mode [M − H]^–^, [M+Cl]^–^, [M+HCOO]^–^, [M+Oac]^–^ and [M‐CH_3_]^–^ were taken into account. In LIPID MAPS, a mass tolerance of *m*/*z* ± 0.05 was chosen and afterwards, values with a calculated deviation of more than 5 ppm between obtained and measured values were discarded. If several different annotations for the same *m*/*z* value were found, annotations which deviated by more than 1 ppm from the annotation with the smallest deviation were discarded. The corresponding results are illustrated in Figure 2. The number of annotations is higher than the number of *m*/*z* values since more than just one annotation can meet the criteria described above. In the BUVEC model system, 982 annotations were determined for the positive‐ (for 116 different *m*/*z* values, Table S5) and 1654 annotations for the negative‐ion mode (for 357 different *m*/*z* values, Table S6). For control samples, 90 annotations (39 different *m*/*z* values, Table S8) were determined for the positive‐ and 68(49 different *m*/*z* values) annotations for the negative‐ion mode (Table S9). Figure 2 shows the fractions of signal numbers (in [%]) of all annotated lipid categories (A1, B1, C1 and D1) as well as the different lipid classes of the particularly prominent lipid category of glycerophospholipids (A2, B2, C2 and D2). While A and B show the results for the positive‐ion mode, C and D represent the negative‐ion mode. In positive‐ion mode, the *N. caninum*‐infected samples had a low percentage in the number of phosphatidylserines (PS) and phosphatidylinositols (PI) compared to the control samples. The proportion of phosphatidylethanolamine and especially phosphatidic acids (PA) was found to be increased in infected samples. Sterols were annotated as marker molecules in negative‐ but not in positive‐ion mode. Due to the lack of acidic and basic groups, sterols are difficult to ionize especially in a positive‐ion mode without derivatization.^47^, ^48^, ^49^, ^50^ In positive‐ion mode, [M+H−H_2_O]^+^ is the most abundant ion, in the negative‐ion mode, it is the deprotonated species [M − H]^–^. Their percentage was particularly high in the control samples in negative‐ion mode. The absolute number of sterol signals in *N. caninum*‐infected samples was still more than three times as high (104 vs. 31). It is well‐known that sterol levels are increased in host cells infected with other apicomplexan parasite species.^51^ In the negative‐ion mode, the proportion of lyso‐lipids in infected samples was almost halved, with a high PI proportion. However, one has to take into account that the presented annotations are solely based on accurate mass at this level and that there are often several annotations for a single *m/z* value. For the MALDI MS data, direct identification via on‐tissue MS/MS was not possible due to low signal intensities.

**FIGURE 3:**
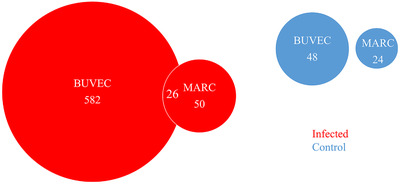
Venn diagram of markers found in the positive‐ion mode for Neospora caninum‐infected and non‐infected bovine umbilical vein endothelial cells (BUVEC) and MARC cells. For infected cells, 582 markers were found in BUVEC and 50 markers in MARC‐125. 26 of these markers (52% of the MARC‐125 markers) were found in both cell models. For the control samples, only 48 (BUVEC) and 24 (MARC) markers were found, with no overlap

In Figure 3, the BUVEC‐ and the MARC‐145‐based models are compared in positive‐ion mode. As expected, the two models led to highly varying results.^41^ The primary bovine host endothelial cells, that is, BUVEC, revealed significantly more markers, thus underlining the in vivo replication site of *N. caninum* tachyzoites and highlighting the importance to indeed analyse primary cell models to be close to the in vivo situation. Thus, in total 582 markers were found for *N. caninum‐*infected BUVEC and only 50 for infected MARC‐145. 26 of these markers were found in both cell line systems, suggesting that these markers originated from the parasites. Fewer markers were found in each of the control samples (BUVEC: 48; MARC‐145: 24), and there was no overlap between the markers of the two model systems here used.

### 3.3 LC‐MS/MS‐based identification of annotated markers

**FIGURE 4:**
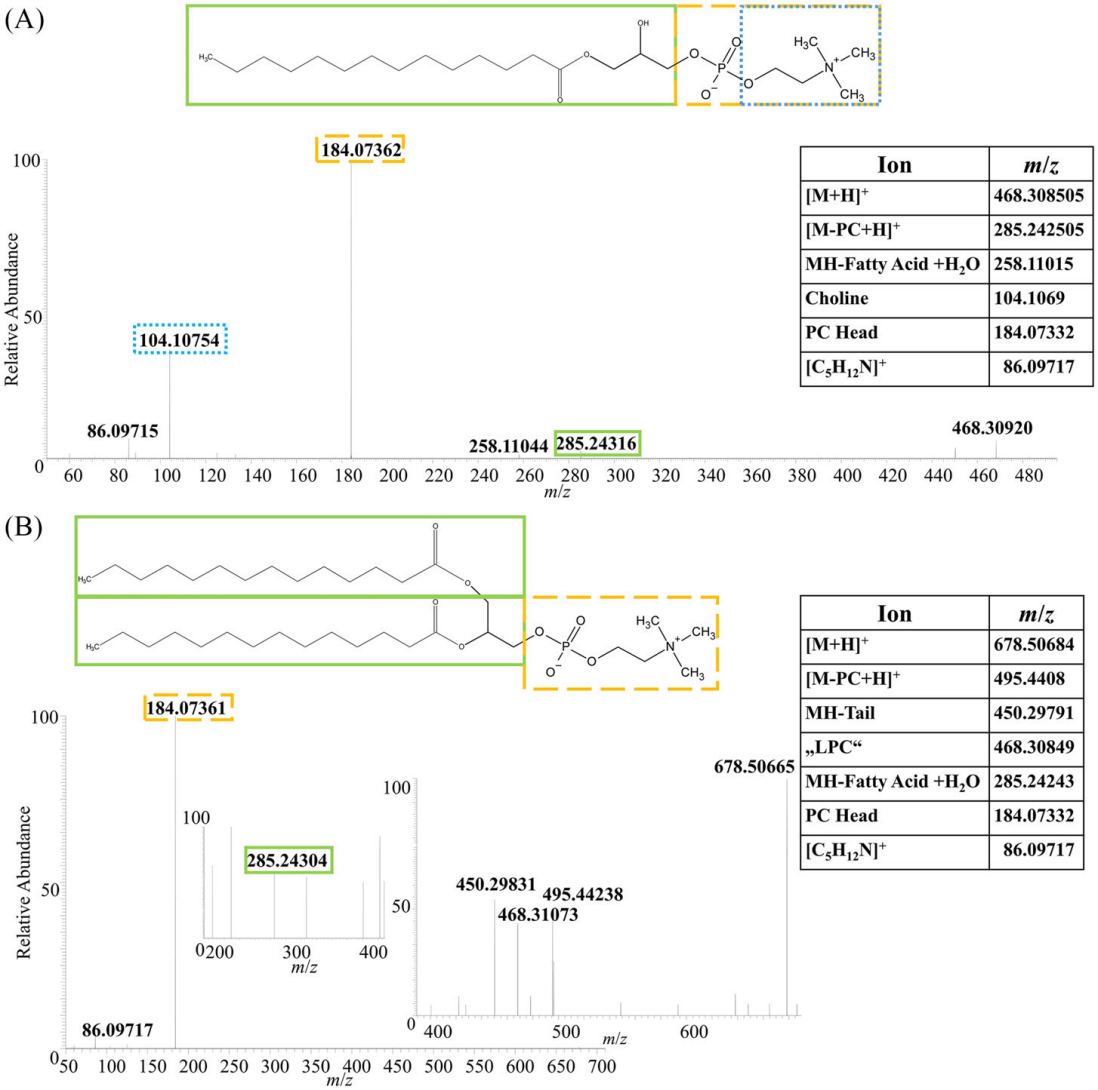
Liquid chromatography‐electrospray ionization tandem mass spectrometry (LC‐ESI‐MS/MS) measurements of two selected infection markers. The resulting fragment ion signals were identified by comparison to a database. (A) (LPC 14:0) as [M+H]^+^; (B) (PC 14:0_14:0) as [M+H]^+^

The annotations obtained from LIPID MAPS were used to create inclusion lists for HPLC‐MS/MS measurements. High‐resolution full MS and MS/MS spectra were recorded. For identification of the detected molecular markers, the software LipidMatch (SECIM, Gainesville, USA) in combination with ProteoWizard (MSConvertGUI) and Mzmine were used.^38^, ^39^, ^40^ The settings made in LipidMatch can be found in Table S7, and two examples are shown in Figure 4.

In total, 28 annotations were confirmed by MS/MS experiments (Table S10) in negative‐ion mode. Phosphatidylinositols, as components of cellular membranes, were the most abundant class of lipids. They play a critical role in membrane dynamics, trafficking, and cellular signalling, which agrees well with our hypothesis that the markers are a cell response to infection.^52^ In positive‐ion mode, 12 annotations were confirmed by MS/MS analysis (Table S11). The phosphatidylcholines (PC) lipid class was highly abundant, which has already been observed for infections with other Apicomplexa.^53^ For corresponding control samples, three annotations were confirmed by MS/MS analysis (Table S12). Remarkably, almost all identified markers, regardless of the ion mode, contained a short‐chain fatty acid. In the literature, this finding is also described to be typical for the zoonotic *T. gondii*.^53^

### 3.4 MSI of cell layers at a high lateral resolution

**FIGURE 5:**
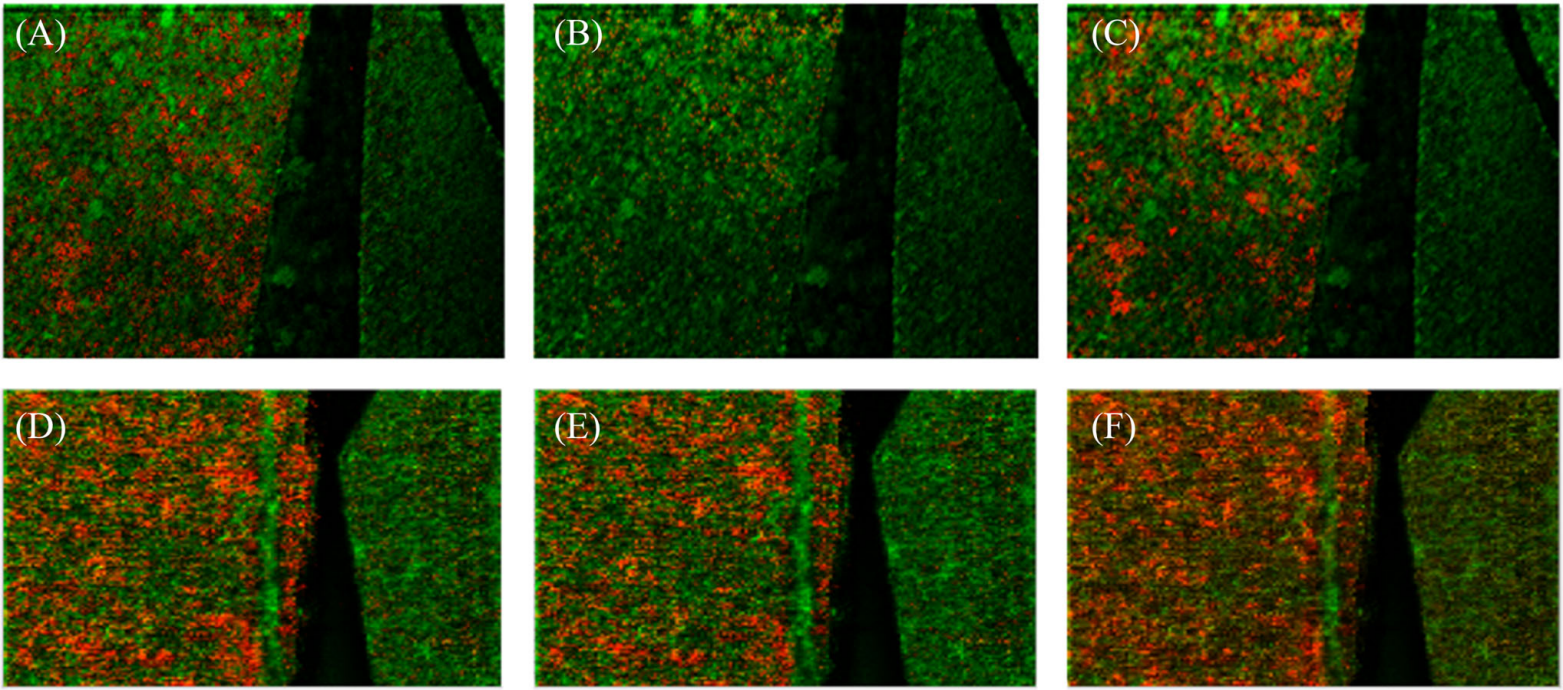
Matrix‐assisted laser desorption/ionisation mass spectrometry imaging (MALDI MSI) measurements of bovine umbilical vein endothelial cells (BUVEC) cell monolayers, infected with N. caninum and measured with 5 µm laser focus diameter and step size. The green channel represents the total ion count (TIC). The red channel shows the distribution of various infection markers (±5 ppm mass tolerance) identified earlier by liquid chromatography‐tandem mass spectrometry (LC‐MS/MS) measurements of cell pellets. Each frame A to F contains an infected monolayer on the left and a control monolayer on the right, separated by a blank area. (A–C) Measurements in negative‐ion mode: (A) infection marker signal at *m/z* 645.4501 (red), identified as both, PA (14:0_18:1) as [M − H]^–^ and PA (16:0_16:1) as [M − H]^–^, (B) infection marker signal at m/z 781.4873 (red), identified as PI (14:0_16:0) as [M − H]^–^, (C) infection marker signal at m/z 836.5447, identified as PS(18:0_22:5) as [M − H]^–^; (D–F) Measurements in positive‐ion mode: (D) infection marker signal at *m/z* 728.5201, identified as phosphatidylcholines (PC) (14:0_16:0) as [M + Na]^+^, E: infection marker signal at *m/z* 704.5225, identified as both, PC (14:0_16:1) as [M + H]^+^and PC (12:0_18:1) as [M + H]^+^, F: infection marker signal at *m/z* 678.5068, identified as both, PC (14:0_14:0) as [M + H]^+^ and PC (12:0_16:0) as [M + H]^+^

In total, 40 biomarkers characteristic for *N. caninum* infection and three biomarkers for control samples were identified in BUVEC layers (Table S10–S12). Visualization of the small tachyzoites (<10 µm)^8^ within cells, requires a lateral resolution of at least 5 µm for MSI of monolayers. In each panel of Figure 5, *N. caninum*‐infected BUVEC layers are illustrated on the left side, and control samples on the right side. The green channel represents the total ion count (TIC). The red channel shows the distribution of different, LC‐MS/MS measurements with a mass tolerance of ±5 ppm identified infection markers. As expected, these signals were found exclusively or significantly more pronounced in *N. caninum*‐infected cell layers (left side of each panel). Figure 5A–C shows the measurement in negative‐ion mode. In Figure 5A, the distribution of an infection‐specific signal at *m*/*z* 645.4501 is shown in red, assigned to PA (32:1). Since PA are the precursors for the biosynthesis of many other lipids, an increase in this species can be interpreted as the response of host cells to the tachyzoite replication. Figure 5B shows the distribution of the signal *m*/*z* 781.4873, which was previously assigned to PI (30:0) as [M − H]^–^ by LC‐MS/MS of the cell pellets. As mentioned above, PI plays a critical role in membrane dynamics, trafficking, and cellular signalling, therefore an increase in the abundance of this species would correspond well to an infection‐driven reaction.^52^ Figure 5C shows in red the distribution of the signal at *m*/*z* 836.5447, which was previously assigned to PS (18:0–22:5) as [M − H]^–^. PC are a major component of biological membranes. In line, PS are also well‐known components of cell membranes and play a major role in programmed cell apoptosis,^54^ which suggests that *N. caninum* intracellular replication might have led to cell death. Figure 5D–F shows MS images of monolayers in positive‐ion mode. While in Figure 5D the distribution of an infection‐specific signal at *m*/*z* 728.5201, assigned to PC (30:0) as [M + Na]^+^, is shown, Figure 5E displays the distribution of the signal *m*/*z* 704.5225, which was previously assigned to PC (30:1) as [M + H]^+^. Figure 5F shows the distribution of the signal *m*/*z* 678.5068, which was previously assigned to PC (28:0) as [M + H]^+^.

**FIGURE 6:**
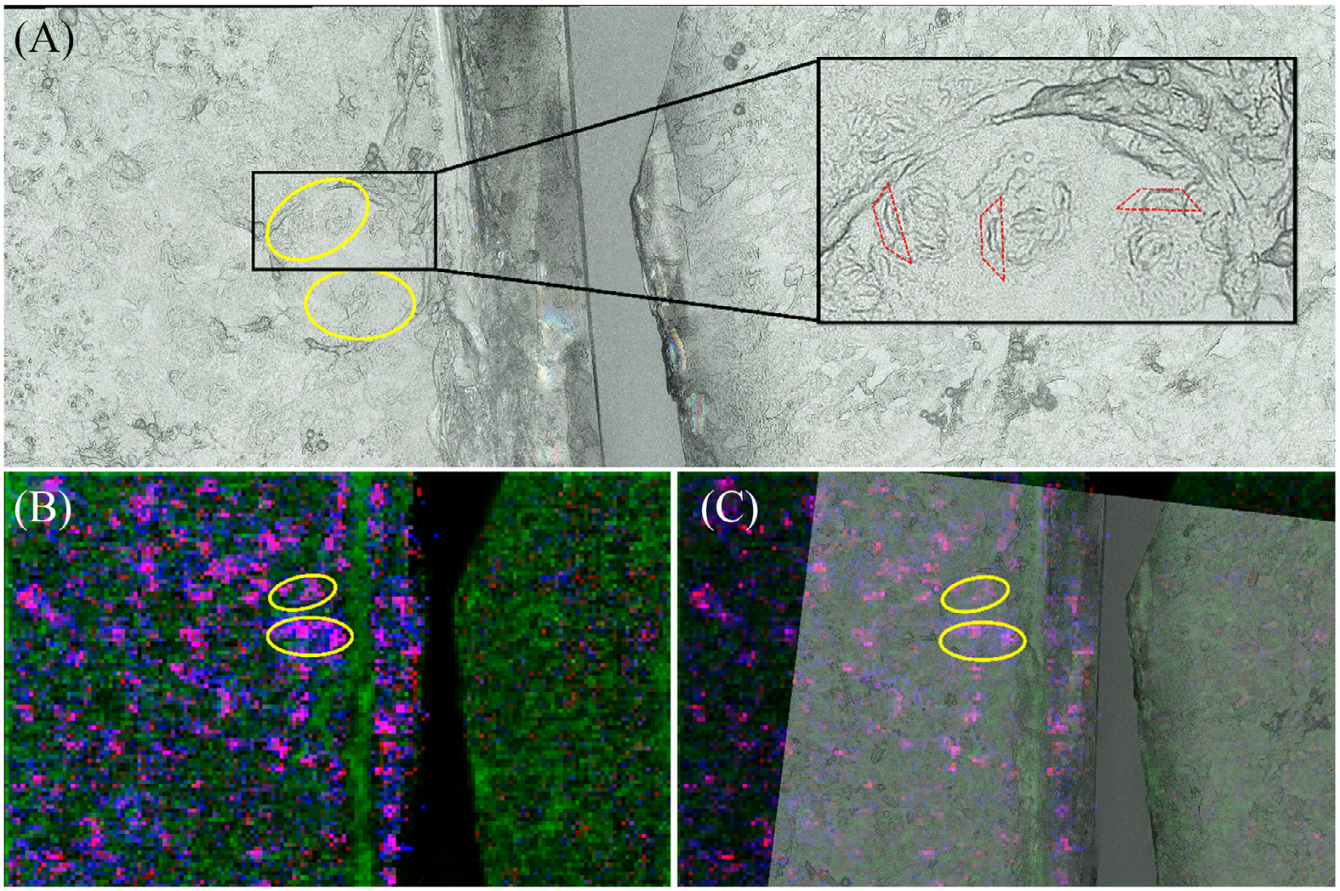
Matrix‐assisted laser desorption/ionisation mass spectrometry imaging (MALDI MSI) measurements of a bovine umbilical vein endothelial cells (BUVEC) layer, infected with N. caninum and measured with 5 µm laser focus diameter and step size. On each panel, the infected monolayer is on the left and a control monolayer is on the right, separated by a blank area. (A) Microscopic image of the two monolayers. Intracellular tachyzoites can be recognized as banana‐shaped structures (outlined in red) exclusively in the infected monolayer, as part of a rosette‐like meront. Two particularly noticeable areas with N. caninum meronts are circled in yellow. (B) MS‐Image of the monolayers in positive‐ion mode. The green channel represents the total ion count (TIC). The red and blue channels show the distribution of two identified infection markers (±5 ppm mass tolerance). The two markers overlap with pink pixels. The red channel represents an infection marker at *m/z* 728.5201, identified as phosphatidylcholines (PC) (29:0) as [M+H]^+^ and the blue channel represents an infection marker at *m/z* 704.5225, identified as PC (30:1) as [M+H]^+^. (C) Overlay of panels A and B. Infection marker signals overlap well with the banana‐shaped (tachyzoites) and rosette‐like (meronts) structures in the yellow circles.

Next, we examined correlations of MS images with structures observed in highly resolved optical microscopic images (Figure 6). In line with previous measurements, an *N. caninum‐*infected monolayer was placed on the left, the control sample on the right of the sample holder to ensure identical experimental conditions. Intracellular tachyzoites of *N. caninum* can be recognized in infected cell layers as typical ‘banana’‐shaped structures in the microscopic image (Figure 6A, encircled in yellow). Figure 6B shows the corresponding MS image of the cell layer in positive‐ion mode (green channel: TIC, red and blue channel: two identified infection markers; ±5 ppm mass tolerance). The red channel represents an infection marker at *m*/*z* 692.5225, identified as PC (29:0) as [M+H]^+^, the blue channel represents an infection marker at *m*/*z* 704.5225, identified as PC (30:1) as [M+H]^+^. The two markers widely overlap, resulting in pink pixels. Both ion signals were also found in controls, but with a much lower signal intensity compared to *N. caninum‐*infected cell layers.

## 4 Comparison of data with other apicomplexan infections on MS/MS level

The identified markers found for *N. caninum* were compared to markers of *Toxoplasma gondii* and *Besnoitia besnoiti*, as previously published.^33^ Ten of identified 40 markers for an *N. caninum* infection were also identified as markers for *T. gondii* and *B. besnoiti* infections of endothelial host cells. A comparison is shown in Table S13. About 80% of these commonly identified markers were PI. As already stated, these molecules play critical roles in membrane dynamics, trafficking and cellular signalling, supporting the hypothesis that these markers are a common response of infected host cells to apicomplexan infections.^52^

## 5 CONCLUSION

To the best of our knowledge, this is the first study examining *N. caninum*‐infected host cells at the molecular level. AP‐SMALDI MS and MSI were used to find lipid biomarkers for infection with the obligate intracellular protozoan parasite *N. caninum*, which belongs to the phylum Apicomplexa, by comparing technical and biological triplicates of infected and non‐infected sections of cell pellet samples. In this context, permanent (MARC‐145) and primary (BUVEC) cell lines were also compared, clearly showing the superiority of the primary cell system. The measurements were carried out in positive‐ and negative‐ion modes and evaluated with statistical software. With this detailed method, 1241 significant markers for infection were found. MS/MS experiments combined with database search were successful in structural verification of 40 markers, while the others could not be unambiguously identified. The obtained data were compared with already published data in the neglected field of apicomplexan parasites (*T. gondii* and *B. besnoiti*). Ten of the 40 identified markers for *N. caninum* infection were also identified as markers for respective infections with *T. gondii* or *B. besnoiti*. Here, 80% of the matching markers were PI. They are assumed to be part of a response of infected host cells to apicomplexan invasion and further replication. For MSI experiments, cell layers were analysed and previously found markers were imaged. This allowed us to display and compare marker compounds in parasite‐infected single host cells and non‐infected controls at 5 µm lateral resolution. The presented work will be the basis for future detailed investigations on fast replicating *N. caninum* tachyzoites and their impact on host cell lipid metabolism. Overall, current data on lipid biomarkers of single *N. caninum*‐infected host endothelial cells unveil lipid sources as essential components for fast replicating tachyzoites. These novel lipid data might help to identify alternative metabolic pathways for novel drug targets not only against *N. caninum* but also against other apicomplexan parasites of veterinary and public health importance.

## CONFLICT OF INTEREST

Bernhard Spengler is a consultant and Nils H. Anschütz is a part‐time employee of TransMIT GmbH, Giessen, Germany. The other authors declare no conflict of interest.

## Supporting information



Supporting Information

## Data Availability

The data that supports the findings of this study are available in the supplementary material of this article.
