# Supplementary material for: Atmospheric‐pressure scanning microprobe matrix‐assisted laser desorption/ionization mass spectrometry imaging of Neospora caninum‐infected cell monolayers
Source: Anal Sci Adv. 2022 Aug 30;3(7-8):244–54. doi: 10.1002/ansa.202200016 (PMC10989629; doi:10.1002/ansa.202200016)
Supplement: Supplementary file 1 — Supporting Information [file ANSA-3-244-s001.pdf]

AP-SMALDI MS imaging of *Neospora caninum*-infected cell monolayers

Nils H. Anschütz[1], Stefanie Gerbig[1], Alejandra M. Peter Ventura[1], Liliana M. R. Silva[2], Camilo Larrazabal[2], Carlos Hermosilla[2], Anja Taubert[2], Bernhard Spengler[1,*]

[1]: Institute of Inorganic and Analytical Chemistry, Justus Liebig University Giessen, Giessen, Germany

[2]: Institute of Parasitology, Biomedical Research Center Seltersberg, Justus Liebig University Giessen, Giessen, Germany

*: Corresponding author

SUPPLEMENTARY INFORMATION

Supplementary Table 1: Posthoc test BUVEC for infected cells in positive-ion mode 1

Supplementary Table 2: Posthoc test BUVEC for infected cells in negative-ion mode........................ 17

Supplementary Table 3: Posthoc test BUVEC for control cells in positive-ion mode 34

Supplementary Table 4: Posthoc test BUVEC for control cells in negative-ion mode 36

Supplementary Table 5: Annotations for infection of BUVEC in positive-ion mode.......................... 47

Supplementary Table 6: Annotations for infection of BUVEC in negative-ion mode 73

Supplementary Table 7: LipidMatch settings 114

Supplementary Table 8: Annotations for control samples of BUVEC in positive-ion mode............. 114

Supplementary Table 9: Annotations for control samples of BUVEC in negative-ion mode 117

Supplementary Table 10: Identified markers for infection in negative-ion mode.............................. 119

Supplementary Table 11: Identified markers for infection in positive-ion mode............................... 120

Supplementary Table 12: Identified markers for control cells in positive-ion mode 121

Supplementary Table 13: Comparison of identified infection markers with literature[30]..................... 121

Supplementary Table 1: Posthoc test BUVEC for infected cells in positive-ion mode

Control	Infected	C: ANOVA Significant	N: -Log ANOVA p value	N: ANOVA q-value	MALDI m/z
**-3.85547**	**3.85547**	+	**3.85547**	**0.00515481**	**313.273839**
**-3.56653**	**3.56653**	+	**3.56653**	**0.00836237**	**314.277114**
**-2.83409**	**2.83409**	+	**2.83409**	**0.0243151**	**339.289487**
**-3.58164**	**3.58164**	+	**3.58164**	**0.0081958**	**340.292846**
**-2.66392**	**2.66392**	+	**2.66392**	**0.031012**	**366.33664**
**-2.78204**	**2.78204**	+	**2.78204**	**0.0262698**	**421.258147**
**-2.30961**	**2.30961**	+	**2.30961**	**0.0485307**	**431.216853**
**-2.49744**	**2.49744**	+	**2.49744**	**0.0382391**	**445.271471**

-3.88451	3.88451	+	3.88451	0.00497854	449.289693
-3.88679	3.88679	+	3.88679	0.00502165	449.290187
-4.84584	4.84584	+	4.84584	0.00103497	450.297803
-3.81771	3.81771	+	3.81772	0.00556735	451.301383
-2.97208	2.97208	+	2.97208	0.0205171	468.308654
-2.48239	2.48239	+	2.48239	0.0387023	469.311985
-3.66539	3.66539	+	3.66539	0.00698529	472.27945
-3.67058	3.67058	+	3.67058	0.00698155	472.280667
-2.60353	2.60353	+	2.60353	0.0337691	475.305567
-2.94067	2.94067	+	2.94067	0.0210423	476.308885
-3.16349	3.16349	+	3.16349	0.0165889	490.289605
-3.16341	3.16341	+	3.16341	0.0165429	490.290552
-2.54634	2.54634	+	2.54634	0.0357832	491.293873
-6.37493	6.37493	+	6.37493	0	495.44103
-5.91509	5.91509	+	5.91509	0	521.456874
-3.88775	3.88775	+	3.88775	0.00504348	522.319336
-8.07849	8.07849	+	8.07839	0	523.472511
-7.61194	7.61194	+	7.61186	0	524.475851
-6.63792	6.63792	+	6.63792	0	525.479078
-2.58491	2.58491	+	2.58491	0.0343385	527.274888
-2.94606	2.94606	+	2.94606	0.0209763	536.335103
-2.30325	2.30325	+	2.30325	0.0487524	539.274914
-3.39247	3.39247	+	3.39247	0.0114444	544.301398
-4.44047	4.44047	+	4.44047	0.00211236	549.488192
-4.88573	4.88573	+	4.88573	0.000942857	550.491572
-3.24842	3.24842	+	3.24842	0.014659	551.49497
-3.89881	3.89881	+	3.89881	0.00500441	558.315579
-2.77243	2.77243	+	2.77243	0.0266525	563.463791
-2.47345	2.47345	+	2.47345	0.0392256	567.304487
-2.30114	2.30114	+	2.30114	0.0486976	576.364739
-2.48417	2.48417	+	2.48417	0.0386977	576.474089
-2.58118	2.58118	+	2.58118	0.0344952	577.252577
-4.08699	4.08699	+	4.08699	0.00359809	579.437376
-2.61794	2.61794	+	2.61794	0.032949	579.524433
-2.95489	2.95489	+	2.95489	0.0207656	579.533395
-2.80726	2.80726	+	2.80726	0.0252129	580.536693
-2.42384	2.42384	+	2.42384	0.0422867	581.31945
-2.40738	2.40738	+	2.40738	0.0429622	581.320679

-3.47945	3.47945	+	3.47945	0.00997351	589.479005
-3.4795	3.4795	+	3.4795	0.0100066	589.480203
-2.87265	2.87265	+	2.87265	0.0230472	589.55414
-2.5266	2.5266	+	2.5266	0.0365725	590.482824
-5.07064	5.07064	+	5.07064	0.0006875	591.494623
-4.5023	4.5023	+	4.5023	0.0019422	592.497915
-2.68201	2.68201	+	2.68201	0.0303659	595.335674
-2.29034	2.29034	+	2.29034	0.0494563	598.346677
-2.53604	2.53604	+	2.53604	0.0363037	599.349143
-2.55134	2.55134	+	2.55134	0.0357815	599.350957
-3.40557	3.40557	+	3.40557	0.0111824	603.533435
-2.7126	2.7126	+	2.7126	0.0286584	604.323525
-2.38898	2.38898	+	2.38898	0.0439524	604.396163
-3.69265	3.69265	+	3.69265	0.00682264	604.536773
-3.49954	3.49954	+	3.49954	0.00944	605.452989
-4.41235	4.41235	+	4.41235	0.00223204	605.539589
-2.82149	2.82149	+	2.82149	0.0248279	605.54849
-2.82134	2.82134	+	2.82134	0.0247739	605.550224
-3.67771	3.67771	+	3.67771	0.00698507	606.552486
-5.11697	5.11697	+	5.11697	0.000605042	607.555753
-3.311	3.311	+	3.311	0.012944	611.367642
-2.38828	2.38828	+	2.38828	0.0439321	612.555433
-2.55118	2.55118	+	2.55118	0.0357218	614.344508
-5.81084	5.81084	+	5.81084	0	617.510934
-4.628	4.628	+	4.628	0.00139759	618.514352
-3.68512	3.68512	+	3.68512	0.00690637	619.517798
-4.82879	4.82879	+	4.82879	0.00111111	619.526453
-4.11823	4.11823	+	4.11823	0.00345098	620.529505
-4.12014	4.12014	+	4.12014	0.00346535	620.53043
-2.42189	2.42189	+	2.42189	0.0423874	626.305941
-3.3446	3.3446	+	3.3446	0.0121802	631.565341
-3.42913	3.42913	+	3.42913	0.0108553	632.568698
-3.34901	3.34901	+	3.34901	0.0121813	633.484752
-3.44653	3.44653	+	3.44653	0.0106405	633.581111
-2.96802	2.96802	+	2.96802	0.0205243	634.487609
-2.34178	2.34178	+	2.34178	0.0470249	635.498876
-2.3455	2.3455	+	2.3455	0.0468952	635.500496
-5.11212	5.11212	+	5.11212	0.000595041	641.414522

-2.81612	2.81612	+	2.81612	0.0248985	643.41338
-6.16615	6.16615	+	6.16615	0	643.429237
-6.17922	6.17922	+	6.17922	0	643.430585
-3.3633	3.3633	+	3.3633	0.0117697	643.526697
-2.36325	2.36325	+	2.36325	0.045541	644.529561
-2.363	2.363	+	2.363	0.0454983	644.530472
-3.77595	3.77595	+	3.77595	0.00602429	645.54233
-2.8446	2.8446	+	2.8446	0.0241242	646.545748
-2.76262	2.76262	+	2.76262	0.0270678	648.459125
-2.7574	2.7574	+	2.7574	0.0272474	648.46045
-2.66851	2.66851	+	2.66851	0.0308491	653.549418
-2.66851	2.66851	+	2.66851	0.0309113	653.550355
-2.59524	2.59524	+	2.59524	0.034191	654.409583
-2.59524	2.59524	+	2.59524	0.0342578	654.4104
-2.96714	2.96714	+	2.96714	0.0205036	654.55291
-2.4697	2.4697	+	2.4697	0.0394752	655.357474
-2.67297	2.67297	+	2.67297	0.0307475	655.413203
-2.98046	2.98046	+	2.98046	0.0206667	658.334597
-3.95618	3.95618	+	3.95618	0.0046758	659.499553
-3.95613	3.95613	+	3.95613	0.00467273	659.50069
-2.36779	2.36779	+	2.36779	0.0452282	660.503968
-2.39004	2.39004	+	2.39004	0.0439591	661.516051
-2.91243	2.91243	+	2.91243	0.0218249	662.474823
-3.34787	3.34787	+	3.34787	0.0121687	663.39788
-2.94396	2.94396	+	2.94396	0.0209906	664.491104
-4.24384	4.24384	+	4.24384	0.00264615	665.412534
-3.29726	3.29726	+	3.29726	0.0134311	666.415759
-3.85794	3.85794	+	3.85794	0.00514286	667.429658
-3.85794	3.85794	+	3.85794	0.00516456	667.430562
-3.76604	3.76604	+	3.76604	0.00603984	668.433495
-2.29148	2.29148	+	2.29148	0.0494204	669.42833
-4.79554	4.79554	+	4.79554	0.00118121	669.437152
-6.70367	6.70367	+	6.70366	0	669.445844
-6.77477	6.77477	+	6.77476	0	670.448329
-6.77477	6.77477	+	6.77476	0	670.450147
-3.7715	3.7715	+	3.7715	0.0060241	671.444346
-5.60799	5.60799	+	5.60799	8.42E-05	671.452394
-2.31725	2.31725	+	2.31725	0.0483987	671.557998

-4.14905	4.14905	+	4.14905	0.00319598	672.456713
-3.20153	3.20153	+	3.20153	0.0155944	676.491132
-6.75982	6.75982	+	6.75982	0	678.50676
-5.98737	5.98737	+	5.98737	0	679.509768
-5.98737	5.98737	+	5.98737	0	679.510287
-3.70551	3.70551	+	3.70551	0.00674809	680.513348
-2.95842	2.95842	+	2.95842	0.0207386	685.419661
-2.95858	2.95858	+	2.95858	0.0207788	685.420459
-2.29569	2.29569	+	2.29569	0.0490526	686.42381
-3.5577	3.5577	+	3.5577	0.00845675	686.474417
-4.116	4.116	+	4.116	0.00341748	691.428547
-3.62494	3.62494	+	3.62494	0.00753957	692.431976
-3.24816	3.24816	+	3.24816	0.0146167	692.522482
-3.60159	3.60159	+	3.60159	0.00781754	693.526117
-2.4913	2.4913	+	2.4913	0.0384649	695.453688
-2.57122	2.57122	+	2.57122	0.0348137	695.462171
-2.86152	2.86152	+	2.86152	0.0235078	696.465554
-2.8374	2.8374	+	2.8374	0.0242731	698.473092
-3.21532	3.21532	+	3.21532	0.0153048	700.382028
-6.65256	6.65256	+	6.65256	0	700.488778
-6.36971	6.36971	+	6.36971	0	701.492178
-4.73836	4.73836	+	4.73836	0.00121053	702.495312
-4.15385	4.15385	+	4.15385	0.00317172	704.522486
-4.04063	4.04063	+	4.04063	0.00394393	705.525873
-7.17557	7.17557	+	7.17554	0	706.538098
-7.24651	7.24651	+	7.24647	0	707.541376
-2.55704	2.55704	+	2.55704	0.0354264	708.45742
-7.50449	7.50449	+	7.50443	0	708.544589
-5.22593	5.22593	+	5.22593	0.000556522	709.547407
-2.88873	2.88873	+	2.88873	0.0225968	712.382041
-2.76265	2.76265	+	2.76265	0.0271253	713.269155
-3.47893	3.47893	+	3.47893	0.0099802	713.385482
-2.5147	2.5147	+	2.5147	0.0372459	714.431826
-4.79948	4.79948	+	4.79948	0.00119728	714.504456
-3.08104	3.08104	+	3.08104	0.0182447	715.402459
-2.62461	2.62461	+	2.62461	0.0327559	715.435343
-5.51709	5.51709	+	5.51709	0.000235294	715.507955
-2.50366	2.50366	+	2.50366	0.0378802	716.462693

**-2.31052**	**2.31052**	+	**2.31052**	**0.0485251**	**722.473073**
**-2.76458**	**2.76458**	+	**2.76458**	**0.0270383**	**726.397595**
**-4.20783**	**4.20783**	+	**4.20783**	**0.00296447**	**726.504389**
**-4.72372**	**4.72372**	+	**4.72372**	**0.00120513**	**727.50748**
**-7.00252**	**7.00252**	+	**7.0025**	**0**	**728.519905**
**-7.00252**	**7.00252**	+	**7.0025**	**0**	**728.520084**
**-7.00308**	**7.00308**	+	**7.00307**	**0**	**729.523426**
**-7.10385**	**7.10385**	+	**7.10383**	**0**	**730.526619**
**-6.04947**	**6.04947**	+	**6.04947**	**0**	**731.529176**
**-6.05061**	**6.05061**	+	**6.05061**	**0**	**731.530622**
**-3.01993**	**3.01993**	+	**3.01993**	**0.0196915**	**732.533638**
**-2.90919**	**2.90919**	+	**2.90919**	**0.021844**	**734.559807**
**-2.66306**	**2.66306**	+	**2.66306**	**0.031022**	**735.563676**
**-3.0339**	**3.0339**	+	**3.0339**	**0.0193886**	**736.488183**
**-3.56235**	**3.56235**	+	**3.56235**	**0.00843056**	**737.520542**
**-2.72832**	**2.72832**	+	**2.72832**	**0.0280911**	**740.413335**
**-2.68353**	**2.68353**	+	**2.68353**	**0.0303707**	**742.46442**
**-2.97645**	**2.97645**	+	**2.97645**	**0.0205504**	**742.498975**
**-3.04723**	**3.04723**	+	**3.04723**	**0.0190914**	**744.494034**
**-3.75429**	**3.75429**	+	**3.75429**	**0.00613333**	**744.514821**
**-3.12463**	**3.12463**	+	**3.12463**	**0.0173552**	**745.497426**
**-2.41385**	**2.41385**	+	**2.41385**	**0.0425448**	**746.492366**
**-2.67301**	**2.67301**	+	**2.67301**	**0.0308016**	**746.500741**
**-3.41012**	**3.41012**	+	**3.41012**	**0.0111266**	**749.509259**
**-3.41012**	**3.41012**	+	**3.41012**	**0.0111619**	**749.510318**
**-3.42952**	**3.42952**	+	**3.42953**	**0.0108903**	**750.512701**
**-3.67765**	**3.67765**	+	**3.67765**	**0.00695911**	**751.51582**
**-2.63089**	**2.63089**	+	**2.63089**	**0.0324348**	**754.535859**
**-2.66265**	**2.66265**	+	**2.66265**	**0.030976**	**755.539216**
**-2.59226**	**2.59226**	+	**2.59226**	**0.0341628**	**755.542324**
**-3.60772**	**3.60772**	+	**3.60772**	**0.00778799**	**756.542435**
**-3.39086**	**3.39086**	+	**3.39086**	**0.0114847**	**757.545605**
**-5.57816**	**5.57816**	+	**5.57816**	**0.000163265**	**760.509453**
**-5.57725**	**5.57725**	+	**5.57725**	**0.000161616**	**760.510625**
**-2.8201**	**2.8201**	+	**2.8201**	**0.0247446**	**761.512885**
**-3.33237**	**3.33237**	+	**3.33237**	**0.0123929**	**768.444761**
**-4.47351**	**4.47351**	+	**4.47351**	**0.00196571**	**769.448179**
**-3.2095**	**3.2095**	+	**3.2095**	**0.0153559**	**770.530922**

-2.67855	2.67855	+	2.67855	0.0304503	772.546585
-2.54926	2.54926	+	2.54926	0.0356629	777.539103
-2.54968	2.54968	+	2.54968	0.0357223	777.540845
-3.51759	3.51759	+	3.51759	0.0090604	778.544758
-6.88792	6.88792	+	6.88791	0	779.447101
-3.07736	3.07736	+	3.07736	0.0183069	786.600867
-3.07549	3.07549	+	3.07549	0.0182902	787.60428
-3.5505	3.5505	+	3.5505	0.00856552	788.607296
-3.75931	3.75931	+	3.75931	0.00609449	789.609671
-3.16769	3.16769	+	3.16769	0.0165125	790.613107
-4.51023	4.51023	+	4.51023	0.00190698	791.627019
-2.43876	2.43876	+	2.43876	0.0415265	792.512786
-4.3685	4.3685	+	4.3685	0.0023617	792.629598
-4.36994	4.36994	+	4.36994	0.00237433	792.630804
-2.90016	2.90016	+	2.90016	0.022137	793.462779
-3.01248	3.01248	+	3.01248	0.0198473	793.52074
-2.52466	2.52466	+	2.52466	0.0366362	795.535411
-4.88639	4.88639	+	4.88639	0.00094964	801.429083
-4.89202	4.89202	+	4.89202	0.000956522	801.431189
-4.75102	4.75102	+	4.75102	0.00121854	802.43263
-3.03745	3.03745	+	3.03745	0.0193125	802.595701
-2.93464	2.93464	+	2.93464	0.0211721	804.611673
-6.71825	6.71825	+	6.71824	0	805.462858
-4.24753	4.24753	+	4.24753	0.00265979	805.48407
-6.09987	6.09987	+	6.09987	0	806.466361
-3.36564	3.36564	+	3.36564	0.011769	808.582992
-3.40252	3.40252	+	3.40252	0.0111651	809.586374
-2.84065	2.84065	+	2.84065	0.0241947	809.639014
-4.21217	4.21217	+	4.21217	0.00297959	810.589345
-5.07538	5.07538	+	5.07538	0.000661417	811.592249
-4.97574	4.97574	+	4.97574	0.000740741	812.507511
-2.34596	2.34596	+	2.34596	0.0469333	813.511442
-8.08877	8.08877	+	8.08868	0	814.523123
-8.21448	8.21448	+	8.21444	0	815.526549
-6.85526	6.85526	+	6.85525	0	816.529233
-6.87133	6.87133	+	6.87133	0	816.530608
-2.28944	2.28944	+	2.28944	0.049473	819.535486
-4.44215	4.44215	+	4.44215	0.00212429	822.49184

-2.41726	2.41726	+	2.41726	0.0424194	824.577706
-3.07805	3.07805	+	3.07805	0.0183236	825.581584
-2.51342	2.51342	+	2.51342	0.0373018	826.497496
-3.28796	3.28796	+	3.28796	0.0135789	826.523274
-2.94887	2.94887	+	2.94887	0.0208931	826.593705
-2.69586	2.69586	+	2.69586	0.0295102	827.444863
-3.43722	3.43722	+	3.43722	0.0108143	827.46606
-2.56203	2.56203	+	2.56203	0.0352424	827.597086
-4.35871	4.35871	+	4.35871	0.00237037	828.538785
-4.40594	4.40594	+	4.40594	0.00225137	828.541127
-4.99753	4.99753	+	4.99753	0.000691729	829.542714
-2.91527	2.91527	+	2.91527	0.0217367	831.478481
-3.40387	3.40387	+	3.40387	0.011185	832.482412
-4.69128	4.69128	+	4.69128	0.00124051	836.505138
-2.30205	2.30205	+	2.30205	0.0487705	836.538968
-2.36997	2.36997	+	2.36997	0.0451496	836.541068
-3.39682	3.39682	+	3.39682	0.0112941	837.508043
-2.64867	2.64867	+	2.64867	0.0317211	838.61866
-5.11669	5.11669	+	5.11669	0.0006	840.537179
-3.61274	3.61274	+	3.61274	0.00768683	840.569507
-3.08431	3.08431	+	3.08431	0.0182567	840.571779
-5.78975	5.78975	+	5.78975	4.26E-05	841.539727
-5.7987	5.7987	+	5.7987	0	841.540817
-3.1753	3.1753	+	3.1753	0.0162905	841.575112
-7.74627	7.74627	+	7.74617	0	842.552784
-2.64366	2.64366	+	2.64366	0.0319284	843.438107
-7.71531	7.71531	+	7.71521	0	843.556173
-3.64596	3.64596	+	3.64596	0.00727007	844.444872
-7.89362	7.89362	+	7.89351	0	844.559275
-7.89369	7.89369	+	7.89358	0	844.560253
-4.6519	4.6519	+	4.6519	0.00129268	845.453683
-7.78655	7.78655	+	7.78644	0	845.562163
-3.73806	3.73806	+	3.73806	0.0063876	846.456525
-4.06507	4.06507	+	4.06507	0.00386667	846.523914
-4.99463	4.99463	+	4.99463	0.000746269	846.563618
-4.71302	4.71302	+	4.71302	0.00122293	850.51891
-4.73506	4.73506	+	4.73506	0.00120261	850.521223
-5.26131	5.26131	+	5.26131	0.000460177	852.573835

-4.87599	4.87599	+	4.87599	0.000964539	853.576984
-5.18053	5.18053	+	5.18053	0.000551724	858.547797
-6.67681	6.67681	+	6.67681	0	859.529187
-6.67636	6.67636	+	6.67635	0	859.530323
-5.89901	5.89901	+	5.89901	0	860.532693
-3.74359	3.74359	+	3.74359	0.0063125	861.513321
-2.82029	2.82029	+	2.82029	0.0247809	863.526813
-4.72618	4.72618	+	4.72618	0.0012129	864.534901
-3.88638	3.88638	+	3.88638	0.005	865.53826
-3.88926	3.88926	+	3.88926	0.00504803	865.540645
-3.15823	3.15823	+	3.15823	0.016584	866.541318
-2.59312	2.59312	+	2.59312	0.0341981	866.659235
-2.59197	2.59197	+	2.59197	0.0341277	866.660516
-3.53345	3.53345	+	3.53345	0.00876976	867.663327
-3.66366	3.66366	+	3.66366	0.00695971	868.50615
-2.49235	2.49235	+	2.49235	0.0384549	868.56844
-2.4176	2.4176	+	2.4176	0.0424653	868.676315
-2.48339	2.48339	+	2.48339	0.0386786	869.571829
-4.9224	4.9224	+	4.9224	0.000823529	870.574461
-2.63835	2.63835	+	2.63835	0.0321508	870.69316
-4.65155	4.65155	+	4.65155	0.00128485	871.576758
-2.87371	2.87371	+	2.87371	0.023036	871.688355
-3.1387	3.1387	+	3.1387	0.0170879	872.538735
-2.8542	2.8542	+	2.8542	0.02368	872.691837
-3.61142	3.61142	+	3.61142	0.00767376	873.52366
-4.79621	4.79621	+	4.79621	0.00118919	873.533949
-3.27103	3.27103	+	3.27103	0.014	873.694346
-4.11939	4.11939	+	4.11939	0.00346798	874.537259
-7.07102	7.07102	+	7.071	0	874.555164
-2.96976	2.96976	+	2.96976	0.0204866	875.503086
-5.84238	5.84238	+	5.84238	0	875.558483
-4.82075	4.82075	+	4.82075	0.00110345	876.495923
-2.97717	2.97717	+	2.97717	0.0205813	877.498645
-3.01323	3.01323	+	3.01323	0.0198673	877.501043
-3.03421	3.03421	+	3.03421	0.0194078	879.496021
-6.4971	6.4971	+	6.4971	0	880.529313
-6.49373	6.49373	+	6.49373	0	880.530472
-2.93484	2.93484	+	2.93484	0.0212617	881.489645

-2.93476	2.93476	+	2.93476	0.0212214	881.490585
-5.45512	5.45512	+	5.45512	0.000342857	881.511548
-6.27405	6.27405	+	6.27405	0	881.537007
-2.8662	2.8662	+	2.8662	0.0232915	882.493752
-4.86496	4.86496	+	4.86496	0.000985915	882.514869
-6.19836	6.19836	+	6.19837	0	882.539217
-6.16807	6.16807	+	6.16807	0	882.541381
-2.66077	2.66077	+	2.66077	0.0310339	883.517967
-3.36801	3.36801	+	3.36801	0.0117805	884.563386
-3.80347	3.80347	+	3.80347	0.00569106	885.523677
-5.40717	5.40717	+	5.40717	0.000336449	885.545026
-2.31376	2.31376	+	2.31376	0.0484553	885.566874
-4.13941	4.13941	+	4.13941	0.0033	886.527238
-5.01484	5.01484	+	5.01484	0.00070229	886.548361
-6.95511	6.95511	+	6.9551	0	887.559376
-6.95167	6.95167	+	6.95166	0	887.560893
-5.59594	5.59594	+	5.59594	8.33E-05	888.564165
-4.73157	4.73157	+	4.73157	0.00122078	889.542185
-2.9881	2.9881	+	2.9881	0.0204788	889.6028
-2.88116	2.88116	+	2.88116	0.0227873	890.606151
-3.76748	3.76748	+	3.76748	0.006048	892.675403
-3.51844	3.51844	+	3.51844	0.00913898	893.677485
-3.72064	3.72064	+	3.72064	0.00661538	894.691193
-2.4387	2.4387	+	2.4387	0.0414603	895.694062
-6.08395	6.08395	+	6.08395	0	896.537502
-2.58848	2.58848	+	2.58848	0.0342351	897.485184
-5.00029	5.00029	+	5.00029	0.00069697	897.541547
-2.32197	2.32197	+	2.32197	0.0481311	898.488823
-2.30846	2.30846	+	2.30846	0.0486139	898.491568
-2.96022	2.96022	+	2.96022	0.0207614	898.541893
-3.40563	3.40563	+	3.40563	0.0111924	901.503867
-3.00943	3.00943	+	3.00943	0.0199289	901.518397
-3.84527	3.84527	+	3.84527	0.00521162	902.505947
-5.16648	5.16648	+	5.16648	0.000547009	902.511553
-6.39373	6.39373	+	6.39373	0	903.518678
-6.39878	6.39878	+	6.39879	0	903.520452
-4.38646	4.38646	+	4.38646	0.00233514	903.533794
-6.38415	6.38415	+	6.38415	0	904.527288

-6.40926	6.40926	+	6.40926	0	905.529766
-6.40908	6.40908	+	6.40908	0	905.530666
-5.81459	5.81459	+	5.81459	0	906.53376
-3.41055	3.41055	+	3.41055	0.0111847	906.54555
-5.07796	5.07796	+	5.07796	0.000634921	907.548846
-5.08285	5.08285	+	5.08285	0.00064	907.552372
-4.37133	4.37133	+	4.37133	0.0023871	911.584193
-2.9975	2.9975	+	2.9975	0.0202116	913.576541
-4.78924	4.78924	+	4.78924	0.00117333	915.461791
-2.83699	2.83699	+	2.83699	0.024193	915.556581
-5.41142	5.41142	+	5.41142	0.000339623	916.465167
-3.01828	3.01828	+	3.01828	0.0197231	920.499259
-3.0182	3.0182	+	3.0182	0.0196726	920.501223
-3.43115	3.43115	+	3.43115	0.0108867	920.70669
-2.56443	2.56443	+	2.56443	0.0351651	921.504562
-3.39189	3.39189	+	3.39189	0.0114831	922.61545
-3.40026	3.40026	+	3.40026	0.0111677	923.618826
-7.66158	7.66158	+	7.66149	0	924.619593
-7.66157	7.66157	+	7.66148	0	924.621132
-3.93941	3.93941	+	3.93941	0.00473543	925.623998
-4.45904	4.45904	+	4.45904	0.00197727	926.625996
-2.92799	2.92799	+	2.92799	0.0213179	927.522872
-4.03166	4.03166	+	4.03166	0.00394419	929.534227
-2.42168	2.42168	+	2.42168	0.0423206	930.542955
-2.34755	2.34755	+	2.34755	0.0468047	931.546529
-2.53628	2.53628	+	2.53628	0.0363488	931.550918
-3.72637	3.72637	+	3.72637	0.00650193	932.547282
-3.27375	3.27375	+	3.27375	0.0139359	938.535077
-4.05535	4.05535	+	4.05535	0.00384977	938.609391
-4.06075	4.06075	+	4.06075	0.00388626	938.610656
-2.97976	2.97976	+	2.97976	0.020665	940.627234
-5.4705	5.4705	+	5.4705	0.000349515	941.478494
-5.13694	5.13694	+	5.13694	0.000576271	942.482043
-5.30781	5.30781	+	5.30781	0.000468468	950.538934
-5.3179	5.3179	+	5.3179	0.000472727	950.540638
-3.60231	3.60231	+	3.60231	0.0078169	950.574787
-6.49894	6.49894	+	6.49894	0	951.542419
-3.29868	3.29868	+	3.29868	0.0134	952.653776

-4.43279	4.43279	+	4.43279	0.00212291	953.598322
-5.09566	5.09566	+	5.09566	0.000585366	954.605226
-4.11768	4.11768	+	4.11768	0.00343415	961.597606
-5.2674	5.2674	+	5.2674	0.000464286	961.601049
-3.76416	3.76416	+	3.76416	0.00600791	962.607388
-2.72072	2.72072	+	2.72072	0.0283967	962.610605
-2.90717	2.90717	+	2.90717	0.0219039	964.554037
-2.32996	2.32996	+	2.32996	0.0477426	967.494148
-2.95035	2.95035	+	2.95035	0.0208476	967.514948
-3.08545	3.08545	+	3.08545	0.0182627	968.49773
-5.46376	5.46376	+	5.46376	0.000346154	977.556711
-8.02294	8.02294	+	8.02283	0	978.56973
-8.02299	8.02299	+	8.02289	0	978.570376
-8.21768	8.21768	+	8.21763	0	979.573617
-8.05234	8.05234	+	8.05224	0	980.576572
-3.86226	3.86226	+	3.86226	0.00519149	980.695117
-4.11305	4.11305	+	4.11305	0.00342029	981.469477
-4.11067	4.11067	+	4.11067	0.00340385	981.471283
-6.80463	6.80463	+	6.80462	0	981.579149
-6.79945	6.79945	+	6.79945	0	981.580728
-2.41177	2.41177	+	2.41177	0.0425955	981.698197
-4.4204	4.4204	+	4.4204	0.0022	982.474652
-2.59434	2.59434	+	2.59434	0.0342257	982.543991
-3.08322	3.08322	+	3.08322	0.0182507	983.579386
-3.10971	3.10971	+	3.10971	0.01775	983.582365
-2.49679	2.49679	+	2.49679	0.0381989	984.494891
-3.3212	3.3212	+	3.3212	0.0127003	984.589662
-3.3212	3.3212	+	3.3212	0.0126627	984.591244
-3.23917	3.23917	+	3.23917	0.0146819	985.594726
-6.6754	6.6754	+	6.67539	0	986.595993
-3.11503	3.11503	+	3.11503	0.0176894	987.597783
-6.05779	6.05779	+	6.05779	0	988.58898
-6.01134	6.01134	+	6.01134	0	988.591236
-3.09124	3.09124	+	3.09124	0.0181505	989.594073
-3.41484	3.41484	+	3.41484	0.0111438	990.531848
-4.54175	4.54175	+	4.54175	0.00175148	990.567347
-5.57851	5.57851	+	5.57851	0.000164948	994.565005
-7.50892	7.50892	+	7.50885	0	995.546653

-3.63865	3.63865	+	3.63865	0.00737681	995.567895
-6.93193	6.93193	+	6.93192	0	996.549385
-6.92164	6.92164	+	6.92163	0	996.550905
-4.68264	4.68264	+	4.68264	0.00128302	1000.55179
-3.26106	3.26106	+	3.26106	0.0142261	1000.58615
-3.33964	3.33964	+	3.33964	0.012203	1001.55504
-2.93932	2.93932	+	2.93932	0.0210492	1002.56834
-3.21069	3.21069	+	3.21069	0.0153314	1002.57444
-2.3756	2.3756	+	2.3756	0.044633	1005.5892
-2.37607	2.37607	+	2.37607	0.0446071	1005.5903
-2.91539	2.91539	+	2.91539	0.0217685	1006.5893
-2.57357	2.57357	+	2.57357	0.0348604	1006.59471
-4.26921	4.26921	+	4.26921	0.00263874	1006.70943
-4.2695	4.2695	+	4.26949	0.00265263	1006.711
-2.48535	2.48535	+	2.48535	0.0386738	1007.48654
-3.50615	3.50615	+	3.50615	0.00935117	1007.7141
-5.08845	5.08845	+	5.08845	0.000645161	1008.72637
-7.69294	7.69294	+	7.69284	0	1010.57277
-2.97723	2.97723	+	2.97723	0.0206321	1011.51874
-2.96706	2.96706	+	2.96706	0.0204541	1011.52099
-6.64114	6.64114	+	6.64113	0	1011.57634
-4.65614	4.65614	+	4.65614	0.00125153	1012.51343
-4.57184	4.57184	+	4.57184	0.00165269	1012.54885
-4.53457	4.53457	+	4.53457	0.00178824	1012.55104
-2.39447	2.39447	+	2.39447	0.0436928	1012.62268
-3.0087	3.0087	+	3.0087	0.0198987	1013.55361
-2.31468	2.31468	+	2.31468	0.048443	1013.62629
-3.18617	3.18617	+	3.18617	0.0161461	1015.57343
-4.40798	4.40798	+	4.40798	0.00226374	1016.54522
-4.25125	4.25125	+	4.25125	0.00267358	1017.52897
-4.25446	4.25446	+	4.25446	0.00266667	1017.53073
-3.67239	3.67239	+	3.67239	0.00699259	1017.55362
-3.77182	3.77182	+	3.77182	0.00603226	1018.53379
-7.36778	7.36778	+	7.36774	0	1018.56288
-3.1384	3.1384	+	3.1384	0.0170411	1019.54659
-7.17216	7.17216	+	7.17213	0	1019.56624
-5.94005	5.94005	+	5.94005	0	1020.56889
-5.9369	5.9369	+	5.93691	0	1020.57084

-4.68134	4.68134	+	4.68134	0.001275	1021.56222
-4.49758	4.49758	+	4.49758	0.00193103	1022.56609
-6.63318	6.63318	+	6.63318	0	1023.5781
-6.29176	6.29176	+	6.29176	0	1024.58186
-2.81086	2.81086	+	2.81086	0.0250517	1025.58488
-2.70288	2.70288	+	2.70288	0.0292131	1030.63739
-3.61918	3.61918	+	3.61918	0.0076129	1032.55476
-2.59043	2.59043	+	2.59043	0.0341853	1034.74222
-2.52582	2.52582	+	2.52582	0.0365934	1037.53597
-4.03125	4.03125	+	4.03125	0.00392593	1038.52823
-4.67607	4.67607	+	4.67607	0.00125926	1039.53486
-3.7658	3.7658	+	3.7658	0.00601587	1039.54682
-3.69506	3.69506	+	3.69506	0.00681818	1039.55197
-6.58488	6.58488	+	6.58487	0	1040.54459
-6.37687	6.37687	+	6.37687	0	1041.54802
-5.04402	5.04402	+	5.04402	0.000682171	1042.54939
-5.04318	5.04318	+	5.04318	0.000676923	1042.55122
-3.96376	3.96376	+	3.96376	0.0046055	1043.56779
-3.70115	3.70115	+	3.70115	0.00673764	1043.57232
-2.97746	2.97746	+	2.97746	0.0206832	1044.57847
-2.97567	2.97567	+	2.97567	0.0205098	1044.58034
-3.02615	3.02615	+	3.02615	0.0195969	1045.57941
-2.99524	2.99524	+	2.99524	0.0202412	1045.58208
-2.42022	2.42022	+	2.42022	0.0423304	1049.59344
-3.37271	3.37271	+	3.37271	0.0117554	1051.47833
-2.83838	2.83838	+	2.83838	0.0242561	1052.50553
-2.48788	2.48788	+	2.48788	0.0386619	1056.51865
-2.48581	2.48581	+	2.48581	0.0387217	1056.52037
-2.7345	2.7345	+	2.7345	0.0280418	1057.5193
-2.73124	2.73124	+	2.73124	0.0281333	1057.52201
-2.85772	2.85772	+	2.85772	0.0236347	1058.52497
-2.53445	2.53445	+	2.53445	0.0363026	1058.63278
-2.45831	2.45831	+	2.45831	0.0401133	1059.63616
-6.40961	6.40961	+	6.40961	0	1060.63783
-3.6891	3.6891	+	3.6891	0.00687218	1065.54835
-3.64154	3.64154	+	3.64154	0.00733091	1065.55252
-3.10833	3.10833	+	3.10833	0.0177453	1066.5596
-3.10817	3.10817	+	3.10817	0.0176973	1066.56083

-3.09231	3.09231	+	3.09231	0.0181671	1067.56427
-2.40497	2.40497	+	2.40497	0.0431149	1070.62849
-3.85156	3.85156	+	3.85156	0.00515	1074.62784
-3.86097	3.86097	+	3.86097	0.00516949	1074.6303
-2.40182	2.40182	+	2.40182	0.0433151	1075.62895
-2.40152	2.40152	+	2.40152	0.0432684	1075.63135
-3.70805	3.70805	+	3.70805	0.00671264	1077.49486
-2.52221	2.52221	+	2.52221	0.0367445	1077.64744
-3.45282	3.45282	+	3.45282	0.0105311	1078.4981
-4.68083	4.68083	+	4.68083	0.00126708	1078.51696
-4.89618	4.89618	+	4.89618	0.000934307	1078.52173
-5.35275	5.35275	+	5.35275	0.000481481	1079.52866
-5.34136	5.34136	+	5.34136	0.000477064	1079.53056
-6.07401	6.07401	+	6.07401	0	1080.53733
-5.52671	5.52671	+	5.52671	0.000158416	1081.53947
-5.5271	5.5271	+	5.5271	0.00016	1081.54108
-4.1204	4.1204	+	4.1204	0.00344279	1082.54435
-4.05576	4.05576	+	4.05576	0.00386792	1086.55602
-2.63549	2.63549	+	2.63549	0.0322614	1086.66434
-3.22289	3.22289	+	3.22289	0.0150743	1087.59457
-3.43384	3.43384	+	3.43384	0.0108701	1092.6128
-3.62976	3.62976	+	3.62976	0.0074657	1093.6165
-2.90995	2.90995	+	2.90995	0.0218575	1095.50399
-2.57189	2.57189	+	2.57189	0.0348779	1096.50925
-2.57144	2.57144	+	2.57144	0.0348571	1096.51138
-2.31307	2.31307	+	2.31307	0.0484091	1098.5166
-3.52795	3.52795	+	3.52795	0.00886301	1098.62609
-3.51812	3.51812	+	3.51812	0.00909091	1099.62926
-3.51819	3.51819	+	3.51819	0.00912162	1099.63031
-6.18144	6.18144	+	6.18144	0	1100.62933
-5.9645	5.9645	+	5.9645	0	1100.63377
-2.71157	2.71157	+	2.71157	0.0286735	1104.53693
-3.1629	3.1629	+	3.1629	0.0164972	1105.54467
-2.34036	2.34036	+	2.34036	0.0470265	1111.5885
-2.38283	2.38283	+	2.38283	0.0442369	1111.592
-6.82945	6.82945	+	6.82945	0	1114.58635
-2.79293	2.79293	+	2.79293	0.0257253	1114.61851
-2.72984	2.72984	+	2.72984	0.0281164	1114.62141

-6.37118	6.37118	+	6.37118	0	1115.58926
-6.37243	6.37243	+	6.37243	0	1115.59059
-7.05371	7.05371	+	7.05369	0	1116.59361
-2.57374	2.57374	+	2.57374	0.0348966	1116.63601
-3.8927	3.8927	+	3.8927	0.00501754	1117.48737
-3.00511	3.00511	+	3.00511	0.0199697	1120.60778
-2.99089	2.99089	+	2.99089	0.0204712	1121.60966
-2.99065	2.99065	+	2.99065	0.02043	1121.6113
-5.23681	5.23681	+	5.23681	0.000526316	1122.61359
-2.97489	2.97489	+	2.97489	0.0204792	1124.60613
-4.39835	4.39835	+	4.39835	0.00230435	1126.58405
-2.69815	2.69815	+	2.69815	0.0294233	1126.65718
-2.41483	2.41483	+	2.41483	0.0425147	1127.58706
-4.52461	4.52461	+	4.52461	0.00187135	1131.55916
-4.5536	4.5536	+	4.5536	0.00171429	1131.56194
-3.92465	3.92465	+	3.92465	0.00483556	1136.60213
-2.63015	2.63015	+	2.63015	0.0323866	1137.60484
-2.6222	2.6222	+	2.6222	0.032833	1141.60568
-3.24228	3.24228	+	3.24228	0.0146437	1146.58619
-3.40319	3.40319	+	3.40319	0.0111875	1147.59222
-3.93682	3.93682	+	3.93682	0.00475	1153.57443
-6.71641	6.71641	+	6.71641	0	1154.57919
-6.71625	6.71625	+	6.71625	0	1154.58046
-6.4291	6.4291	+	6.4291	0	1155.58286
-5.11144	5.11144	+	5.11144	0.000590164	1156.586
-3.94482	3.94482	+	3.94482	0.00473874	1159.58927
-3.94829	3.94829	+	3.94829	0.00472398	1159.59214
-3.52683	3.52683	+	3.52683	0.00894198	1160.59936
-3.52674	3.52674	+	3.52674	0.00891156	1160.60065
-3.17582	3.17582	+	3.17582	0.0163249	1161.60412
-4.81852	4.81852	+	4.81852	0.00109589	1162.60716
-2.37775	2.37775	+	2.37775	0.0445541	1171.55633
-2.83737	2.83737	+	2.83737	0.0242198	1176.55891
-2.86114	2.86114	+	2.86114	0.0234911	1176.56171
-3.84167	3.84167	+	3.84167	0.00522314	1177.5637
-2.56106	2.56106	+	2.56106	0.0352212	1178.57671
-2.82608	2.82608	+	2.82608	0.0246812	1179.58618
-2.95367	2.95367	+	2.95367	0.0207828	1180.59516

**-2.94436**	**2.94436**	+	**2.94436**	**0.0210024**	**1181.59849**
**-2.94095**	**2.94095**	+	**2.94095**	**0.0210635**	**1181.60061**
**-3.21207**	**3.21207**	+	**3.21207**	**0.0153409**	**1193.5378**
**-2.38703**	**2.38703**	+	**2.38703**	**0.0439593**	**1196.65657**

Supplementary Table 2: Posthoc test BUVEC for infected cells in negative-ion mode

Control	Infected	C: ANOVA Significant	N: -Log ANOVA p value	N: ANOVA q-value	MALDI m/z
**-205,988**	**205,988**	+	**205,988**	**0,0459015**	**252.9066284**
**-207,053**	**207,053**	+	**207,053**	**0,0455799**	**257.1123665**
**-226,754**	**226,754**	+	**226,754**	**0,0369461**	**272.9828792**
**-217,322**	**217,322**	+	**217,322**	**0,0410437**	**273.008503**
**-219,945**	**219,945**	+	**219,945**	**0,0400697**	**273.0238352**
**-19,963**	**19,963**	+	**19,963**	**0,0487667**	**273.9438563**
**-204,925**	**204,925**	+	**204,925**	**0,0464253**	**274.1882706**
**-244,468**	**244,468**	+	**244,468**	**0,0313129**	**276.0260536**
**-242,646**	**242,646**	+	**242,646**	**0,0318891**	**286.058508**
**-220,582**	**220,582**	+	**220,582**	**0,0398155**	**288.9152702**
**-200,539**	**200,539**	+	**200,539**	**0,048334**	**289.0061826**
**-22,891**	**22,891**	+	**22,891**	**0,0360247**	**309.9951364**
**-215,141**	**215,141**	+	**215,141**	**0,0416485**	**325.9897952**
**-285,625**	**285,625**	+	**285,625**	**0,0206638**	**325.9902574**
**-289,951**	**289,951**	+	**289,951**	**0,0188142**	**331.1319174**
**-254,539**	**254,539**	+	**254,539**	**0,02913**	**336.9963962**
**-287,409**	**287,409**	+	**287,409**	**0,0200346**	**341.0381952**
**-199,619**	**199,619**	+	**199,619**	**0,048735**	**345.1477908**
**-237,643**	**237,643**	+	**237,643**	**0,033233**	**348.1058788**
**-263,246**	**263,246**	+	**263,246**	**0,0276776**	**349.1139472**
**-230,764**	**230,764**	+	**230,764**	**0,0356469**	**356.0446788**
**-218,173**	**218,173**	+	**218,173**	**0,040846**	**357.0345737**
**-217,756**	**217,756**	+	**217,756**	**0,040832**	**359.0920484**
**-31,231**	**31,231**	+	**31,231**	**0,0114894**	**361.1057399**
**-20,825**	**20,825**	+	**20,825**	**0,0450413**	**362.1572749**
**-254,771**	**254,771**	+	**254,771**	**0,0290025**	**363.1947318**
**-200,212**	**200,212**	+	**200,212**	**0,0486353**	**367.988699**
**-206,621**	**206,621**	+	**206,621**	**0,0456277**	**371.9651817**

-207,789	207,789	+	207,789	0,0453689	373.9493953
-226,045	226,045	+	226,045	0,0374405	374.0358092
-237,291	237,291	+	237,291	0,0333096	378.1596144
-226,295	226,295	+	226,295	0,0373254	379.9018406
-303,021	303,021	+	303,021	0,01426	382.1065825
-260,201	260,201	+	260,201	0,0281337	384.1043078
-253,973	253,973	+	253,973	0,0288802	390.214243
-224,543	224,543	+	224,543	0,0382591	393.9972479
-267,908	267,908	+	267,908	0,0264466	394.9812384
-213,088	213,088	+	213,088	0,043023	406.9305783
-209,925	209,925	+	209,925	0,0441876	408.9274203
-237,971	237,971	+	237,971	0,0332514	411.0963605
-251,791	251,791	+	251,791	0,0292028	419.2482485
-234,018	234,018	+	234,018	0,034585	431.2575854
-248,184	248,184	+	248,184	0,030337	443.2575328
-238,521	238,521	+	238,521	0,0331335	444.2609521
-273,413	273,413	+	273,413	0,0254348	452.1248235
-247,748	247,748	+	247,748	0,030308	456.1440745
-232,242	232,242	+	232,242	0,0351287	460.2088339
-216,907	216,907	+	216,907	0,0410357	461.0718022
-206,839	206,839	+	206,839	0,0455522	462.0795965
-208,628	208,628	+	208,628	0,0447856	464.0723679
-269,058	269,058	+	269,058	0,0261914	465.0780014
-318,128	318,128	+	318,128	0,0103218	468.1194913
-262,068	262,068	+	262,068	0,0279417	468.1429905
-265,611	265,611	+	265,611	0,0269408	472.1382548
-200,068	200,068	+	200,068	0,0487084	472.1458147
-223,965	223,965	+	223,965	0,0382726	474.018753
-20,613	20,613	+	20,613	0,0459634	474.0203614
-198,947	198,947	+	198,947	0,0487985	474.0321373
-207,201	207,201	+	2,072	0,0457181	475.13763
-244,955	244,955	+	244,955	0,0311653	475.983303
-249,756	249,756	+	249,756	0,0297054	476.1429806
-223,511	223,511	+	223,511	0,0382493	476.2039909
-219,073	219,073	+	219,073	0,0403974	477.0659963
-284,311	284,311	+	284,311	0,0212437	478.0174201
-224,406	224,406	+	224,406	0,0381534	478.073308
-225,793	225,793	+	225,793	0,0374357	481.1149564

-219,248	219,248	+	219,248	0,0404096	482.0029107
-206,972	206,972	+	206,972	0,0456437	482.0136267
-224,999	224,999	+	224,999	0,0380498	483.1255415
-212,441	212,441	+	212,441	0,0433141	485.1413733
-325,964	325,964	+	325,964	0,008975	486.1541948
-250,552	250,552	+	250,552	0,0295113	486.2248007
-247,442	247,442	+	247,442	0,0303966	488.1329876
-229,026	229,026	+	229,026	0,0360558	488.140954
-212,817	212,817	+	212,817	0,0431384	489.01265
-262,719	262,719	+	262,719	0,027705	494.0683131
-225,951	225,951	+	225,951	0,0374859	494.0714124
-199,835	199,835	+	199,835	0,048622	495.0536705
-197,391	197,391	+	197,391	0,0499457	496.0850146
-21,913	21,913	+	21,913	0,0404568	497.1097137
-247,071	247,071	+	247,071	0,0304582	497.1345124
-256,603	256,603	+	256,603	0,0290105	498.117307
-238,371	238,371	+	238,371	0,0331898	499.1205021
-218,037	218,037	+	218,037	0,0406848	501.0117298
-27,634	27,634	+	27,634	0,0244242	501.1364143
-20,275	20,275	+	20,275	0,0471197	502.1206233
-21,063	21,063	+	21,063	0,0439582	502.1489096
-316,596	316,596	+	316,596	0,0104444	502.1491062
-228,183	228,183	+	228,183	0,0362504	502.2194061
-240,932	240,932	+	240,932	0,0327582	502.2195957
-253,565	253,565	+	253,565	0,0289104	503.1522698
-199,169	199,169	+	199,169	0,0489319	511.1124791
-323,878	323,878	+	323,878	0,00916364	513.1284925
-239,291	239,291	+	239,291	0,0330448	513.1295621
-28,128	28,128	+	28,128	0,0222169	515.2785154
-302,913	302,913	+	302,913	0,0143284	515.2787414
-278,332	278,332	+	278,332	0,0236576	516.2821655
-247,194	247,194	+	247,194	0,0305548	517.1311762
-201,482	201,482	+	201,482	0,0479321	518.1434021
-214,389	214,389	+	21,439	0,041978	529.1232916
-256,016	256,016	+	256,016	0,0291013	529.2942844
-211,337	211,337	+	211,337	0,0436898	531.1396586
-266,693	266,693	+	266,693	0,026859	531.141322
-223,169	223,169	+	223,169	0,038244	533.1263203

-276,201	276,201	+	276,201	0,0244075	534.139383
-228,653	228,653	+	228,653	0,0360307	535.142007
-276,551	276,551	+	276,551	0,0244106	537.2739011
-19,901	19,901	+	19,901	0,0487536	543.3106003
-204,691	204,691	+	204,691	0,0464017	543.3106003
-218,903	218,903	+	218,903	0,0405397	545.1185561
-475,856	475,856	+	475,856	0,000193548	553.2692686
-32,524	32,524	+	32,524	0,00904348	553.2790725
-36,607	36,607	+	36,607	0,00344715	553.2790725
-338,297	338,297	+	338,297	0,00682993	554.2830253
-218,176	218,176	+	218,176	0,0408993	557.3259241
-222,763	222,763	+	222,763	0,03839	564.1316734
-220,487	220,487	+	220,487	0,0397351	567.1009992
-281,166	281,166	+	281,166	0,02216	569.274807
-361,344	361,344	+	361,344	0,00381395	571.2901028
-412,046	412,046	+	412,046	0,00123077	571.2901028
-279,081	279,081	+	279,081	0,0232784	571.3416585
-408,171	408,171	+	408,171	0,00124731	572.2938431
-231,034	231,034	+	231,034	0,0356888	572.3452777
-222,969	222,969	+	222,969	0,0383329	575.1808179
-315,528	315,528	+	315,528	0,0107845	575.2522336
-252,223	252,223	+	252,223	0,0291698	580.2988178
-220,314	220,314	+	220,313	0,039892	584.1043428
-23,505	23,505	+	23,505	0,0340103	585.1115369
-240,456	240,456	+	240,456	0,032687	588.1289421
-261,107	261,107	+	261,107	0,0283782	588.1512219
-22,948	22,948	+	22,948	0,0359875	590.1649992
-199,204	199,204	+	199,204	0,048929	591.1506223
-232,664	232,664	+	232,664	0,0349502	591.1757614
-230,997	230,997	+	230,997	0,0356828	592.1803017
-198,665	198,665	+	198,665	0,0489242	599.373237
-233,369	233,369	+	233,369	0,0348465	601.0508018
-302,015	302,015	+	302,015	0,0144706	602.1556091
-254,509	254,509	+	254,509	0,0290149	604.1457884
-344,198	344,198	+	344,198	0,00591429	604.1718096
-261,112	261,112	+	261,112	0,0284598	606.1605065
-214,764	214,764	+	214,764	0,041786	608.0467438
-268,466	268,466	+	268,466	0,0263475	608.1745801

-199,293	199,293	+	199,293	0,0488338	616.1144765
-749,788	749,788	+	749,782	0	617.4213867
-760,597	760,597	+	760,589	0	617.4213867
-203,824	203,824	+	203,824	0,0468767	618.1502296
-237,791	237,791	+	237,791	0,0332374	618.1502428
-633,122	633,122	+	633,122	0	619.4361133
-220,277	220,277	+	220,277	0,0398544	620.1407579
-332,027	332,027	+	332,027	0,00789474	620.1663537
-582,766	582,766	+	582,766	0	620.4392552
-586,483	586,483	+	586,483	0	620.4407689
-198,264	198,264	+	198,264	0,0492113	621.1710094
-224,451	224,451	+	224,451	0,0381507	622.1554368
-318,027	318,027	+	318,027	0,0102857	622.1830302
-232,819	232,819	+	232,819	0,0349533	623.1841416
-206,038	206,038	+	206,038	0,0458778	624.1701555
-218,285	218,285	+	218,285	0,0408115	625.301645
-244,719	244,719	+	244,719	0,0312675	625.3896525
-246,129	246,129	+	246,129	0,0307453	626.1836846
-201,605	201,605	+	201,605	0,0478358	627.404676
-3,554	3,554	+	3,554	0,00442105	631.4367786
-692,177	692,177	+	692,176	0	633.4521849
-215,186	215,186	+	215,186	0,0417689	634.1453808
-725,368	725,368	+	725,365	0	634.4535522
-238,839	238,839	+	238,839	0,033321	635.1501947
-210,811	210,811	+	210,811	0,0438789	636.1357289
-267,423	267,423	+	267,423	0,026671	636.1613504
-249,412	249,412	+	249,412	0,0299065	637.164995
-255,599	255,599	+	255,599	0,0289514	637.263916
-273,855	273,855	+	273,855	0,0252601	638.177695
-262,051	262,051	+	262,051	0,0278605	638.1787268
-270,507	270,507	+	270,507	0,0258847	639.1814765
-283,262	283,262	+	283,262	0,0219167	639.4044036
-199,961	199,961	+	199,961	0,0485965	640.1652213
-225,221	225,221	+	225,221	0,0378886	640.1944568
-236,863	236,863	+	236,863	0,0333968	641.4209362
-511,285	511,285	+	511,285	7.84E+00	643.4362074
-370,075	370,075	+	370,075	0,00317355	644.4398804
-479,772	479,772	+	479,772	0,0002	645.4517773

-614,353	614,353	+	614,353	0	646.4554777
-475,899	475,899	+	475,899	0,000196721	647.4578277
-214,483	214,483	+	214,483	0,042	649.1582461
-272,452	272,452	+	272,452	0,0257367	651.3171947
-217,172	217,172	+	217,172	0,0410192	652.1567309
-314,781	314,781	+	314,781	0,0109348	652.3233643
-241,446	241,446	+	241,446	0,032537	654.172621
-260,897	260,897	+	260,897	0,02825	655.1752452
-211,766	211,766	+	211,766	0,0435492	655.4373002
-21,841	21,841	+	21,841	0,0408474	656.1889696
-260,894	260,894	+	260,894	0,02817	656.1893628
-197,424	197,424	+	197,424	0,0499551	657.1918327
-200,395	200,395	+	200,395	0,048526	657.2352277
-220,609	220,609	+	220,609	0,0398587	660.4711649
-271,661	271,661	+	271,661	0,0255972	662.3240247
-232,279	232,279	+	232,279	0,0351537	663.2823836
-376,053	376,053	+	376,053	0,00274783	665.3313982
-253,624	253,624	+	253,624	0,028932	666.3016431
-212,176	212,176	+	212,176	0,0434552	667.1697022
-215,561	215,561	+	215,561	0,0414782	670.1680251
-242,063	242,063	+	242,063	0,0324348	670.1684511
-237,523	237,523	+	237,523	0,0332451	671.171538
-197,966	197,966	+	197,966	0,0495104	671.4677303
-21,718	21,718	+	21,718	0,0410615	671.4679745
-227,747	227,747	+	227,747	0,0364863	672.1838656
-257,774	257,774	+	257,774	0,028973	672.4712289
-265,671	265,671	+	265,671	0,0270721	672.4712289
-275,991	275,991	+	275,991	0,0244361	673.1837494
-197,527	197,527	+	197,527	0,0498632	674.1392253
-203,757	203,757	+	203,757	0,0467534	674.1396533
-230,055	230,055	+	230,055	0,0356157	675.1447261
-227,927	227,927	+	227,927	0,0363836	676.1553493
-258,372	258,372	+	258,372	0,029011	676.3031057
-212,567	212,567	+	212,567	0,0433029	679.3471256
-22,708	22,708	+	22,708	0,0367759	679.3478133
-204,275	204,275	+	204,275	0,0468225	681.1487165
-258,057	258,057	+	258,057	0,0290136	681.364563
-233,694	233,694	+	233,694	0,0345946	682.1928266

-22,353	22,353	+	22,353	0,0382865	684.2121028
-224,456	224,456	+	224,456	0,0382061	685.179622
-240,313	240,313	+	240,313	0,0325369	685.1798479
-207,171	207,171	+	207,171	0,0456352	685.2196759
-202,958	202,958	+	202,958	0,0470234	686.1632948
-249,855	249,855	+	249,855	0,0297092	686.1824517
-221,806	221,806	+	221,806	0,0391007	689.296395
-202,924	202,924	+	202,924	0,047	690.1345616
-312,631	312,631	+	312,631	0,0114866	690.5091403
-31,492	31,492	+	31,492	0,0109727	690.5091403
-549,787	549,787	+	549,787	0	691.3132312
-245,085	245,085	+	245,085	0,0311056	691.511391
-220,167	220,167	+	220,167	0,0398443	692.1507212
-628,358	628,358	+	628,358	0	692.3172702
-329,397	329,397	+	329,397	0,00835669	693.3647088
-253,986	253,986	+	253,986	0,0289412	696.1983158
-230,108	230,108	+	230,108	0,0356461	699.1588533
-210,469	210,469	+	210,469	0,043903	700.1618092
-253,548	253,548	+	253,548	0,0288406	700.2305761
-21,795	21,795	+	21,795	0,0407098	701.1740647
-223,281	223,281	+	223,281	0,0382447	702.1748633
-210,976	210,976	+	210,976	0,0437203	703.1306505
-263,204	263,204	+	263,204	0,027631	703.4097342
-262,591	262,591	+	262,591	0,0277059	703.4097342
-26,406	26,406	+	26,406	0,0275976	704.1364091
-290,896	290,896	+	290,896	0,0185291	704.4145869
-230,673	230,673	+	230,673	0,0355847	705.1465588
-215,976	215,976	+	215,976	0,0412965	709.1931678
-199,129	199,129	+	199,129	0,0488804	711.2090518
-211,897	211,897	+	211,897	0,04359	711.2100771
-203,043	203,043	+	203,043	0,0469572	712.1927049
-209,501	209,501	+	209,501	0,0442951	713.2251691
-329,456	329,456	+	329,456	0,00838462	713.5146338
-243,726	243,726	+	243,726	0,0315242	714.2087723
-237,821	237,821	+	237,821	0,0332685	714.4356689
-30,282	30,282	+	30,282	0,0143168	714.5183174
-291,615	291,615	+	291,615	0,0184182	714.5183829
-229,788	229,788	+	229,788	0,0357111	717.3294067

-201,401	201,401	+	201,401	0,0477773	721.1415565
-198,848	198,848	+	198,848	0,0488197	721.3849806
-27,206	27,206	+	27,206	0,0257042	721.3972778
-219,098	219,098	+	219,098	0,0404297	723.1725115
-270,796	270,796	+	270,796	0,025843	723.4121774
-264,679	264,679	+	264,679	0,0274585	725.1882532
-273,392	273,392	+	273,392	0,0253574	725.1887475
-262,729	262,729	+	262,729	0,0277633	725.5146533
-197,365	197,365	+	197,365	0,0499065	726.193732
-308,444	308,444	+	308,444	0,0126947	726.5183283
-313,123	313,123	+	313,123	0,0113763	726.5184153
-260,629	260,629	+	260,629	0,0281124	727.2041844
-230,066	230,066	+	230,066	0,0356593	728.1884157
-23,239	23,239	+	23,239	0,0350993	728.2068738
-215,052	215,052	+	215,052	0,0416642	729.2201637
-26,611	26,611	+	26,611	0,0268931	730.4312027
-218,352	218,352	+	218,352	0,0408778	731.2352899
-427,067	427,067	+	427,067	0,000597701	735.4122263
-200,867	200,867	+	200,867	0,0482455	739.1674989
-223,957	223,957	+	223,957	0,0382292	739.1683844
-197,737	197,737	+	197,737	0,0497052	740.1986521
-244,186	244,186	+	244,186	0,0312308	740.2014282
-333,815	333,815	+	333,815	0,00767785	740.5021388
-232,141	232,141	+	232,141	0,0352026	741.1833113
-257,169	257,169	+	257,169	0,0289813	741.5072753
-230,985	230,985	+	230,985	0,0356511	741.50867
-202,695	202,695	+	202,695	0,0470738	742.1877215
-223,324	223,324	+	223,324	0,0383131	742.4982245
-228,074	228,074	+	228,074	0,0362866	742.4990631
-275,432	275,432	+	275,432	0,0245075	743.1991666
-297,938	297,938	+	297,938	0,0160957	743.199325
-201,859	201,859	+	201,859	0,0476948	743.5033186
-231,345	231,345	+	231,345	0,0356928	744.203595
-254,836	254,836	+	254,836	0,0290352	745.2150122
-211,845	211,845	+	211,845	0,0435577	746.2166852
-255,244	255,244	+	255,244	0,0290789	746.2206002
-203,278	203,278	+	203,278	0,0470719	747.2311013
-292,248	292,248	+	292,248	0,0183041	749.4257866

-324,412	324,412	+	324,412	0,00912883	750.5316162
-263,345	263,345	+	263,345	0,0276886	753.5478516
-202,753	202,753	+	202,753	0,0471635	754.1792951
-200,547	200,547	+	200,547	0,0483579	755.1622168
-586,408	586,408	+	586,408	0	755.4645841
-216,376	216,376	+	216,376	0,0411429	756.1947519
-20,976	20,976	+	20,976	0,0442603	757.1780412
-217,895	217,895	+	217,895	0,0406986	757.1786638
-240,878	240,878	+	240,878	0,0326551	758.2115507
-205,179	205,179	+	205,179	0,0461201	758.7950847
-205,622	205,622	+	205,622	0,0461601	758.8052552
-198,699	198,699	+	198,699	0,0489479	758.8259541
-227,402	227,402	+	227,402	0,0366163	759.1939855
-209,044	209,044	+	209,044	0,0445688	760.1969608
-206,863	206,863	+	206,863	0,0455626	760.2003979
-199,444	199,444	+	199,444	0,0487199	761.2097631
-255,943	255,943	+	255,943	0,0289509	761.2099789
-245,852	245,852	+	245,852	0,0308033	762.2129355
-464,811	464,811	+	464,811	0,00030303	763.4450684
-353,528	353,528	+	353,528	0,00465185	765.4123286
-331,376	331,376	+	331,376	0,00792157	765.4841952
-22,021	22,021	+	22,021	0,0398923	767.5208392
-229,253	229,253	+	229,253	0,0361815	767.5208392
-201,796	201,796	+	201,796	0,0476673	769.4853152
-207,161	207,161	+	207,161	0,045607	770.1736834
-210,153	210,153	+	210,153	0,0439862	772.1894083
-224,231	224,231	+	224,231	0,0382453	772.1897771
-248,572	248,572	+	248,572	0,0300747	774.2052219
-23,889	23,889	+	23,889	0,0333383	775.1890692
-247,615	247,615	+	247,615	0,0302894	775.189333
-223,325	223,325	+	223,325	0,0383672	775.2073179
-218,047	218,047	+	218,047	0,0407802	775.2110113
-318,619	318,619	+	318,619	0,0102428	775.5513417
-205,184	205,184	+	205,184	0,0461646	776.1933152
-281,605	281,605	+	281,605	0,0222348	776.2216115
-224,274	224,274	+	224,274	0,0382139	777.2047931
-282,922	282,922	+	282,922	0,0219504	777.2235349
-464,691	464,691	+	464,691	0,000298507	777.4587402

-247,725	247,725	+	247,725	0,0302857	778.2058429
-231,173	231,173	+	231,173	0,0356938	778.2117875
-50,574	50,574	+	505,741	7.14E+00	778.5265695
-199,729	199,729	+	199,729	0,0487379	779.2211885
-409,946	409,946	+	409,946	0,00121739	779.5289484
-431,334	431,334	+	431,334	0,000611765	779.531095
-223,943	223,943	+	223,943	0,0381803	780.2231384
-253,104	253,104	+	253,104	0,0289091	780.4679972
-403,948	403,948	+	403,948	0,00141667	780.5327352
-453,918	453,918	+	453,918	0,000285714	781.488641
-499,803	499,803	+	499,803	7.02E+00	781.4912332
-4,326	4,326	+	4,326	0,00052381	782.4934627
-591,658	591,658	+	591,658	0	783.4961135
-206,755	206,755	+	206,755	0,0455298	784.4767522
-20,147	20,147	+	20,147	0,0478882	786.1697127
-205,292	205,292	+	205,292	0,0462542	786.2106991
-207,697	207,697	+	207,697	0,0453925	788.184654
-250,511	250,511	+	250,511	0,0294898	790.2006846
-201,863	201,863	+	201,863	0,0477347	791.1847103
-233,766	233,766	+	233,766	0,0346576	791.2035663
-20,233	20,233	+	20,233	0,0473824	791.4266186
-383,867	383,867	+	383,867	0,00230631	791.4720113
-271,825	271,825	+	271,825	0,0255889	792.2162584
-198,422	198,422	+	198,422	0,0490926	792.2188634
-21,059	21,059	+	21,059	0,0439258	793.2009041
-231,442	231,442	+	231,442	0,0356334	793.2172424
-25,604	25,604	+	25,604	0,0291667	793.2203743
-239,608	239,608	+	239,608	0,0328315	793.4811704
-22,823	22,823	+	22,823	0,0362879	793.5165922
-205,515	205,515	+	205,515	0,0461512	794.2332782
-211,932	211,932	+	211,932	0,0435943	797.1911151
-557,894	557,894	+	557,894	0	799.4918172
-20,316	20,316	+	20,316	0,0468898	800.19306
-244,714	244,714	+	244,714	0,0312115	803.1966941
-209,734	209,734	+	209,734	0,0442326	805.1848425
-274,254	274,254	+	274,254	0,0249853	805.2136975
-22,623	22,623	+	22,623	0,0373294	806.1957193
-429,104	429,104	+	429,104	0,000604651	806.5575435

-223,675	223,675	+	223,675	0,038302	807.201027
-646,214	646,214	+	646,214	0	807.5057632
-425,495	425,495	+	425,495	0,000636364	807.5617855
-27,199	27,199	+	27,199	0,0256421	808.2115086
-595,086	595,086	+	595,086	0	808.5084258
-631,581	631,581	+	631,582	0	808.5112476
-218,146	218,146	+	218,146	0,0408188	809.2141678
-247,077	247,077	+	247,077	0,0305236	809.5110269
-272,732	272,732	+	272,732	0,0256774	809.5111034
-235,418	235,418	+	235,418	0,0338722	810.1652219
-293,684	293,684	+	293,684	0,0178047	810.5144951
-227,467	227,467	+	227,467	0,0365991	812.1830572
-200,847	200,847	+	200,847	0,0482176	814.2152636
-253,803	253,803	+	253,803	0,0289659	815.1999376
-206,884	206,884	+	206,884	0,0455861	818.1827191
-223,998	223,998	+	223,998	0,0382931	819.5019783
-317,857	317,857	+	317,857	0,0102825	819.5321045
-21,728	21,728	+	21,728	0,041027	820.1949575
-227,173	227,173	+	227,173	0,036741	821.206842
-220,562	220,562	+	220,562	0,0397724	821.4613647
-221,622	221,622	+	221,622	0,0390824	822.1910589
-207,455	207,455	+	207,455	0,0455011	822.2076041
-229,177	229,177	+	229,177	0,0361685	822.2114569
-306,058	306,058	+	306,058	0,013299	823.527422
-320,961	320,961	+	320,961	0,00969767	825.5065398
-241,556	241,556	+	241,556	0,0327466	826.1557582
-262,285	262,285	+	262,285	0,0279648	826.1804819
-236,845	236,845	+	236,845	0,0333521	826.5747681
-220,962	220,962	+	220,962	0,0395102	827.1637833
-251,294	251,294	+	251,294	0,0294411	827.5221959
-226,892	226,892	+	226,892	0,0368636	828.1744067
-217,135	217,135	+	217,135	0,0410179	830.5952675
-26,647	26,647	+	26,647	0,0267595	831.5025059
-208,153	208,153	+	208,153	0,0450714	833.2101287
-473,206	473,206	+	473,206	0,0001875	833.5202337
-511,158	511,158	+	511,158	7.69E+00	833.5202337
-201,413	201,413	+	201,413	0,0478685	834.2418143
-204,194	204,194	+	204,194	0,0468083	834.4285889

**-38,596**	**38,596**	+	**38,596**	**0,00225926**	**834.525377**
**-201,959**	**201,959**	+	**201,959**	**0,0476338**	**835.1864664**
**-720,122**	**720,122**	+	**720,119**	**0**	**835.5370483**
**-658,813**	**658,813**	+	**658,813**	**0**	**835.5394897**
**-69,529**	**69,529**	+	**695,288**	**0**	**836.5391192**
**-72,277**	**72,277**	+	**722,767**	**0**	**836.5412607**
**-224,791**	**224,791**	+	**224,791**	**0,0380933**	**837.2021751**
**-537,956**	**537,956**	+	**537,956**	**0**	**837.5414653**
**-726,288**	**726,288**	+	**726,284**	**0**	**837.5416962**
**-230,026**	**230,026**	+	**230,026**	**0,0355786**	**838.2043505**
**-589,795**	**589,795**	+	**589,795**	**0**	**838.545559**
**-215,455**	**215,455**	+	**215,455**	**0,0415262**	**842.172605**
**-215,872**	**215,872**	+	**215,872**	**0,0412682**	**842.5683598**
**-219,274**	**219,274**	+	**219,274**	**0,0404314**	**842.5710677**
**-210,629**	**210,629**	+	**210,629**	**0,0439165**	**845.194415**
**-218,315**	**218,315**	+	**218,315**	**0,0408231**	**846.5856323**
**-300,662**	**300,662**	+	**300,662**	**0,015122**	**847.2361227**
**-236,502**	**236,502**	+	**236,502**	**0,0334011**	**847.5367752**
**-360,262**	**360,262**	+	**360,262**	**0,00387692**	**849.5499878**
**-402,435**	**402,435**	+	**402,435**	**0,00146939**	**849.5515363**
**-212,874**	**212,874**	+	**212,874**	**0,0432174**	**850.2358256**
**-557,939**	**557,939**	+	**557,939**	**0**	**850.5582004**
**-608,058**	**608,058**	+	**608,058**	**0**	**850.5629524**
**-207,992**	**207,992**	+	**207,992**	**0,0452129**	**851.5567597**
**-368,776**	**368,776**	+	**368,776**	**0,00327869**	**851.5655126**
**-203,217**	**203,217**	+	**203,217**	**0,0469887**	**852.2518115**
**-462,528**	**462,528**	+	**462,528**	**0,000294118**	**853.5370802**
**-278,007**	**278,007**	+	**278,007**	**0,0237829**	**854.5395211**
**-210,323**	**210,323**	+	**210,323**	**0,0440046**	**855.1670456**
**-287,112**	**287,112**	+	**287,112**	**0,0200172**	**855.5536434**
**-258,247**	**258,247**	+	**258,247**	**0,0289399**	**856.5373151**
**-209,399**	**209,399**	+	**209,399**	**0,0443356**	**857.1646772**
**-437,133**	**437,133**	+	**437,133**	**0,000395062**	**857.5208326**
**-452,047**	**452,047**	+	**452,047**	**0,000333333**	**857.5209324**
**-238,227**	**238,227**	+	**238,227**	**0,0332291**	**857.5430908**
**-285,276**	**285,276**	+	**285,276**	**0,0208305**	**857.5554469**
**-438,765**	**438,765**	+	**438,765**	**0,000415584**	**858.5243291**
**-292,047**	**292,047**	+	**292,047**	**0,018367**	**858.550368**

-30,439	30,439	+	30,439	0,0137868	858.5514328
-438,184	438,184	+	438,184	0,000410256	858.5620728
-198,912	198,912	+	198,912	0,0487977	859.5365875
-273,441	273,441	+	273,441	0,0255127	859.5557753
-349,545	349,545	+	349,545	0,00518841	859.5691528
-19,948	19,948	+	19,948	0,0487637	860.5399524
-200,776	200,776	+	200,776	0,0482369	860.5401385
-224,959	224,959	+	224,959	0,0380409	861.4537354
-228,198	228,198	+	228,198	0,0362813	861.5518176
-292,513	292,513	+	292,513	0,0182222	861.5521234
-236,505	236,505	+	236,505	0,0334596	862.4985962
-315,372	315,372	+	315,372	0,0108571	862.5556134
-213,459	213,459	+	213,459	0,0427767	863.2321827
-223,176	223,176	+	223,176	0,0382865	863.5082063
-694,126	694,126	+	694,125	0	863.5670722
-761,041	761,041	+	761,032	0	863.568287
-664,128	664,128	+	664,128	0	864.5708818
-729,197	729,197	+	729,193	0	864.5712833
-227,497	227,497	+	227,497	0,0366182	865.2468145
-201,443	201,443	+	201,443	0,0478843	865.2511018
-692,026	692,026	+	692,025	0	865.5740234
-221,441	221,441	+	221,441	0,0390971	866.2308389
-294,213	294,213	+	294,213	0,0176526	866.2523772
-382,358	382,358	+	382,358	0,00235714	866.5761108
-370,563	370,563	+	370,563	0,00319328	867.2639441
-221,652	221,652	+	221,652	0,0391197	868.2500293
-403,019	403,019	+	403,019	0,00148454	871.5598419
-405,801	405,801	+	405,801	0,00140426	871.5598419
-231,124	231,124	+	231,124	0,0356878	872.564685
-199,841	199,841	+	199,841	0,0486615	873.2239478
-211,836	211,836	+	211,836	0,0435154	879.2264754
-380,865	380,865	+	380,865	0,00237168	879.553827
-228,774	228,774	+	228,774	0,0359938	881.2423935
-267,951	267,951	+	267,951	0,0265065	882.2468344
-265,584	265,584	+	265,584	0,026882	883.2606014
-215,384	215,384	+	215,384	0,0415791	884.2634511
-204,094	204,094	+	204,094	0,0468025	884.5675514
-216,239	216,239	+	216,239	0,0412323	884.570246

-258,818	258,818	+	258,818	0,028895	885.446228
-473,108	473,108	+	473,108	0,000184615	888.5154042
-244,606	244,606	+	244,606	0,0312459	889.5830982
-200,739	200,739	+	200,739	0,0482446	890.2515599
-397,772	397,772	+	397,772	0,00166337	890.5352427
-212,856	212,856	+	212,856	0,0431325	890.5868026
-216,415	216,415	+	216,415	0,0411696	890.5877605
-508,215	508,215	+	508,215	7.41E+00	891.4930712
-257,941	257,941	+	257,941	0,0290109	891.5912703
-218,641	218,641	+	218,641	0,0407124	892.2237831
-24,445	24,445	+	24,445	0,031249	893.4943831
-387,241	387,241	+	387,241	0,00216822	893.5067512
-439,505	439,505	+	439,505	0,000426667	893.5073853
-218,038	218,038	+	218,038	0,0407325	894.2372077
-219,567	219,567	+	219,567	0,040369	894.2409443
-241,487	241,487	+	241,487	0,0325536	894.4976364
-34,009	34,009	+	34,009	0,00658333	894.505188
-519,103	519,103	+	519,103	8.33E+00	894.512249
-200,186	200,186	+	200,186	0,0486033	895.2219449
-202,746	202,746	+	202,746	0,047076	896.2257207
-30,385	30,385	+	30,385	0,0139798	896.2554286
-215,811	215,811	+	215,811	0,0412816	897.2370496
-243,657	243,657	+	243,657	0,0315091	897.2392559
-235,588	235,588	+	235,588	0,033875	897.5747354
-255,828	255,828	+	255,828	0,0290722	898.241848
-248,413	248,413	+	248,413	0,0301754	900.2555702
-485,793	485,793	+	485,793	0,00020339	903.5293857
-51,671	51,671	+	51,671	8.16E+00	904.5111694
-573,342	573,342	+	573,342	0	907.5237754
-21,059	21,059	+	21,059	0,043875	908.2201009
-6,716	6,716	+	6,716	0	908.5263208
-257,936	257,936	+	257,936	0,0289322	909.4533955
-230,266	230,266	+	230,266	0,0356698	909.5346292
-22,222	22,222	+	22,222	0,0388033	910.2334342
-199,516	199,516	+	199,516	0,0487606	911.2160851
-217,511	217,511	+	217,511	0,040933	911.2368038
-264,533	264,533	+	264,533	0,0273761	911.2411245
-280,357	280,357	+	280,357	0,022757	912.2475799

-308,794	308,794	+	308,794	0,0125926	912.2505869
-205,288	205,288	+	205,288	0,0461649	912.611659
-208,851	208,851	+	208,851	0,0446982	913.232275
-199,558	199,558	+	199,558	0,0487652	915.2504423
-256,055	256,055	+	256,055	0,0292219	915.5031683
-633,606	633,606	+	633,606	0	917.5087039
-869,846	869,846	+	869,913	0	917.5093415
-676,577	676,577	+	676,576	0	918.5132153
-275,651	275,651	+	275,651	0,0244494	919.5160776
-731,085	731,085	+	731,081	0	919.5234222
-261,777	261,777	+	261,777	0,0279652	920.2205102
-605,079	605,079	+	605,079	0	920.5261055
-550,139	550,139	+	550,139	0	920.527626
-399,056	399,056	+	399,056	0,00164	921.5287476
-272,137	272,137	+	272,137	0,0257102	921.5371767
-333,082	333,082	+	333,082	0,00776	922.5355225
-276,956	276,956	+	276,956	0,0243065	923.5245693
-1,977	1,977	+	1,977	0,0497186	926.2271862
-209,526	209,526	+	209,526	0,0444009	926.2299595
-222,572	222,572	+	222,572	0,0385167	927.2329539
-249,038	249,038	+	249,038	0,0299823	928.2441223
-281,362	281,362	+	281,362	0,0222742	929.5411987
-306,471	306,471	+	306,471	0,0133542	930.2626881
-29,843	29,843	+	29,843	0,0159423	931.5586094
-271,645	271,645	+	271,645	0,0255225	931.5600136
-339,872	339,872	+	339,872	0,00656552	932.5440875
-239,873	239,873	+	239,873	0,0327068	932.5555842
-230,242	230,242	+	230,242	0,035645	935.2326321
-438,077	438,077	+	438,077	0,000405063	935.5157137
-290,273	290,273	+	290,273	0,0187022	937.4831543
-229,188	229,188	+	229,188	0,0362125	937.6135745
-202,706	202,706	+	202,705	0,0470972	938.615515
-216,656	216,656	+	216,656	0,0411182	938.6267514
-212,862	212,862	+	212,862	0,0431749	939.6204975
-211,051	211,051	+	211,051	0,0438782	939.6327515
-256,645	256,645	+	256,645	0,0290554	940.4943926
-223,038	223,038	+	223,038	0,0383081	941.6012202
-203,906	203,906	+	203,906	0,04692	941.6354705

-203,165	203,165	+	203,165	0,0469377	942.2227393
-379,735	379,735	+	379,735	0,00249123	942.5643999
-213,093	213,093	+	213,093	0,0430751	942.605658
-282,626	282,626	+	282,626	0,0219508	943.5335249
-203,741	203,741	+	203,741	0,0467093	944.2368767
-254,252	254,252	+	254,252	0,0290594	944.2413735
-552,517	552,517	+	552,517	0	944.5796283
-405,152	405,152	+	405,152	0,00138947	944.5800682
-62,871	62,871	+	62,871	0	945.5384424
-562,569	562,569	+	562,569	0	945.5388343
-393,155	393,155	+	393,155	0,00192157	945.5769971
-324,673	324,673	+	324,673	0,0091358	945.5831238
-294,345	294,345	+	294,345	0,0176604	946.5358959
-374,641	374,641	+	374,641	0,0027931	946.5434583
-492,703	492,703	+	492,703	6.90E+00	947.5901578
-439,236	439,236	+	439,236	0,000421053	947.5911648
-317,963	317,963	+	317,963	0,0102727	948.5938049
-20,544	20,544	+	20,544	0,0461807	949.2136185
-291,891	291,891	+	291,891	0,0183562	949.5586279
-235,021	235,021	+	235,021	0,0339863	949.6055624
-208,568	208,568	+	208,568	0,0448117	950.2141896
-203,268	203,268	+	203,268	0,04704	951.621933
-214,682	214,682	+	214,682	0,0418133	951.6220498
-207,586	207,586	+	207,586	0,0453878	953.6380168
-203,875	203,875	+	203,875	0,0468921	955.2215512
-225,644	225,644	+	225,644	0,0375376	955.2611663
-393,029	393,029	+	393,029	0,00190291	955.4995728
-231,561	231,561	+	231,561	0,0356066	957.2354159
-308,156	308,156	+	308,156	0,0127539	957.574483
-453,658	453,658	+	453,658	0,000338028	958.5585867
-355,784	355,784	+	355,784	0,00442424	958.5599535
-32,241	32,241	+	32,241	0,00955689	959.5549462
-216,555	216,555	+	216,555	0,0411624	959.5899685
-222,124	222,124	+	222,124	0,0388177	959.5903252
-255,065	255,065	+	255,065	0,0290937	960.5575551
-423,439	423,439	+	423,439	0,000808989	961.5699248
-419,223	419,223	+	419,223	0,000977778	961.5700657
-305,052	305,052	+	305,052	0,013551	962.5733726

-321,861	321,861	+	321,861	0,00955294	963.5778352
-370,153	370,153	+	370,153	0,0032	963.5822359
-206,611	206,611	+	206,611	0,0455335	964.2247747
-221,544	221,544	+	221,544	0,0390411	964.5483394
-462,329	462,329	+	462,329	0,000289855	964.5823975
-211,892	211,892	+	211,892	0,0435524	965.3842216
-267,988	267,988	+	267,988	0,0265277	966.5619295
-241,388	241,388	+	241,388	0,0325282	967.6158005
-329,644	329,644	+	329,644	0,0084129	968.5819431
-256,247	256,247	+	256,247	0,0292178	969.2626885
-255,948	255,948	+	255,948	0,0290259	969.5353099
-321,998	321,998	+	321,998	0,00966667	969.547102
-216,774	216,774	+	216,774	0,0410636	970.2717836
-388,362	388,362	+	388,362	0,00217143	970.5947603
-473,389	473,389	+	473,389	0,000190476	971.5545653
-679,265	679,265	+	679,265	0	971.5594482
-323,944	323,944	+	323,944	0,00919512	971.5994764
-436,494	436,494	+	436,494	0,000385542	972.5587868
-566,741	566,741	+	566,741	0	972.5605565
-506,888	506,888	+	506,888	7.27E+00	973.5693081
-51,613	51,613	+	51,613	8.00E-05	973.5693775
-353,337	353,337	+	353,337	0,00464706	974.5724093
-5,245	5,245	+	5,245	0	974.5731103
-362,972	362,972	+	362,972	0,00371654	975.5810419
-240,373	240,373	+	240,373	0,0326616	975.5840511
-225,857	225,857	+	225,857	0,037549	976.5885458
-200,011	200,011	+	200,011	0,0486523	976.5892564
-364,435	364,435	+	364,435	0,00358065	977.5908813
-341,322	341,322	+	341,322	0,00643972	977.5946322
-384,611	384,611	+	384,611	0,00229091	978.5971662
-332,138	332,138	+	332,138	0,00792053	979.5982092
-345,063	345,063	+	345,063	0,00581295	979.6021014
-235,302	235,302	+	235,302	0,0337938	981.5126666
-270,248	270,248	+	270,248	0,0259125	981.5722991
-294,023	294,023	+	294,023	0,017757	983.5217896
-282,095	282,095	+	282,095	0,0220813	983.5906068
-352,344	352,344	+	352,344	0,00481752	984.5715332
-209,516	209,516	+	209,516	0,0443273	985.283557

-354,444	354,444	+	354,444	0,00450746	985.572104
-569,033	569,033	+	569,033	0	987.5854103
-287,366	287,366	+	287,366	0,0199483	988.2812186
-436,766	436,766	+	436,766	0,000390244	988.5886262
-385,334	385,334	+	385,334	0,00231193	988.5894864
-364,031	364,031	+	364,031	0,003584	989.5647034
-327,483	327,483	+	327,483	0,00878481	989.6006532
-278,594	278,594	+	278,594	0,0235	989.6012798
-317,389	317,389	+	317,389	0,0103464	990.5658206
-240,028	240,028	+	240,028	0,0326629	990.5707936
-25,279	25,279	+	25,279	0,0290095	990.5982835
-232,702	232,702	+	232,702	0,0349884	990.6045576
-257,059	257,059	+	257,059	0,0289681	991.5797543
-274,439	274,439	+	274,439	0,0249446	991.58013
-446,631	446,631	+	446,631	0,000328767	993.5426905
-242,026	242,026	+	242,026	0,0324103	994.5835928
-283,257	283,257	+	283,257	0,0218257	995.5530108
-31,441	31,441	+	31,441	0,0109405	997.5694631
-388,476	388,476	+	388,476	0,00219231	997.5713033
-339,101	339,101	+	339,101	0,00668493	998.5750814
-291,186	291,186	+	291,186	0,0185701	998.5791632
-444,053	444,053	+	444,053	0,000324324	999.5852016
-321,898	321,898	+	321,898	0,00960947	999.5872787

Supplementary Table 3: Posthoc test BUVEC for control cells in positive-ion mode

Control	Infected	C: ANOVA Significant	N: -Log ANOVA p value	N: ANOVA q-value	MALDI m/z
2.43784	-2.43784	+	2.43784	0.0414155	361.2737885
2.43663	-2.43663	+	2.43663	0.0414552	363.2889906
3.9936	-3.9936	+	3.9936	0.00435023	385.2737679
2.88406	-2.88406	+	2.88406	0.0227211	387.2892692
2.53601	-2.53601	+	2.53601	0.0362366	566.3198033
2.53633	-2.53633	+	2.53633	0.0364164	566.3206856
2.32571	-2.32571	+	2.32571	0.0478227	567.3240585
2.31481	-2.31481	+	2.31481	0.048509	584.3092298
2.31791	-2.31791	+	2.31791	0.048419	584.3108605

2.43589	-2.43589	+	2.43589	0.0414246	611.5388808
2.33311	-2.33311	+	2.33311	0.0476099	644.5009491
3.05381	-3.05381	+	3.05381	0.0188377	678.3393849
3.06162	-3.06162	+	3.06162	0.0187263	678.3403438
2.34412	-2.34412	+	2.34412	0.0469435	679.3436198
2.87686	-2.87686	+	2.87686	0.0229436	704.3557465
2.88616	-2.88616	+	2.88616	0.0226727	705.3590423
3.4767	-3.4767	+	3.4767	0.00998684	706.3714581
3.34369	-3.34369	+	3.34369	0.0121557	707.374831
2.74007	-2.74007	+	2.74007	0.0278742	787.5937226
3.82509	-3.82509	+	3.82509	0.0054918	840.3689965
3.88361	-3.88361	+	3.88361	0.00497436	840.3708738
3.90917	-3.90917	+	3.90917	0.00490265	842.385889
2.30691	-2.30691	+	2.30691	0.0486516	858.6094119
2.30675	-2.30675	+	2.30675	0.0485862	858.6113279
2.7153	-2.7153	+	2.7153	0.0285773	859.6141394
2.47465	-2.47465	+	2.47465	0.0391815	865.6051994
2.77698	-2.77698	+	2.77698	0.026453	866.6086251
3.0241	-3.0241	+	3.0241	0.0195773	902.5885121
3.06142	-3.06142	+	3.06142	0.0186772	902.5904
3.74299	-3.74299	+	3.74299	0.00628794	903.592499
3.615	-3.615	+	3.615	0.00765714	907.5337917
3.84062	-3.84062	+	3.84062	0.00528395	922.6713775
2.53263	-2.53263	+	2.53263	0.0363757	928.6046923
2.74544	-2.74544	+	2.74544	0.0277731	929.6082157
2.54486	-2.54486	+	2.54486	0.0358507	930.6197399
2.54486	-2.54486	+	2.54486	0.035784	930.6206399
2.7347	-2.7347	+	2.7347	0.0280837	931.6238551
2.43252	-2.43252	+	2.43252	0.0417093	931.6880925
3.42851	-3.42851	+	3.42851	0.0108462	932.6252655
2.74772	-2.74772	+	2.74772	0.0277221	934.6424793
2.53097	-2.53097	+	2.53097	0.0363897	977.6672613
2.41598	-2.41598	+	2.41598	0.0424775	1040.621805
2.3006	-2.3006	+	2.3006	0.0486454	1047.578258
2.30148	-2.30148	+	2.30148	0.04875	1047.580625
2.32973	-2.32973	+	2.32973	0.0476837	1064.622211
2.72933	-2.72933	+	2.72933	0.0280913	1066.638117
2.75043	-2.75043	+	2.75043	0.0276371	1067.641909

2.32913	-2.32913	+	2.32914	0.0476645	1068.654043

Supplementary Table 4: Posthoc test BUVEC for control cells in negative-ion mode

Control	Infected	C: ANOVA Significant	N: -Log ANOVA p value	N: ANOVA q-value	MALDI m/z
257,348	-257,348	+	257,348	0,0289973	251.0488104
252,182	-252,182	+	252,182	0,02912	251.0613547
258,315	-258,315	+	258,315	0,0289644	252.0566252
238,609	-238,609	+	238,609	0,033145	252.0691094
241,534	-241,534	+	241,534	0,0326902	253.0519294
245,934	-245,934	+	245,934	0,0308319	253.0645187
254,214	-254,214	+	254,214	0,0290272	254.0723111
198,533	-198,533	+	198,533	0,0490104	255.0439031
207,675	-207,675	+	207,675	0,0453732	255.0756868
220,976	-220,976	+	220,976	0,0395531	256.087977
216,862	-216,862	+	216,862	0,0410038	257.0204124
233,292	-233,292	+	233,292	0,0347966	257.0568107
201,412	-201,412	+	201,412	0,0478209	257.0716642
20,885	-20,885	+	20,885	0,0446479	257.0832284
215,088	-215,088	+	215,088	0,0416761	260.0254293
26,657	-26,657	+	26,657	0,0268153	261.0458061
253,064	-253,064	+	253,064	0,0288592	263.0363432
200,611	-200,611	+	200,611	0,0483228	263.0460111
215,919	-215,919	+	215,919	0,0412798	263.0575892
276,623	-276,623	+	276,623	0,024458	263.0614868
211,532	-211,532	+	211,532	0,0435477	264.0316368
270,855	-270,855	+	270,855	0,0259041	264.0647657
235,316	-235,316	+	235,316	0,0338451	264.0779681
250,387	-250,387	+	250,387	0,0294292	264.0816884
261,293	-261,293	+	261,293	0,0283343	265.0521116
21,865	-21,865	+	21,865	0,0407662	265.0607684
238,565	-238,565	+	238,565	0,0331282	266.0360664
214,915	-214,915	+	214,915	0,0417559	266.0473478
203,084	-203,084	+	203,084	0,046948	266.0572899
256,073	-256,073	+	256,073	0,0292775	266.0724903
238,098	-238,098	+	238,098	0,0332559	266.0805427

213,505	-213,505	+	213,505	0,0428175	266.093601
241,122	-241,122	+	241,122	0,0326512	267.0416115
243,229	-243,229	+	243,229	0,0315936	267.067794
233,317	-233,317	+	233,317	0,0348283	267.0758457
231,058	-231,058	+	231,057	0,0357208	267.092863
235,336	-235,336	+	235,336	0,0338828	268.036667
240,892	-240,892	+	240,892	0,0327104	268.0518283
255,067	-255,067	+	255,067	0,0291574	268.0729015
222,135	-222,135	+	222,135	0,0388603	268.0882162
205,412	-205,412	+	205,412	0,0461656	269.0466262
230,759	-230,759	+	230,759	0,0355328	269.0721919
214,888	-214,888	+	214,888	0,0417094	269.0834772
270,348	-270,348	+	270,348	0,0259054	270.0523039
21,319	-21,319	+	21,319	0,0430448	270.0675681
210,249	-210,249	+	210,249	0,0440138	273.0397617
213,464	-213,464	+	213,464	0,042819	273.0397863
222,723	-222,723	+	222,723	0,0383922	275.0310806
246,641	-246,641	+	246,641	0,0305254	276.0442001
269,525	-269,525	+	269,525	0,0261333	277.0646046
238,777	-238,777	+	238,777	0,0332495	278.0478019
26,662	-26,662	+	26,662	0,0268754	278.0723256
197,928	-197,928	+	197,928	0,0495165	278.0934436
234,582	-234,582	+	234,582	0,034198	279.0432806
255,494	-255,494	+	255,494	0,028949	279.0676451
24,799	-24,799	+	24,799	0,0302522	279.0762051
245,119	-245,119	+	245,119	0,0311203	279.0926896
232,843	-232,843	+	232,843	0,034985	280.051666
223,608	-223,608	+	223,608	0,0383101	280.063483
246,902	-246,902	+	246,902	0,0304255	280.0880006
22,166	-22,166	+	22,166	0,039168	281.0680347
232,639	-232,639	+	232,639	0,0349254	281.0833277
20,472	-20,472	+	20,472	0,046457	282.0348941
243,892	-243,892	+	243,892	0,0313778	282.0520882
225,843	-225,843	+	225,843	0,0374556	282.0673654
31,763	-31,763	+	31,763	0,0103146	282.0874865
211,496	-211,496	+	211,496	0,0435671	282.1036955
251,264	-251,264	+	251,264	0,0294194	283.0474143
200,935	-200,935	+	200,935	0,0482022	284.0255602

289,435	-289,435	+	289,435	0,0189604	284.067704
202,031	-202,031	+	202,031	0,047577	284.0830107
252,486	-252,486	+	252,486	0,0291374	285.0459236
207,054	-207,054	+	207,054	0,0456254	287.057597
273,123	-273,123	+	273,123	0,0254245	287.0615567
253,396	-253,396	+	253,396	0,0288289	288.0650485
254,943	-254,943	+	254,943	0,0291212	288.0817868
267,158	-267,158	+	267,157	0,0266881	290.072475
242,648	-242,648	+	242,648	0,0319524	290.0807305
259,259	-259,259	+	259,259	0,0286537	291.0678896
209,026	-209,026	+	209,026	0,0445411	291.0889383
254,883	-254,883	+	254,883	0,0290882	291.0929064
203,411	-203,411	+	203,411	0,0470608	292.0521498
199,454	-199,454	+	199,454	0,0487553	292.0630453
249,167	-249,167	+	249,167	0,0300044	292.0882063
204,194	-204,194	+	204,194	0,0467596	292.0960514
204,718	-204,718	+	204,718	0,0464126	293.047182
229,163	-229,163	+	229,163	0,0361246	293.0568427
235,537	-235,537	+	235,537	0,0337855	293.0720999
237,902	-237,902	+	237,902	0,033278	293.0835056
228,279	-228,279	+	228,279	0,0362883	294.0675294
206,814	-206,814	+	206,814	0,0454924	294.0881609
215,733	-215,733	+	215,733	0,041325	294.1038969
210,208	-210,208	+	210,208	0,0440138	295.0989114
277,547	-277,547	+	277,547	0,0240309	296.0675897
211,108	-211,108	+	211,108	0,0438453	296.0829246
26,093	-26,093	+	26,093	0,0283305	298.0469455
199,118	-199,118	+	199,118	0,0488566	298.0834249
24,803	-24,803	+	24,803	0,0303755	299.0422596
270,565	-270,565	+	270,565	0,025932	300.0449223
261,433	-261,433	+	261,433	0,0282312	300.0568518
272,176	-272,176	+	272,176	0,0258014	301.064771
233,857	-233,857	+	233,857	0,0346282	302.0683247
271,206	-271,206	+	271,206	0,0258207	302.0724614
258,505	-258,505	+	258,505	0,0290028	303.0678173
235,644	-235,644	+	235,644	0,0338852	304.0635026
246,134	-246,134	+	246,134	0,0308101	305.0835781
225,706	-225,706	+	225,706	0,0374985	306.1038181

216,097	-216,097	+	216,097	0,0412695	307.0991141
210,984	-210,984	+	210,984	0,0437573	308.0677874
212,226	-212,226	+	212,226	0,0434641	308.0831233
211,119	-211,119	+	211,119	0,043892	309.0518177
241,508	-241,508	+	241,508	0,0326497	309.0631525
201,202	-201,202	+	201,202	0,0479881	309.0875027
26,919	-26,919	+	26,919	0,026299	310.0664722
214,257	-214,257	+	214,257	0,0420317	310.0833577
26,651	-26,651	+	26,651	0,0268063	311.0617048
260,865	-260,865	+	260,865	0,0281356	312.0569707
335,142	-335,142	+	335,142	0,00743243	312.0644427
371,723	-371,723	+	371,723	0,00311864	312.0821683
206,111	-206,111	+	206,111	0,0458818	313.0649801
214,331	-214,331	+	214,331	0,0419951	313.0856389
269,676	-269,676	+	269,676	0,0261672	314.0726155
21,247	-21,247	+	21,247	0,0433421	314.0809299
262,833	-262,833	+	262,833	0,0277745	315.0680452
290,363	-290,363	+	290,363	0,0187143	315.0761052
265,667	-265,667	+	265,667	0,027	315.0930467
282,887	-282,887	+	282,887	0,0218601	316.0517537
228,878	-228,878	+	228,878	0,0360185	316.0630984
240,348	-240,348	+	240,348	0,0325606	317.0835434
199,706	-199,706	+	199,706	0,04871	319.0627228
236,355	-236,355	+	236,355	0,0334406	323.0569814
243,314	-243,314	+	243,314	0,031624	323.0786715
220,204	-220,204	+	220,204	0,0398441	324.0442514
204,733	-204,733	+	204,733	0,0464806	325.0468193
264,785	-264,785	+	264,785	0,0274568	325.0579585
263,484	-263,484	+	263,484	0,0278308	325.0648355
262,081	-262,081	+	262,081	0,028	326.0725796
251,448	-251,448	+	251,448	0,0293148	326.08517
248,781	-248,781	+	248,781	0,0300088	327.0678485
211,573	-211,573	+	211,573	0,0435519	327.073512
263,364	-263,364	+	263,364	0,0277477	327.0930336
257,332	-257,332	+	257,332	0,0289519	328.0759819
207,963	-207,963	+	207,963	0,0451893	328.0964017
210,797	-210,797	+	210,797	0,0438419	329.0531608
256,705	-256,705	+	256,705	0,0290794	329.0835011

203,235	-203,235	+	203,235	0,0470246	330.087679
233,289	-233,289	+	233,289	0,0347383	331.099136
199,968	-199,968	+	199,968	0,04864	332.1030158
199,071	-199,071	+	199,071	0,0488168	333.0462953
207,176	-207,176	+	207,176	0,045681	335.0669963
250,653	-250,653	+	250,653	0,0294966	336.0571901
220,525	-220,525	+	220,525	0,0397348	337.0469664
275,241	-275,241	+	275,241	0,0244	337.0775302
22,103	-22,103	+	22,103	0,0395252	339.0561768
260,984	-260,984	+	260,984	0,0283771	339.0676879
340,535	-340,535	+	340,535	0,00648951	339.0737308
270,908	-270,908	+	270,908	0,0259656	339.092954
279,641	-279,641	+	279,641	0,0231146	340.0762571
205,626	-205,626	+	205,626	0,0462094	340.0879915
204,788	-204,788	+	204,788	0,0464874	340.0953213
246,913	-246,913	+	246,913	0,0304819	341.0530076
25,249	-25,249	+	25,249	0,0292067	341.0834301
21,762	-21,762	+	21,762	0,0409032	341.0914702
230,817	-230,817	+	230,817	0,0357101	342.0787511
206,115	-206,115	+	206,115	0,0459312	342.0873756
253,196	-253,196	+	253,196	0,0289423	342.1038697
237,973	-237,973	+	237,973	0,0333116	343.0990405
199,049	-199,049	+	199,049	0,0487931	343.1071639
200,734	-200,734	+	200,734	0,0482089	344.094394
306,177	-306,177	+	306,177	0,0133472	345.0742797
227,336	-227,336	+	227,336	0,0366395	345.0992437
201,797	-201,797	+	201,797	0,0477151	345.1147122
218,317	-218,317	+	218,317	0,0408714	346.0833785
208,258	-208,258	+	208,258	0,0450783	347.1148163
219,389	-219,389	+	219,389	0,0404053	348.0986937
229,061	-229,061	+	229,061	0,0360807	350.1144305
237,177	-237,177	+	237,177	0,033305	351.0932498
247,039	-247,039	+	247,039	0,0304188	352.0766595
268,246	-268,246	+	268,246	0,0264575	353.0530504
250,866	-250,866	+	250,866	0,0295251	353.0670415
237,471	-237,471	+	237,471	0,0332286	353.1255605
238,839	-238,839	+	238,839	0,0332593	354.0556624
268,765	-268,765	+	268,765	0,0262895	354.1040358

215,149	-215,149	+	215,149	0,0417469	355.0577184
249,309	-249,309	+	249,309	0,0299111	355.099249
232,035	-232,035	+	232,035	0,0351856	356.0835046
217,037	-217,037	+	217,037	0,0410013	356.0945322
206,544	-206,544	+	206,544	0,0455707	356.1197218
284,982	-284,982	+	284,982	0,0209789	357.0868134
214,416	-214,416	+	214,416	0,0420465	357.1148672
205,338	-205,338	+	205,338	0,0462481	359.0788846
203,819	-203,819	+	203,819	0,0468323	359.1149548
210,367	-210,367	+	210,367	0,0440185	360.0570325
245,429	-245,429	+	245,429	0,0310981	361.0945123
230,767	-230,767	+	230,767	0,0357042	362.0729367
223,309	-223,309	+	223,309	0,0382648	365.0528936
250,966	-250,966	+	250,966	0,029578	366.0492667
265,057	-265,057	+	265,057	0,0272941	366.103951
240,705	-240,705	+	240,705	0,032691	367.0685071
211,716	-211,716	+	211,716	0,0435266	367.1195946
22,759	-22,759	+	22,759	0,0365645	367.1241897
219,415	-219,415	+	219,415	0,0404379	368.0945765
219,595	-219,595	+	219,595	0,0403855	369.1146556
199,079	-199,079	+	199,079	0,0488558	371.0660834
326,897	-326,897	+	326,897	0,00890566	371.089061
203,357	-203,357	+	203,357	0,0470658	373.0942936
202,195	-202,195	+	202,195	0,0474879	374.0661287
23,694	-23,694	+	23,694	0,0333922	374.0896784
234,754	-234,754	+	234,754	0,0341333	374.0896792
237,051	-237,051	+	237,051	0,033331	375.0738051
216,176	-216,176	+	216,176	0,0412156	375.1099337
230,614	-230,614	+	230,614	0,0355534	375.1099768
251,959	-251,959	+	251,959	0,0292178	376.0881951
218,628	-218,628	+	218,628	0,0406746	376.1053156
240,132	-240,132	+	240,132	0,0326491	378.1038912
264,659	-264,659	+	264,659	0,0273742	379.1054817
230,355	-230,355	+	230,355	0,035707	381.1146915
200,801	-200,801	+	200,801	0,048253	383.0632845
23,091	-23,091	+	23,091	0,0356645	384.0569749
237,751	-237,751	+	237,751	0,0332208	385.0774785
244,442	-244,442	+	244,442	0,0311935	386.0726901

250,366	-250,366	+	250,366	0,0293812	387.0932432
207,284	-207,284	+	207,284	0,0456626	387.1100239
218,138	-218,138	+	218,138	0,0407656	387.1100949
260,768	-260,768	+	260,768	0,0280789	388.0885014
21,103	-21,103	+	21,103	0,0438409	388.0948106
207,661	-207,661	+	207,661	0,0453274	388.1053863
282,553	-282,553	+	282,553	0,0219429	389.0775746
223,724	-223,724	+	223,724	0,038311	389.0904666
211,665	-211,665	+	211,665	0,0435461	389.1152147
253,124	-253,124	+	253,124	0,0289496	389.1255099
277,352	-277,352	+	277,352	0,0240462	390.0848099
269,983	-269,983	+	269,983	0,0260537	390.1043193
271,983	-271,983	+	271,983	0,0255524	391.105753
222,545	-222,545	+	222,545	0,038491	392.1006103
236,611	-236,611	+	236,611	0,0334411	393.1148949
214,391	-214,391	+	214,391	0,0420293	393.1229534
20,381	-20,381	+	20,381	0,046788	395.0635536
204,837	-204,837	+	204,837	0,0464858	396.0667419
237,437	-237,437	+	237,437	0,0332264	399.0944802
216,452	-216,452	+	216,452	0,0411863	400.0756214
232,046	-232,046	+	232,046	0,0352368	402.0983924
245,919	-245,919	+	245,919	0,0307841	402.1040149
254,123	-254,123	+	254,123	0,0289533	402.1208964
279,646	-279,646	+	279,646	0,0231905	403.0555519
239,849	-239,849	+	239,849	0,0326679	403.0678553
200,016	-200,016	+	200,016	0,048696	403.0987985
26,033	-26,033	+	26,033	0,0281341	403.1056042
203,778	-203,778	+	203,778	0,0467851	404.0638654
210,991	-210,991	+	210,991	0,0438084	404.0945443
260,402	-260,402	+	260,402	0,0281793	404.1004855
199,018	-199,018	+	199,018	0,0487733	405.0842083
252,122	-252,122	+	252,122	0,029108	405.1147775
275,346	-275,346	+	275,346	0,024461	406.0796826
222,883	-222,883	+	222,883	0,0382929	406.1163649
228,954	-228,954	+	228,954	0,0360495	408.0947617
243,398	-243,398	+	243,399	0,0316386	413.0835273
216,055	-216,055	+	216,055	0,0412679	414.1044223
223,912	-223,912	+	223,912	0,0381829	416.0943714

286,857	-286,857	+	286,857	0,0200513	416.1006483
254,325	-254,325	+	254,325	0,029072	417.0845231
215,202	-215,202	+	215,202	0,0418109	417.1043975
212,273	-212,273	+	212,273	0,0434539	417.1143864
208,832	-208,832	+	208,832	0,0446067	417.1213344
210,172	-210,172	+	210,172	0,044	418.1059906
363,569	-363,569	+	363,569	0,0035873	418.1164467
221,286	-221,286	+	221,286	0,0392623	418.12334
206,618	-206,618	+	206,618	0,0455784	419.0627433
238,671	-238,671	+	238,671	0,0331544	419.0749605
19,787	-19,787	+	19,787	0,0495489	419.0940071
253,755	-253,755	+	253,755	0,0289246	419.1013435
237,213	-237,213	+	237,213	0,0333144	419.11364
257,525	-257,525	+	257,525	0,028957	420.0953224
207,701	-207,701	+	207,701	0,0454384	420.1333747
199,158	-199,158	+	199,158	0,0489004	421.0789247
203,191	-203,191	+	203,191	0,0469611	422.0746491
254,126	-254,126	+	254,126	0,0290246	422.1133525
341,102	-341,102	+	341,102	0,00642254	423.1455828
23,374	-23,374	+	23,374	0,0346261	426.1041848
250,931	-250,931	+	250,931	0,0295561	429.1146626
25,167	-25,167	+	25,167	0,0292372	431.0885899
358,698	-358,698	+	358,698	0,00418321	431.1115975
303,058	-303,058	+	303,058	0,0143116	432.0952555
239,286	-239,286	+	239,286	0,0329832	433.1156861
330,852	-330,852	+	330,852	0,00812987	434.111516
205,289	-205,289	+	205,289	0,0462095	435.0946328
226,328	-226,328	+	226,328	0,0373154	435.1453236
235,669	-235,669	+	235,669	0,0339233	436.0882636
243,263	-243,263	+	243,263	0,0316088	438.1039571
263,674	-263,674	+	263,674	0,0277212	441.1144929
217,436	-217,436	+	217,436	0,0409678	443.1253033
207,101	-207,101	+	207,101	0,0456184	445.0907245
272,536	-272,536	+	272,536	0,0258	445.1050028
256,811	-256,811	+	256,811	0,0290292	446.0986377
263,465	-263,465	+	263,465	0,027759	447.1064745
29,906	-29,906	+	29,906	0,0156505	447.1444395
236,187	-236,187	+	236,187	0,0335009	448.0891047

230,346	-230,346	+	230,346	0,0356566	449.1249863
230,194	-230,194	+	230,194	0,0356076	451.1242259
22,558	-22,558	+	22,558	0,0375471	457.1043667
2,49	-2,49	+	2,49	0,0299249	459.0686102
288,051	-288,051	+	288,051	0,0195478	459.083695
199,882	-199,882	+	199,882	0,0486621	459.1065795
229,119	-229,119	+	229,119	0,0360995	460.0892927
209,183	-209,183	+	209,183	0,0445611	460.0898491
321,604	-321,604	+	321,604	0,00954386	461.0845643
437,752	-437,752	+	437,752	0,0004	461.098438
246,252	-246,252	+	246,252	0,0307738	462.1047835
291,113	-291,113	+	291,113	0,0185045	463.1013795
238,765	-238,765	+	238,765	0,033203	463.1259018
254,519	-254,519	+	254,519	0,0290673	465.1147575
323,058	-323,058	+	323,058	0,00937349	473.0982597
204,258	-204,258	+	204,258	0,0467946	477.0822449
251,048	-251,048	+	251,048	0,0295724	477.1156539
298,641	-298,641	+	298,641	0,0158841	483.1172009
269,094	-269,094	+	269,094	0,0262781	485.1354237
214,677	-214,677	+	214,677	0,0417669	492.152302
244,363	-244,363	+	244,363	0,0312276	493.1371121
211,736	-211,736	+	211,736	0,0435403	495.0817825
22,536	-22,536	+	22,536	0,0376975	496.1134928
199,359	-199,359	+	199,359	0,0488038	497.0965316
209,122	-209,122	+	209,122	0,0445605	509.0840403
23,957	-23,957	+	23,957	0,0328299	509.1087761
225,852	-225,852	+	225,852	0,0374993	511.1004782
224,093	-224,093	+	224,093	0,0382619	511.1249059
240,629	-240,629	+	240,629	0,0326743	512.1058821
238,746	-238,746	+	238,746	0,0331565	513.1162664
215,143	-215,143	+	215,143	0,0416952	514.1495565
255,778	-255,778	+	255,778	0,0290283	515.1316135
240,849	-240,849	+	240,849	0,0326308	517.1466628
243,327	-243,327	+	243,327	0,0316713	524.1075177
228,822	-228,822	+	228,822	0,0360185	525.1156318
199,639	-199,639	+	199,639	0,0487791	525.1296789
205,581	-205,581	+	205,581	0,0461493	525.1533667
19,813	-19,813	+	19,813	0,049327	525.1680191

22,341	-22,341	+	22,341	0,0383083	526.1364841
240,367	-240,367	+	240,367	0,0326072	527.1317181
248,021	-248,021	+	248,021	0,0303094	529.1468633
222,954	-222,954	+	222,954	0,0382905	531.1635507
21,056	-21,056	+	21,056	0,0438566	535.0997821
224,485	-224,485	+	224,485	0,0382558	537.1161477
207,838	-207,838	+	207,838	0,045317	537.1284763
205,475	-205,475	+	205,475	0,0461574	537.141238
208,364	-208,364	+	208,364	0,0449944	538.1364244
23,305	-23,305	+	23,305	0,0348562	538.148797
259,851	-259,851	+	259,851	0,0283222	539.107972
264,004	-264,004	+	264,004	0,0275502	539.132045
255,725	-255,725	+	255,725	0,0289641	539.1574317
206,814	-206,814	+	206,814	0,0455418	540.1529791
209,522	-209,522	+	209,522	0,0443595	541.0984758
244,314	-244,314	+	244,314	0,0312049	541.1476953
240,386	-240,386	+	240,386	0,0327238	542.1439045
362,503	-362,503	+	362,503	0,00371875	543.127428
230,761	-230,761	+	230,761	0,0355897	543.1783397
205,195	-205,195	+	205,195	0,0461964	551.0955807
531,012	-531,012	+	531,012	0	551.1183222
201,537	-201,537	+	201,537	0,04788	551.1323546
205,964	-205,964	+	205,964	0,0458952	551.157368
206,529	-206,529	+	206,529	0,0455345	553.1370828
224,816	-224,816	+	224,816	0,0381197	553.1479581
273,688	-273,688	+	273,688	0,0253431	554.1434774
210,088	-210,088	+	210,088	0,0440412	555.1781258
22,701	-22,701	+	22,701	0,0367868	556.1574985
231,302	-231,302	+	231,302	0,0356607	559.1739009
28,411	-28,411	+	28,411	0,0213054	565.1593324
400,803	-400,803	+	400,803	0,00149495	567.1389856
244,804	-244,804	+	244,804	0,0312412	567.1770476
206,954	-206,954	+	206,954	0,0455529	575.1687765
240,479	-240,479	+	240,479	0,0327189	581.1069037
234,076	-234,076	+	234,076	0,0345826	581.1188296
289,063	-289,063	+	289,063	0,0190526	581.155413
252,449	-252,449	+	252,449	0,0291064	583.1103142
202,639	-202,639	+	202,639	0,0470828	583.12282

204,487	-204,487	+	204,487	0,0466207	583.1359289
241,503	-241,503	+	241,503	0,0325938	583.1483173
224,117	-224,117	+	224,117	0,0382939	583.173373
203,303	-203,303	+	203,303	0,0470709	585.1877398
246,669	-246,669	+	246,669	0,0305902	593.1790133
245,413	-245,413	+	245,413	0,0310333	595.1094927
251,574	-251,574	+	251,574	0,0292715	595.111015
223,598	-223,598	+	223,598	0,0382727	595.1478811
302,232	-302,232	+	302,232	0,0144433	597.1148148
305,246	-305,246	+	305,246	0,0135179	597.1272337
206,048	-206,048	+	206,048	0,0459227	599.116521
20,108	-20,108	+	20,108	0,0480794	599.1675437
241,857	-241,857	+	241,857	0,0324882	601.1349056
198,309	-198,309	+	198,309	0,0492125	601.1587683
243,208	-243,208	+	243,208	0,0315547	605.1675738
233,101	-233,101	+	233,101	0,0348543	611.129238
25,768	-25,768	+	25,768	0,028938	613.1467579
266,177	-266,177	+	266,177	0,0269022	615.1626901
209,859	-209,859	+	209,859	0,0441966	616.146554
250,414	-250,414	+	250,414	0,0294865	631.1577979
211,604	-211,604	+	211,604	0,0435797	631.1691298
571,177	-571,177	+	571,177	0	633.1744993
279,443	-279,443	+	279,443	0,0231654	643.1574039
206,971	-206,971	+	206,971	0,0455939	647.1290577
221,555	-221,555	+	221,555	0,0390947	653.178372
296,232	-296,232	+	296,232	0,0167619	655.1945489
235,587	-235,587	+	235,587	0,0338163	659.1533217
245,386	-245,386	+	245,386	0,0309688	659.1879205
250,741	-250,741	+	250,741	0,0294636	661.1821817
20,926	-20,926	+	20,926	0,0445119	665.1775369
388,126	-388,126	+	388,126	0,00215094	675.1845749
251,893	-251,893	+	251,893	0,029215	685.1938446
510,069	-510,069	+	510,069	7.55E+00	689.1764731
374,283	-374,283	+	374,283	0,00280342	719.1762392
25,077	-25,077	+	25,077	0,0295308	733.1920308
288,824	-288,824	+	288,824	0,0191441	778.5162159
295,168	-295,168	+	295,168	0,017346	835.527128
238,228	-238,228	+	238,228	0,0332896	863.5578143

Supplementary Table 5: Annotations for infection of BUVEC in positive-ion mode

Input Mass	Matched Mass	Delta	ppm	Name	Formula	Adduct
313.2738392	313.2737	0.0001	0.3192	MG(16:0)	C19H38O4	[M+H-H2O]+
339.2894868	339.2894	0.0001	0.2947	MG(18:1)	C21H40O4	[M+H-H2O]+
366.3366396	366.3367	0	0.0000	WE(23:3)	C23H40O2	[M+NH4]+
431.216853	431.2169	0.0001	0.2319	LPA(16:1)	C19H37O7PNa	[M+Na]+
445.2714709	445.2713	0.0001	0.2246	LPA(20:2)	C23H43O7P	[M+H-H2O]+
445.2714709	445.2715	0	0.0000	MG(22:4)	C25H42O4K	[M+K]+
450.2978033	450.2979	0.0001	0.2221	LPC(14:0)	C22H46NO7P	[M+H-H2O]+
450.2978033	450.2979	0.0001	0.2221	LPE(17:0)	C22H46NO7P	[M+H-H2O]+
450.2978033	450.2979	0.0001	0.2221	PC(O-14:0)	C22H46NO7P	[M+H-H2O]+
450.2978033	450.298	0.0002	0.4442	CAR(17:1)	C24H45NO4K	[M+K]+
468.3086543	468.3084	0.0002	0.4271	CAR(20:5)	C27H43NO4Na	[M+Na]+
468.3086543	468.3085	0.0002	0.4271	LPA(19:1)	C22H43O7P	[M+NH4]+
468.3086543	468.3085	0.0002	0.4271	LPC(14:0)	C22H46NO7P	[M+H]+
468.3086543	468.3085	0.0002	0.4271	LPE(17:0)	C22H46NO7P	[M+H]+
468.3086543	468.3085	0.0002	0.4271	PC(O-14:0)	C22H46NO7P	[M+H]+
490.2896049	490.2904	0.0008	1.6317	LPC(14:0)	C22H46NO7PNa	[M+Na]+
490.2896049	490.2904	0.0008	1.6317	LPE(17:0)	C22H46NO7PNa	[M+Na]+
490.2896049	490.2904	0.0008	1.6317	PC(O-14:0)	C22H46NO7PNa	[M+Na]+
490.2905522	490.2904	0.0001	0.2040	LPC(14:0)	C22H46NO7PNa	[M+Na]+
490.2905522	490.2904	0.0001	0.2040	LPE(17:0)	C22H46NO7PNa	[M+Na]+
490.2905522	490.2904	0.0001	0.2040	PC(O-14:0)	C22H46NO7PNa	[M+Na]+
495.4410303	495.4408	0.0003	0.6055	DG(28:0)	C31H60O5	[M+H-H2O]+
521.4568736	521.4564	0.0004	0.7671	DG(30:1)	C33H62O5	[M+H-H2O]+
522.3193362	522.319	0.0003	0.5744	LPS(19:0)	C25H50NO9P	[M+H-H2O]+
522.3193362	522.319	0.0003	0.5744	PE(20:0(OH))	C25H50NO9P	[M+H-H2O]+
523.4725108	523.4721	0.0004	0.7641	DG(30:0)	C33H64O5	[M+H-H2O]+
536.3351027	536.3347	0.0004	0.7458	LPS(20:0)	C26H52NO9P	[M+H-H2O]+
539.2749142	539.2768	0.0019	3.5232	LPG22:6)	C28H45O9P	[M+H-H2O]+
549.4881916	549.4877	0.0005	0.9099	DG(32:1)	C35H66O5	[M+H-H2O]+
563.4637913	563.4646	0.0008	1.4198	DG(30:0)	C33H64O5Na	[M+Na]+
576.3647394	576.366	0.0012	2.0820	PE(24:1(OH))	C29H56NO9P	[M+H-H2O]+
576.4740891	576.4752	0.0012	2.0816	Cer(d34:1)	C34H67NO3K	[M+K]+
579.437376	579.4384	0.001	1.7258	LPA(28:0)	C31H63O7P	[M+H]+
579.437376	579.4384	0.001	1.7258	PA(O-28:0)	C31H63O7P	[M+H]+

579.437376	579.4385	0.0012	2.0710	DG(30:0)	C33H64O5K	[M+K]+
579.533395	579.5347	0.0013	2.2432	DG(34:0)	C37H72O5	[M+H-H2O]+
589.4790047	589.4802	0.0012	2.0357	DG(32:1)	C35H66O5Na	[M+Na]+
589.4802031	589.4802	0	0.0000	DG(32:1)	C35H66O5Na	[M+Na]+
589.5541403	589.5554	0.0013	2.2051	DG(O-36:2)	C39H74O4	[M+H-H2O]+
589.5541403	589.5554	0.0013	2.2051	DG(P-36:1)	C39H74O4	[M+H-H2O]+
591.4946232	591.4959	0.0013	2.1978	DG(32:0)	C35H68O5Na	[M+Na]+
603.5334352	603.5323	0.0012	1.9883	DG(O-18:1)	C37H72O4Na	[M+Na]+
603.5334352	603.5323	0.0012	1.9883	DG(O-34:1)	C37H72O4Na	[M+Na]+
603.5334352	603.5323	0.0012	1.9883	DG(P-34:0)	C37H72O4Na	[M+Na]+
603.5334352	603.5347	0.0012	1.9883	DG(36:2)	C39H72O5	[M+H-H2O]+
604.3961634	604.3973	0.0011	1.8200	LPS(25:1)	C31H60NO9P	[M+H-H2O]+
604.3961634	604.3973	0.0011	1.8200	PA(28:3)	C31H55O8P	[M+NH4]+
604.3961634	604.3973	0.0011	1.8200	PE(26:1(OH))	C31H60NO9P	[M+H-H2O]+
605.4529894	605.4541	0.0011	1.8168	LPA(30:1)	C33H65O7P	[M+H]+
605.4529894	605.4541	0.0011	1.8168	PA(O-30:0(OH))	C33H67O8P	[M+H-H2O]+
605.4529894	605.4541	0.0011	1.8168	PA(O-30:1)	C33H65O7P	[M+H]+
605.4529894	605.4541	0.0011	1.8168	PA(P-30:0)	C33H65O7P	[M+H]+
605.4529894	605.4542	0.0012	1.9820	DG(32:1)	C35H66O5K	[M+K]+
605.5484904	605.5479	0.0006	0.9908	DG(O-34:0)	C37H74O4Na	[M+Na]+
605.5484904	605.5479	0.0006	0.9908	MG(34:0)	C37H74O4Na	[M+Na]+
605.5502236	605.5503	0.0001	0.1651	DG(36:1)	C39H74O5	[M+H-H2O]+
605.5502236	605.5503	0.0001	0.1651	TG(P-36:0)	C39H74O5	[M+H-H2O]+
611.367642	611.3683	0.0007	1.1450	PA(28:2)	C31H57O8PNa	[M+Na]+
612.5554329	612.5561	0.0007	1.1428	DG(34:1)	C37H70O5	[M+NH4]+
617.5109335	617.5115	0.0006	0.9716	DG(34:1)	C37H70O5Na	[M+Na]+
619.5264527	619.5272	0.0007	1.1299	DG(34:0)	C37H72O5Na	[M+Na]+
631.5653414	631.566	0.0006	0.9500	DG(38:2)	C41H76O5	[M+H-H2O]+
633.4847518	633.4854	0.0006	0.9471	LPA(32:1)	C35H69O7P	[M+H]+
633.4847518	633.4854	0.0006	0.9471	PA(O-32:0(OH))	C35H71O8P	[M+H-H2O]+
633.4847518	633.4854	0.0006	0.9471	PA(O-32:1)	C35H69O7P	[M+H]+
633.4847518	633.4854	0.0006	0.9471	PA(P-32:0)	C35H69O7P	[M+H]+
633.4847518	633.4855	0.0007	1.1050	DG(34:1)	C37H70O5K	[M+K]+
633.5811105	633.5816	0.0005	0.7892	DG(38:1)	C41H78O5	[M+H-H2O]+
633.5811105	633.5816	0.0005	0.7892	TG(P-38:0)	C41H78O5	[M+H-H2O]+
634.4876089	634.4889	0.0012	1.8913	HexCer(t28:0)	C34H67NO9	[M+H]+
635.498876	635.501	0.0021	3.3045	LPA(32:0)	C35H71O7P	[M+H]+
635.498876	635.501	0.0021	3.3045	PA(O-32:0)	C35H71O7P	[M+H]+

635.498876	635.5011	0.0023	3.6192	DG(34:0)	C37H72O5K	[M+K]+
635.5004957	635.501	0.0005	0.7868	LPA(32:0)	C35H71O7P	[M+H]+
635.5004957	635.501	0.0005	0.7868	PA(O-32:0)	C35H71O7P	[M+H]+
635.5004957	635.5011	0.0006	0.9441	DG(34:0)	C37H72O5K	[M+K]+
641.4145216	641.4137	0.0009	1.4032	PS(24:0)	C30H58NO10P	[M+NH4]+
641.4145216	641.4153	0.0008	1.2472	PA(30:1)	C33H63O8PNa	[M+Na]+
641.4145216	641.4153	0.0008	1.2472	PA(P-30:1(OH))	C33H63O8PNa	[M+Na]+
643.4292365	643.4309	0.0017	2.6421	PA(30:0)	C33H65O8PNa	[M+Na]+
643.4292365	643.4309	0.0017	2.6421	PA(O-30:1(OH))	C33H65O8PNa	[M+Na]+
643.4292365	643.4309	0.0017	2.6421	PA(P-30:0(OH))	C33H65O8PNa	[M+Na]+
643.4305846	643.4309	0.0003	0.4663	PA(30:0)	C33H65O8PNa	[M+Na]+
643.4305846	643.4309	0.0003	0.4663	PA(O-30:1(OH))	C33H65O8PNa	[M+Na]+
643.4305846	643.4309	0.0003	0.4663	PA(P-30:0(OH))	C33H65O8PNa	[M+Na]+
643.5266973	643.5272	0.0005	0.7770	DG(36:2)	C39H72O5Na	[M+Na]+
645.5423295	645.5428	0.0005	0.7745	DG(36:1)	C39H74O5Na	[M+Na]+
645.5423295	645.5428	0.0005	0.7745	TG(P-36:0)	C39H74O5Na	[M+Na]+
648.4591248	648.4599	0.0008	1.2337	LPS(28:0)	C34H68NO9P	[M+H-H2O]+
648.4591248	648.4599	0.0008	1.2337	PA(31:2)	C34H63O8P	[M+NH4]+
648.4591248	648.4599	0.0008	1.2337	PC(26:0(OH))	C34H68NO9P	[M+H-H2O]+
648.4591248	648.4599	0.0008	1.2337	PC(26:1)	C34H66NO8P	[M+H]+
648.4591248	648.4599	0.0008	1.2337	PE(29:0(OH))	C34H68NO9P	[M+H-H2O]+
648.4591248	648.4599	0.0008	1.2337	PE(29:1)	C34H66NO8P	[M+H]+
648.4591248	648.4599	0.0008	1.2337	PS(O-28:0)	C34H68NO9P	[M+H-H2O]+
648.4604502	648.4599	0.0006	0.9253	LPS(28:0)	C34H68NO9P	[M+H-H2O]+
648.4604502	648.4599	0.0006	0.9253	PA(31:2)	C34H63O8P	[M+NH4]+
648.4604502	648.4599	0.0006	0.9253	PC(26:0(OH))	C34H68NO9P	[M+H-H2O]+
648.4604502	648.4599	0.0006	0.9253	PC(26:1)	C34H66NO8P	[M+H]+
648.4604502	648.4599	0.0006	0.9253	PE(29:0(OH))	C34H68NO9P	[M+H-H2O]+
648.4604502	648.4599	0.0006	0.9253	PE(29:1)	C34H66NO8P	[M+H]+
648.4604502	648.4599	0.0006	0.9253	PS(O-28:0)	C34H68NO9P	[M+H-H2O]+
653.5494179	653.5479	0.0015	2.2952	DG(O-38:4)	C41H74O4Na	[M+Na]+
653.5494179	653.5479	0.0015	2.2952	DG(P-38:3)	C41H74O4Na	[M+Na]+
653.5494179	653.5503	0.0009	1.3771	DG(40:5)	C43H74O5	[M+H-H2O]+
653.5503546	653.5503	0	0.0000	DG(40:5)	C43H74O5	[M+H-H2O]+
654.4095834	654.4105	0.0009	1.3753	PE(28:2)	C33H62NO8PNa	[M+Na]+
654.4104004	654.4105	0.0001	0.1528	PE(28:2)	C33H62NO8PNa	[M+Na]+
659.4995527	659.501	0.0015	2.2744	PA(34:0)	C37H73O8P	[M+H-H2O]+
659.4995527	659.501	0.0015	2.2744	PA(O-34:1(OH))	C37H73O8P	[M+H-H2O]+

659.4995527	659.501	0.0015	2.2744	PA(O-34:2)	C37H71O7P	[M+H]+
659.4995527	659.501	0.0015	2.2744	PA(P-34:0(OH))	C37H73O8P	[M+H-H2O]+
659.4995527	659.501	0.0015	2.2744	PA(P-34:1)	C37H71O7P	[M+H]+
659.4995527	659.5011	0.0016	2.4261	DG(36:2)	C39H72O5K	[M+K]+
659.5006899	659.501	0.0003	0.4549	PA(34:0)	C37H73O8P	[M+H-H2O]+
659.5006899	659.501	0.0003	0.4549	PA(O-34:1(OH))	C37H73O8P	[M+H-H2O]+
659.5006899	659.501	0.0003	0.4549	PA(O-34:2)	C37H71O7P	[M+H]+
659.5006899	659.501	0.0003	0.4549	PA(P-34:0(OH))	C37H73O8P	[M+H-H2O]+
659.5006899	659.501	0.0003	0.4549	PA(P-34:1)	C37H71O7P	[M+H]+
659.5006899	659.5011	0.0004	0.6065	DG(36:2)	C39H72O5K	[M+K]+
660.5039679	660.5045	0.0005	0.7570	HexCer(t30:1)	C36H69NO9	[M+H]+
661.5160511	661.5167	0.0006	0.9070	PA(O-34:0(OH))	C37H75O8P	[M+H-H2O]+
661.5160511	661.5167	0.0006	0.9070	PA(O-34:1)	C37H73O7P	[M+H]+
661.5160511	661.5167	0.0006	0.9070	PA(P-34:0)	C37H73O7P	[M+H]+
661.5160511	661.5168	0.0007	1.0582	DG(36:1)	C39H74O5K	[M+K]+
661.5160511	661.5168	0.0007	1.0582	TG(P-36:0)	C39H74O5K	[M+K]+
662.4748226	662.4755	0.0007	1.0566	LPS(29:0)	C35H70NO9P	[M+H-H2O]+
662.4748226	662.4755	0.0007	1.0566	PA(32:2)	C35H65O8P	[M+NH4]+
662.4748226	662.4755	0.0007	1.0566	PC(27:0(OH))	C35H70NO9P	[M+H-H2O]+
662.4748226	662.4755	0.0007	1.0566	PC(27:1)	C35H68NO8P	[M+H]+
662.4748226	662.4755	0.0007	1.0566	PE(30:0(OH))	C35H70NO9P	[M+H-H2O]+
662.4748226	662.4755	0.0007	1.0566	PE(30:1)	C35H68NO8P	[M+H]+
662.4748226	662.4755	0.0007	1.0566	PE(P-30:1(OH))	C35H68NO8P	[M+H]+
662.4748226	662.4755	0.0007	1.0566	PS(O-29:0)	C35H70NO9P	[M+H-H2O]+
663.3978801	663.3996	0.0017	2.5626	PA(32:4)	C35H61O8PNa	[M+Na]+
663.3978801	663.3998	0.0019	2.8640	LPG26:0)	C32H65O9PK	[M+K]+
663.3978801	663.3998	0.0019	2.8640	PGO-26:0)	C32H65O9PK	[M+K]+
664.4911038	664.4912	0.0001	0.1505	PA(32:1)	C35H67O8P	[M+NH4]+
664.4911038	664.4912	0.0001	0.1505	PA(P-32:1(OH))	C35H67O8P	[M+NH4]+
664.4911038	664.4912	0.0001	0.1505	PC(27:0)	C35H70NO8P	[M+H]+
664.4911038	664.4912	0.0001	0.1505	PE(30:0)	C35H70NO8P	[M+H]+
664.4911038	664.4912	0.0001	0.1505	PE(O-30:1(OH))	C35H70NO8P	[M+H]+
664.4911038	664.4912	0.0001	0.1505	PE(P-30:0(OH))	C35H70NO8P	[M+H]+
664.4911038	664.4912	0.0001	0.1505	PENMe2(28:0)	C35H70NO8P	[M+H]+
665.4125337	665.4153	0.0027	4.0576	PA(32:3)	C35H63O8PNa	[M+Na]+
666.4157593	666.4129	0.0028	4.2016	LPS(30:5)	C36H62NO9P	[M+H-H2O]+
667.4296579	667.4293	0.0004	0.5993	PS(26:1)	C32H60NO10P	[M+NH4]+
667.4305618	667.4309	0.0004	0.5993	PA(32:2)	C35H65O8PNa	[M+Na]+

668.4334952	668.4368	0.0033	4.9369	MGDG(27:5)	C36H58O10	[M+NH4]+
669.42833	669.4256	0.0027	4.0333	PA(P-32:1)	C35H67O7PK	[M+K]+
669.4371523	669.4368	0.0003	0.4481	PECer(d32:2)	C34H67N2O6PK	[M+K]+
669.4371523	669.4368	0.0003	0.4481	SMd29:2)	C34H67N2O6PK	[M+K]+
669.4458437	669.445	0.0009	1.3444	PS(26:0)	C32H62NO10P	[M+NH4]+
669.4458437	669.445	0.0009	1.3444	PS(P-26:0(OH))	C32H62NO10P	[M+NH4]+
669.4458437	669.4466	0.0007	1.0456	PA(32:1)	C35H67O8PNa	[M+Na]+
669.4458437	669.4466	0.0007	1.0456	PA(P-32:1(OH))	C35H67O8PNa	[M+Na]+
670.4501467	670.4525	0.0023	3.4305	MGDG(27:4)	C36H60O10	[M+NH4]+
671.4443459	671.4412	0.0031	4.6169	LPA(32:1)	C35H69O7PK	[M+K]+
671.4443459	671.4412	0.0031	4.6169	PA(O-32:1)	C35H69O7PK	[M+K]+
671.4443459	671.4412	0.0031	4.6169	PA(P-32:0)	C35H69O7PK	[M+K]+
671.452394	671.4525	0.0001	0.1489	PECer(d32:1)	C34H69N2O6PK	[M+K]+
671.452394	671.4525	0.0001	0.1489	SMd29:1)	C34H69N2O6PK	[M+K]+
671.5579976	671.5585	0.0005	0.7445	DG(38:2)	C41H76O5Na	[M+Na]+
672.4567131	672.4575	0.0008	1.1897	PC(26:0)	C34H68NO8PNa	[M+Na]+
672.4567131	672.4575	0.0008	1.1897	PC(P-26:0(OH))	C34H68NO8PNa	[M+Na]+
672.4567131	672.4575	0.0008	1.1897	PE(29:0)	C34H68NO8PNa	[M+Na]+
672.4567131	672.4575	0.0008	1.1897	PE(P-29:0(OH))	C34H68NO8PNa	[M+Na]+
672.4567131	672.4575	0.0008	1.1897	PENMe(28:0)	C34H68NO8PNa	[M+Na]+
676.4911324	676.4912	0	0.0000	LPS(30:0)	C36H72NO9P	[M+H-H2O]+
676.4911324	676.4912	0	0.0000	PA(33:2)	C36H67O8P	[M+NH4]+
676.4911324	676.4912	0	0.0000	PA(P-33:2(OH))	C36H67O8P	[M+NH4]+
676.4911324	676.4912	0	0.0000	PC(28:0(OH))	C36H72NO9P	[M+H-H2O]+
676.4911324	676.4912	0	0.0000	PC(28:1)	C36H70NO8P	[M+H]+
676.4911324	676.4912	0	0.0000	PE(31:0(OH))	C36H72NO9P	[M+H-H2O]+
676.4911324	676.4912	0	0.0000	PE(31:1)	C36H70NO8P	[M+H]+
676.4911324	676.4912	0	0.0000	PE(P-31:1(OH))	C36H70NO8P	[M+H]+
676.4911324	676.4912	0	0.0000	PS(O-30:0)	C36H72NO9P	[M+H-H2O]+
678.5067599	678.5068	0.0001	0.1474	PA(33:1)	C36H69O8P	[M+NH4]+
678.5067599	678.5068	0.0001	0.1474	PA(O-33:2(OH))	C36H69O8P	[M+NH4]+
678.5067599	678.5068	0.0001	0.1474	PA(P-33:1(OH))	C36H69O8P	[M+NH4]+
678.5067599	678.5068	0.0001	0.1474	PC(28:0)	C36H72NO8P	[M+H]+
678.5067599	678.5068	0.0001	0.1474	PC(P-28:0(OH))	C36H72NO8P	[M+H]+
678.5067599	678.5068	0.0001	0.1474	PE(31:0)	C36H72NO8P	[M+H]+
678.5067599	678.5068	0.0001	0.1474	PE(O-31:1(OH))	C36H72NO8P	[M+H]+
678.5067599	678.5068	0.0001	0.1474	PE(P-31:0(OH))	C36H72NO8P	[M+H]+
685.4196608	685.4205	0.0009	1.3131	PA(32:1)	C35H67O8PK	[M+K]+

685.4196608	685.4205	0.0009	1.3131	PA(P-32:1(OH))	C35H67O8PK	[M+K]+
685.4204589	685.4205	0.0001	0.1459	PA(32:1)	C35H67O8PK	[M+K]+
685.4204589	685.4205	0.0001	0.1459	PA(P-32:1(OH))	C35H67O8PK	[M+K]+
686.4238095	686.4239	0.0001	0.1457	PICer(t26:0)	C32H64NO12P	[M+H]+
686.4744169	686.4731	0.0013	1.8937	PC(27:0)	C35H70NO8PNa	[M+Na]+
686.4744169	686.4731	0.0013	1.8937	PE(30:0)	C35H70NO8PNa	[M+Na]+
686.4744169	686.4731	0.0013	1.8937	PE(O-30:1(OH))	C35H70NO8PNa	[M+Na]+
686.4744169	686.4731	0.0013	1.8937	PE(P-30:0(OH))	C35H70NO8PNa	[M+Na]+
686.4744169	686.4731	0.0013	1.8937	PENMe2(28:0)	C35H70NO8PNa	[M+Na]+
686.4744169	686.4755	0.0011	1.6024	PA(34:4)	C37H65O8P	[M+NH4]+
686.4744169	686.4755	0.0011	1.6024	PA(P-34:4(OH))	C37H65O8P	[M+NH4]+
686.4744169	686.4755	0.0011	1.6024	PC(29:2(OH))	C37H70NO9P	[M+H-H2O]+
686.4744169	686.4755	0.0011	1.6024	PE(32:2(OH))	C37H70NO9P	[M+H-H2O]+
686.4744169	686.4755	0.0011	1.6024	PE(32:3)	C37H68NO8P	[M+H]+
686.4744169	686.4755	0.0011	1.6024	PS(P-31:1)	C37H70NO9P	[M+H-H2O]+
691.4285473	691.4293	0.0008	1.1570	PS(28:3)	C34H60NO10P	[M+NH4]+
692.4319759	692.4286	0.0034	4.9103	LPS(32:6)	C38H64NO9P	[M+H-H2O]+
692.5224818	692.5225	0	0.0000	PA(34:1)	C37H71O8P	[M+NH4]+
692.5224818	692.5225	0	0.0000	PA(O-34:2(OH))	C37H71O8P	[M+NH4]+
692.5224818	692.5225	0	0.0000	PA(P-34:1(OH))	C37H71O8P	[M+NH4]+
692.5224818	692.5225	0	0.0000	PC(29:0)	C37H74NO8P	[M+H]+
692.5224818	692.5225	0	0.0000	PC(P-29:0(OH))	C37H74NO8P	[M+H]+
692.5224818	692.5225	0	0.0000	PE(32:0)	C37H74NO8P	[M+H]+
692.5224818	692.5225	0	0.0000	PE(O-32:1(OH))	C37H74NO8P	[M+H]+
692.5224818	692.5225	0	0.0000	PE(P-32:0(OH))	C37H74NO8P	[M+H]+
695.4536879	695.4525	0.0012	1.7255	PECer(d34:3)	C36H69N2O6PK	[M+K]+
695.4621709	695.4622	0.0001	0.1438	PA(34:2)	C37H69O8PNa	[M+Na]+
695.4621709	695.4622	0.0001	0.1438	PA(O-34:3(OH))	C37H69O8PNa	[M+Na]+
695.4621709	695.4622	0.0001	0.1438	PA(P-34:2(OH))	C37H69O8PNa	[M+Na]+
696.4655535	696.4681	0.0026	3.7331	MGDG(29:5)	C38H62O10	[M+NH4]+
698.4730924	698.4731	0	0.0000	PC(28:1)	C36H70NO8PNa	[M+Na]+
698.4730924	698.4731	0	0.0000	PE(31:1)	C36H70NO8PNa	[M+Na]+
698.4730924	698.4731	0	0.0000	PE(P-31:1(OH))	C36H70NO8PNa	[M+Na]+
700.3820277	700.3796	0.0024	3.4267	PS(27:2(OH))	C33H60NO11PNa	[M+Na]+
700.4887777	700.4888	0	0.0000	PC(28:0)	C36H72NO8PNa	[M+Na]+
700.4887777	700.4888	0	0.0000	PC(P-28:0(OH))	C36H72NO8PNa	[M+Na]+
700.4887777	700.4888	0	0.0000	PE(31:0)	C36H72NO8PNa	[M+Na]+
700.4887777	700.4888	0	0.0000	PE(O-31:1(OH))	C36H72NO8PNa	[M+Na]+

700.4887777	700.4888	0	0.0000	PE(P-31:0(OH))	C36H72NO8PNa	[M+Na]+
704.5224864	704.5225	0	0.0000	LPS(32:0)	C38H76NO9P	[M+H-H2O]+
704.5224864	704.5225	0	0.0000	PA(35:2)	C38H71O8P	[M+NH4]+
704.5224864	704.5225	0	0.0000	PA(P-35:2(OH))	C38H71O8P	[M+NH4]+
704.5224864	704.5225	0	0.0000	PC(30:0(OH))	C38H76NO9P	[M+H-H2O]+
704.5224864	704.5225	0	0.0000	PC(30:1)	C38H74NO8P	[M+H]+
704.5224864	704.5225	0	0.0000	PC(P-30:1(OH))	C38H74NO8P	[M+H]+
704.5224864	704.5225	0	0.0000	PE(33:0(OH))	C38H76NO9P	[M+H-H2O]+
704.5224864	704.5225	0	0.0000	PE(33:1)	C38H74NO8P	[M+H]+
704.5224864	704.5225	0	0.0000	PE(O-33:2(OH))	C38H74NO8P	[M+H]+
704.5224864	704.5225	0	0.0000	PE(P-33:1(OH))	C38H74NO8P	[M+H]+
704.5224864	704.5225	0	0.0000	PS(O-32:0)	C38H76NO9P	[M+H-H2O]+
706.5380984	706.5381	0	0.0000	PA(35:1)	C38H73O8P	[M+NH4]+
706.5380984	706.5381	0	0.0000	PA(O-35:2(OH))	C38H73O8P	[M+NH4]+
706.5380984	706.5381	0	0.0000	PA(P-35:1(OH))	C38H73O8P	[M+NH4]+
706.5380984	706.5381	0	0.0000	PC(30:0)	C38H76NO8P	[M+H]+
706.5380984	706.5381	0	0.0000	PC(O-30:1(OH))	C38H76NO8P	[M+H]+
706.5380984	706.5381	0	0.0000	PC(P-30:0(OH))	C38H76NO8P	[M+H]+
706.5380984	706.5381	0	0.0000	PE(33:0)	C38H76NO8P	[M+H]+
706.5380984	706.5381	0	0.0000	PE(O-33:1(OH))	C38H76NO8P	[M+H]+
706.5380984	706.5381	0	0.0000	PE(P-33:0(OH))	C38H76NO8P	[M+H]+
706.5380984	706.5381	0	0.0000	PENMe(32:0)	C38H76NO8P	[M+H]+
708.4574202	708.4575	0.0001	0.1412	PE(32:3)	C37H68NO8PNa	[M+Na]+
709.5474067	709.549	0.0016	2.2550	PC(29:0)	C37H74NO8P	[M+NH4]+
709.5474067	709.549	0.0016	2.2550	PC(P-29:0(OH))	C37H74NO8P	[M+NH4]+
709.5474067	709.549	0.0016	2.2550	PE(32:0)	C37H74NO8P	[M+NH4]+
709.5474067	709.549	0.0016	2.2550	PE(O-32:1(OH))	C37H74NO8P	[M+NH4]+
709.5474067	709.549	0.0016	2.2550	PE(P-32:0(OH))	C37H74NO8P	[M+NH4]+
712.3820408	712.3796	0.0024	3.3690	PS(28:3(OH))	C34H60NO11PNa	[M+Na]+
712.3820408	712.3797	0.0024	3.3690	LPIP(O-20:0)	C29H60O14P2	[M+NH4]+
713.3854816	713.3872	0.0017	2.3830	PI(24:1(OH))	C33H61O14P	[M+H]+
714.4318263	714.4317	0.0002	0.2799	PS(29:1)	C35H66NO10PNa	[M+Na]+
714.5044559	714.5044	0	0.0000	PC(29:0)	C37H74NO8PNa	[M+Na]+
714.5044559	714.5044	0	0.0000	PC(P-29:0(OH))	C37H74NO8PNa	[M+Na]+
714.5044559	714.5044	0	0.0000	PE(32:0)	C37H74NO8PNa	[M+Na]+
714.5044559	714.5044	0	0.0000	PE(O-32:1(OH))	C37H74NO8PNa	[M+Na]+
714.5044559	714.5044	0	0.0000	PE(P-32:0(OH))	C37H74NO8PNa	[M+Na]+
715.402459	715.4028	0.0004	0.5591	PI(24:0(OH))	C33H63O14P	[M+H]+

715.4353433	715.4333	0.002	2.7955	PA(38:8(OH))	C41H65O9P	[M+H-H2O]+
715.4353433	715.4333	0.002	2.7955	PA(38:9)	C41H63O8P	[M+H]+
715.507955	715.5061	0.0019	2.6555	PA(P-40:6)	C43H73O7P	[M+H-H2O]+
716.4626925	716.4626	0.0001	0.1396	LPE(34:6)	C39H68NO7PNa	[M+Na]+
716.4626925	716.4627	0	0.0000	PC(28:0)	C36H72NO8PK	[M+K]+
716.4626925	716.4627	0	0.0000	PC(P-28:0(OH))	C36H72NO8PK	[M+K]+
716.4626925	716.4627	0	0.0000	PE(31:0)	C36H72NO8PK	[M+K]+
716.4626925	716.4627	0	0.0000	PE(O-31:1(OH))	C36H72NO8PK	[M+K]+
716.4626925	716.4627	0	0.0000	PE(P-31:0(OH))	C36H72NO8PK	[M+K]+
722.4730725	722.4731	0	0.0000	PC(30:3)	C38H70NO8PNa	[M+Na]+
722.4730725	722.4731	0	0.0000	PE(33:3)	C38H70NO8PNa	[M+Na]+
726.5043886	726.5044	0	0.0000	PC(30:1)	C38H74NO8PNa	[M+Na]+
726.5043886	726.5044	0	0.0000	PC(P-30:1(OH))	C38H74NO8PNa	[M+Na]+
726.5043886	726.5044	0	0.0000	PE(33:1)	C38H74NO8PNa	[M+Na]+
726.5043886	726.5044	0	0.0000	PE(O-33:2(OH))	C38H74NO8PNa	[M+Na]+
726.5043886	726.5044	0	0.0000	PE(P-33:1(OH))	C38H74NO8PNa	[M+Na]+
727.50748	727.5038	0.0036	4.9484	PA(O-36:1)	C39H77O7PK	[M+K]+
727.50748	727.5038	0.0036	4.9484	PA(P-36:0)	C39H77O7PK	[M+K]+
728.5199054	728.5201	0.0002	0.2745	PC(30:0)	C38H76NO8PNa	[M+Na]+
728.5199054	728.5201	0.0002	0.2745	PC(O-30:1(OH))	C38H76NO8PNa	[M+Na]+
728.5199054	728.5201	0.0002	0.2745	PC(P-30:0(OH))	C38H76NO8PNa	[M+Na]+
728.5199054	728.5201	0.0002	0.2745	PE(33:0)	C38H76NO8PNa	[M+Na]+
728.5199054	728.5201	0.0002	0.2745	PE(O-33:1(OH))	C38H76NO8PNa	[M+Na]+
728.5199054	728.5201	0.0002	0.2745	PE(P-33:0(OH))	C38H76NO8PNa	[M+Na]+
728.5199054	728.5201	0.0002	0.2745	PENMe(32:0)	C38H76NO8PNa	[M+Na]+
728.5200841	728.5201	0	0.0000	PC(30:0)	C38H76NO8PNa	[M+Na]+
728.5200841	728.5201	0	0.0000	PC(O-30:1(OH))	C38H76NO8PNa	[M+Na]+
728.5200841	728.5201	0	0.0000	PC(P-30:0(OH))	C38H76NO8PNa	[M+Na]+
728.5200841	728.5201	0	0.0000	PE(33:0)	C38H76NO8PNa	[M+Na]+
728.5200841	728.5201	0	0.0000	PE(O-33:1(OH))	C38H76NO8PNa	[M+Na]+
728.5200841	728.5201	0	0.0000	PE(P-33:0(OH))	C38H76NO8PNa	[M+Na]+
728.5200841	728.5201	0	0.0000	PENMe(32:0)	C38H76NO8PNa	[M+Na]+
731.5306218	731.5334	0.0028	3.8276	PC(31:3)	C39H72NO8P	[M+NH4]+
731.5306218	731.5334	0.0028	3.8276	PE(34:3)	C39H72NO8P	[M+NH4]+
731.5306218	731.5334	0.0028	3.8276	PE(O-34:4(OH))	C39H72NO8P	[M+NH4]+
731.5306218	731.5334	0.0028	3.8276	PE(P-34:3(OH))	C39H72NO8P	[M+NH4]+
732.5336381	732.5326	0.001	1.3651	PE(O-38:6)	C43H76NO7P	[M+H-H2O]+
732.5336381	732.5326	0.001	1.3651	PE(P-38:5)	C43H76NO7P	[M+H-H2O]+

735.5636759	735.5647	0.001	1.3595	PC(31:1)	C39H76NO8P	[M+NH4]+
735.5636759	735.5647	0.001	1.3595	PC(P-31:1(OH))	C39H76NO8P	[M+NH4]+
735.5636759	735.5647	0.001	1.3595	PE(34:1)	C39H76NO8P	[M+NH4]+
735.5636759	735.5647	0.001	1.3595	PE(O-34:2(OH))	C39H76NO8P	[M+NH4]+
735.5636759	735.5647	0.001	1.3595	PE(P-34:1(OH))	C39H76NO8P	[M+NH4]+
736.4881832	736.4888	0.0006	0.8147	PC(31:3)	C39H72NO8PNa	[M+Na]+
736.4881832	736.4888	0.0006	0.8147	PE(34:3)	C39H72NO8PNa	[M+Na]+
736.4881832	736.4888	0.0006	0.8147	PE(O-34:4(OH))	C39H72NO8PNa	[M+Na]+
736.4881832	736.4888	0.0006	0.8147	PE(P-34:3(OH))	C39H72NO8PNa	[M+Na]+
737.5205418	737.5198	0.0007	0.9491	MGDG(33:4)	C42H72O10	[M+H]+
740.4133353	740.4133	0	0.0000	PS(32:6(OH))	C38H62NO11P	[M+H]+
742.46442	742.463	0.0015	2.0203	PS(31:1)	C37H70NO10PNa	[M+Na]+
742.46442	742.463	0.0015	2.0203	PS(P-31:1(OH))	C37H70NO10PNa	[M+Na]+
742.46442	742.4654	0.0009	1.2122	PS(33:3(OH))	C39H70NO11P	[M+H-H2O]+
742.46442	742.4654	0.0009	1.2122	PS(33:4)	C39H68NO10P	[M+H]+
742.4989746	742.4993	0.0004	0.5387	LPS(32:1)	C38H74NO9PNa	[M+Na]+
742.4989746	742.4993	0.0004	0.5387	PC(30:1(OH))	C38H74NO9PNa	[M+Na]+
742.4989746	742.4993	0.0004	0.5387	PE(33:1(OH))	C38H74NO9PNa	[M+Na]+
742.4989746	742.4993	0.0004	0.5387	PS(O-32:1)	C38H74NO9PNa	[M+Na]+
742.4989746	742.4993	0.0004	0.5387	PS(P-32:0)	C38H74NO9PNa	[M+Na]+
744.4940344	744.4939	0.0002	0.2686	PE(P-36:5)	C41H72NO7PNa	[M+Na]+
744.4940344	744.494	0	0.0000	PC(30:0)	C38H76NO8PK	[M+K]+
744.4940344	744.494	0	0.0000	PC(O-30:1(OH))	C38H76NO8PK	[M+K]+
744.4940344	744.494	0	0.0000	PC(P-30:0(OH))	C38H76NO8PK	[M+K]+
744.4940344	744.494	0	0.0000	PE(33:0)	C38H76NO8PK	[M+K]+
744.4940344	744.494	0	0.0000	PE(O-33:1(OH))	C38H76NO8PK	[M+K]+
744.4940344	744.494	0	0.0000	PE(P-33:0(OH))	C38H76NO8PK	[M+K]+
744.4940344	744.494	0	0.0000	PENMe(32:0)	C38H76NO8PK	[M+K]+
744.5148207	744.515	0.0002	0.2686	LPS(32:0)	C38H76NO9PNa	[M+Na]+
744.5148207	744.515	0.0002	0.2686	PC(30:0(OH))	C38H76NO9PNa	[M+Na]+
744.5148207	744.515	0.0002	0.2686	PE(33:0(OH))	C38H76NO9PNa	[M+Na]+
744.5148207	744.515	0.0002	0.2686	PS(O-32:0)	C38H76NO9PNa	[M+Na]+
745.4974257	745.4974	0	0.0000	PICer(t29:0)	C35H70NO12P	[M+NH4]+
746.4923664	746.4943	0.0019	2.5452	PS(O-31:0(OH))	C37H74NO10PNa	[M+Na]+
749.5092591	749.5092	0.0001	0.1334	PA(38:3)	C41H75O8PNa	[M+Na]+
749.5092591	749.5092	0.0001	0.1334	PA(O-38:4(OH))	C41H75O8PNa	[M+Na]+
749.5092591	749.5092	0.0001	0.1334	PA(P-38:3(OH))	C41H75O8PNa	[M+Na]+
749.5103176	749.5092	0.0011	1.4676	PA(38:3)	C41H75O8PNa	[M+Na]+

749.5103176	749.5092	0.0011	1.4676	PA(O-38:4(OH))	C41H75O8PNa	[M+Na]+
749.5103176	749.5092	0.0011	1.4676	PA(P-38:3(OH))	C41H75O8PNa	[M+Na]+
749.5103176	749.5116	0.0013	1.7345	PA(40:5(OH))	C43H75O9P	[M+H-H2O]+
749.5103176	749.5116	0.0013	1.7345	PA(40:6)	C43H73O8P	[M+H]+
749.5103176	749.5116	0.0013	1.7345	PA(P-40:6(OH))	C43H73O8P	[M+H]+
749.5103176	749.5117	0.0014	1.8679	TG(42:6)	C45H74O6K	[M+K]+
750.5127006	750.5151	0.0024	3.1978	MGDG(33:6)	C42H68O10	[M+NH4]+
751.5158204	751.5151	0.0007	0.9315	PECer(d38:3)	C40H77N2O6PK	[M+K]+
754.5358589	754.5357	0.0001	0.1325	PC(32:1)	C40H78NO8PNa	[M+Na]+
754.5358589	754.5357	0.0001	0.1325	PC(P-32:1(OH))	C40H78NO8PNa	[M+Na]+
754.5358589	754.5357	0.0001	0.1325	PE(35:1)	C40H78NO8PNa	[M+Na]+
754.5358589	754.5357	0.0001	0.1325	PE(O-35:2(OH))	C40H78NO8PNa	[M+Na]+
754.5358589	754.5357	0.0001	0.1325	PE(P-35:1(OH))	C40H78NO8PNa	[M+Na]+
754.5358589	754.5357	0.0001	0.1325	PENMe(34:1)	C40H78NO8PNa	[M+Na]+
757.5456052	757.549	0.0034	4.4882	PC(33:4)	C41H74NO8P	[M+NH4]+
757.5456052	757.549	0.0034	4.4882	PE(36:4)	C41H74NO8P	[M+NH4]+
757.5456052	757.549	0.0034	4.4882	PE(O-36:5(OH))	C41H74NO8P	[M+NH4]+
757.5456052	757.549	0.0034	4.4882	PE(P-36:4(OH))	C41H74NO8P	[M+NH4]+
760.5094525	760.5099	0.0004	0.5260	PS(O-32:0(OH))	C38H76NO10PNa	[M+Na]+
760.510625	760.5099	0.0007	0.9204	PS(O-32:0(OH))	C38H76NO10PNa	[M+Na]+
761.5128851	761.5116	0.0013	1.7071	PA(41:6(OH))	C44H75O9P	[M+H-H2O]+
761.5128851	761.5116	0.0013	1.7071	PA(41:7)	C44H73O8P	[M+H]+
761.5128851	761.5116	0.0013	1.7071	PGP-38:6)	C44H75O9P	[M+H-H2O]+
768.4447609	768.4446	0.0001	0.1301	PS(34:6(OH))	C40H66NO11P	[M+H]+
769.4481788	769.4498	0.0016	2.0794	PI(28:1(OH))	C37H69O14P	[M+H]+
770.5309224	770.5306	0.0003	0.3893	PC(32:1(OH))	C40H78NO9PNa	[M+Na]+
770.5309224	770.5306	0.0003	0.3893	PE(35:1(OH))	C40H78NO9PNa	[M+Na]+
770.5309224	770.5306	0.0003	0.3893	PS(O-34:1)	C40H78NO9PNa	[M+Na]+
770.5309224	770.5306	0.0003	0.3893	PS(P-34:0)	C40H78NO9PNa	[M+Na]+
772.5465854	772.5463	0.0003	0.3883	LPS(34:0)	C40H80NO9PNa	[M+Na]+
772.5465854	772.5463	0.0003	0.3883	PC(32:0(OH))	C40H80NO9PNa	[M+Na]+
772.5465854	772.5463	0.0003	0.3883	PE(35:0(OH))	C40H80NO9PNa	[M+Na]+
772.5465854	772.5463	0.0003	0.3883	PS(O-34:0)	C40H80NO9PNa	[M+Na]+
777.5391026	777.5389	0.0002	0.2572	PS(34:2)	C40H74NO10P	[M+NH4]+
777.5391026	777.5389	0.0002	0.2572	PS(O-34:3(OH))	C40H74NO10P	[M+NH4]+
777.5391026	777.5389	0.0002	0.2572	PS(P-34:2(OH))	C40H74NO10P	[M+NH4]+
777.5408448	777.5405	0.0004	0.5144	PA(40:3)	C43H79O8PNa	[M+Na]+
777.5408448	777.5405	0.0004	0.5144	PA(O-40:4(OH))	C43H79O8PNa	[M+Na]+

777.5408448	777.5405	0.0004	0.5144	PA(P-40:3(OH))	C43H79O8PNa	[M+Na]+
778.5447581	778.5464	0.0016	2.0551	MGDG(35:6)	C44H72O10	[M+NH4]+
779.4471009	779.447	0.0001	0.1283	PG34:5(OH))	C40H69O11PNa	[M+Na]+
779.4471009	779.4471	0	0.0000	LPI(28:0)	C37H73O12PK	[M+K]+
779.4471009	779.4471	0	0.0000	PI(O-28:0)	C37H73O12PK	[M+K]+
786.6008673	786.6007	0.0001	0.1271	PA(41:3)	C44H81O8P	[M+NH4]+
786.6008673	786.6007	0.0001	0.1271	PC(36:1(OH))	C44H86NO9P	[M+H-H2O]+
786.6008673	786.6007	0.0001	0.1271	PC(36:2)	C44H84NO8P	[M+H]+
786.6008673	786.6007	0.0001	0.1271	PC(O-36:3(OH))	C44H84NO8P	[M+H]+
786.6008673	786.6007	0.0001	0.1271	PC(P-36:2(OH))	C44H84NO8P	[M+H]+
786.6008673	786.6007	0.0001	0.1271	PE(39:1(OH))	C44H86NO9P	[M+H-H2O]+
786.6008673	786.6007	0.0001	0.1271	PE(39:2)	C44H84NO8P	[M+H]+
786.6008673	786.6007	0.0001	0.1271	PS(O-38:1)	C44H86NO9P	[M+H-H2O]+
786.6008673	786.6007	0.0001	0.1271	PS(P-38:0)	C44H86NO9P	[M+H-H2O]+
789.6096707	789.6116	0.002	2.5329	PC(35:2)	C43H82NO8P	[M+NH4]+
789.6096707	789.6116	0.002	2.5329	PC(P-35:2(OH))	C43H82NO8P	[M+NH4]+
789.6096707	789.6116	0.002	2.5329	PE(38:2)	C43H82NO8P	[M+NH4]+
789.6096707	789.6116	0.002	2.5329	PE(O-38:3(OH))	C43H82NO8P	[M+NH4]+
789.6096707	789.6116	0.002	2.5329	PE(P-38:2(OH))	C43H82NO8P	[M+NH4]+
789.6096707	789.6116	0.002	2.5329	PENMe2(36:2)	C43H82NO8P	[M+NH4]+
790.6131073	790.6109	0.0022	2.7827	PE(O-42:5)	C47H86NO7P	[M+H-H2O]+
790.6131073	790.6109	0.0022	2.7827	PE(P-42:4)	C47H86NO7P	[M+H-H2O]+
791.6270193	791.6273	0.0003	0.3790	PC(35:1)	C43H84NO8P	[M+NH4]+
791.6270193	791.6273	0.0003	0.3790	PC(O-35:2(OH))	C43H84NO8P	[M+NH4]+
791.6270193	791.6273	0.0003	0.3790	PC(P-35:1(OH))	C43H84NO8P	[M+NH4]+
791.6270193	791.6273	0.0003	0.3790	PE(38:1)	C43H84NO8P	[M+NH4]+
791.6270193	791.6273	0.0003	0.3790	PE(O-38:2(OH))	C43H84NO8P	[M+NH4]+
791.6270193	791.6273	0.0003	0.3790	PE(P-38:1(OH))	C43H84NO8P	[M+NH4]+
792.5127862	792.515	0.0022	2.7760	PC(34:4(OH))	C42H76NO9PNa	[M+Na]+
792.5127862	792.515	0.0022	2.7760	PE(37:4(OH))	C42H76NO9PNa	[M+Na]+
792.5127862	792.515	0.0022	2.7760	PS(O-36:4)	C42H76NO9PNa	[M+Na]+
792.5127862	792.515	0.0022	2.7760	PS(P-36:3)	C42H76NO9PNa	[M+Na]+
792.6308042	792.6324	0.0016	2.0186	HexCer(d39:1)	C45H87NO8Na	[M+Na]+
793.4627791	793.4626	0.0002	0.2521	PG35:5(OH))	C41H71O11PNa	[M+Na]+
793.4627791	793.4628	0	0.0000	LPI(29:0)	C38H75O12PK	[M+K]+
793.4627791	793.4628	0	0.0000	PI(O-29:0)	C38H75O12PK	[M+K]+
793.5207399	793.5225	0.0018	2.2684	PI(32:0)	C41H79O13P	[M+H-H2O]+
793.5207399	793.5225	0.0018	2.2684	PI(O-32:1(OH))	C41H79O13P	[M+H-H2O]+

793.5207399	793.5225	0.0018	2.2684	PI(P-32:0(OH))	C41H79O13P	[M+H-H2O]+
793.5207399	793.5225	0.0018	2.2684	PI(P-32:1)	C41H77O12P	[M+H]+
793.5207399	793.5227	0.0019	2.3944	MGDG(34:2)	C43H78O10K	[M+K]+
795.5354106	795.5382	0.0028	3.5196	LPI(32:1)	C41H79O12P	[M+H]+
795.5354106	795.5382	0.0028	3.5196	PI(O-32:0(OH))	C41H81O13P	[M+H-H2O]+
795.5354106	795.5382	0.0028	3.5196	PI(O-32:1)	C41H79O12P	[M+H]+
795.5354106	795.5382	0.0028	3.5196	PI(P-32:0)	C41H79O12P	[M+H]+
795.5354106	795.5383	0.0029	3.6453	MGDG(34:1)	C43H80O10K	[M+K]+
801.4290831	801.4313	0.0022	2.7451	PG36:8(OH))	C42H67O11PNa	[M+Na]+
801.4290831	801.4314	0.0023	2.8699	LPIP(28:1)	C37H72O15P2	[M+H-H2O]+
801.4290831	801.4314	0.0023	2.8699	PIP(P-28:0)	C37H72O15P2	[M+H-H2O]+
801.4290831	801.4315	0.0024	2.9946	LPI(30:3)	C39H71O12PK	[M+K]+
801.4311894	801.4313	0.0001	0.1248	PG36:8(OH))	C42H67O11PNa	[M+Na]+
801.4311894	801.4314	0.0002	0.2496	LPIP(28:1)	C37H72O15P2	[M+H-H2O]+
801.4311894	801.4314	0.0002	0.2496	PIP(P-28:0)	C37H72O15P2	[M+H-H2O]+
801.4311894	801.4315	0.0003	0.3743	LPI(30:3)	C39H71O12PK	[M+K]+
802.5957005	802.5956	0.0001	0.1246	PA(41:3(OH))	C44H81O9P	[M+NH4]+
802.5957005	802.5956	0.0001	0.1246	PC(36:2(OH))	C44H84NO9P	[M+H]+
802.5957005	802.5956	0.0001	0.1246	PE(39:2(OH))	C44H84NO9P	[M+H]+
802.5957005	802.5956	0.0001	0.1246	PGO-38:4)	C44H81O9P	[M+NH4]+
802.5957005	802.5956	0.0001	0.1246	PGP-38:3)	C44H81O9P	[M+NH4]+
802.5957005	802.5956	0.0001	0.1246	PS(38:0)	C44H86NO10P	[M+H-H2O]+
802.5957005	802.5956	0.0001	0.1246	PS(O-38:1(OH))	C44H86NO10P	[M+H-H2O]+
802.5957005	802.5956	0.0001	0.1246	PS(O-38:2)	C44H84NO9P	[M+H]+
802.5957005	802.5956	0.0001	0.1246	PS(P-38:0(OH))	C44H86NO10P	[M+H-H2O]+
802.5957005	802.5956	0.0001	0.1246	PS(P-38:1)	C44H84NO9P	[M+H]+
804.6116727	804.6113	0.0004	0.4971	PA(41:2(OH))	C44H83O9P	[M+NH4]+
804.6116727	804.6113	0.0004	0.4971	PC(36:1(OH))	C44H86NO9P	[M+H]+
804.6116727	804.6113	0.0004	0.4971	PE(39:1(OH))	C44H86NO9P	[M+H]+
804.6116727	804.6113	0.0004	0.4971	PGO-38:3)	C44H83O9P	[M+NH4]+
804.6116727	804.6113	0.0004	0.4971	PGP-38:2)	C44H83O9P	[M+NH4]+
804.6116727	804.6113	0.0004	0.4971	PS(O-38:0(OH))	C44H88NO10P	[M+H-H2O]+
804.6116727	804.6113	0.0004	0.4971	PS(O-38:1)	C44H86NO9P	[M+H]+
804.6116727	804.6113	0.0004	0.4971	PS(P-38:0)	C44H86NO9P	[M+H]+
805.4628583	805.4626	0.0002	0.2483	PG36:6(OH))	C42H71O11PNa	[M+Na]+
805.4628583	805.4628	0.0001	0.1242	LPI(30:1)	C39H75O12PK	[M+K]+
805.4628583	805.4628	0.0001	0.1242	PI(O-30:1)	C39H75O12PK	[M+K]+
805.4628583	805.4628	0.0001	0.1242	PI(P-30:0)	C39H75O12PK	[M+K]+

805.48407	805.4837	0.0003	0.3724	PI(30:0)	C39H75O13PNa	[M+Na]+
805.48407	805.4837	0.0003	0.3724	PI(O-30:1(OH))	C39H75O13PNa	[M+Na]+
805.48407	805.4837	0.0003	0.3724	PI(P-30:0(OH))	C39H75O13PNa	[M+Na]+
806.466361	806.4663	0.0001	0.1240	LacCer(t26:0)	C38H73NO14K	[M+K]+
808.5829918	808.5827	0.0003	0.3710	PC(36:2)	C44H84NO8PNa	[M+Na]+
808.5829918	808.5827	0.0003	0.3710	PC(O-36:3(OH))	C44H84NO8PNa	[M+Na]+
808.5829918	808.5827	0.0003	0.3710	PC(P-36:2(OH))	C44H84NO8PNa	[M+Na]+
808.5829918	808.5827	0.0003	0.3710	PE(39:2)	C44H84NO8PNa	[M+Na]+
809.586374	809.5902	0.0038	4.6937	PG37:0(OH))	C43H85O11P	[M+H]+
809.6390142	809.6378	0.0012	1.4821	PC(35:0(OH))	C43H86NO9P	[M+NH4]+
809.6390142	809.6378	0.0012	1.4821	PE(38:0(OH))	C43H86NO9P	[M+NH4]+
809.6390142	809.6378	0.0012	1.4821	PS(O-37:0)	C43H86NO9P	[M+NH4]+
809.6390142	809.6395	0.0004	0.4940	PA(O-43:1)	C46H91O7PNa	[M+Na]+
809.6390142	809.6395	0.0004	0.4940	PA(P-43:0)	C46H91O7PNa	[M+Na]+
810.5893447	810.5855	0.0039	4.8113	PG36:1(OH))	C42H81O11P	[M+NH4]+
810.5893447	810.5855	0.0039	4.8113	PICer(d36:0)	C42H84NO11P	[M+H]+
810.5893447	810.5856	0.0038	4.6880	HexCer(t38:1)	C44H85NO9K	[M+K]+
811.5922486	811.589	0.0033	4.0661	LacCer(d29:0)	C41H79NO13	[M+NH4]+
811.5922486	811.596	0.0037	4.5589	PC(37:5)	C45H80NO8P	[M+NH4]+
811.5922486	811.596	0.0037	4.5589	PE(40:5)	C45H80NO8P	[M+NH4]+
811.5922486	811.596	0.0037	4.5589	PE(O-40:6(OH))	C45H80NO8P	[M+NH4]+
811.5922486	811.596	0.0037	4.5589	PE(P-40:5(OH))	C45H80NO8P	[M+NH4]+
812.5075106	812.5072	0.0003	0.3692	PG37:7(OH))	C43H71O11P	[M+NH4]+
812.5075106	812.5072	0.0003	0.3692	PS(37:5(OH))	C43H74NO11P	[M+H]+
813.5114417	813.5124	0.0009	1.1063	PI(31:0(OH))	C40H77O14P	[M+H]+
814.5231233	814.5229	0.0003	0.3683	PG37:6(OH))	C43H73O11P	[M+NH4]+
814.5231233	814.5229	0.0003	0.3683	PS(37:4(OH))	C43H76NO11P	[M+H]+
816.5292329	816.5304	0.0012	1.4696	PE(O-40:6)	C45H80NO7PK	[M+K]+
816.5292329	816.5304	0.0012	1.4696	PE(P-40:5)	C45H80NO7PK	[M+K]+
816.5306084	816.5304	0.0002	0.2449	PE(O-40:6)	C45H80NO7PK	[M+K]+
816.5306084	816.5304	0.0002	0.2449	PE(P-40:5)	C45H80NO7PK	[M+K]+
819.5354856	819.5358	0.0003	0.3661	LPI(32:0)	C41H81O12PNa	[M+Na]+
819.5354856	819.5358	0.0003	0.3661	PI(O-32:0)	C41H81O12PNa	[M+Na]+
822.49184	822.4916	0.0003	0.3647	PG38:9(OH))	C44H69O11P	[M+NH4]+
822.49184	822.4916	0.0003	0.3647	PS(38:7(OH))	C44H72NO11P	[M+H]+
824.5777057	824.5776	0.0001	0.1213	PC(36:2(OH))	C44H84NO9PNa	[M+Na]+
824.5777057	824.5776	0.0001	0.1213	PE(39:2(OH))	C44H84NO9PNa	[M+Na]+
824.5777057	824.5776	0.0001	0.1213	PS(O-38:2)	C44H84NO9PNa	[M+Na]+

824.5777057	824.5776	0.0001	0.1213	PS(P-38:1)	C44H84NO9PNa	[M+Na]+
825.5815842	825.5793	0.0023	2.7859	PA(47:8)	C50H83O8P	[M+H-H2O]+
826.4974958	826.4993	0.0018	2.1779	PE(40:8(OH))	C45H74NO9PNa	[M+Na]+
826.4974958	826.4995	0.002	2.4198	PS(36:2)	C42H78NO10PK	[M+K]+
826.4974958	826.4995	0.002	2.4198	PS(O-36:3(OH))	C42H78NO10PK	[M+K]+
826.4974958	826.4995	0.002	2.4198	PS(P-36:2(OH))	C42H78NO10PK	[M+K]+
826.5232744	826.5229	0.0004	0.4840	PG38:7(OH))	C44H73O11P	[M+NH4]+
826.5232744	826.5229	0.0004	0.4840	PS(38:5(OH))	C44H76NO11P	[M+H]+
826.5937053	826.5932	0.0005	0.6049	PC(36:1(OH))	C44H86NO9PNa	[M+Na]+
826.5937053	826.5932	0.0005	0.6049	PE(39:1(OH))	C44H86NO9PNa	[M+Na]+
826.5937053	826.5932	0.0005	0.6049	PS(O-38:1)	C44H86NO9PNa	[M+Na]+
826.5937053	826.5932	0.0005	0.6049	PS(P-38:0)	C44H86NO9PNa	[M+Na]+
827.4448625	827.447	0.0021	2.5379	LPIP(30:2)	C39H74O15P2	[M+H-H2O]+
827.4448625	827.447	0.0021	2.5379	PG38:9(OH))	C44H69O11PNa	[M+Na]+
827.4448625	827.447	0.0021	2.5379	PIP(P-30:1)	C39H74O15P2	[M+H-H2O]+
827.4448625	827.4471	0.0023	2.7796	LPI(32:4)	C41H73O12PK	[M+K]+
827.4660601	827.4681	0.002	2.4170	PI(32:3)	C41H73O13PNa	[M+Na]+
827.5970862	827.5949	0.0022	2.6583	PA(47:7)	C50H85O8P	[M+H-H2O]+
827.5970862	827.595	0.0021	2.5375	DG(49:9)	C52H84O5K	[M+K]+
828.5387851	828.5385	0.0003	0.3621	PG38:6(OH))	C44H75O11P	[M+NH4]+
828.5387851	828.5385	0.0003	0.3621	PS(38:4(OH))	C44H78NO11P	[M+H]+
828.541127	828.5385	0.0026	3.1381	PG38:6(OH))	C44H75O11P	[M+NH4]+
828.541127	828.5385	0.0026	3.1381	PS(38:4(OH))	C44H78NO11P	[M+H]+
828.541127	828.5444	0.0032	3.8622	LacCer(d30:1)	C42H79NO13Na	[M+Na]+
831.4784808	831.4783	0.0002	0.2405	LPIP(30:0)	C39H78O15P2	[M+H-H2O]+
831.4784808	831.4783	0.0002	0.2405	PG38:7(OH))	C44H73O11PNa	[M+Na]+
831.4784808	831.4783	0.0002	0.2405	PIP(O-30:0)	C39H78O15P2	[M+H-H2O]+
831.4784808	831.4784	0.0001	0.1203	PI(P-32:1)	C41H77O12PK	[M+K]+
832.4824118	832.4818	0.0006	0.7207	MIPC(d26:0)	C38H74NO16P	[M+H]+
832.4824118	832.4819	0.0005	0.6006	LacCer(t28:1)	C40H75NO14K	[M+K]+
836.5051383	836.5048	0.0003	0.3586	PS(37:4(OH))	C43H76NO11PNa	[M+Na]+
836.5051383	836.505	0.0002	0.2391	PICer(t34:0)	C40H80NO12PK	[M+K]+
836.5389675	836.5366	0.0024	2.8690	DGDG(27:2)	C42H74O15	[M+NH4]+
836.5389675	836.5412	0.0022	2.6299	PS(38:3)	C44H80NO10PNa	[M+Na]+
836.5389675	836.5412	0.0022	2.6299	PS(O-38:4(OH))	C44H80NO10PNa	[M+Na]+
836.5389675	836.5412	0.0022	2.6299	PS(P-38:3(OH))	C44H80NO10PNa	[M+Na]+
836.541068	836.5412	0.0001	0.1195	PS(38:3)	C44H80NO10PNa	[M+Na]+
836.541068	836.5412	0.0001	0.1195	PS(O-38:4(OH))	C44H80NO10PNa	[M+Na]+

836.541068	836.5412	0.0001	0.1195	PS(P-38:3(OH))	C44H80NO10PNa	[M+Na]+
837.5080433	837.5041	0.004	4.7761	PA(44:9(OH))	C47H75O9PNa	[M+Na]+
837.5080433	837.5042	0.0038	4.5373	PG38:4)	C44H79O10PK	[M+K]+
837.5080433	837.5042	0.0038	4.5373	PGO-38:5(OH))	C44H79O10PK	[M+K]+
837.5080433	837.5042	0.0038	4.5373	PGP-38:4(OH))	C44H79O10PK	[M+K]+
838.61866	838.6168	0.0019	2.2656	PG38:1(OH))	C44H85O11P	[M+NH4]+
838.61866	838.6168	0.0019	2.2656	PICer(d38:0)	C44H88NO11P	[M+H]+
838.61866	838.6169	0.0018	2.1464	HexCer(t40:1)	C46H89NO9K	[M+K]+
840.5371786	840.5361	0.0011	1.3087	PS(37:2(OH))	C43H80NO11PNa	[M+Na]+
840.5371786	840.5385	0.0013	1.5466	PG39:7(OH))	C45H75O11P	[M+NH4]+
840.5371786	840.5385	0.0013	1.5466	PS(39:5(OH))	C45H78NO11P	[M+H]+
840.5695065	840.5679	0.0016	1.9035	DGDG(27:0)	C42H78O15	[M+NH4]+
840.5717786	840.5725	0.0007	0.8328	PS(38:1)	C44H84NO10PNa	[M+Na]+
840.5717786	840.5725	0.0007	0.8328	PS(O-38:2(OH))	C44H84NO10PNa	[M+Na]+
840.5717786	840.5725	0.0007	0.8328	PS(P-38:1(OH))	C44H84NO10PNa	[M+Na]+
841.5397265	841.5378	0.0019	2.2578	PA(46:10(OH))	C49H77O9P	[M+H]+
841.5408167	841.5378	0.003	3.5649	PA(46:10(OH))	C49H77O9P	[M+H]+
841.5408167	841.5437	0.0029	3.4460	PI(33:0(OH))	C42H81O14P	[M+H]+
841.575112	841.5742	0.0009	1.0694	PA(47:8(OH))	C50H83O9P	[M+H-H2O]+
842.5527839	842.5518	0.001	1.1869	PICer(d37:2)	C43H82NO11PNa	[M+Na]+
842.5527839	842.5518	0.001	1.1869	PS(37:1(OH))	C43H82NO11PNa	[M+Na]+
842.5527839	842.5542	0.0014	1.6616	PG39:6(OH))	C45H77O11P	[M+NH4]+
842.5527839	842.5542	0.0014	1.6616	PS(39:4(OH))	C45H80NO11P	[M+H]+
843.4381071	843.4395	0.0014	1.6599	LPIP(28:0)	C37H74O15P2Na	[M+Na]+
843.4381071	843.4395	0.0014	1.6599	PIP(O-28:0)	C37H74O15P2Na	[M+Na]+
843.5561731	843.5534	0.0027	3.2007	PA(46:9(OH))	C49H79O9P	[M+H]+
843.5561731	843.5534	0.0027	3.2007	PG43:8)	C49H81O10P	[M+H-H2O]+
844.5592747	844.5579	0.0014	1.6577	SHexCer(d37:1)	C43H83NO11SNa	[M+Na]+
844.5602527	844.5617	0.0014	1.6577	PE(O-42:6)	C47H84NO7PK	[M+K]+
844.5602527	844.5617	0.0014	1.6577	PE(P-42:5)	C47H84NO7PK	[M+K]+
845.4536832	845.4504	0.0033	3.9032	SQDG(37:10)	C46H68O12S	[M+H]+
845.4536832	845.4576	0.0039	4.6129	LPIP(30:2)	C39H74O15P2	[M+H]+
845.4536832	845.4576	0.0039	4.6129	PIP(30:0)	C39H76O16P2	[M+H-H2O]+
845.4536832	845.4576	0.0039	4.6129	PIP(O-30:1(OH))	C39H76O16P2	[M+H-H2O]+
845.4536832	845.4576	0.0039	4.6129	PIP(P-30:0(OH))	C39H76O16P2	[M+H-H2O]+
845.4536832	845.4576	0.0039	4.6129	PIP(P-30:1)	C39H74O15P2	[M+H]+
845.4536832	845.4577	0.004	4.7312	PI(32:2)	C41H75O13PK	[M+K]+
845.562163	845.5621	0.0001	0.1183	DGDG(30:1)	C45H82O15	[M+H-H2O]+

846.4565253	846.4528	0.0037	4.3712	LPIP(29:3)	C38H70O15P2	[M+NH4]+
846.5239137	846.5256	0.0017	2.0082	PGP(34:1)	C40H78O13P2	[M+NH4]+
846.5239137	846.5256	0.0016	1.8901	PS(39:5)	C45H78NO10PNa	[M+Na]+
846.5239137	846.5257	0.0018	2.1263	PICer(d36:1)	C42H82NO11PK	[M+K]+
846.5239137	846.5257	0.0018	2.1263	PS(36:0(OH))	C42H82NO11PK	[M+K]+
846.5636175	846.5621	0.0015	1.7719	PS(O-37:0(OH))	C43H86NO10PK	[M+K]+
846.5636175	846.5643	0.0007	0.8269	PC(40:8(OH))	C48H80NO9P	[M+H]+
846.5636175	846.5643	0.0007	0.8269	PE(43:8(OH))	C48H80NO9P	[M+H]+
846.5636175	846.5643	0.0007	0.8269	PS(42:6)	C48H82NO10P	[M+H-H2O]+
846.5636175	846.5643	0.0007	0.8269	PS(P-42:6(OH))	C48H82NO10P	[M+H-H2O]+
850.5189097	850.5205	0.0016	1.8812	PS(38:4(OH))	C44H78NO11PNa	[M+Na]+
850.5189097	850.5206	0.0017	1.9988	PICer(t35:0)	C41H82NO12PK	[M+K]+
850.5212228	850.5205	0.0008	0.9406	PS(38:4(OH))	C44H78NO11PNa	[M+Na]+
850.5212228	850.5206	0.0006	0.7055	PICer(t35:0)	C41H82NO12PK	[M+K]+
850.5212228	850.5229	0.0016	1.8812	PG40:9(OH))	C46H73O11P	[M+NH4]+
850.5212228	850.5229	0.0016	1.8812	PS(40:7(OH))	C46H76NO11P	[M+H]+
852.5738345	852.5725	0.0013	1.5248	PS(39:2)	C45H84NO10PNa	[M+Na]+
852.5738345	852.5749	0.0011	1.2902	PG41:7)	C47H79O10P	[M+NH4]+
852.5738345	852.5749	0.0011	1.2902	PS(41:4(OH))	C47H84NO11P	[M+H-H2O]+
852.5738345	852.5749	0.0011	1.2902	PS(41:5)	C47H82NO10P	[M+H]+
853.5769844	853.5742	0.0028	3.2803	PA(48:10)	C51H81O8P	[M+H]+
853.5769844	853.5742	0.0028	3.2803	PA(48:9(OH))	C51H83O9P	[M+H-H2O]+
853.5769844	853.5743	0.0027	3.1632	TG(50:10)	C53H82O6K	[M+K]+
853.5769844	853.58	0.003	3.5146	MGDG(40:6)	C49H82O10Na	[M+Na]+
853.5769844	853.5801	0.0031	3.6318	PI(35:0)	C44H85O13P	[M+H]+
853.5769844	853.5801	0.0031	3.6318	PI(O-35:1(OH))	C44H85O13P	[M+H]+
853.5769844	853.5801	0.0031	3.6318	PI(P-35:0(OH))	C44H85O13P	[M+H]+
858.5477971	858.5491	0.0013	1.5142	PI(P-36:5)	C45H77O12P	[M+NH4]+
859.529187	859.5291	0.0001	0.1163	MIPC(m28:1)	C40H76NO15P	[M+NH4]+
859.5303227	859.5307	0.0004	0.4654	PI(34:1)	C43H81O13PNa	[M+Na]+
859.5303227	859.5307	0.0004	0.4654	PI(O-34:2(OH))	C43H81O13PNa	[M+Na]+
859.5303227	859.5307	0.0004	0.4654	PI(P-34:1(OH))	C43H81O13PNa	[M+Na]+
860.532693	860.5318	0.0009	1.0459	SHexCer(d37:1)	C43H83NO11SK	[M+K]+
861.5133212	861.5124	0.001	1.1607	PI(35:4(OH))	C44H77O14P	[M+H]+
863.5268128	863.524	0.0028	3.2425	MIPC(d27:0)	C39H76NO16P	[M+NH4]+
863.5268128	863.5256	0.0012	1.3897	PI(33:0(OH))	C42H81O14PNa	[M+Na]+
863.5268128	863.528	0.0012	1.3896	PI(35:3(OH))	C44H79O14P	[M+H]+
864.5349008	864.5361	0.0012	1.3880	PS(39:4(OH))	C45H80NO11PNa	[M+Na]+

864.5349008	864.5363	0.0014	1.6194	PICer(t36:0)	C42H84NO12PK	[M+K]+
865.5382601	865.5378	0.0005	0.5777	PA(48:12(OH))	C51H77O9P	[M+H]+
865.5406449	865.5378	0.0029	3.3505	PA(48:12(OH))	C51H77O9P	[M+H]+
865.5406449	865.5437	0.003	3.4660	PI(35:2(OH))	C44H81O14P	[M+H]+
866.5413178	866.5389	0.0024	2.7696	MIPC(m31:1)	C43H82NO15P	[M+H-H2O]+
866.5413178	866.5389	0.0024	2.7696	PI(34:3(OH))	C43H77O14P	[M+NH4]+
866.6592345	866.6609	0.0017	1.9616	PC(40:1)	C48H94NO8PNa	[M+Na]+
866.6592345	866.6609	0.0017	1.9616	PC(O-40:2(OH))	C48H94NO8PNa	[M+Na]+
866.6592345	866.6609	0.0017	1.9616	PC(P-40:1(OH))	C48H94NO8PNa	[M+Na]+
866.6592345	866.6609	0.0017	1.9616	PE(43:1)	C48H94NO8PNa	[M+Na]+
866.6592345	866.6609	0.0017	1.9616	PE(P-43:1(OH))	C48H94NO8PNa	[M+Na]+
866.6605156	866.6609	0.0004	0.4615	PC(40:1)	C48H94NO8PNa	[M+Na]+
866.6605156	866.6609	0.0004	0.4615	PC(O-40:2(OH))	C48H94NO8PNa	[M+Na]+
866.6605156	866.6609	0.0004	0.4615	PC(P-40:1(OH))	C48H94NO8PNa	[M+Na]+
866.6605156	866.6609	0.0004	0.4615	PE(43:1)	C48H94NO8PNa	[M+Na]+
866.6605156	866.6609	0.0004	0.4615	PE(P-43:1(OH))	C48H94NO8PNa	[M+Na]+
867.6633266	867.6603	0.003	3.4576	PA(O-46:1)	C49H97O7PK	[M+K]+
867.6633266	867.6603	0.003	3.4576	PA(P-46:0)	C49H97O7PK	[M+K]+
868.5061498	868.5053	0.0009	1.0363	DGDG(30:7)	C45H70O15	[M+NH4]+
868.5684404	868.5674	0.001	1.1513	PS(39:2(OH))	C45H84NO11PNa	[M+Na]+
868.5684404	868.5698	0.0014	1.6118	PG41:7(OH))	C47H79O11P	[M+NH4]+
868.5684404	868.5698	0.0014	1.6118	PS(41:5(OH))	C47H82NO11P	[M+H]+
868.6763153	868.6766	0.0003	0.3454	PC(40:0)	C48H96NO8PNa	[M+Na]+
868.6763153	868.6766	0.0003	0.3454	PC(O-40:1(OH))	C48H96NO8PNa	[M+Na]+
868.6763153	868.6766	0.0003	0.3454	PC(P-40:0(OH))	C48H96NO8PNa	[M+Na]+
868.6763153	868.6766	0.0003	0.3454	PE(43:0)	C48H96NO8PNa	[M+Na]+
868.6763153	868.6766	0.0003	0.3454	PE(O-43:1(OH))	C48H96NO8PNa	[M+Na]+
868.6763153	868.6766	0.0003	0.3454	PE(P-43:0(OH))	C48H96NO8PNa	[M+Na]+
869.5718292	869.5691	0.0027	3.1050	PA(48:10(OH))	C51H81O9P	[M+H]+
869.5718292	869.575	0.0031	3.5650	PI(35:0(OH))	C44H85O14P	[M+H]+
870.5744607	870.5736	0.0009	1.0338	SHexCer(d39:2)	C45H85NO11SNa	[M+Na]+
870.6931595	870.6922	0.0009	1.0337	PC(O-40:0(OH))	C48H98NO8PNa	[M+Na]+
870.6931595	870.6922	0.0009	1.0337	PE(O-43:0(OH))	C48H98NO8PNa	[M+Na]+
870.6931595	870.6946	0.0015	1.7228	PA(47:3)	C50H93O8P	[M+NH4]+
870.6931595	870.6946	0.0015	1.7228	PA(P-47:3(OH))	C50H93O8P	[M+NH4]+
870.6931595	870.6946	0.0015	1.7228	PC(42:1(OH))	C50H98NO9P	[M+H-H2O]+
870.6931595	870.6946	0.0015	1.7228	PC(42:2)	C50H96NO8P	[M+H]+
870.6931595	870.6946	0.0015	1.7228	PC(O-42:3(OH))	C50H96NO8P	[M+H]+

870.6931595	870.6946	0.0015	1.7228	PC(P-42:2(OH))	C50H96NO8P	[M+H]+
870.6931595	870.6946	0.0015	1.7228	PE(45:1(OH))	C50H98NO9P	[M+H-H2O]+
870.6931595	870.6946	0.0015	1.7228	PE(45:2)	C50H96NO8P	[M+H]+
870.6931595	870.6946	0.0015	1.7228	PE(O-45:3(OH))	C50H96NO8P	[M+H]+
870.6931595	870.6946	0.0015	1.7228	PE(P-45:2(OH))	C50H96NO8P	[M+H]+
870.6931595	870.6946	0.0015	1.7228	PS(O-44:1)	C50H98NO9P	[M+H-H2O]+
870.6931595	870.6946	0.0015	1.7228	PS(P-44:0)	C50H98NO9P	[M+H-H2O]+
871.5767584	871.5777	0.001	1.1473	DGDG(32:2)	C47H84O15	[M+H-H2O]+
871.6883553	871.6899	0.0015	1.7208	PC(41:3)	C49H92NO8P	[M+NH4]+
871.6883553	871.6899	0.0015	1.7208	PE(44:3)	C49H92NO8P	[M+NH4]+
871.6883553	871.6899	0.0015	1.7208	PE(O-44:4(OH))	C49H92NO8P	[M+NH4]+
871.6883553	871.6899	0.0015	1.7208	PE(P-44:3(OH))	C49H92NO8P	[M+NH4]+
872.5387352	872.5366	0.0021	2.4068	DGDG(30:5)	C45H74O15	[M+NH4]+
872.5387352	872.5412	0.0025	2.8652	PS(41:6)	C47H80NO10PNa	[M+Na]+
872.5387352	872.5414	0.0026	2.9798	PICer(d38:2)	C44H84NO11PK	[M+K]+
872.5387352	872.5414	0.0026	2.9798	PS(38:1(OH))	C44H84NO11PK	[M+K]+
872.6918374	872.6891	0.0027	3.0939	PE(O-48:6)	C53H96NO7P	[M+H-H2O]+
872.6918374	872.6891	0.0027	3.0939	PE(P-48:5)	C53H96NO7P	[M+H-H2O]+
873.5236595	873.5252	0.0016	1.8317	PG41:7(OH))	C47H79O11PNa	[M+Na]+
873.5236595	873.5253	0.0016	1.8317	LPIP(33:0)	C42H84O15P2	[M+H-H2O]+
873.5236595	873.5253	0.0016	1.8317	PIP(O-33:0)	C42H84O15P2	[M+H-H2O]+
873.5236595	873.5254	0.0017	1.9461	PI(O-35:2)	C44H83O12PK	[M+K]+
873.5236595	873.5254	0.0017	1.9461	PI(P-35:1)	C44H83O12PK	[M+K]+
873.6943464	873.6943	0.0001	0.1145	PA(47:1(OH))	C50H97O9P	[M+H]+
873.6943464	873.6943	0.0001	0.1145	PG44:0)	C50H99O10P	[M+H-H2O]+
873.6943464	873.6943	0.0001	0.1145	PGO-44:1(OH))	C50H99O10P	[M+H-H2O]+
873.6943464	873.6943	0.0001	0.1145	PGO-44:2)	C50H97O9P	[M+H]+
873.6943464	873.6943	0.0001	0.1145	PGP-44:0(OH))	C50H99O10P	[M+H-H2O]+
873.6943464	873.6943	0.0001	0.1145	PGP-44:1)	C50H97O9P	[M+H]+
873.6943464	873.6943	0.0001	0.1145	TG(52:6)	C55H94O6Na	[M+Na]+
874.5372593	874.5357	0.0015	1.7152	PC(42:11)	C50H78NO8PNa	[M+Na]+
874.5372593	874.5359	0.0014	1.6008	PC(39:6(OH))	C47H82NO9PK	[M+K]+
874.5372593	874.5359	0.0014	1.6008	PE(42:6(OH))	C47H82NO9PK	[M+K]+
874.5551639	874.5569	0.0017	1.9438	PS(41:5)	C47H82NO10PNa	[M+Na]+
874.5551639	874.557	0.0018	2.0582	PICer(d38:1)	C44H86NO11PK	[M+K]+
874.5551639	874.557	0.0018	2.0582	PS(38:0(OH))	C44H86NO11PK	[M+K]+
875.5030864	875.5045	0.0014	1.5991	LPIP(32:1)	C41H80O15P2	[M+H]+
875.5030864	875.5045	0.0014	1.5991	PIP(O-32:0(OH))	C41H82O16P2	[M+H-H2O]+

875.5030864	875.5045	0.0014	1.5991	PIP(O-32:1)	C41H80O15P2	[M+H]+
875.5030864	875.5045	0.0014	1.5991	PIP(P-32:0)	C41H80O15P2	[M+H]+
875.5030864	875.5046	0.0015	1.7133	PI(34:1)	C43H81O13PK	[M+K]+
875.5030864	875.5046	0.0015	1.7133	PI(O-34:2(OH))	C43H81O13PK	[M+K]+
875.5030864	875.5046	0.0015	1.7133	PI(P-34:1(OH))	C43H81O13PK	[M+K]+
875.558483	875.5585	0	0.0000	PA(50:12(OH))	C53H81O9P	[M+H-H2O]+
876.4959233	876.494	0.0019	2.1677	PE(44:11)	C49H76NO8PK	[M+K]+
877.4986449	877.4992	0.0005	0.5698	PG40:6(OH))	C46H79O11PK	[M+K]+
877.5010434	877.4992	0.0019	2.1652	PG40:6(OH))	C46H79O11PK	[M+K]+
877.5010434	877.5033	0.0022	2.5071	MIPC(t27:1)	C39H74NO17P	[M+NH4]+
879.4960208	879.4937	0.0023	2.6151	PA(46:10(OH))	C49H77O9PK	[M+K]+
880.5293132	880.5311	0.0018	2.0442	LPIP(31:0)	C40H80O15P2	[M+NH4]+
880.5293132	880.5311	0.0018	2.0442	PIP(O-31:0)	C40H80O15P2	[M+NH4]+
880.5293132	880.5312	0.0019	2.1578	PICer(t36:0(OH))	C42H84NO13PK	[M+K]+
880.5304722	880.5311	0.0006	0.6814	LPIP(31:0)	C40H80O15P2	[M+NH4]+
880.5304722	880.5311	0.0006	0.6814	PIP(O-31:0)	C40H80O15P2	[M+NH4]+
880.5304722	880.5312	0.0007	0.7950	PICer(t36:0(OH))	C42H84NO13PK	[M+K]+
881.4896449	881.4868	0.0028	3.1765	SQDG(41:11)	C50H74O12S	[M+H-H2O]+
881.490585	881.4868	0.0038	4.3109	SQDG(41:11)	C50H74O12S	[M+H-H2O]+
881.490585	881.4939	0.0033	3.7436	PG42:10(OH))	C48H75O11PNa	[M+Na]+
881.490585	881.494	0.0034	3.8571	PIP(O-34:3)	C43H80O15P2	[M+H-H2O]+
881.490585	881.494	0.0034	3.8571	PIP(P-34:2)	C43H80O15P2	[M+H-H2O]+
881.490585	881.4941	0.0035	3.9705	PI(O-36:5)	C45H79O12PK	[M+K]+
881.490585	881.4941	0.0035	3.9705	PI(P-36:4)	C45H79O12PK	[M+K]+
881.5115481	881.5093	0.0022	2.4957	PA(46:9(OH))	C49H79O9PK	[M+K]+
882.4937524	882.495	0.0013	1.4731	MIPC(d28:0)	C40H78NO16PNa	[M+Na]+
882.5392165	882.5396	0.0004	0.4532	SQDG(38:7)	C47H76O12S	[M+NH4]+
882.5413805	882.541	0.0004	0.4532	PE(44:8)	C49H82NO8PK	[M+K]+
884.5633855	884.5647	0.0014	1.5827	PI(P-38:6)	C47H79O12P	[M+NH4]+
885.5236768	885.5252	0.0015	1.6939	PG42:8(OH))	C48H79O11PNa	[M+Na]+
885.5236768	885.5253	0.0016	1.8068	PIP(O-34:1)	C43H84O15P2	[M+H-H2O]+
885.5236768	885.5253	0.0016	1.8068	PIP(P-34:0)	C43H84O15P2	[M+H-H2O]+
885.5236768	885.5254	0.0017	1.9198	PI(O-36:3)	C45H83O12PK	[M+K]+
885.5236768	885.5254	0.0017	1.9198	PI(P-36:2)	C45H83O12PK	[M+K]+
885.5450262	885.5447	0.0003	0.3388	MIPC(m30:2)	C42H78NO15P	[M+NH4]+
886.5272378	886.5287	0.0015	1.6920	MIPC(d30:1)	C42H80NO16P	[M+H]+
886.5272378	886.5287	0.0015	1.6920	MIPC(t30:0)	C42H82NO17P	[M+H-H2O]+
886.5272378	886.5289	0.0016	1.8048	LacCer(t32:2)	C44H81NO14K	[M+K]+

886.5483606	886.5475	0.0009	1.0152	SHexCer(d39:2)	C45H85NO11SK	[M+K]+
887.5593759	887.5604	0.001	1.1267	MIPC(m30:1)	C42H80NO15P	[M+NH4]+
887.5608931	887.5604	0.0005	0.5633	MIPC(m30:1)	C42H80NO15P	[M+NH4]+
887.5608931	887.562	0.0011	1.2394	PI(36:1)	C45H85O13PNa	[M+Na]+
887.5608931	887.562	0.0011	1.2394	PI(O-36:2(OH))	C45H85O13PNa	[M+Na]+
887.5608931	887.562	0.0011	1.2394	PI(P-36:1(OH))	C45H85O13PNa	[M+Na]+
888.564165	888.5631	0.001	1.1254	SHexCer(d39:1)	C45H87NO11SK	[M+K]+
889.5421848	889.5413	0.0009	1.0118	PI(35:1(OH))	C44H83O14PNa	[M+Na]+
889.5421848	889.5437	0.0015	1.6863	PI(37:4(OH))	C46H81O14P	[M+H]+
889.6027999	889.6065	0.0037	4.1591	PC(42:9(OH))	C50H82NO9P	[M+NH4]+
889.6027999	889.6069	0.0041	4.6088	SQDG(39:2)	C48H88O12S	[M+H]+
890.6061509	890.6058	0.0003	0.3368	PC(46:11)	C54H86NO8P	[M+H-H2O]+
892.6754033	892.6766	0.0012	1.3443	PC(42:2)	C50H96NO8PNa	[M+Na]+
892.6754033	892.6766	0.0012	1.3443	PC(O-42:3(OH))	C50H96NO8PNa	[M+Na]+
892.6754033	892.6766	0.0012	1.3443	PC(P-42:2(OH))	C50H96NO8PNa	[M+Na]+
892.6754033	892.6766	0.0012	1.3443	PE(45:2)	C50H96NO8PNa	[M+Na]+
892.6754033	892.6766	0.0012	1.3443	PE(O-45:3(OH))	C50H96NO8PNa	[M+Na]+
892.6754033	892.6766	0.0012	1.3443	PE(P-45:2(OH))	C50H96NO8PNa	[M+Na]+
893.6774852	893.676	0.0015	1.6785	PA(O-48:2)	C51H99O7PK	[M+K]+
893.6774852	893.676	0.0015	1.6785	PA(P-48:1)	C51H99O7PK	[M+K]+
894.6911932	894.6922	0.001	1.1177	PC(42:1)	C50H98NO8PNa	[M+Na]+
894.6911932	894.6922	0.001	1.1177	PC(O-42:2(OH))	C50H98NO8PNa	[M+Na]+
894.6911932	894.6922	0.001	1.1177	PC(P-42:1(OH))	C50H98NO8PNa	[M+Na]+
894.6911932	894.6922	0.001	1.1177	PE(45:1)	C50H98NO8PNa	[M+Na]+
894.6911932	894.6922	0.001	1.1177	PE(O-45:2(OH))	C50H98NO8PNa	[M+Na]+
894.6911932	894.6922	0.001	1.1177	PE(P-45:1(OH))	C50H98NO8PNa	[M+Na]+
895.6940619	895.6916	0.0024	2.6795	PA(O-48:1)	C51H101O7PK	[M+K]+
895.6940619	895.6916	0.0024	2.6795	PA(P-48:0)	C51H101O7PK	[M+K]+
896.5375015	896.5366	0.0009	1.0039	DGDG(32:7)	C47H74O15	[M+NH4]+
897.4851841	897.4865	0.0013	1.4485	LPIP(32:1)	C41H80O15P2Na	[M+Na]+
897.4851841	897.4865	0.0013	1.4485	PIP(O-32:1)	C41H80O15P2Na	[M+Na]+
897.4851841	897.4865	0.0013	1.4485	PIP(P-32:0)	C41H80O15P2Na	[M+Na]+
897.5415471	897.5406	0.0009	1.0027	PA(47:8(OH))	C50H83O9PK	[M+K]+
898.4888226	898.49	0.0011	1.2243	MIPC(t28:0)	C40H78NO17PNa	[M+Na]+
898.4915682	898.49	0.0016	1.7808	MIPC(t28:0)	C40H78NO17PNa	[M+Na]+
898.5418933	898.544	0.0021	2.3371	PI(38:7)	C47H77O13P	[M+NH4]+
901.518397	901.5202	0.0018	1.9966	PIP(34:0)	C43H84O16P2	[M+H-H2O]+
901.518397	901.5202	0.0018	1.9966	PIP(O-34:1(OH))	C43H84O16P2	[M+H-H2O]+

901.518397	901.5202	0.0018	1.9966	PIP(O-34:2)	C43H82O15P2	[M+H]+
901.518397	901.5202	0.0018	1.9966	PIP(P-34:0(OH))	C43H84O16P2	[M+H-H2O]+
901.518397	901.5202	0.0018	1.9966	PIP(P-34:1)	C43H82O15P2	[M+H]+
901.518397	901.5203	0.0019	2.1076	PI(36:2)	C45H83O13PK	[M+K]+
901.518397	901.5203	0.0019	2.1076	PI(O-36:3(OH))	C45H83O13PK	[M+K]+
901.518397	901.5203	0.0019	2.1076	PI(P-36:2(OH))	C45H83O13PK	[M+K]+
902.5059473	902.5083	0.0023	2.5485	SQDG(40:11)	C49H72O12S	[M+NH4]+
903.5186775	903.5148	0.0039	4.3165	PG42:7(OH))	C48H81O11PK	[M+K]+
903.5337943	903.5358	0.002	2.2135	PIP(O-34:0(OH))	C43H86O16P2	[M+H-H2O]+
903.5337943	903.5358	0.002	2.2135	PIP(O-34:1)	C43H84O15P2	[M+H]+
903.5337943	903.5358	0.002	2.2135	PIP(P-34:0)	C43H84O15P2	[M+H]+
903.5337943	903.5359	0.0021	2.3242	PI(36:1)	C45H85O13PK	[M+K]+
903.5337943	903.5359	0.0021	2.3242	PI(O-36:2(OH))	C45H85O13PK	[M+K]+
903.5337943	903.5359	0.0021	2.3242	PI(P-36:1(OH))	C45H85O13PK	[M+K]+
904.527288	904.5253	0.002	2.2111	PE(46:11)	C51H80NO8PK	[M+K]+
905.5297662	905.5305	0.0007	0.7730	PG42:6(OH))	C48H83O11PK	[M+K]+
905.5306659	905.5305	0.0002	0.2209	PG42:6(OH))	C48H83O11PK	[M+K]+
906.5337597	906.5314	0.0023	2.5371	MIPC(m31:1)	C43H82NO15PNa	[M+Na]+
906.5455499	906.5467	0.0012	1.3237	PIP(O-33:1)	C42H82O15P2	[M+NH4]+
906.5455499	906.5467	0.0012	1.3237	PIP(P-33:0)	C42H82O15P2	[M+NH4]+
907.5488456	907.5502	0.0014	1.5426	MIPC(t29:0)	C41H80NO17P	[M+NH4]+
907.552372	907.5502	0.0022	2.4241	MIPC(t29:0)	C41H80NO17P	[M+NH4]+
913.5765413	913.576	0.0005	0.5473	MIPC(m32:2)	C44H82NO15P	[M+NH4]+
913.5765413	913.5776	0.0011	1.2041	PI(38:2)	C47H87O13PNa	[M+Na]+
913.5765413	913.5776	0.0011	1.2041	PI(O-38:3(OH))	C47H87O13PNa	[M+Na]+
913.5765413	913.5776	0.0011	1.2041	PI(P-38:2(OH))	C47H87O13PNa	[M+Na]+
915.4617912	915.4606	0.0011	1.2016	PIP(31:0(OH))	C40H78O17P2Na	[M+Na]+
915.4617912	915.463	0.0012	1.3108	PI(38:9(OH))	C47H73O14PNa	[M+Na]+
915.4617912	915.4631	0.0013	1.4200	PIP(33:3(OH))	C42H76O17P2	[M+H]+
915.5565813	915.5569	0.0003	0.3277	PI(37:2(OH))	C46H85O14PNa	[M+Na]+
920.5012234	920.5048	0.0036	3.9109	PS(44:11(OH))	C50H76NO11PNa	[M+Na]+
920.7066897	920.7079	0.0012	1.3033	PC(44:2)	C52H100NO8PNa	[M+Na]+
920.7066897	920.7079	0.0012	1.3033	PC(O-44:3(OH))	C52H100NO8PNa	[M+Na]+
920.7066897	920.7079	0.0012	1.3033	PC(P-44:2(OH))	C52H100NO8PNa	[M+Na]+
920.7066897	920.7079	0.0012	1.3033	PE(47:2)	C52H100NO8PNa	[M+Na]+
920.7066897	920.7079	0.0012	1.3033	PE(O-47:3(OH))	C52H100NO8PNa	[M+Na]+
920.7066897	920.7079	0.0012	1.3033	PE(P-47:2(OH))	C52H100NO8PNa	[M+Na]+
922.6154498	922.6144	0.0011	1.1923	PS(43:3(OH))	C49H90NO11PNa	[M+Na]+

922.6154498	922.6168	0.0013	1.4090	PG45:8(OH))	C51H85O11P	[M+NH4]+
922.6154498	922.6168	0.0013	1.4090	PS(45:6(OH))	C51H88NO11P	[M+H]+
923.6188256	923.616	0.0028	3.0316	PA(52:11(OH))	C55H87O9P	[M+H]+
923.6188256	923.6219	0.0031	3.3564	PI(39:1(OH))	C48H91O14P	[M+H]+
924.6195931	924.6172	0.0024	2.5957	MIPC(m35:0)	C47H92NO15P	[M+H-H2O]+
924.6195931	924.6172	0.0024	2.5957	PI(38:2(OH))	C47H87O14P	[M+NH4]+
924.6211321	924.6172	0.004	4.3261	MIPC(m35:0)	C47H92NO15P	[M+H-H2O]+
924.6211321	924.6172	0.004	4.3261	PI(38:2(OH))	C47H87O14P	[M+NH4]+
925.6239976	925.6247	0.0007	0.7562	DGDG(36:3)	C51H90O15	[M+H-H2O]+
926.6259962	926.6245	0.0015	1.6188	PC(44:7(OH))	C52H90NO9PNa	[M+Na]+
926.6259962	926.6245	0.0015	1.6188	PE(47:7(OH))	C52H90NO9PNa	[M+Na]+
926.6259962	926.6245	0.0015	1.6188	PS(P-46:6)	C52H90NO9PNa	[M+Na]+
926.6259962	926.6247	0.0013	1.4029	PS(43:1)	C49H94NO10PK	[M+K]+
926.6259962	926.6247	0.0013	1.4029	PS(P-43:1(OH))	C49H94NO10PK	[M+K]+
926.6259962	926.6269	0.0009	0.9713	PC(46:10(OH))	C54H88NO9P	[M+H]+
926.6259962	926.6269	0.0009	0.9713	PS(48:8)	C54H90NO10P	[M+H-H2O]+
927.5228716	927.5263	0.0034	3.6657	SQDG(41:8)	C50H80O12SNa	[M+Na]+
929.5342267	929.5305	0.0038	4.0881	PG44:8(OH))	C50H83O11PK	[M+K]+
930.5429554	930.541	0.002	2.1493	PE(48:12)	C53H82NO8PK	[M+K]+
931.5465287	931.5461	0.0004	0.4294	PG44:7(OH))	C50H85O11PK	[M+K]+
938.5350774	938.5366	0.0015	1.5982	PIP(33:0(OH))	C42H82O17P2	[M+NH4]+
938.5350774	938.5367	0.0016	1.7048	MIPC(m32:0)	C44H86NO15PK	[M+K]+
940.6272342	940.6273	0.0001	0.1063	PI(P-42:6)	C51H87O12P	[M+NH4]+
941.4784943	941.4787	0.0002	0.2124	PI(40:10(OH))	C49H75O14PNa	[M+Na]+
941.4784943	941.4787	0.0002	0.2124	PIP(35:4(OH))	C44H78O17P2	[M+H]+
950.5389341	950.5366	0.0024	2.5249	PIP(34:1(OH))	C43H82O17P2	[M+NH4]+
950.5389341	950.5367	0.0023	2.4197	MIPC(m33:1)	C45H86NO15PK	[M+K]+
950.5406378	950.5366	0.0041	4.3134	PIP(34:1(OH))	C43H82O17P2	[M+NH4]+
950.5747869	950.5753	0.0005	0.5260	PI(42:9)	C51H81O13P	[M+NH4]+
951.5424185	951.5441	0.0016	1.6815	PIM1(32:2)	C47H85O18P	[M+H-H2O]+
951.5424185	951.5442	0.0018	1.8917	DGDG(34:4)	C49H84O15K	[M+K]+
953.598322	953.6015	0.0031	3.2508	PS(48:12)	C54H82NO10P	[M+NH4]+
954.6052256	954.6066	0.0014	1.4666	PI(42:7)	C51H85O13P	[M+NH4]+
961.5976064	961.5972	0.0004	0.4160	MIPC(t33:1)	C45H86NO17P	[M+NH4]+
961.6010485	961.5972	0.0039	4.0558	MIPC(t33:1)	C45H86NO17P	[M+NH4]+
961.6010485	961.6045	0.0035	3.6398	SQDG(43:5)	C52H90O12SNa	[M+Na]+
962.6073875	962.6093	0.0019	1.9738	PIP(O-37:1)	C46H90O15P2	[M+NH4]+
962.6073875	962.6093	0.0019	1.9738	PIP(P-37:0)	C46H90O15P2	[M+NH4]+

962.6106049	962.6093	0.0013	1.3505	PIP(O-37:1)	C46H90O15P2	[M+NH4]+
962.6106049	962.6093	0.0013	1.3505	PIP(P-37:0)	C46H90O15P2	[M+NH4]+
964.5540372	964.5546	0.0005	0.5184	PI(42:10(OH))	C51H79O14P	[M+NH4]+
967.4941481	967.4943	0.0002	0.2067	PI(42:11(OH))	C51H77O14PNa	[M+Na]+
967.4941481	967.4944	0.0002	0.2067	PIP(37:5(OH))	C46H80O17P2	[M+H]+
977.5567112	977.5597	0.003	3.0689	PIM1(34:3)	C49H87O18P	[M+H-H2O]+
977.5567112	977.5598	0.0031	3.1712	DGDG(36:5)	C51H86O15K	[M+K]+
978.5697295	978.5679	0.0019	1.9416	PIP(36:1(OH))	C45H86O17P2	[M+NH4]+
978.5697295	978.568	0.0018	1.8394	MIPC(m35:1)	C47H90NO15PK	[M+K]+
978.5703761	978.5679	0.0025	2.5548	PIP(36:1(OH))	C45H86O17P2	[M+NH4]+
978.5703761	978.568	0.0024	2.4526	MIPC(m35:1)	C47H90NO15PK	[M+K]+
979.5736174	979.5754	0.0017	1.7354	PIM1(34:2)	C49H89O18P	[M+H-H2O]+
979.5736174	979.5755	0.0019	1.9396	DGDG(36:4)	C51H88O15K	[M+K]+
980.5765718	980.5777	0.0012	1.2238	PS(48:9)	C54H88NO10PK	[M+K]+
980.6951166	980.695	0.0001	0.1020	PG49:7(OH))	C55H95O11P	[M+NH4]+
980.6951166	980.695	0.0001	0.1020	PS(49:5(OH))	C55H98NO11P	[M+H]+
981.5791488	981.5804	0.0012	1.2225	PIP(O-38:1)	C47H92O15P2Na	[M+Na]+
981.5791488	981.5804	0.0012	1.2225	PIP(P-38:0)	C47H92O15P2Na	[M+Na]+
981.5791488	981.5828	0.0036	3.6675	PIP(40:2)	C49H92O16P2	[M+H-H2O]+
981.5791488	981.5828	0.0036	3.6675	PIP(O-40:3(OH))	C49H92O16P2	[M+H-H2O]+
981.5791488	981.5828	0.0036	3.6675	PIP(O-40:4)	C49H90O15P2	[M+H]+
981.5791488	981.5828	0.0036	3.6675	PIP(P-40:2(OH))	C49H92O16P2	[M+H-H2O]+
981.5791488	981.5828	0.0036	3.6675	PIP(P-40:3)	C49H90O15P2	[M+H]+
981.5791488	981.5829	0.0037	3.7694	PI(42:4)	C51H91O13PK	[M+K]+
981.5791488	981.5829	0.0037	3.7694	PI(O-42:5(OH))	C51H91O13PK	[M+K]+
981.5791488	981.5829	0.0037	3.7694	PI(P-42:4(OH))	C51H91O13PK	[M+K]+
981.5807279	981.5804	0.0004	0.4075	PIP(O-38:1)	C47H92O15P2Na	[M+Na]+
981.5807279	981.5804	0.0004	0.4075	PIP(P-38:0)	C47H92O15P2Na	[M+Na]+
981.6981972	981.7002	0.002	2.0373	PI(43:0(OH))	C52H101O14P	[M+H]+
982.5439909	982.5416	0.0024	2.4426	PIP(38:5)	C47H82O16P2	[M+NH4]+
982.5439909	982.5416	0.0024	2.4426	PIP(O-38:6(OH))	C47H82O16P2	[M+NH4]+
982.5439909	982.5416	0.0024	2.4426	PIP(P-38:5(OH))	C47H82O16P2	[M+NH4]+
983.5793864	983.5774	0.002	2.0334	PG48:9(OH))	C54H89O11PK	[M+K]+
984.5896618	984.5865	0.0031	3.1485	SQDG(46:12)	C55H82O12S	[M+NH4]+
984.5912436	984.5865	0.0047	4.7736	SQDG(46:12)	C55H82O12S	[M+NH4]+
985.5947261	985.5931	0.0017	1.7248	PG48:8(OH))	C54H91O11PK	[M+K]+
987.5977826	987.5957	0.0021	2.1264	PI(46:10)	C55H87O13P	[M+H]+
987.5977826	987.5957	0.0021	2.1264	PI(46:9(OH))	C55H89O14P	[M+H-H2O]+

988.5889803	988.5886	0.0004	0.4046	PIP(38:2)	C47H88O16P2	[M+NH4]+
988.5889803	988.5886	0.0004	0.4046	PIP(O-38:3(OH))	C47H88O16P2	[M+NH4]+
988.5889803	988.5886	0.0004	0.4046	PIP(P-38:2(OH))	C47H88O16P2	[M+NH4]+
988.5912362	988.5886	0.0027	2.7312	PIP(38:2)	C47H88O16P2	[M+NH4]+
988.5912362	988.5886	0.0027	2.7312	PIP(O-38:3(OH))	C47H88O16P2	[M+NH4]+
988.5912362	988.5886	0.0027	2.7312	PIP(P-38:2(OH))	C47H88O16P2	[M+NH4]+
990.5673472	990.5679	0.0005	0.5048	PIP(37:2(OH))	C46H86O17P2	[M+NH4]+
990.5673472	990.568	0.0006	0.6057	MIPC(m36:2)	C48H90NO15PK	[M+K]+
994.5650051	994.5629	0.0021	2.1115	MIPC(d35:1)	C47H90NO16PK	[M+K]+
995.5466534	995.5481	0.0015	1.5067	CDP-DG(34:2)	C46H81N3O15P2	[M+NH4]+
995.5678945	995.5679	0	0.0000	PIM1(32:0)	C47H89O18PNa	[M+Na]+
1000.551792	1000.5522	0.0004	0.3998	PIP(38:4(OH))	C47H84O17P2	[M+NH4]+
1000.586146	1000.5886	0.0024	2.3986	PIP(39:3)	C48H88O16P2	[M+NH4]+
1001.555036	1001.5515	0.0036	3.5944	PIP(42:6)	C51H88O16P2	[M+H-H2O]+
1001.555036	1001.5515	0.0036	3.5944	PIP(P-42:6(OH))	C51H88O16P2	[M+H-H2O]+
1001.555036	1001.5516	0.0035	3.4946	PI(44:8)	C53H87O13PK	[M+K]+
1002.568336	1002.5679	0.0005	0.4987	PIP(38:3(OH))	C47H86O17P2	[M+NH4]+
1005.589195	1005.591	0.0018	1.7900	PIM1(36:3)	C51H91O18P	[M+H-H2O]+
1005.589195	1005.5911	0.0019	1.8894	DGDG(38:5)	C53H90O15K	[M+K]+
1005.590298	1005.591	0.0007	0.6961	PIM1(36:3)	C51H91O18P	[M+H-H2O]+
1005.590298	1005.5911	0.0008	0.7956	DGDG(38:5)	C53H90O15K	[M+K]+
1006.5893	1006.5934	0.0041	4.0731	PS(50:10)	C56H90NO10PK	[M+K]+
1006.594714	1006.5934	0.0013	1.2915	PS(50:10)	C56H90NO10PK	[M+K]+
1006.709427	1006.7083	0.0012	1.1920	PS(49:3(OH))	C55H102NO11PNa	[M+Na]+
1006.709427	1006.7107	0.0012	1.1920	PG51:8(OH))	C57H97O11P	[M+NH4]+
1006.709427	1006.7107	0.0012	1.1920	PS(51:6(OH))	C57H100NO11P	[M+H]+
1006.710999	1006.7107	0.0003	0.2980	PG51:8(OH))	C57H97O11P	[M+NH4]+
1006.710999	1006.7107	0.0003	0.2980	PS(51:6(OH))	C57H100NO11P	[M+H]+
1007.714102	1007.7158	0.0017	1.6870	PI(45:1(OH))	C54H103O14P	[M+H]+
1008.726366	1008.7263	0	0.0000	PG51:7(OH))	C57H99O11P	[M+NH4]+
1008.726366	1008.7263	0	0.0000	PS(51:5(OH))	C57H102NO11P	[M+H]+
1010.572768	1010.5729	0.0002	0.1979	PIP(40:5)	C49H86O16P2	[M+NH4]+
1010.572768	1010.5729	0.0002	0.1979	PIP(O-40:6(OH))	C49H86O16P2	[M+NH4]+
1010.572768	1010.5729	0.0002	0.1979	PIP(P-40:5(OH))	C49H86O16P2	[M+NH4]+
1012.548854	1012.5522	0.0033	3.2591	PIP(39:5(OH))	C48H84O17P2	[M+NH4]+
1012.551037	1012.5522	0.0012	1.1851	PIP(39:5(OH))	C48H84O17P2	[M+NH4]+
1012.622681	1012.6178	0.0049	4.8389	SQDG(48:12)	C57H86O12S	[M+NH4]+
1013.553608	1013.5515	0.0021	2.0719	PIP(43:7)	C52H88O16P2	[M+H-H2O]+

1013.626286	1013.6244	0.0019	1.8745	PG50:8(OH))	C56H95O11PK	[M+K]+
1015.573426	1015.5755	0.0021	2.0678	DGDG(39:7)	C54H88O15K	[M+K]+
1017.553621	1017.5522	0.0014	1.3759	PIM1(34:3)	C49H87O18PNa	[M+Na]+
1019.546593	1019.5481	0.0015	1.4712	CDP-DG(36:4)	C48H81N3O15P2	[M+NH4]+
1019.566236	1019.5679	0.0016	1.5693	PIM1(34:2)	C49H89O18PNa	[M+Na]+
1020.568893	1020.5727	0.0038	3.7234	PS(50:11(OH))	C56H88NO11PK	[M+K]+
1020.570838	1020.5727	0.0018	1.7637	PS(50:11(OH))	C56H88NO11PK	[M+K]+
1021.56222	1021.5638	0.0015	1.4683	CDP-DG(36:3)	C48H83N3O15P2	[M+NH4]+
1023.578096	1023.5794	0.0013	1.2701	CDP-DG(36:2)	C48H85N3O15P2	[M+NH4]+
1025.584884	1025.5856	0.0007	0.6825	PIP(O-40:1)	C49H96O15P2K	[M+K]+
1025.584884	1025.5856	0.0007	0.6825	PIP(P-40:0)	C49H96O15P2K	[M+K]+
1030.637389	1030.6379	0.0005	0.4851	PI(48:11)	C57H89O13P	[M+NH4]+
1032.554762	1032.5573	0.0025	2.4212	PIP(42:8)	C51H84O16P2	[M+NH4]+
1034.742216	1034.742	0.0002	0.1933	PG53:8(OH))	C59H101O11P	[M+NH4]+
1034.742216	1034.742	0.0002	0.1933	PS(53:6(OH))	C59H104NO11P	[M+H]+
1039.534856	1039.5307	0.0041	3.9441	PIP(44:10)	C53H84O16P2	[M+H]+
1039.534856	1039.5307	0.0041	3.9441	PIP(44:9(OH))	C53H86O17P2	[M+H-H2O]+
1039.534856	1039.5309	0.004	3.8479	PI(46:11(OH))	C55H85O14PK	[M+K]+
1041.548023	1041.5464	0.0016	1.5362	PIP(44:8(OH))	C53H88O17P2	[M+H-H2O]+
1041.548023	1041.5464	0.0016	1.5362	PIP(44:9)	C53H86O16P2	[M+H]+
1041.548023	1041.5465	0.0015	1.4402	PI(46:10(OH))	C55H87O14PK	[M+K]+
1045.579405	1045.5777	0.0017	1.6259	PIP(44:6(OH))	C53H92O17P2	[M+H-H2O]+
1045.579405	1045.5777	0.0017	1.6259	PIP(44:7)	C53H90O16P2	[M+H]+
1045.579405	1045.5778	0.0016	1.5303	PI(46:8(OH))	C55H91O14PK	[M+K]+
1045.582079	1045.5835	0.0014	1.3390	PIM1(36:3)	C51H91O18PNa	[M+Na]+
1049.593437	1049.5951	0.0016	1.5244	CDP-DG(38:3)	C50H87N3O15P2	[M+NH4]+
1057.519295	1057.5179	0.0014	1.3239	PIP(42:6)	C51H88O16P2K	[M+K]+
1057.519295	1057.5179	0.0014	1.3239	PIP(P-42:6(OH))	C51H88O16P2K	[M+K]+
1057.522006	1057.5179	0.0041	3.8770	PIP(42:6)	C51H88O16P2K	[M+K]+
1057.522006	1057.5179	0.0041	3.8770	PIP(P-42:6(OH))	C51H88O16P2K	[M+K]+
1058.632782	1058.6305	0.0023	2.1726	PIP(42:3(OH))	C51H94O17P2	[M+NH4]+
1059.636155	1059.6381	0.0019	1.7931	DGDG(42:6)	C57H96O15K	[M+K]+
1060.637825	1060.6403	0.0025	2.3571	PS(54:11)	C60H96NO10PK	[M+K]+
1065.548349	1065.5464	0.002	1.8770	PIP(46:10(OH))	C55H88O17P2	[M+H-H2O]+
1065.548349	1065.5464	0.002	1.8770	PIP(46:11)	C55H86O16P2	[M+H]+
1065.548349	1065.5465	0.0018	1.6893	PI(48:12(OH))	C57H87O14PK	[M+K]+
1067.564273	1067.562	0.0022	2.0608	PIP(46:10)	C55H88O16P2	[M+H]+
1067.564273	1067.562	0.0022	2.0608	PIP(46:9(OH))	C55H90O17P2	[M+H-H2O]+

1067.564273	1067.5622	0.0021	1.9671	PI(48:11(OH))	C57H89O14PK	[M+K]+
1070.628489	1070.6305	0.002	1.8681	PIP(43:4(OH))	C52H94O17P2	[M+NH4]+
1075.628949	1075.6246	0.0043	3.9977	PIP(46:5(OH))	C55H98O17P2	[M+H-H2O]+
1075.628949	1075.6246	0.0043	3.9977	PIP(46:6)	C55H96O16P2	[M+H]+
1075.628949	1075.6246	0.0043	3.9977	PIP(P-46:6(OH))	C55H96O16P2	[M+H]+
1075.628949	1075.6248	0.0042	3.9047	PI(48:7(OH))	C57H97O14PK	[M+K]+
1075.628949	1075.6328	0.0039	3.6258	DGDG(45:11)	C60H92O15Na	[M+Na]+
1075.63135	1075.6328	0.0015	1.3945	DGDG(45:11)	C60H92O15Na	[M+Na]+
1077.647435	1077.6485	0.0011	1.0207	DGDG(45:10)	C60H94O15Na	[M+Na]+
1081.539467	1081.5389	0.0006	0.5548	PIP(44:8(OH))	C53H88O17P2Na	[M+Na]+
1081.541081	1081.5413	0.0002	0.1849	PIP(46:11(OH))	C55H86O17P2	[M+H]+
1086.664336	1086.6618	0.0026	2.3926	PIP(44:3(OH))	C53H98O17P2	[M+NH4]+
1092.6128	1092.6148	0.002	1.8305	PIP(45:7(OH))	C54H92O17P2	[M+NH4]+
1093.616496	1093.6141	0.0024	2.1946	PIP(49:9)	C58H96O16P2	[M+H-H2O]+
1099.629263	1099.6246	0.0046	4.1832	PIP(48:7(OH))	C57H98O17P2	[M+H-H2O]+
1099.629263	1099.6246	0.0046	4.1832	PIP(48:8)	C57H96O16P2	[M+H]+
1099.629263	1099.6248	0.0045	4.0923	PI(50:9(OH))	C59H97O14PK	[M+K]+
1105.544672	1105.5432	0.0015	1.3568	M(IP)2C(d27:0)	C45H87NO24P2	[M+NH4]+
1111.588503	1111.5883	0.0003	0.2699	PIP(48:10(OH))	C57H92O17P2	[M+H]+
1111.591996	1111.5883	0.0037	3.3286	PIP(48:10(OH))	C57H92O17P2	[M+H]+
1121.60966	1121.609	0.0007	0.6241	PIP(50:10(OH))	C59H96O17P2	[M+H-H2O]+
1121.60966	1121.609	0.0007	0.6241	PIP(50:11)	C59H94O16P2	[M+H]+
1121.60966	1121.6091	0.0006	0.5349	PI(52:12(OH))	C61H95O14PK	[M+K]+
1121.611296	1121.609	0.0023	2.0506	PIP(50:10(OH))	C59H96O17P2	[M+H-H2O]+
1121.611296	1121.609	0.0023	2.0506	PIP(50:11)	C59H94O16P2	[M+H]+
1121.611296	1121.6091	0.0022	1.9615	PI(52:12(OH))	C61H95O14PK	[M+K]+
1131.559159	1131.5588	0.0004	0.3535	M(IP)2C(d29:1)	C47H89NO24P2	[M+NH4]+
1131.561938	1131.5588	0.0031	2.7396	M(IP)2C(d29:1)	C47H89NO24P2	[M+NH4]+
1137.604836	1137.6039	0.0009	0.7911	PIP(50:11(OH))	C59H94O17P2	[M+H]+
1147.592222	1147.5901	0.0021	1.8299	M(IP)2C(d30:0)	C48H93NO24P2	[M+NH4]+
1153.574432	1153.5754	0.001	0.8669	PIP(48:8(OH))	C57H96O17P2K	[M+K]+
1159.589269	1159.5901	0.0008	0.6899	M(IP)2C(d31:1)	C49H93NO24P2	[M+NH4]+
1159.59214	1159.5901	0.002	1.7247	M(IP)2C(d31:1)	C49H93NO24P2	[M+NH4]+
1161.604117	1161.6015	0.0026	2.2383	PIP(50:10(OH))	C59H96O17P2Na	[M+Na]+
1161.604117	1161.6058	0.0016	1.3774	M(IP)2C(d31:0)	C49H95NO24P2	[M+NH4]+
1179.586178	1179.5911	0.0049	4.1540	PIP(50:9(OH))	C59H98O17P2K	[M+K]+

Supplementary Table 6: Annotations for infection of BUVEC in negative-ion mode

Input Mass	Matched Mass	Delta	ppm	Name	Formula	Adduct
331.1319174	331.1318	0.0001	0.3020	FA 16:4;O3	C16H24O5	[M+Cl]-
345.1477908	345.1474	0.0004	1.1589	FA 17:4;O3	C17H26O5	[M+Cl]-
345.1477908	345.1474	0.0004	1.1589	MG 14:4;O	C17H26O5	[M+Cl]-
363.1947318	363.1942	0.0005	1.3767	LPA O-14:2	C17H33O6P	[M-H]-
363.1947318	363.1944	0.0003	0.8260	FA 18:2;O3	C18H32O5	[M+Cl]-
363.1947318	363.1944	0.0003	0.8260	MG 15:2;O	C18H32O5	[M+Cl]-
378.1596144	378.1592	0.0004	1.0578	NAT 14:2;O3	C16H29NO7S	[M-H]-
378.1596144	378.1592	0.0004	1.0578	NAT 13:2;O	C15H27NO5S	[M+Formate]-
378.1596144	378.1592	0.0004	1.0578	NAT 12:2;O	C14H25NO5S	[M+OAc]-
390.214243	390.2133	0.0009	2.3064	CAR 11:1;O4	C18H33NO8	[M-H]-
390.214243	390.2133	0.0009	2.3064	CAR 10:1;O2	C17H31NO6	[M+Formate]-
390.214243	390.2133	0.0009	2.3064	NAE 15:2;O4	C17H31NO6	[M+Formate]-
390.214243	390.2133	0.0009	2.3064	NAE 14:2;O4	C16H29NO6	[M+OAc]-
431.2575854	431.2568	0.0008	1.8550	LPA O-19:3	C22H41O6P	[M-H]-
431.2575854	431.257	0.0006	1.3913	DG 20:2	C23H40O5	[M+Cl]-
431.2575854	431.257	0.0006	1.3913	DG O-20:3;O	C23H40O5	[M+Cl]-
431.2575854	431.257	0.0006	1.3913	FA 23:3;O3	C23H40O5	[M+Cl]-
431.2575854	431.257	0.0006	1.3913	MG 20:3;O	C23H40O5	[M+Cl]-
431.2575854	431.257	0.0006	1.3913	ST 23:0;O5	C23H40O5	[M+Cl]-
443.2575328	443.2568	0.0007	1.5792	LPA O-20:4	C23H41O6P	[M-H]-
443.2575328	443.257	0.0005	1.1280	DG 21:3	C24H40O5	[M+Cl]-
443.2575328	443.257	0.0005	1.1280	DG O-21:4;O	C24H40O5	[M+Cl]-
443.2575328	443.257	0.0005	1.1280	FA 24:4;O3	C24H40O5	[M+Cl]-
443.2575328	443.257	0.0005	1.1280	MG 21:4;O	C24H40O5	[M+Cl]-
443.2575328	443.257	0.0005	1.1280	ST 24:1;O5	C24H40O5	[M+Cl]-
444.2609521	444.2603	0.0007	1.5757	CAR 15:2;O4	C22H39NO8	[M-H]-
444.2609521	444.2603	0.0007	1.5757	CAR 14:2;O2	C21H37NO6	[M+Formate]-
444.2609521	444.2603	0.0007	1.5757	NAE 19:3;O4	C21H37NO6	[M+Formate]-
444.2609521	444.2603	0.0007	1.5757	CAR 13:2;O2	C20H35NO6	[M+OAc]-
444.2609521	444.2603	0.0007	1.5757	NAE 18:3;O4	C20H35NO6	[M+OAc]-
456.1440745	456.1431	0.001	2.1923	ST 19:4;O7;G	C21H27NO8	[M+Cl]-
460.2088339	460.2106	0.0018	3.9113	LPE 16:4;O	C21H36NO8P	[M-H]-
460.2088339	460.2106	0.0018	3.9113	LPS O-15:4	C21H36NO8P	[M-H]-
460.2088339	460.2108	0.0019	4.1285	CAR 15:4;O3	C22H35NO7	[M+Cl]-
460.2088339	460.2108	0.0019	4.1285	ST 20:1;O6;G	C22H35NO7	[M+Cl]-

460.2088339	460.2106	0.0018	3.9113	LPC 14:4;O	C22H38NO8P	[M-CH3]-
468.1429905	468.1431	0.0001	0.2136	ST 20:5;O7;G	C22H27NO8	[M+Cl]-
472.1382548	472.138	0.0003	0.6354	ST 19:4;O8;G	C21H27NO9	[M+Cl]-
476.2039909	476.2055	0.0015	3.1499	LPS 15:3	C21H36NO9P	[M-H]-
476.2039909	476.2055	0.0015	3.1499	LPS O-15:4;O	C21H36NO9P	[M-H]-
476.2039909	476.2057	0.0017	3.5699	CAR 15:4;O4	C22H35NO8	[M+Cl]-
476.2039909	476.2057	0.0017	3.5699	ST 20:1;O7;G	C22H35NO8	[M+Cl]-
476.2039909	476.2055	0.0015	3.1499	LPE 15:4	C20H34NO7P	[M+Formate]-
476.2039909	476.2055	0.0015	3.1499	LPE 14:4	C19H32NO7P	[M+OAc]-
481.1149564	481.1174	0.0024	4.9884	ST 22:6;O7;S	C22H26O10S	[M-H]-
481.1149564	481.1174	0.0024	4.9884	ST 21:6;O5;S	C21H24O8S	[M+Formate]-
483.1255415	483.125	0.0006	1.2419	ST 23:6;O4;S	C23H28O7S	[M+Cl]-
485.1413733	485.1406	0.0007	1.4429	ST 23:5;O4;S	C23H30O7S	[M+Cl]-
485.1413733	485.143	0.0016	3.2980	BMP 11:2;O	C17H29O11P	[M+Formate]-
485.1413733	485.143	0.0016	3.2980	BMP 10:2;O	C16H27O11P	[M+OAc]-
486.1541948	486.1536	0.0006	1.2342	ST 20:4;O8;G	C22H29NO9	[M+Cl]-
486.2248007	486.2262	0.0014	2.8793	LPE 18:5;O	C23H38NO8P	[M-H]-
486.2248007	486.2264	0.0016	3.2906	ST 22:2;O6;G	C24H37NO7	[M+Cl]-
497.1345124	497.1349	0.0004	0.8046	BMP 14:4	C20H31O10P	[M+Cl]-
499.1205021	499.1199	0.0006	1.2021	ST 23:6;O5;S	C23H28O8S	[M+Cl]-
501.1364143	501.1356	0.0009	1.7959	ST 23:5;O5;S	C23H30O8S	[M+Cl]-
502.2194061	502.2212	0.0017	3.3850	LPS 17:4	C23H38NO9P	[M-H]-
502.2194061	502.2213	0.0019	3.7832	ST 22:2;O7;G	C24H37NO8	[M+Cl]-
502.2195957	502.2212	0.0016	3.1858	LPS 17:4	C23H38NO9P	[M-H]-
502.2195957	502.2213	0.0017	3.3850	ST 22:2;O7;G	C24H37NO8	[M+Cl]-
503.1522698	503.1512	0.0011	2.1862	ST 23:4;O5;S	C23H32O8S	[M+Cl]-
513.1284925	513.1298	0.0013	2.5335	BMP 14:4;O	C20H31O11P	[M+Cl]-
513.1295621	513.1298	0.0002	0.3898	BMP 14:4;O	C20H31O11P	[M+Cl]-
515.2785154	515.2779	0.0006	1.1644	LPA 23:4;O	C26H45O8P	[M-H]-
515.2785154	515.2779	0.0006	1.1644	LPG O-20:5	C26H45O8P	[M-H]-
515.2785154	515.2779	0.0006	1.1644	PA 23:3	C26H45O8P	[M-H]-
515.2785154	515.2779	0.0006	1.1644	PA O-23:4;O	C26H45O8P	[M-H]-
515.2785154	515.2781	0.0004	0.7763	DG 24:4;O2	C27H44O7	[M+Cl]-
515.2785154	515.2781	0.0004	0.7763	ST 21:0;O;GlcA	C27H44O7	[M+Cl]-
515.2785154	515.2781	0.0004	0.7763	ST 21:1;O2;Hex	C27H44O7	[M+Cl]-
515.2785154	515.2781	0.0004	0.7763	ST 27:2;O7	C27H44O7	[M+Cl]-
515.2785154	515.2779	0.0006	1.1644	LPA O-22:5	C25H43O6P	[M+Formate]-
515.2785154	515.2779	0.0006	1.1644	LPA O-21:5	C24H41O6P	[M+OAc]-

515.2787414	515.2779	0.0008	1.5526	LPA 23:4;O	C26H45O8P	[M-H]-
515.2787414	515.2779	0.0008	1.5526	LPG O-20:5	C26H45O8P	[M-H]-
515.2787414	515.2779	0.0008	1.5526	PA 23:3	C26H45O8P	[M-H]-
515.2787414	515.2779	0.0008	1.5526	PA O-23:4;O	C26H45O8P	[M-H]-
515.2787414	515.2781	0.0006	1.1644	DG 24:4;O2	C27H44O7	[M+Cl]-
515.2787414	515.2781	0.0006	1.1644	ST 21:0;O;GlcA	C27H44O7	[M+Cl]-
515.2787414	515.2781	0.0006	1.1644	ST 21:1;O2;Hex	C27H44O7	[M+Cl]-
515.2787414	515.2781	0.0006	1.1644	ST 27:2;O7	C27H44O7	[M+Cl]-
515.2787414	515.2779	0.0008	1.5526	LPA O-22:5	C25H43O6P	[M+Formate]-
515.2787414	515.2779	0.0008	1.5526	LPA O-21:5	C24H41O6P	[M+OAc]-
516.2821655	516.2814	0.0007	1.3558	CAR 17:3;O4	C24H41NO8	[M+Formate]-
516.2821655	516.2814	0.0007	1.3558	ST 22:0;O7;G	C24H41NO8	[M+Formate]-
516.2821655	516.2814	0.0007	1.3558	CAR 16:3;O4	C23H39NO8	[M+OAc]-
516.2821655	516.2814	0.0007	1.3558	ST 21:0;O7;G	C23H39NO8	[M+OAc]-
517.1311762	517.1305	0.0007	1.3536	ST 23:5;O6;S	C23H30O9S	[M+Cl]-
529.2942844	529.2936	0.0007	1.3225	LPA 24:4;O	C27H47O8P	[M-H]-
529.2942844	529.2936	0.0007	1.3225	LPG O-21:5	C27H47O8P	[M-H]-
529.2942844	529.2936	0.0007	1.3225	PA 24:3	C27H47O8P	[M-H]-
529.2942844	529.2936	0.0007	1.3225	PA O-24:4;O	C27H47O8P	[M-H]-
529.2942844	529.2938	0.0005	0.9447	DG 25:4;O2	C28H46O7	[M+Cl]-
529.2942844	529.2938	0.0005	0.9447	ST 22:0;O;GlcA	C28H46O7	[M+Cl]-
529.2942844	529.2938	0.0005	0.9447	ST 22:1;O2;Hex	C28H46O7	[M+Cl]-
529.2942844	529.2938	0.0005	0.9447	ST 28:2;O7	C28H46O7	[M+Cl]-
529.2942844	529.2936	0.0007	1.3225	LPA O-23:5	C26H45O6P	[M+Formate]-
529.2942844	529.2936	0.0007	1.3225	LPA O-22:5	C25H43O6P	[M+OAc]-
531.1396586	531.1404	0.0007	1.3179	LPI 11:3	C20H33O12P	[M+Cl]-
531.141322	531.1404	0.0009	1.6945	LPI 11:3	C20H33O12P	[M+Cl]-
533.1263203	533.1254	0.0009	1.6882	ST 23:5;O7;S	C23H30O10S	[M+Cl]-
535.142007	535.141	0.001	1.8687	ST 23:4;O7;S	C23H32O10S	[M+Cl]-
537.2739011	537.2754	0.0014	2.6057	LPA 23:3	C26H47O7P	[M+Cl]-
537.2739011	537.2754	0.0014	2.6057	LPA O-23:4;O	C26H47O7P	[M+Cl]-
537.2739011	537.2754	0.0014	2.6057	PA O-23:3	C26H47O7P	[M+Cl]-
543.3106003	543.3092	0.0014	2.5768	LPA 25:4;O	C28H49O8P	[M-H]-
543.3106003	543.3092	0.0014	2.5768	LPG O-22:5	C28H49O8P	[M-H]-
543.3106003	543.3092	0.0014	2.5768	PA 25:3	C28H49O8P	[M-H]-
543.3106003	543.3092	0.0014	2.5768	PA O-25:4;O	C28H49O8P	[M-H]-
543.3106003	543.3094	0.0012	2.2087	DG 26:4;O2	C29H48O7	[M+Cl]-
543.3106003	543.3094	0.0012	2.2087	ST 23:0;O;GlcA	C29H48O7	[M+Cl]-

543.3106003	543.3094	0.0012	2.2087	ST 23:1;O2;Hex	C29H48O7	[M+Cl]-
543.3106003	543.3094	0.0012	2.2087	ST 29:2;O7	C29H48O7	[M+Cl]-
543.3106003	543.3092	0.0014	2.5768	LPA O-24:5	C27H47O6P	[M+Formate]-
543.3106003	543.3092	0.0014	2.5768	LPA O-23:5	C26H45O6P	[M+OAc]-
543.3106003	543.3092	0.0014	2.5768	LPA 25:4;O	C28H49O8P	[M-H]-
543.3106003	543.3092	0.0014	2.5768	LPG O-22:5	C28H49O8P	[M-H]-
543.3106003	543.3092	0.0014	2.5768	PA 25:3	C28H49O8P	[M-H]-
543.3106003	543.3092	0.0014	2.5768	PA O-25:4;O	C28H49O8P	[M-H]-
543.3106003	543.3094	0.0012	2.2087	DG 26:4;O2	C29H48O7	[M+Cl]-
543.3106003	543.3094	0.0012	2.2087	ST 23:0;O;GlcA	C29H48O7	[M+Cl]-
543.3106003	543.3094	0.0012	2.2087	ST 23:1;O2;Hex	C29H48O7	[M+Cl]-
543.3106003	543.3094	0.0012	2.2087	ST 29:2;O7	C29H48O7	[M+Cl]-
543.3106003	543.3092	0.0014	2.5768	LPA O-24:5	C27H47O6P	[M+Formate]-
543.3106003	543.3092	0.0014	2.5768	LPA O-23:5	C26H45O6P	[M+OAc]-
553.2692686	553.2703	0.001	1.8074	LPA 23:3;O	C26H47O8P	[M+Cl]-
553.2692686	553.2703	0.001	1.8074	LPG O-20:4	C26H47O8P	[M+Cl]-
553.2692686	553.2703	0.001	1.8074	PA 23:2	C26H47O8P	[M+Cl]-
553.2692686	553.2703	0.001	1.8074	PA O-23:3;O	C26H47O8P	[M+Cl]-
553.2790725	553.2783	0.0007	1.2652	BMP 19:1;O	C25H47O11P	[M-H]-
553.2790725	553.2783	0.0007	1.2652	LPI O-16:2	C25H47O11P	[M-H]-
553.2790725	553.2783	0.0007	1.2652	LPG 18:2	C24H45O9P	[M+Formate]-
553.2790725	553.2783	0.0007	1.2652	LPG O-18:3;O	C24H45O9P	[M+Formate]-
553.2790725	553.2783	0.0007	1.2652	PA 21:1;O	C24H45O9P	[M+Formate]-
553.2790725	553.2783	0.0007	1.2652	LPG 17:2	C23H43O9P	[M+OAc]-
553.2790725	553.2783	0.0007	1.2652	LPG O-17:3;O	C23H43O9P	[M+OAc]-
553.2790725	553.2783	0.0007	1.2652	PA 20:1;O	C23H43O9P	[M+OAc]-
553.2790725	553.2783	0.0007	1.2652	BMP 19:1;O	C25H47O11P	[M-H]-
553.2790725	553.2783	0.0007	1.2652	LPI O-16:2	C25H47O11P	[M-H]-
553.2790725	553.2783	0.0007	1.2652	LPG 18:2	C24H45O9P	[M+Formate]-
553.2790725	553.2783	0.0007	1.2652	LPG O-18:3;O	C24H45O9P	[M+Formate]-
553.2790725	553.2783	0.0007	1.2652	PA 21:1;O	C24H45O9P	[M+Formate]-
553.2790725	553.2783	0.0007	1.2652	LPG 17:2	C23H43O9P	[M+OAc]-
553.2790725	553.2783	0.0007	1.2652	LPG O-17:3;O	C23H43O9P	[M+OAc]-
553.2790725	553.2783	0.0007	1.2652	PA 20:1;O	C23H43O9P	[M+OAc]-
557.3259241	557.3249	0.001	1.7943	LPA 26:4;O	C29H51O8P	[M-H]-
557.3259241	557.3249	0.001	1.7943	LPG O-23:5	C29H51O8P	[M-H]-
557.3259241	557.3249	0.001	1.7943	PA 26:3	C29H51O8P	[M-H]-
557.3259241	557.3249	0.001	1.7943	PA O-26:4;O	C29H51O8P	[M-H]-

557.3259241	557.3251	0.0009	1.6149	DG 27:4;O2	C30H50O7	[M+Cl]-
557.3259241	557.3251	0.0009	1.6149	ST 24:0;O;GlcA	C30H50O7	[M+Cl]-
557.3259241	557.3251	0.0009	1.6149	ST 24:1;O2;Hex	C30H50O7	[M+Cl]-
557.3259241	557.3251	0.0009	1.6149	ST 30:2;O7	C30H50O7	[M+Cl]-
557.3259241	557.3249	0.001	1.7943	LPA O-25:5	C28H49O6P	[M+Formate]-
557.3259241	557.3249	0.001	1.7943	LPA O-24:5	C27H47O6P	[M+OAc]-
569.274807	569.2756	0.0008	1.4053	ST 26:6;O3;GlcA	C32H42O9	[M-H]-
569.274807	569.2756	0.0008	1.4053	ST 26:7;O4;Hex	C32H42O9	[M-H]-
569.274807	569.2756	0.0008	1.4053	ST 25:6;O;GlcA	C31H40O7	[M+Formate]-
569.274807	569.2756	0.0008	1.4053	ST 25:7;O2;Hex	C31H40O7	[M+Formate]-
569.274807	569.2756	0.0008	1.4053	ST 24:6;O;GlcA	C30H38O7	[M+OAc]-
571.2901028	571.2889	0.0012	2.1005	LPI 16:0	C25H49O12P	[M-H]-
571.2901028	571.2889	0.0012	2.1005	LPI O-16:1;O	C25H49O12P	[M-H]-
571.2901028	571.2913	0.0012	2.1005	ST 26:5;O3;GlcA	C32H44O9	[M-H]-
571.2901028	571.2913	0.0012	2.1005	ST 26:6;O4;Hex	C32H44O9	[M-H]-
571.2901028	571.2889	0.0012	2.1005	BMP 18:0	C24H47O10P	[M+Formate]-
571.2901028	571.2889	0.0012	2.1005	LPG 18:1;O	C24H47O10P	[M+Formate]-
571.2901028	571.2913	0.0012	2.1005	ST 25:5;O;GlcA	C31H42O7	[M+Formate]-
571.2901028	571.2913	0.0012	2.1005	ST 25:6;O2;Hex	C31H42O7	[M+Formate]-
571.2901028	571.2889	0.0012	2.1005	BMP 17:0	C23H45O10P	[M+OAc]-
571.2901028	571.2889	0.0012	2.1005	LPG 17:1;O	C23H45O10P	[M+OAc]-
571.2901028	571.2913	0.0012	2.1005	ST 24:5;O;GlcA	C30H40O7	[M+OAc]-
571.2901028	571.2913	0.0012	2.1005	ST 24:6;O2;Hex	C30H40O7	[M+OAc]-
571.2901028	571.2913	0.0012	2.1005	ST 30:7;O7	C30H40O7	[M+OAc]-
571.2901028	571.2889	0.0012	2.1005	LPI 16:0	C25H49O12P	[M-H]-
571.2901028	571.2889	0.0012	2.1005	LPI O-16:1;O	C25H49O12P	[M-H]-
571.2901028	571.2913	0.0012	2.1005	ST 26:5;O3;GlcA	C32H44O9	[M-H]-
571.2901028	571.2913	0.0012	2.1005	ST 26:6;O4;Hex	C32H44O9	[M-H]-
571.2901028	571.2889	0.0012	2.1005	BMP 18:0	C24H47O10P	[M+Formate]-
571.2901028	571.2889	0.0012	2.1005	LPG 18:1;O	C24H47O10P	[M+Formate]-
571.2901028	571.2913	0.0012	2.1005	ST 25:5;O;GlcA	C31H42O7	[M+Formate]-
571.2901028	571.2913	0.0012	2.1005	ST 25:6;O2;Hex	C31H42O7	[M+Formate]-
571.2901028	571.2889	0.0012	2.1005	BMP 17:0	C23H45O10P	[M+OAc]-
571.2901028	571.2889	0.0012	2.1005	LPG 17:1;O	C23H45O10P	[M+OAc]-
571.2901028	571.2913	0.0012	2.1005	ST 24:5;O;GlcA	C30H40O7	[M+OAc]-
571.2901028	571.2913	0.0012	2.1005	ST 24:6;O2;Hex	C30H40O7	[M+OAc]-
571.2901028	571.2913	0.0012	2.1005	ST 30:7;O7	C30H40O7	[M+OAc]-
571.3416585	571.3405	0.0011	1.9253	LPA 27:4;O	C30H53O8P	[M-H]-

571.3416585	571.3405	0.0011	1.9253	LPG O-24:5	C30H53O8P	[M-H]-
571.3416585	571.3405	0.0011	1.9253	PA 27:3	C30H53O8P	[M-H]-
571.3416585	571.3405	0.0011	1.9253	PA O-27:4;O	C30H53O8P	[M-H]-
571.3416585	571.3407	0.0009	1.5752	DG 28:4;O2	C31H52O7	[M+Cl]-
571.3416585	571.3407	0.0009	1.5752	ST 25:0;O;GlcA	C31H52O7	[M+Cl]-
571.3416585	571.3407	0.0009	1.5752	ST 25:1;O2;Hex	C31H52O7	[M+Cl]-
571.3416585	571.3405	0.0011	1.9253	LPA O-26:5	C29H51O6P	[M+Formate]-
571.3416585	571.3405	0.0011	1.9253	LPA O-25:5	C28H49O6P	[M+OAc]-
572.2938431	572.2913	0.0025	4.3684	CerP 29:6;O2	C29H48NO6P	[M+Cl]-
572.3452777	572.344	0.0012	2.0966	CAR 21:3;O4	C28H49NO8	[M+Formate]-
572.3452777	572.344	0.0012	2.0966	ST 26:0;O7;G	C28H49NO8	[M+Formate]-
572.3452777	572.344	0.0012	2.0966	CAR 20:3;O4	C27H47NO8	[M+OAc]-
572.3452777	572.344	0.0012	2.0966	ST 25:0;O7;G	C27H47NO8	[M+OAc]-
575.1808179	575.1804	0.0004	0.6954	ST 24:4;O8;S	C24H34O11S	[M+Formate]-
575.1808179	575.1804	0.0004	0.6954	ST 23:4;O8;S	C23H32O11S	[M+OAc]-
575.2522336	575.2532	0.0009	1.5645	ST 27:2;O8;S	C27H44O11S	[M-H]-
575.2522336	575.2532	0.0009	1.5645	ST 26:2;O6;S	C26H42O9S	[M+Formate]-
575.2522336	575.2532	0.0009	1.5645	ST 25:2;O6;S	C25H40O9S	[M+OAc]-
591.1506223	591.1486	0.002	3.3832	ST 19:4;O8;GlcA	C25H32O14	[M+Cl]-
599.373237	599.3718	0.0014	2.3358	LPA 29:4;O	C32H57O8P	[M-H]-
599.373237	599.3718	0.0014	2.3358	LPG O-26:5	C32H57O8P	[M-H]-
599.373237	599.3718	0.0014	2.3358	PA 29:3	C32H57O8P	[M-H]-
599.373237	599.3718	0.0014	2.3358	PA O-29:4;O	C32H57O8P	[M-H]-
599.373237	599.372	0.0012	2.0021	DG 30:4;O2	C33H56O7	[M+Cl]-
599.373237	599.372	0.0012	2.0021	ST 27:0;O;GlcA	C33H56O7	[M+Cl]-
599.373237	599.372	0.0012	2.0021	ST 27:1;O2;Hex	C33H56O7	[M+Cl]-
599.373237	599.372	0.0012	2.0021	TG 30:3;O	C33H56O7	[M+Cl]-
599.373237	599.372	0.0012	2.0021	TG O-30:4;O2	C33H56O7	[M+Cl]-
599.373237	599.3718	0.0014	2.3358	LPA O-28:5	C31H55O6P	[M+Formate]-
599.373237	599.3718	0.0014	2.3358	LPA O-27:5	C30H53O6P	[M+OAc]-
617.4213867	617.4212	0.0002	0.3239	DG 37:10	C40H58O5	[M-H]-
617.4213867	617.4212	0.0002	0.3239	TG O-37:10	C40H58O5	[M-H]-
617.4213867	617.4212	0.0002	0.3239	DG 37:10	C40H58O5	[M-H]-
617.4213867	617.4212	0.0002	0.3239	TG O-37:10	C40H58O5	[M-H]-
619.4361133	619.4368	0.0007	1.1301	DG 37:9	C40H60O5	[M-H]-
619.4361133	619.4368	0.0007	1.1301	DG O-37:10;O	C40H60O5	[M-H]-
619.4361133	619.4368	0.0007	1.1301	TG O-37:9	C40H60O5	[M-H]-
620.4392552	620.4379	0.0013	2.0953	CAR 24:0;O4	C31H61NO8	[M+Formate]-

620.4392552	620.4379	0.0013	2.0953	CAR 23:0;O4	C30H59NO8	[M+OAc]-
620.4407689	620.4379	0.0028	4.5129	CAR 24:0;O4	C31H61NO8	[M+Formate]-
620.4407689	620.4379	0.0028	4.5129	CAR 23:0;O4	C30H59NO8	[M+OAc]-
625.301645	625.3018	0.0002	0.3198	ST 29:7;O4;GlcA	C35H46O10	[M-H]-
625.301645	625.3018	0.0002	0.3198	ST 28:7;O2;GlcA	C34H44O8	[M+Formate]-
625.301645	625.3018	0.0002	0.3198	ST 27:7;O2;GlcA	C33H42O8	[M+OAc]-
625.3896525	625.3875	0.0022	3.5178	LPA 31:5;O	C34H59O8P	[M-H]-
625.3896525	625.3875	0.0022	3.5178	LPG O-28:6	C34H59O8P	[M-H]-
625.3896525	625.3875	0.0022	3.5178	PA 31:4	C34H59O8P	[M-H]-
625.3896525	625.3875	0.0022	3.5178	PA O-31:5;O	C34H59O8P	[M-H]-
625.3896525	625.3877	0.002	3.1980	DG 32:5;O2	C35H58O7	[M+Cl]-
625.3896525	625.3877	0.002	3.1980	ST 29:1;O;GlcA	C35H58O7	[M+Cl]-
625.3896525	625.3877	0.002	3.1980	ST 29:2;O2;Hex	C35H58O7	[M+Cl]-
625.3896525	625.3877	0.002	3.1980	TG 32:4;O	C35H58O7	[M+Cl]-
625.3896525	625.3877	0.002	3.1980	TG O-32:5;O2	C35H58O7	[M+Cl]-
625.3896525	625.3875	0.0022	3.5178	LPA O-30:6	C33H57O6P	[M+Formate]-
625.3896525	625.3875	0.0022	3.5178	LPA O-29:6	C32H55O6P	[M+OAc]-
627.404676	627.4031	0.0015	2.3908	LPA 31:4;O	C34H61O8P	[M-H]-
627.404676	627.4031	0.0015	2.3908	LPG O-28:5	C34H61O8P	[M-H]-
627.404676	627.4031	0.0015	2.3908	PA 31:3	C34H61O8P	[M-H]-
627.404676	627.4031	0.0015	2.3908	PA O-31:4;O	C34H61O8P	[M-H]-
627.404676	627.4033	0.0014	2.2314	DG 32:4;O2	C35H60O7	[M+Cl]-
627.404676	627.4033	0.0014	2.2314	ST 29:0;O;GlcA	C35H60O7	[M+Cl]-
627.404676	627.4033	0.0014	2.2314	ST 29:1;O2;Hex	C35H60O7	[M+Cl]-
627.404676	627.4033	0.0014	2.2314	TG 32:3;O	C35H60O7	[M+Cl]-
627.404676	627.4033	0.0014	2.2314	TG O-32:4;O2	C35H60O7	[M+Cl]-
627.404676	627.4031	0.0015	2.3908	LPA O-30:5	C33H59O6P	[M+Formate]-
627.404676	627.4031	0.0015	2.3908	LPA O-29:5	C32H57O6P	[M+OAc]-
631.4367786	631.4368	0	0.0000	DG 38:10	C41H60O5	[M-H]-
631.4367786	631.4368	0	0.0000	TG O-38:10	C41H60O5	[M-H]-
633.4521849	633.4525	0.0003	0.4736	DG 38:9	C41H62O5	[M-H]-
633.4521849	633.4525	0.0003	0.4736	DG O-38:10;O	C41H62O5	[M-H]-
633.4521849	633.4525	0.0003	0.4736	TG O-38:9	C41H62O5	[M-H]-
633.4521849	633.4501	0.0021	3.3152	LPA 31:1;O	C34H67O8P	[M-H]-
633.4521849	633.4501	0.0021	3.3152	LPG O-28:2	C34H67O8P	[M-H]-
633.4521849	633.4501	0.0021	3.3152	PA 31:0	C34H67O8P	[M-H]-
633.4521849	633.4501	0.0021	3.3152	PA O-31:1;O	C34H67O8P	[M-H]-
633.4521849	633.4503	0.0019	2.9994	DG 32:1;O2	C35H66O7	[M+Cl]-

633.4521849	633.4503	0.0019	2.9994	TG 32:0;O	C35H66O7	[M+Cl]-
633.4521849	633.4503	0.0019	2.9994	TG O-32:1;O2	C35H66O7	[M+Cl]-
633.4521849	633.4501	0.0021	3.3152	LPA O-30:2	C33H65O6P	[M+Formate]-
633.4521849	633.4501	0.0021	3.3152	LPA O-29:2	C32H63O6P	[M+OAc]-
634.4535522	634.4536	0	0.0000	CAR 25:0;O4	C32H63NO8	[M+Formate]-
634.4535522	634.4536	0	0.0000	CAR 24:0;O4	C31H61NO8	[M+OAc]-
637.263916	637.2633	0.0007	1.0984	ST 23:1;O7;GlcA	C29H46O13	[M+Cl]-
637.263916	637.2633	0.0007	1.0984	ST 23:2;O8;Hex	C29H46O13	[M+Cl]-
637.263916	637.2631	0.0008	1.2554	LPI 18:4	C27H45O12P	[M+Formate]-
637.263916	637.2631	0.0008	1.2554	LPI O-18:5;O	C27H45O12P	[M+Formate]-
637.263916	637.2631	0.0008	1.2554	LPI 17:4	C26H43O12P	[M+OAc]-
639.4044036	639.4031	0.0013	2.0331	LPA 32:5;O	C35H61O8P	[M-H]-
639.4044036	639.4031	0.0013	2.0331	LPG O-29:6	C35H61O8P	[M-H]-
639.4044036	639.4031	0.0013	2.0331	PA 32:4	C35H61O8P	[M-H]-
639.4044036	639.4031	0.0013	2.0331	PA O-32:5;O	C35H61O8P	[M-H]-
639.4044036	639.4033	0.0011	1.7204	DG 33:5;O2	C36H60O7	[M+Cl]-
639.4044036	639.4033	0.0011	1.7204	ST 30:1;O;GlcA	C36H60O7	[M+Cl]-
639.4044036	639.4033	0.0011	1.7204	ST 30:2;O2;Hex	C36H60O7	[M+Cl]-
639.4044036	639.4033	0.0011	1.7204	TG 33:4;O	C36H60O7	[M+Cl]-
639.4044036	639.4033	0.0011	1.7204	TG O-33:5;O2	C36H60O7	[M+Cl]-
639.4044036	639.4031	0.0013	2.0331	LPA O-31:6	C34H59O6P	[M+Formate]-
639.4044036	639.4031	0.0013	2.0331	LPA O-30:6	C33H57O6P	[M+OAc]-
641.4209362	641.4188	0.0021	3.2740	LPA 32:4;O	C35H63O8P	[M-H]-
641.4209362	641.4188	0.0021	3.2740	LPG O-29:5	C35H63O8P	[M-H]-
641.4209362	641.4188	0.0021	3.2740	PA 32:3	C35H63O8P	[M-H]-
641.4209362	641.4188	0.0021	3.2740	PA O-32:4;O	C35H63O8P	[M-H]-
641.4209362	641.419	0.002	3.1181	DG 33:4;O2	C36H62O7	[M+Cl]-
641.4209362	641.419	0.002	3.1181	ST 30:0;O;GlcA	C36H62O7	[M+Cl]-
641.4209362	641.419	0.002	3.1181	ST 30:1;O2;Hex	C36H62O7	[M+Cl]-
641.4209362	641.419	0.002	3.1181	TG 33:3;O	C36H62O7	[M+Cl]-
641.4209362	641.419	0.002	3.1181	TG O-33:4;O2	C36H62O7	[M+Cl]-
641.4209362	641.4188	0.0021	3.2740	LPA O-31:5	C34H61O6P	[M+Formate]-
641.4209362	641.4188	0.0021	3.2740	LPA O-30:5	C33H59O6P	[M+OAc]-
643.4362074	643.4368	0.0006	0.9325	DG 39:11	C42H60O5	[M-H]-
643.4362074	643.4368	0.0006	0.9325	TG O-39:11	C42H60O5	[M-H]-
644.4398804	644.4379	0.002	3.1035	HexCer 28:2;O4	C34H63NO10	[M-H]-
644.4398804	644.4379	0.002	3.1035	CAR 26:2;O4	C33H61NO8	[M+Formate]-
644.4398804	644.4379	0.002	3.1035	CAR 25:2;O4	C32H59NO8	[M+OAc]-

645.4517773	645.4525	0.0007	1.0845	DG 39:10	C42H62O5	[M-H]-
645.4517773	645.4525	0.0007	1.0845	DG O-39:11;O	C42H62O5	[M-H]-
645.4517773	645.4525	0.0007	1.0845	TG O-39:10	C42H62O5	[M-H]-
646.4554777	646.4536	0.0019	2.9391	HexCer 28:1;O4	C34H65NO10	[M-H]-
646.4554777	646.4536	0.0019	2.9391	CAR 26:1;O4	C33H63NO8	[M+Formate]-
646.4554777	646.4536	0.0019	2.9391	CAR 25:1;O4	C32H61NO8	[M+OAc]-
651.3171947	651.3183	0.0011	1.6889	EPC 28:5;O5	C30H53N2O9P	[M+Cl]-
652.3233643	652.3256	0.0023	3.5258	LPS 27:7;O	C33H52NO10P	[M-H]-
652.3233643	652.3256	0.0023	3.5258	PS 27:6	C33H52NO10P	[M-H]-
652.3233643	652.3256	0.0023	3.5258	PS O-27:7;O	C33H52NO10P	[M-H]-
652.3233643	652.3258	0.0024	3.6791	ST 26:4;O4;HexNAc	C34H51NO9	[M+Cl]-
652.3233643	652.3256	0.0023	3.5258	PE 27:7	C32H50NO8P	[M+Formate]-
652.3233643	652.3256	0.0023	3.5258	PE 26:7	C31H48NO8P	[M+OAc]-
655.4373002	655.4344	0.0029	4.4245	LPA 33:4;O	C36H65O8P	[M-H]-
655.4373002	655.4344	0.0029	4.4245	LPG O-30:5	C36H65O8P	[M-H]-
655.4373002	655.4344	0.0029	4.4245	PA 33:3	C36H65O8P	[M-H]-
655.4373002	655.4344	0.0029	4.4245	PA O-33:4;O	C36H65O8P	[M-H]-
655.4373002	655.4346	0.0027	4.1194	DG 34:4;O2	C37H64O7	[M+Cl]-
655.4373002	655.4346	0.0027	4.1194	TG 34:3;O	C37H64O7	[M+Cl]-
655.4373002	655.4346	0.0027	4.1194	TG O-34:4;O2	C37H64O7	[M+Cl]-
655.4373002	655.4344	0.0029	4.4245	LPA O-32:5	C35H63O6P	[M+Formate]-
655.4373002	655.4344	0.0029	4.4245	LPA O-31:5	C34H61O6P	[M+OAc]-
660.4711649	660.4692	0.0019	2.8767	HexCer 29:1;O4	C35H67NO10	[M-H]-
660.4711649	660.4692	0.0019	2.8767	CAR 27:1;O4	C34H65NO8	[M+Formate]-
660.4711649	660.4692	0.0019	2.8767	HexCer 28:1;O2	C34H65NO8	[M+Formate]-
660.4711649	660.4692	0.0019	2.8767	CAR 26:1;O4	C33H63NO8	[M+OAc]-
662.3240247	662.323	0.001	1.5098	CerP 32:6;O5	C32H54NO9P	[M+Cl]-
662.3240247	662.323	0.001	1.5098	LPS 26:5	C32H54NO9P	[M+Cl]-
662.3240247	662.323	0.001	1.5098	LPS O-26:6;O	C32H54NO9P	[M+Cl]-
662.3240247	662.323	0.001	1.5098	PC 24:5;O	C32H54NO9P	[M+Cl]-
662.3240247	662.323	0.001	1.5098	PE 27:5;O	C32H54NO9P	[M+Cl]-
662.3240247	662.323	0.001	1.5098	PS O-26:5	C32H54NO9P	[M+Cl]-
662.3240247	662.3216	0.0024	3.6236	ST 28:2;O8;T	C30H51NO10S	[M+Formate]-
662.3240247	662.3216	0.0024	3.6236	ST 27:2;O8;T	C29H49NO10S	[M+OAc]-
665.3313982	665.3308	0.0006	0.9018	LPI 22:3;O	C31H55O13P	[M-H]-
665.3313982	665.3308	0.0006	0.9018	PI 22:2	C31H55O13P	[M-H]-
665.3313982	665.3308	0.0006	0.9018	PI O-22:3;O	C31H55O13P	[M-H]-
665.3313982	665.3309	0.0005	0.7515	ST 26:0;O6;GlcA	C32H54O12	[M+Cl]-

665.3313982	665.3309	0.0005	0.7515	ST 26:1;O7;Hex	C32H54O12	[M+Cl]-
665.3313982	665.3308	0.0006	0.9018	BMP 24:3;O	C30H53O11P	[M+Formate]-
665.3313982	665.3308	0.0006	0.9018	LPI O-21:4	C30H53O11P	[M+Formate]-
665.3313982	665.3308	0.0006	0.9018	PG 24:3;O	C30H53O11P	[M+Formate]-
665.3313982	665.3308	0.0006	0.9018	BMP 23:3;O	C29H51O11P	[M+OAc]-
665.3313982	665.3308	0.0006	0.9018	LPI O-20:4	C29H51O11P	[M+OAc]-
665.3313982	665.3308	0.0006	0.9018	PG 23:3;O	C29H51O11P	[M+OAc]-
666.3016431	666.3049	0.0032	4.8026	PS 27:7;O	C33H50NO11P	[M-H]-
671.4677303	671.4681	0.0004	0.5957	DG 41:11	C44H64O5	[M-H]-
671.4677303	671.4681	0.0004	0.5957	TG O-41:11	C44H64O5	[M-H]-
671.4679745	671.4681	0.0001	0.1489	DG 41:11	C44H64O5	[M-H]-
671.4679745	671.4681	0.0001	0.1489	TG O-41:11	C44H64O5	[M-H]-
672.4712289	672.4692	0.002	2.9741	HexCer 30:2;O4	C36H67NO10	[M-H]-
672.4712289	672.4692	0.002	2.9741	CAR 28:2;O4	C35H65NO8	[M+Formate]-
672.4712289	672.4692	0.002	2.9741	HexCer 29:2;O2	C35H65NO8	[M+Formate]-
672.4712289	672.4692	0.002	2.9741	CAR 27:2;O4	C34H63NO8	[M+OAc]-
672.4712289	672.4692	0.002	2.9741	HexCer 28:2;O2	C34H63NO8	[M+OAc]-
672.4712289	672.4692	0.002	2.9741	HexCer 30:2;O4	C36H67NO10	[M-H]-
672.4712289	672.4692	0.002	2.9741	CAR 28:2;O4	C35H65NO8	[M+Formate]-
672.4712289	672.4692	0.002	2.9741	HexCer 29:2;O2	C35H65NO8	[M+Formate]-
672.4712289	672.4692	0.002	2.9741	CAR 27:2;O4	C34H63NO8	[M+OAc]-
672.4712289	672.4692	0.002	2.9741	HexCer 28:2;O2	C34H63NO8	[M+OAc]-
676.3031057	676.3023	0.0008	1.1829	LPS 26:6;O	C32H52NO10P	[M+Cl]-
676.3031057	676.3023	0.0008	1.1829	PS 26:5	C32H52NO10P	[M+Cl]-
676.3031057	676.3023	0.0008	1.1829	PS O-26:6;O	C32H52NO10P	[M+Cl]-
679.3471256	679.3464	0.0007	1.0304	LPI 23:3;O	C32H57O13P	[M-H]-
679.3471256	679.3464	0.0007	1.0304	PI 23:2	C32H57O13P	[M-H]-
679.3471256	679.3464	0.0007	1.0304	PI O-23:3;O	C32H57O13P	[M-H]-
679.3471256	679.3466	0.0005	0.7360	ST 27:0;O6;GlcA	C33H56O12	[M+Cl]-
679.3471256	679.3466	0.0005	0.7360	ST 27:1;O7;Hex	C33H56O12	[M+Cl]-
679.3471256	679.3464	0.0007	1.0304	BMP 25:3;O	C31H55O11P	[M+Formate]-
679.3471256	679.3464	0.0007	1.0304	LPI O-22:4	C31H55O11P	[M+Formate]-
679.3471256	679.3464	0.0007	1.0304	PG 25:3;O	C31H55O11P	[M+Formate]-
679.3471256	679.3464	0.0007	1.0304	BMP 24:3;O	C30H53O11P	[M+OAc]-
679.3471256	679.3464	0.0007	1.0304	LPI O-21:4	C30H53O11P	[M+OAc]-
679.3471256	679.3464	0.0007	1.0304	PG 24:3;O	C30H53O11P	[M+OAc]-
679.3478133	679.3464	0.0014	2.0608	LPI 23:3;O	C32H57O13P	[M-H]-
679.3478133	679.3464	0.0014	2.0608	PI 23:2	C32H57O13P	[M-H]-

679.3478133	679.3464	0.0014	2.0608	PI O-23:3;O	C32H57O13P	[M-H]-
679.3478133	679.3466	0.0012	1.7664	ST 27:0;O6;GlcA	C33H56O12	[M+Cl]-
679.3478133	679.3466	0.0012	1.7664	ST 27:1;O7;Hex	C33H56O12	[M+Cl]-
679.3478133	679.3496	0.0018	2.6496	EPC 30:5;O5	C32H57N2O9P	[M+Cl]-
679.3478133	679.3464	0.0014	2.0608	BMP 25:3;O	C31H55O11P	[M+Formate]-
679.3478133	679.3464	0.0014	2.0608	LPI O-22:4	C31H55O11P	[M+Formate]-
679.3478133	679.3464	0.0014	2.0608	PG 25:3;O	C31H55O11P	[M+Formate]-
679.3478133	679.3464	0.0014	2.0608	BMP 24:3;O	C30H53O11P	[M+OAc]-
679.3478133	679.3464	0.0014	2.0608	LPI O-21:4	C30H53O11P	[M+OAc]-
679.3478133	679.3464	0.0014	2.0608	PG 24:3;O	C30H53O11P	[M+OAc]-
681.364563	681.3652	0.0007	1.0273	EPC 30:4;O5	C32H59N2O9P	[M+Cl]-
681.364563	681.3644	0.0001	0.1468	TG 35:10;O2	C38H52O8	[M+Formate]-
689.296395	689.2944	0.002	2.9015	PI 23:5;O	C32H51O14P	[M-H]-
689.296395	689.2946	0.0018	2.6114	ST 27:3;O7;GlcA	C33H50O13	[M+Cl]-
689.296395	689.2946	0.0018	2.6114	ST 27:4;O8;Hex	C33H50O13	[M+Cl]-
689.296395	689.2944	0.002	2.9015	LPI 22:6	C31H49O12P	[M+Formate]-
689.296395	689.2944	0.002	2.9015	PI O-22:6	C31H49O12P	[M+Formate]-
689.296395	689.2944	0.002	2.9015	LPI 21:6	C30H47O12P	[M+OAc]-
689.296395	689.2944	0.002	2.9015	PI O-21:6	C30H47O12P	[M+OAc]-
690.5091403	690.5079	0.0012	1.7379	CerP 37:1;O4	C37H74NO8P	[M-H]-
690.5091403	690.5079	0.0012	1.7379	LPC 29:1;O	C37H74NO8P	[M-H]-
690.5091403	690.5079	0.0012	1.7379	LPE 32:1;O	C37H74NO8P	[M-H]-
690.5091403	690.5079	0.0012	1.7379	LPS O-31:1	C37H74NO8P	[M-H]-
690.5091403	690.5079	0.0012	1.7379	PE 32:0	C37H74NO8P	[M-H]-
690.5091403	690.5079	0.0012	1.7379	PE O-32:1;O	C37H74NO8P	[M-H]-
690.5091403	690.5081	0.001	1.4482	CAR 31:1;O3	C38H73NO7	[M+Cl]-
690.5091403	690.5081	0.001	1.4482	Cer 38:2;O6	C38H73NO7	[M+Cl]-
690.5091403	690.5079	0.0012	1.7379	CerP 36:1;O2	C36H72NO6P	[M+Formate]-
690.5091403	690.5079	0.0012	1.7379	LPC O-28:2	C36H72NO6P	[M+Formate]-
690.5091403	690.5079	0.0012	1.7379	LPE O-31:2	C36H72NO6P	[M+Formate]-
690.5091403	690.5079	0.0012	1.7379	CerP 35:1;O2	C35H70NO6P	[M+OAc]-
690.5091403	690.5079	0.0012	1.7379	LPC O-27:2	C35H70NO6P	[M+OAc]-
690.5091403	690.5079	0.0012	1.7379	LPE O-30:2	C35H70NO6P	[M+OAc]-
690.5091403	690.5079	0.0012	1.7379	LPC 30:1;O	C38H76NO8P	[M-CH3]-
690.5091403	690.5079	0.0012	1.7379	PC 30:0	C38H76NO8P	[M-CH3]-
690.5091403	690.5079	0.0012	1.7379	PC O-30:1;O	C38H76NO8P	[M-CH3]-
690.5091403	690.5079	0.0012	1.7379	CerP 37:1;O4	C37H74NO8P	[M-H]-
690.5091403	690.5079	0.0012	1.7379	LPC 29:1;O	C37H74NO8P	[M-H]-

690.5091403	690.5079	0.0012	1.7379	LPE 32:1;O	C37H74NO8P	[M-H]-
690.5091403	690.5079	0.0012	1.7379	LPS O-31:1	C37H74NO8P	[M-H]-
690.5091403	690.5079	0.0012	1.7379	PE 32:0	C37H74NO8P	[M-H]-
690.5091403	690.5079	0.0012	1.7379	PE O-32:1;O	C37H74NO8P	[M-H]-
690.5091403	690.5081	0.001	1.4482	CAR 31:1;O3	C38H73NO7	[M+Cl]-
690.5091403	690.5081	0.001	1.4482	Cer 38:2;O6	C38H73NO7	[M+Cl]-
690.5091403	690.5079	0.0012	1.7379	CerP 36:1;O2	C36H72NO6P	[M+Formate]-
690.5091403	690.5079	0.0012	1.7379	LPC O-28:2	C36H72NO6P	[M+Formate]-
690.5091403	690.5079	0.0012	1.7379	LPE O-31:2	C36H72NO6P	[M+Formate]-
690.5091403	690.5079	0.0012	1.7379	CerP 35:1;O2	C35H70NO6P	[M+OAc]-
690.5091403	690.5079	0.0012	1.7379	LPC O-27:2	C35H70NO6P	[M+OAc]-
690.5091403	690.5079	0.0012	1.7379	LPE O-30:2	C35H70NO6P	[M+OAc]-
690.5091403	690.5079	0.0012	1.7379	LPC 30:1;O	C38H76NO8P	[M-CH3]-
690.5091403	690.5079	0.0012	1.7379	PC 30:0	C38H76NO8P	[M-CH3]-
690.5091403	690.5079	0.0012	1.7379	PC O-30:1;O	C38H76NO8P	[M-CH3]-
691.3132312	691.31	0.0032	4.6289	PI 23:4;O	C32H53O14P	[M-H]-
691.3132312	691.3102	0.003	4.3396	ST 27:2;O7;GlcA	C33H52O13	[M+Cl]-
691.3132312	691.3102	0.003	4.3396	ST 27:3;O8;Hex	C33H52O13	[M+Cl]-
691.3132312	691.31	0.0032	4.6289	LPI 22:5	C31H51O12P	[M+Formate]-
691.3132312	691.31	0.0032	4.6289	LPI O-22:6;O	C31H51O12P	[M+Formate]-
691.3132312	691.31	0.0032	4.6289	PI O-22:5	C31H51O12P	[M+Formate]-
691.3132312	691.31	0.0032	4.6289	LPI 21:5	C30H49O12P	[M+OAc]-
691.3132312	691.31	0.0032	4.6289	LPI O-21:6;O	C30H49O12P	[M+OAc]-
691.3132312	691.31	0.0032	4.6289	PI O-21:5	C30H49O12P	[M+OAc]-
692.3172702	692.3205	0.0033	4.7666	PS 29:8;O	C35H52NO11P	[M-H]-
692.3172702	692.3207	0.0034	4.9110	ST 28:6;O5;HexNAc	C36H51NO10	[M+Cl]-
693.3647088	693.3652	0.0005	0.7211	EPC 31:5;O5	C33H59N2O9P	[M+Cl]-
693.3647088	693.3652	0.0005	0.7211	SM 28:5;O5	C33H59N2O9P	[M+Cl]-
703.4097342	703.4111	0.0014	1.9903	LPA 34:5;O	C37H65O8P	[M+Cl]-
703.4097342	703.4111	0.0014	1.9903	LPG O-31:6	C37H65O8P	[M+Cl]-
703.4097342	703.4111	0.0014	1.9903	PA 34:4	C37H65O8P	[M+Cl]-
703.4097342	703.4111	0.0014	1.9903	PA O-34:5;O	C37H65O8P	[M+Cl]-
703.4097342	703.4111	0.0014	1.9903	LPA 34:5;O	C37H65O8P	[M+Cl]-
703.4097342	703.4111	0.0014	1.9903	LPG O-31:6	C37H65O8P	[M+Cl]-
703.4097342	703.4111	0.0014	1.9903	PA 34:4	C37H65O8P	[M+Cl]-
703.4097342	703.4111	0.0014	1.9903	PA O-34:5;O	C37H65O8P	[M+Cl]-
704.4145869	704.4144	0.0002	0.2839	IPC 29:3;O2	C35H64NO11P	[M-H]-
704.4145869	704.4144	0.0002	0.2839	PS 29:2;O	C35H64NO11P	[M-H]-

704.4145869	704.4146	0	0.0000	HexCer 30:4;O4	C36H63NO10	[M+Cl]-
704.4145869	704.4146	0	0.0000	ST 28:0;O5;HexNAc	C36H63NO10	[M+Cl]-
704.4145869	704.4144	0.0002	0.2839	CerP 34:4;O5	C34H62NO9P	[M+Formate]-
704.4145869	704.4144	0.0002	0.2839	LPS 28:3	C34H62NO9P	[M+Formate]-
704.4145869	704.4144	0.0002	0.2839	LPS O-28:4;O	C34H62NO9P	[M+Formate]-
704.4145869	704.4144	0.0002	0.2839	PC 26:3;O	C34H62NO9P	[M+Formate]-
704.4145869	704.4144	0.0002	0.2839	PE 29:3;O	C34H62NO9P	[M+Formate]-
704.4145869	704.4144	0.0002	0.2839	PS O-28:3	C34H62NO9P	[M+Formate]-
704.4145869	704.4144	0.0002	0.2839	CerP 33:4;O5	C33H60NO9P	[M+OAc]-
704.4145869	704.4144	0.0002	0.2839	LPS 27:3	C33H60NO9P	[M+OAc]-
704.4145869	704.4144	0.0002	0.2839	LPS O-27:4;O	C33H60NO9P	[M+OAc]-
704.4145869	704.4144	0.0002	0.2839	PC 25:3;O	C33H60NO9P	[M+OAc]-
704.4145869	704.4144	0.0002	0.2839	PE 28:3;O	C33H60NO9P	[M+OAc]-
704.4145869	704.4144	0.0002	0.2839	PS O-27:3	C33H60NO9P	[M+OAc]-
704.4145869	704.4144	0.0002	0.2839	IPC 30:3;O2	C36H66NO11P	[M-CH3]-
713.5146338	713.5151	0.0004	0.5606	DG 44:11	C47H70O5	[M-H]-
713.5146338	713.5151	0.0004	0.5606	DG O-44:12;O	C47H70O5	[M-H]-
713.5146338	713.5151	0.0004	0.5606	TG O-44:11	C47H70O5	[M-H]-
714.4356689	714.4352	0.0005	0.6999	CerP 37:5;O6	C37H66NO10P	[M-H]-
714.4356689	714.4352	0.0005	0.6999	LPS 31:4;O	C37H66NO10P	[M-H]-
714.4356689	714.4352	0.0005	0.6999	PS 31:3	C37H66NO10P	[M-H]-
714.4356689	714.4352	0.0005	0.6999	PS O-31:4;O	C37H66NO10P	[M-H]-
714.4356689	714.4353	0.0003	0.4199	HexCer 32:5;O3	C38H65NO9	[M+Cl]-
714.4356689	714.4353	0.0003	0.4199	ST 30:1;O4;HexNAc	C38H65NO9	[M+Cl]-
714.4356689	714.4352	0.0005	0.6999	CerP 36:5;O4	C36H64NO8P	[M+Formate]-
714.4356689	714.4352	0.0005	0.6999	LPC 28:5;O	C36H64NO8P	[M+Formate]-
714.4356689	714.4352	0.0005	0.6999	LPE 31:5;O	C36H64NO8P	[M+Formate]-
714.4356689	714.4352	0.0005	0.6999	LPS O-30:5	C36H64NO8P	[M+Formate]-
714.4356689	714.4352	0.0005	0.6999	PC 28:4	C36H64NO8P	[M+Formate]-
714.4356689	714.4352	0.0005	0.6999	PC O-28:5;O	C36H64NO8P	[M+Formate]-
714.4356689	714.4352	0.0005	0.6999	PE 31:4	C36H64NO8P	[M+Formate]-
714.4356689	714.4352	0.0005	0.6999	PE O-31:5;O	C36H64NO8P	[M+Formate]-
714.4356689	714.4352	0.0005	0.6999	CerP 35:5;O4	C35H62NO8P	[M+OAc]-
714.4356689	714.4352	0.0005	0.6999	LPC 27:5;O	C35H62NO8P	[M+OAc]-
714.4356689	714.4352	0.0005	0.6999	LPE 30:5;O	C35H62NO8P	[M+OAc]-
714.4356689	714.4352	0.0005	0.6999	LPS O-29:5	C35H62NO8P	[M+OAc]-
714.4356689	714.4352	0.0005	0.6999	PC 27:4	C35H62NO8P	[M+OAc]-
714.4356689	714.4352	0.0005	0.6999	PC O-27:5;O	C35H62NO8P	[M+OAc]-

714.4356689	714.4352	0.0005	0.6999	PE 30:4	C35H62NO8P	[M+OAc]-
714.4356689	714.4352	0.0005	0.6999	PE O-30:5;O	C35H62NO8P	[M+OAc]-
714.5183174	714.5162	0.0021	2.9391	HexCer 33:2;O4	C39H73NO10	[M-H]-
714.5183174	714.521	0.0027	3.7788	LPC O-29:0;O	C37H78NO7P	[M+Cl]-
714.5183174	714.521	0.0027	3.7788	LPE O-32:0;O	C37H78NO7P	[M+Cl]-
714.5183174	714.5162	0.0021	2.9391	CAR 31:2;O4	C38H71NO8	[M+Formate]-
714.5183174	714.5162	0.0021	2.9391	HexCer 32:2;O2	C38H71NO8	[M+Formate]-
714.5183174	714.5162	0.0021	2.9391	CAR 30:2;O4	C37H69NO8	[M+OAc]-
714.5183174	714.5162	0.0021	2.9391	HexCer 31:2;O2	C37H69NO8	[M+OAc]-
714.5183829	714.5162	0.0022	3.0790	HexCer 33:2;O4	C39H73NO10	[M-H]-
714.5183829	714.521	0.0026	3.6388	LPC O-29:0;O	C37H78NO7P	[M+Cl]-
714.5183829	714.521	0.0026	3.6388	LPE O-32:0;O	C37H78NO7P	[M+Cl]-
714.5183829	714.5162	0.0022	3.0790	CAR 31:2;O4	C38H71NO8	[M+Formate]-
714.5183829	714.5162	0.0022	3.0790	HexCer 32:2;O2	C38H71NO8	[M+Formate]-
714.5183829	714.5162	0.0022	3.0790	CAR 30:2;O4	C37H69NO8	[M+OAc]-
714.5183829	714.5162	0.0022	3.0790	HexCer 31:2;O2	C37H69NO8	[M+OAc]-
721.3849806	721.3853	0.0003	0.4159	BMP 30:4	C36H63O10P	[M+Cl]-
721.3849806	721.3853	0.0003	0.4159	LPG 30:5;O	C36H63O10P	[M+Cl]-
721.3849806	721.3853	0.0003	0.4159	PG 30:4	C36H63O10P	[M+Cl]-
721.3849806	721.3853	0.0003	0.4159	PG O-30:5;O	C36H63O10P	[M+Cl]-
721.3972778	721.3965	0.0007	0.9703	EPC 33:5;O5	C35H63N2O9P	[M+Cl]-
721.3972778	721.3965	0.0007	0.9703	SM 30:5;O5	C35H63N2O9P	[M+Cl]-
721.3972778	721.4006	0.0033	4.5744	PA O-37:9	C40H63O7P	[M+Cl]-
723.4121774	723.4122	0	0.0000	EPC 33:4;O5	C35H65N2O9P	[M+Cl]-
723.4121774	723.4122	0	0.0000	SM 30:4;O5	C35H65N2O9P	[M+Cl]-
725.5146533	725.5151	0.0004	0.5513	DG 45:12	C48H70O5	[M-H]-
725.5146533	725.5151	0.0004	0.5513	TG O-45:12	C48H70O5	[M-H]-
726.5183283	726.5162	0.0021	2.8905	HexCer 34:3;O4	C40H73NO10	[M-H]-
726.5183283	726.521	0.0027	3.7163	CerP 38:0;O3	C38H78NO7P	[M+Cl]-
726.5183283	726.521	0.0027	3.7163	LPC 30:0	C38H78NO7P	[M+Cl]-
726.5183283	726.521	0.0027	3.7163	LPC O-30:1;O	C38H78NO7P	[M+Cl]-
726.5183283	726.521	0.0027	3.7163	LPE 33:0	C38H78NO7P	[M+Cl]-
726.5183283	726.521	0.0027	3.7163	LPE O-33:1;O	C38H78NO7P	[M+Cl]-
726.5183283	726.521	0.0027	3.7163	PC O-30:0	C38H78NO7P	[M+Cl]-
726.5183283	726.521	0.0027	3.7163	PE O-33:0	C38H78NO7P	[M+Cl]-
726.5183283	726.5162	0.0021	2.8905	CAR 32:3;O4	C39H71NO8	[M+Formate]-
726.5183283	726.5162	0.0021	2.8905	HexCer 33:3;O2	C39H71NO8	[M+Formate]-
726.5183283	726.5162	0.0021	2.8905	CAR 31:3;O4	C38H69NO8	[M+OAc]-

726.5183283	726.5162	0.0021	2.8905	HexCer 32:3;O2	C38H69NO8	[M+OAc]-
726.5184153	726.5162	0.0022	3.0281	HexCer 34:3;O4	C40H73NO10	[M-H]-
726.5184153	726.521	0.0026	3.5787	CerP 38:0;O3	C38H78NO7P	[M+Cl]-
726.5184153	726.521	0.0026	3.5787	LPC 30:0	C38H78NO7P	[M+Cl]-
726.5184153	726.521	0.0026	3.5787	LPC O-30:1;O	C38H78NO7P	[M+Cl]-
726.5184153	726.521	0.0026	3.5787	LPE 33:0	C38H78NO7P	[M+Cl]-
726.5184153	726.521	0.0026	3.5787	LPE O-33:1;O	C38H78NO7P	[M+Cl]-
726.5184153	726.521	0.0026	3.5787	PC O-30:0	C38H78NO7P	[M+Cl]-
726.5184153	726.521	0.0026	3.5787	PE O-33:0	C38H78NO7P	[M+Cl]-
726.5184153	726.5162	0.0022	3.0281	CAR 32:3;O4	C39H71NO8	[M+Formate]-
726.5184153	726.5162	0.0022	3.0281	HexCer 33:3;O2	C39H71NO8	[M+Formate]-
726.5184153	726.5162	0.0022	3.0281	CAR 31:3;O4	C38H69NO8	[M+OAc]-
726.5184153	726.5162	0.0022	3.0281	HexCer 32:3;O2	C38H69NO8	[M+OAc]-
730.4312027	730.4301	0.0011	1.5060	IPC 31:4;O2	C37H66NO11P	[M-H]-
730.4312027	730.4301	0.0011	1.5060	PS 31:3;O	C37H66NO11P	[M-H]-
730.4312027	730.4303	0.0009	1.2322	HexCer 32:5;O4	C38H65NO10	[M+Cl]-
730.4312027	730.4303	0.0009	1.2322	ST 30:1;O5;HexNAc	C38H65NO10	[M+Cl]-
730.4312027	730.4301	0.0011	1.5060	CerP 36:5;O5	C36H64NO9P	[M+Formate]-
730.4312027	730.4301	0.0011	1.5060	LPS 30:4	C36H64NO9P	[M+Formate]-
730.4312027	730.4301	0.0011	1.5060	LPS O-30:5;O	C36H64NO9P	[M+Formate]-
730.4312027	730.4301	0.0011	1.5060	PC 28:4;O	C36H64NO9P	[M+Formate]-
730.4312027	730.4301	0.0011	1.5060	PE 31:4;O	C36H64NO9P	[M+Formate]-
730.4312027	730.4301	0.0011	1.5060	PS O-30:4	C36H64NO9P	[M+Formate]-
730.4312027	730.4301	0.0011	1.5060	CerP 35:5;O5	C35H62NO9P	[M+OAc]-
730.4312027	730.4301	0.0011	1.5060	LPS 29:4	C35H62NO9P	[M+OAc]-
730.4312027	730.4301	0.0011	1.5060	LPS O-29:5;O	C35H62NO9P	[M+OAc]-
730.4312027	730.4301	0.0011	1.5060	PC 27:4;O	C35H62NO9P	[M+OAc]-
730.4312027	730.4301	0.0011	1.5060	PE 30:4;O	C35H62NO9P	[M+OAc]-
730.4312027	730.4301	0.0011	1.5060	PS O-29:4	C35H62NO9P	[M+OAc]-
730.4312027	730.4301	0.0011	1.5060	IPC 32:4;O2	C38H68NO11P	[M-CH3]-
735.4122263	735.4122	0	0.0000	EPC 34:5;O5	C36H65N2O9P	[M+Cl]-
735.4122263	735.4122	0	0.0000	SM 31:5;O5	C36H65N2O9P	[M+Cl]-
735.4122263	735.4114	0.0008	1.0878	TG 39:11;O2	C42H58O8	[M+Formate]-
740.5021388	740.5003	0.0019	2.5658	CerP 38:1;O4	C38H76NO8P	[M+Cl]-
740.5021388	740.5003	0.0019	2.5658	LPC 30:1;O	C38H76NO8P	[M+Cl]-
740.5021388	740.5003	0.0019	2.5658	LPE 33:1;O	C38H76NO8P	[M+Cl]-
740.5021388	740.5003	0.0019	2.5658	LPS O-32:1	C38H76NO8P	[M+Cl]-
740.5021388	740.5003	0.0019	2.5658	PC 30:0	C38H76NO8P	[M+Cl]-

740.5021388	740.5003	0.0019	2.5658	PC O-30:1;O	C38H76NO8P	[M+Cl]-
740.5021388	740.5003	0.0019	2.5658	PE 33:0	C38H76NO8P	[M+Cl]-
740.5021388	740.5003	0.0019	2.5658	PE O-33:1;O	C38H76NO8P	[M+Cl]-
741.5072753	741.5076	0.0003	0.4046	PA 38:3;O	C41H75O9P	[M-H]-
741.5072753	741.5076	0.0003	0.4046	PG O-35:4	C41H75O9P	[M-H]-
741.5072753	741.5078	0.0005	0.6743	TG 39:3;O2	C42H74O8	[M+Cl]-
741.5072753	741.5078	0.0005	0.6743	TG O-39:4;O3	C42H74O8	[M+Cl]-
741.5072753	741.5076	0.0003	0.4046	PA O-37:4	C40H73O7P	[M+Formate]-
741.5072753	741.5076	0.0003	0.4046	PA O-36:4	C39H71O7P	[M+OAc]-
741.50867	741.5076	0.0011	1.4835	PA 38:3;O	C41H75O9P	[M-H]-
741.50867	741.5076	0.0011	1.4835	PG O-35:4	C41H75O9P	[M-H]-
741.50867	741.51	0.0013	1.7532	DG 45:12;O	C48H70O6	[M-H]-
741.50867	741.51	0.0013	1.7532	TG 45:11	C48H70O6	[M-H]-
741.50867	741.51	0.0013	1.7532	TG O-45:12;O	C48H70O6	[M-H]-
741.50867	741.5078	0.0009	1.2137	TG 39:3;O2	C42H74O8	[M+Cl]-
741.50867	741.5078	0.0009	1.2137	TG O-39:4;O3	C42H74O8	[M+Cl]-
741.50867	741.5076	0.0011	1.4835	PA O-37:4	C40H73O7P	[M+Formate]-
741.50867	741.5076	0.0011	1.4835	PA O-36:4	C39H71O7P	[M+OAc]-
742.4982245	742.4948	0.0034	4.5792	CerP 41:5;O2	C41H74NO6P	[M+Cl]-
742.4982245	742.4948	0.0034	4.5792	LPC O-33:6	C41H74NO6P	[M+Cl]-
743.5033186	743.5021	0.0012	1.6140	PA O-41:8	C44H73O7P	[M-H]-
743.5033186	743.5023	0.001	1.3450	CE 18:4;O4	C45H72O6	[M+Cl]-
743.5033186	743.5023	0.001	1.3450	DG 42:8;O	C45H72O6	[M+Cl]-
743.5033186	743.5023	0.001	1.3450	DG O-42:9;O2	C45H72O6	[M+Cl]-
743.5033186	743.5023	0.001	1.3450	TG 42:7	C45H72O6	[M+Cl]-
743.5033186	743.5023	0.001	1.3450	TG O-42:8;O	C45H72O6	[M+Cl]-
749.4257866	749.4247	0.0011	1.4678	LPI 28:3;O	C37H67O13P	[M-H]-
749.4257866	749.4247	0.0011	1.4678	PI 28:2	C37H67O13P	[M-H]-
749.4257866	749.4247	0.0011	1.4678	PI O-28:3;O	C37H67O13P	[M-H]-
749.4257866	749.4247	0.0011	1.4678	BMP 30:3;O	C36H65O11P	[M+Formate]-
749.4257866	749.4247	0.0011	1.4678	LPI O-27:4	C36H65O11P	[M+Formate]-
749.4257866	749.4247	0.0011	1.4678	PG 30:3;O	C36H65O11P	[M+Formate]-
749.4257866	749.427	0.0012	1.6012	TG 40:11;O2	C43H60O8	[M+Formate]-
749.4257866	749.4247	0.0011	1.4678	BMP 29:3;O	C35H63O11P	[M+OAc]-
749.4257866	749.4247	0.0011	1.4678	LPI O-26:4	C35H63O11P	[M+OAc]-
749.4257866	749.4247	0.0011	1.4678	PG 29:3;O	C35H63O11P	[M+OAc]-
749.4257866	749.427	0.0012	1.6012	TG 39:11;O2	C42H58O8	[M+OAc]-
750.5316162	750.5291	0.0025	3.3310	CerP 39:1;O6	C39H78NO10P	[M-H]-

750.5316162	750.5291	0.0025	3.3310	LPS 33:0;O	C39H78NO10P	[M-H]-
750.5316162	750.5291	0.0025	3.3310	PS O-33:0;O	C39H78NO10P	[M-H]-
750.5316162	750.5292	0.0024	3.1977	HexCer 34:1;O3	C40H77NO9	[M+Cl]-
750.5316162	750.5291	0.0025	3.3310	CerP 38:1;O4	C38H76NO8P	[M+Formate]-
750.5316162	750.5291	0.0025	3.3310	LPC 30:1;O	C38H76NO8P	[M+Formate]-
750.5316162	750.5291	0.0025	3.3310	LPE 33:1;O	C38H76NO8P	[M+Formate]-
750.5316162	750.5291	0.0025	3.3310	LPS O-32:1	C38H76NO8P	[M+Formate]-
750.5316162	750.5291	0.0025	3.3310	PC 30:0	C38H76NO8P	[M+Formate]-
750.5316162	750.5291	0.0025	3.3310	PC O-30:1;O	C38H76NO8P	[M+Formate]-
750.5316162	750.5291	0.0025	3.3310	PE 33:0	C38H76NO8P	[M+Formate]-
750.5316162	750.5291	0.0025	3.3310	PE O-33:1;O	C38H76NO8P	[M+Formate]-
750.5316162	750.5291	0.0025	3.3310	CerP 37:1;O4	C37H74NO8P	[M+OAc]-
750.5316162	750.5291	0.0025	3.3310	LPC 29:1;O	C37H74NO8P	[M+OAc]-
750.5316162	750.5291	0.0025	3.3310	LPE 32:1;O	C37H74NO8P	[M+OAc]-
750.5316162	750.5291	0.0025	3.3310	LPS O-31:1	C37H74NO8P	[M+OAc]-
750.5316162	750.5291	0.0025	3.3310	PC 29:0	C37H74NO8P	[M+OAc]-
750.5316162	750.5291	0.0025	3.3310	PC O-29:1;O	C37H74NO8P	[M+OAc]-
750.5316162	750.5291	0.0025	3.3310	PE 32:0	C37H74NO8P	[M+OAc]-
750.5316162	750.5291	0.0025	3.3310	PE O-32:1;O	C37H74NO8P	[M+OAc]-
753.5478516	753.5464	0.0015	1.9906	DG 47:12	C50H74O5	[M-H]-
753.5478516	753.5464	0.0015	1.9906	DG O-47:13;O	C50H74O5	[M-H]-
753.5478516	753.5464	0.0015	1.9906	TG O-47:12	C50H74O5	[M-H]-
755.4645841	755.4657	0.0012	1.5884	PA 41:9	C44H69O8P	[M-H]-
755.4645841	755.4657	0.0012	1.5884	PA O-41:10;O	C44H69O8P	[M-H]-
755.4645841	755.4635	0.001	1.3237	BMP 32:1	C38H73O10P	[M+Cl]-
755.4645841	755.4635	0.001	1.3237	LPG 32:2;O	C38H73O10P	[M+Cl]-
755.4645841	755.4635	0.001	1.3237	PG 32:1	C38H73O10P	[M+Cl]-
755.4645841	755.4635	0.001	1.3237	PG O-32:2;O	C38H73O10P	[M+Cl]-
755.4645841	755.4659	0.0013	1.7208	DG 42:10;O2	C45H68O7	[M+Cl]-
755.4645841	755.4659	0.0013	1.7208	TG 42:9;O	C45H68O7	[M+Cl]-
755.4645841	755.4659	0.0013	1.7208	TG O-42:10;O2	C45H68O7	[M+Cl]-
759.1939855	759.1955	0.0015	1.9758	PIP 21:6	C30H46O16P2	[M+Cl]-
761.2097631	761.2112	0.0014	1.8392	PIP 21:5	C30H48O16P2	[M+Cl]-
761.2099789	761.2112	0.0012	1.5764	PIP 21:5	C30H48O16P2	[M+Cl]-
763.4450684	763.4435	0.0016	2.0958	EPC 36:5;O5	C38H69N2O9P	[M+Cl]-
763.4450684	763.4435	0.0016	2.0958	SM 33:5;O5	C38H69N2O9P	[M+Cl]-
765.4123286	765.4115	0.0008	1.0452	BMP 32:4;O	C38H67O11P	[M+Cl]-
765.4123286	765.4115	0.0008	1.0452	LPI O-29:5	C38H67O11P	[M+Cl]-

765.4123286	765.4115	0.0008	1.0452	PG 32:4;O	C38H67O11P	[M+Cl]-
765.4123286	765.4139	0.0015	1.9597	TG 42:12;O2	C45H62O8	[M+Cl]-
765.4841952	765.4843	0.0001	0.1306	LPG 34:3	C40H75O9P	[M+Cl]-
765.4841952	765.4843	0.0001	0.1306	LPG O-34:4;O	C40H75O9P	[M+Cl]-
765.4841952	765.4843	0.0001	0.1306	PA 37:2;O	C40H75O9P	[M+Cl]-
765.4841952	765.4843	0.0001	0.1306	PG O-34:3	C40H75O9P	[M+Cl]-
767.5208392	767.5192	0.0016	2.0846	EPC 35:1;O5	C37H75N2O9P	[M+Formate]-
767.5208392	767.5192	0.0016	2.0846	SM 32:1;O5	C37H75N2O9P	[M+Formate]-
767.5208392	767.5192	0.0016	2.0846	EPC 34:1;O5	C36H73N2O9P	[M+OAc]-
767.5208392	767.5192	0.0016	2.0846	SM 31:1;O5	C36H73N2O9P	[M+OAc]-
767.5208392	767.5192	0.0016	2.0846	EPC 35:1;O5	C37H75N2O9P	[M+Formate]-
767.5208392	767.5192	0.0016	2.0846	SM 32:1;O5	C37H75N2O9P	[M+Formate]-
767.5208392	767.5192	0.0016	2.0846	EPC 34:1;O5	C36H73N2O9P	[M+OAc]-
767.5208392	767.5192	0.0016	2.0846	SM 31:1;O5	C36H73N2O9P	[M+OAc]-
769.4853152	769.4873	0.0019	2.4692	LPI 29:0;O	C38H75O13P	[M-H]-
769.4853152	769.4873	0.0019	2.4692	PI O-29:0;O	C38H75O13P	[M-H]-
769.4853152	769.4873	0.0019	2.4692	BMP 31:0;O	C37H73O11P	[M+Formate]-
769.4853152	769.4873	0.0019	2.4692	LPI O-28:1	C37H73O11P	[M+Formate]-
769.4853152	769.4873	0.0019	2.4692	PG 31:0;O	C37H73O11P	[M+Formate]-
769.4853152	769.4873	0.0019	2.4692	BMP 30:0;O	C36H71O11P	[M+OAc]-
769.4853152	769.4873	0.0019	2.4692	LPI O-27:1	C36H71O11P	[M+OAc]-
769.4853152	769.4873	0.0019	2.4692	PG 30:0;O	C36H71O11P	[M+OAc]-
775.1890692	775.1904	0.0014	1.8060	PIP 21:6;O	C30H46O17P2	[M+Cl]-
775.189333	775.1904	0.0011	1.4190	PIP 21:6;O	C30H46O17P2	[M+Cl]-
775.5513417	775.5518	0.0005	0.6447	DG 46:10;O2	C49H76O7	[M-H]-
775.5513417	775.5518	0.0005	0.6447	TG 46:9;O	C49H76O7	[M-H]-
775.5513417	775.5518	0.0005	0.6447	TG O-46:10;O2	C49H76O7	[M-H]-
775.5513417	775.5518	0.0005	0.6447	CE 21:6;O3	C48H74O5	[M+Formate]-
775.5513417	775.5518	0.0005	0.6447	DG 45:10	C48H74O5	[M+Formate]-
775.5513417	775.5518	0.0005	0.6447	DG O-45:11;O	C48H74O5	[M+Formate]-
775.5513417	775.5518	0.0005	0.6447	TG O-45:10	C48H74O5	[M+Formate]-
775.5513417	775.5518	0.0005	0.6447	DG 44:10	C47H72O5	[M+OAc]-
775.5513417	775.5518	0.0005	0.6447	DG O-44:11;O	C47H72O5	[M+OAc]-
775.5513417	775.5518	0.0005	0.6447	TG O-44:10	C47H72O5	[M+OAc]-
777.2047931	777.2061	0.0013	1.6727	PIP 21:5;O	C30H48O17P2	[M+Cl]-
777.4587402	777.4591	0.0004	0.5145	EPC 37:5;O5	C39H71N2O9P	[M+Cl]-
777.4587402	777.4591	0.0004	0.5145	SM 34:5;O5	C39H71N2O9P	[M+Cl]-
777.4587402	777.4583	0.0004	0.5145	TG 42:11;O2	C45H64O8	[M+Formate]-

777.4587402	777.4583	0.0004	0.5145	TG O-42:12;O3	C45H64O8	[M+Formate]-
777.4587402	777.4583	0.0004	0.5145	TG 41:11;O2	C44H62O8	[M+OAc]-
778.5265695	778.524	0.0026	3.3397	IPC 34:1;O2	C40H78NO11P	[M-H]-
778.5265695	778.524	0.0026	3.3397	PS 34:0;O	C40H78NO11P	[M-H]-
778.5265695	778.5242	0.0024	3.0828	HexCer 35:2;O4	C41H77NO10	[M+Cl]-
778.5265695	778.524	0.0026	3.3397	CerP 39:2;O5	C39H76NO9P	[M+Formate]-
778.5265695	778.524	0.0026	3.3397	LPS 33:1	C39H76NO9P	[M+Formate]-
778.5265695	778.524	0.0026	3.3397	LPS O-33:2;O	C39H76NO9P	[M+Formate]-
778.5265695	778.524	0.0026	3.3397	PC 31:1;O	C39H76NO9P	[M+Formate]-
778.5265695	778.524	0.0026	3.3397	PE 34:1;O	C39H76NO9P	[M+Formate]-
778.5265695	778.524	0.0026	3.3397	PS O-33:1	C39H76NO9P	[M+Formate]-
778.5265695	778.524	0.0026	3.3397	CerP 38:2;O5	C38H74NO9P	[M+OAc]-
778.5265695	778.524	0.0026	3.3397	LPS 32:1	C38H74NO9P	[M+OAc]-
778.5265695	778.524	0.0026	3.3397	LPS O-32:2;O	C38H74NO9P	[M+OAc]-
778.5265695	778.524	0.0026	3.3397	PC 30:1;O	C38H74NO9P	[M+OAc]-
778.5265695	778.524	0.0026	3.3397	PE 33:1;O	C38H74NO9P	[M+OAc]-
778.5265695	778.524	0.0026	3.3397	PS O-32:1	C38H74NO9P	[M+OAc]-
778.5265695	778.524	0.0026	3.3397	IPC 35:1;O2	C41H80NO11P	[M-CH3]-
779.2211885	779.2217	0.0006	0.7700	PIP 21:4;O	C30H50O17P2	[M+Cl]-
779.5289484	779.5315	0.0025	3.2071	TG 40:4;O3	C43H74O9	[M+Formate]-
779.5289484	779.5315	0.0025	3.2071	TG 39:4;O3	C42H72O9	[M+OAc]-
779.531095	779.5315	0.0004	0.5131	TG 40:4;O3	C43H74O9	[M+Formate]-
779.531095	779.5315	0.0004	0.5131	TG 39:4;O3	C42H72O9	[M+OAc]-
780.4679972	780.4669	0.0011	1.4094	IPC 32:2;O4	C38H72NO13P	[M-H]-
780.4679972	780.467	0.001	1.2813	HexCer 33:3;O6	C39H71NO12	[M+Cl]-
780.4679972	780.4669	0.0011	1.4094	IPC 31:2;O2	C37H70NO11P	[M+Formate]-
780.4679972	780.4669	0.0011	1.4094	PS 31:1;O	C37H70NO11P	[M+Formate]-
780.4679972	780.4669	0.0011	1.4094	IPC 30:2;O2	C36H68NO11P	[M+OAc]-
780.4679972	780.4669	0.0011	1.4094	PS 30:1;O	C36H68NO11P	[M+OAc]-
780.4679972	780.4669	0.0011	1.4094	IPC 33:2;O4	C39H74NO13P	[M-CH3]-
780.5327352	780.5316	0.0012	1.5374	CerP 41:2;O4	C41H80NO8P	[M+Cl]-
780.5327352	780.5316	0.0012	1.5374	LPC 33:2;O	C41H80NO8P	[M+Cl]-
780.5327352	780.5316	0.0012	1.5374	PC 33:1	C41H80NO8P	[M+Cl]-
780.5327352	780.5316	0.0012	1.5374	PC O-33:2;O	C41H80NO8P	[M+Cl]-
780.5327352	780.5316	0.0012	1.5374	PE 36:1	C41H80NO8P	[M+Cl]-
780.5327352	780.5316	0.0012	1.5374	PE O-36:2;O	C41H80NO8P	[M+Cl]-
781.488641	781.4873	0.0014	1.7915	LPI 30:1;O	C39H75O13P	[M-H]-
781.488641	781.4873	0.0014	1.7915	PI 30:0	C39H75O13P	[M-H]-

781.488641	781.4873	0.0014	1.7915	PI O-30:1;O	C39H75O13P	[M-H]-
781.488641	781.4896	0.001	1.2796	TG 42:9;O2	C45H68O8	[M+Formate]-
781.488641	781.4896	0.001	1.2796	TG O-42:10;O3	C45H68O8	[M+Formate]-
781.488641	781.4873	0.0014	1.7915	BMP 32:1;O	C38H73O11P	[M+Formate]-
781.488641	781.4873	0.0014	1.7915	LPI O-29:2	C38H73O11P	[M+Formate]-
781.488641	781.4873	0.0014	1.7915	PG 32:1;O	C38H73O11P	[M+Formate]-
781.488641	781.4896	0.001	1.2796	TG 41:9;O2	C44H66O8	[M+OAc]-
781.488641	781.4896	0.001	1.2796	TG O-41:10;O3	C44H66O8	[M+OAc]-
781.488641	781.4873	0.0014	1.7915	BMP 31:1;O	C37H71O11P	[M+OAc]-
781.488641	781.4873	0.0014	1.7915	LPI O-28:2	C37H71O11P	[M+OAc]-
781.488641	781.4873	0.0014	1.7915	PG 31:1;O	C37H71O11P	[M+OAc]-
781.4912332	781.4904	0.0008	1.0237	EPC 37:3;O5	C39H75N2O9P	[M+Cl]-
781.4912332	781.4904	0.0008	1.0237	SM 34:3;O5	C39H75N2O9P	[M+Cl]-
782.4934627	782.4897	0.0038	4.8563	PC O-35:7	C43H74NO7P	[M+Cl]-
782.4934627	782.4897	0.0038	4.8563	PE O-38:7	C43H74NO7P	[M+Cl]-
783.4961135	783.497	0.0009	1.1487	PA 43:9	C46H73O8P	[M-H]-
783.4961135	783.497	0.0009	1.1487	PA O-43:10;O	C46H73O8P	[M-H]-
783.4961135	783.4972	0.0011	1.4040	DG 44:10;O2	C47H72O7	[M+Cl]-
783.4961135	783.4972	0.0011	1.4040	TG 44:9;O	C47H72O7	[M+Cl]-
783.4961135	783.4972	0.0011	1.4040	TG O-44:10;O2	C47H72O7	[M+Cl]-
783.4961135	783.4948	0.0013	1.6592	BMP 34:1	C40H77O10P	[M+Cl]-
783.4961135	783.4948	0.0013	1.6592	LPG 34:2;O	C40H77O10P	[M+Cl]-
783.4961135	783.4948	0.0013	1.6592	PG 34:1	C40H77O10P	[M+Cl]-
783.4961135	783.4948	0.0013	1.6592	PG O-34:2;O	C40H77O10P	[M+Cl]-
784.4767522	784.477	0.0003	0.3824	IPC 35:5;O2	C41H72NO11P	[M-H]-
784.4767522	784.477	0.0003	0.3824	PS 35:4;O	C41H72NO11P	[M-H]-
784.4767522	784.4772	0.0005	0.6374	HexCer 36:6;O4	C42H71NO10	[M+Cl]-
784.4767522	784.477	0.0003	0.3824	CerP 40:6;O5	C40H70NO9P	[M+Formate]-
784.4767522	784.477	0.0003	0.3824	LPS 34:5	C40H70NO9P	[M+Formate]-
784.4767522	784.477	0.0003	0.3824	LPS O-34:6;O	C40H70NO9P	[M+Formate]-
784.4767522	784.477	0.0003	0.3824	PC 32:5;O	C40H70NO9P	[M+Formate]-
784.4767522	784.477	0.0003	0.3824	PE 35:5;O	C40H70NO9P	[M+Formate]-
784.4767522	784.477	0.0003	0.3824	PS O-34:5	C40H70NO9P	[M+Formate]-
784.4767522	784.477	0.0003	0.3824	CerP 39:6;O5	C39H68NO9P	[M+OAc]-
784.4767522	784.477	0.0003	0.3824	LPS 33:5	C39H68NO9P	[M+OAc]-
784.4767522	784.477	0.0003	0.3824	LPS O-33:6;O	C39H68NO9P	[M+OAc]-
784.4767522	784.477	0.0003	0.3824	PC 31:5;O	C39H68NO9P	[M+OAc]-
784.4767522	784.477	0.0003	0.3824	PE 34:5;O	C39H68NO9P	[M+OAc]-

784.4767522	784.477	0.0003	0.3824	PS O-33:5	C39H68NO9P	[M+OAc]-
784.4767522	784.477	0.0003	0.3824	IPC 36:5;O2	C42H74NO11P	[M-CH3]-
791.1847103	791.1852	0.0005	0.6320	PIP2 20:5	C29H47O19P3	[M-H]-
791.4266186	791.4272	0.0005	0.6318	BMP 34:5;O	C40H69O11P	[M+Cl]-
791.4266186	791.4272	0.0005	0.6318	LPI O-31:6	C40H69O11P	[M+Cl]-
791.4266186	791.4272	0.0005	0.6318	PG 34:5;O	C40H69O11P	[M+Cl]-
791.4720113	791.4716	0.0004	0.5054	LPI 31:3;O	C40H73O13P	[M-H]-
791.4720113	791.4716	0.0004	0.5054	PI 31:2	C40H73O13P	[M-H]-
791.4720113	791.4716	0.0004	0.5054	PI O-31:3;O	C40H73O13P	[M-H]-
791.4720113	791.4716	0.0004	0.5054	BMP 33:3;O	C39H71O11P	[M+Formate]-
791.4720113	791.4716	0.0004	0.5054	LPI O-30:4	C39H71O11P	[M+Formate]-
791.4720113	791.4716	0.0004	0.5054	PG 33:3;O	C39H71O11P	[M+Formate]-
791.4720113	791.4716	0.0004	0.5054	BMP 32:3;O	C38H69O11P	[M+OAc]-
791.4720113	791.4716	0.0004	0.5054	LPI O-29:4	C38H69O11P	[M+OAc]-
791.4720113	791.4716	0.0004	0.5054	PG 32:3;O	C38H69O11P	[M+OAc]-
793.2009041	793.2008	0.0001	0.1261	PIP2 20:4	C29H49O19P3	[M-H]-
793.4811704	793.4814	0.0002	0.2521	PA 44:11	C47H71O8P	[M-H]-
793.4811704	793.4814	0.0002	0.2521	PA O-44:12;O	C47H71O8P	[M-H]-
793.4811704	793.4816	0.0004	0.5041	DG 45:12;O2	C48H70O7	[M+Cl]-
793.4811704	793.4816	0.0004	0.5041	TG 45:11;O	C48H70O7	[M+Cl]-
793.4811704	793.4816	0.0004	0.5041	TG O-45:12;O2	C48H70O7	[M+Cl]-
793.5165922	793.5178	0.0012	1.5123	PA O-45:11	C48H75O7P	[M-H]-
793.5165922	793.5156	0.001	1.2602	PA 39:2;O	C42H79O9P	[M+Cl]-
793.5165922	793.5156	0.001	1.2602	PG O-36:3	C42H79O9P	[M+Cl]-
793.5165922	793.5179	0.0014	1.7643	DG 46:11;O	C49H74O6	[M+Cl]-
793.5165922	793.5179	0.0014	1.7643	DG O-46:12;O2	C49H74O6	[M+Cl]-
793.5165922	793.5179	0.0014	1.7643	TG 46:10	C49H74O6	[M+Cl]-
793.5165922	793.5179	0.0014	1.7643	TG O-46:11;O	C49H74O6	[M+Cl]-
799.4918172	799.492	0.0001	0.1251	PA 43:9;O	C46H73O9P	[M-H]-
799.4918172	799.492	0.0001	0.1251	PG O-40:10	C46H73O9P	[M-H]-
799.4918172	799.4921	0.0003	0.3752	TG 44:9;O2	C47H72O8	[M+Cl]-
799.4918172	799.4921	0.0003	0.3752	TG O-44:10;O3	C47H72O8	[M+Cl]-
799.4918172	799.492	0.0001	0.1251	PA O-42:10	C45H71O7P	[M+Formate]-
799.4918172	799.492	0.0001	0.1251	PA O-41:10	C44H69O7P	[M+OAc]-
806.5575435	806.5553	0.0023	2.8516	IPC 36:1;O2	C42H82NO11P	[M-H]-
806.5575435	806.5553	0.0023	2.8516	PS 36:0;O	C42H82NO11P	[M-H]-
806.5575435	806.5555	0.0021	2.6037	HexCer 37:2;O4	C43H81NO10	[M+Cl]-
806.5575435	806.5553	0.0023	2.8516	CerP 41:2;O5	C41H80NO9P	[M+Formate]-

806.5575435	806.5553	0.0023	2.8516	PC 33:1;O	C41H80NO9P	[M+Formate]-
806.5575435	806.5553	0.0023	2.8516	PE 36:1;O	C41H80NO9P	[M+Formate]-
806.5575435	806.5553	0.0023	2.8516	PS O-35:1	C41H80NO9P	[M+Formate]-
806.5575435	806.5553	0.0023	2.8516	CerP 40:2;O5	C40H78NO9P	[M+OAc]-
806.5575435	806.5553	0.0023	2.8516	LPS 34:1	C40H78NO9P	[M+OAc]-
806.5575435	806.5553	0.0023	2.8516	LPS O-34:2;O	C40H78NO9P	[M+OAc]-
806.5575435	806.5553	0.0023	2.8516	PC 32:1;O	C40H78NO9P	[M+OAc]-
806.5575435	806.5553	0.0023	2.8516	PE 35:1;O	C40H78NO9P	[M+OAc]-
806.5575435	806.5553	0.0023	2.8516	PS O-34:1	C40H78NO9P	[M+OAc]-
806.5575435	806.5553	0.0023	2.8516	IPC 37:1;O2	C43H84NO11P	[M-CH3]-
807.5057632	807.5061	0.0003	0.3715	EPC 39:4;O5	C41H77N2O9P	[M+Cl]-
807.5057632	807.5061	0.0003	0.3715	SM 36:4;O5	C41H77N2O9P	[M+Cl]-
807.5057632	807.5053	0.0005	0.6192	TG 44:10;O2	C47H70O8	[M+Formate]-
807.5057632	807.5053	0.0005	0.6192	TG O-44:11;O3	C47H70O8	[M+Formate]-
807.5057632	807.5053	0.0005	0.6192	TG 43:10;O2	C46H68O8	[M+OAc]-
807.5057632	807.5053	0.0005	0.6192	TG O-43:11;O3	C46H68O8	[M+OAc]-
807.5617855	807.5628	0.001	1.2383	TG 42:4;O3	C45H78O9	[M+Formate]-
807.5617855	807.5628	0.001	1.2383	TG 41:4;O3	C44H76O9	[M+OAc]-
808.5084258	808.5064	0.002	2.4737	Hex2Cer 28:1;O4	C40H75NO15	[M-H]-
808.5112476	808.5134	0.0022	2.7210	PS 38:5	C44H76NO10P	[M-H]-
808.5112476	808.5134	0.0022	2.7210	PS O-38:6;O	C44H76NO10P	[M-H]-
808.5112476	808.5134	0.0022	2.7210	PC 35:6	C43H74NO8P	[M+Formate]-
808.5112476	808.5134	0.0022	2.7210	PC O-35:7;O	C43H74NO8P	[M+Formate]-
808.5112476	808.5134	0.0022	2.7210	PE 38:6	C43H74NO8P	[M+Formate]-
808.5112476	808.5134	0.0022	2.7210	PE O-38:7;O	C43H74NO8P	[M+Formate]-
808.5112476	808.5134	0.0022	2.7210	LPC 34:7;O	C42H72NO8P	[M+OAc]-
808.5112476	808.5134	0.0022	2.7210	PC 34:6	C42H72NO8P	[M+OAc]-
808.5112476	808.5134	0.0022	2.7210	PC O-34:7;O	C42H72NO8P	[M+OAc]-
808.5112476	808.5134	0.0022	2.7210	PE 37:6	C42H72NO8P	[M+OAc]-
808.5112476	808.5134	0.0022	2.7210	PE O-37:7;O	C42H72NO8P	[M+OAc]-
809.5110269	809.5105	0.0005	0.6177	PG 36:2	C42H79O10P	[M+Cl]-
809.5110269	809.5105	0.0005	0.6177	PG O-36:3;O	C42H79O10P	[M+Cl]-
809.5111034	809.5105	0.0006	0.7412	PG 36:2	C42H79O10P	[M+Cl]-
809.5111034	809.5105	0.0006	0.7412	PG O-36:3;O	C42H79O10P	[M+Cl]-
810.5144951	810.5138	0.0007	0.8636	IPC 34:1;O4	C40H78NO13P	[M-H]-
810.5144951	810.514	0.0005	0.6169	HexCer 35:2;O6	C41H77NO12	[M+Cl]-
810.5144951	810.5138	0.0007	0.8636	IPC 33:1;O2	C39H76NO11P	[M+Formate]-
810.5144951	810.5138	0.0007	0.8636	PS 33:0;O	C39H76NO11P	[M+Formate]-

810.5144951	810.5138	0.0007	0.8636	IPC 32:1;O2	C38H74NO11P	[M+OAc]-
810.5144951	810.5138	0.0007	0.8636	PS 32:0;O	C38H74NO11P	[M+OAc]-
810.5144951	810.5138	0.0007	0.8636	IPC 35:1;O4	C41H80NO13P	[M-CH3]-
819.5019783	819.5029	0.0009	1.0982	LPI 33:3;O	C42H77O13P	[M-H]-
819.5019783	819.5029	0.0009	1.0982	PI 33:2	C42H77O13P	[M-H]-
819.5019783	819.5029	0.0009	1.0982	PI O-33:3;O	C42H77O13P	[M-H]-
819.5019783	819.5029	0.0009	1.0982	LPI O-32:4	C41H75O11P	[M+Formate]-
819.5019783	819.5029	0.0009	1.0982	PG 35:3;O	C41H75O11P	[M+Formate]-
819.5019783	819.5029	0.0009	1.0982	BMP 34:3;O	C40H73O11P	[M+OAc]-
819.5019783	819.5029	0.0009	1.0982	LPI O-31:4	C40H73O11P	[M+OAc]-
819.5019783	819.5029	0.0009	1.0982	PG 34:3;O	C40H73O11P	[M+OAc]-
819.5321045	819.5334	0.0013	1.5863	PA O-47:12	C50H77O7P	[M-H]-
819.5321045	819.5312	0.0009	1.0982	PA 41:3;O	C44H81O9P	[M+Cl]-
819.5321045	819.5312	0.0009	1.0982	PG O-38:4	C44H81O9P	[M+Cl]-
819.5321045	819.5336	0.0015	1.8303	DG 48:12;O	C51H76O6	[M+Cl]-
819.5321045	819.5336	0.0015	1.8303	DG O-48:13;O2	C51H76O6	[M+Cl]-
819.5321045	819.5336	0.0015	1.8303	TG 48:11	C51H76O6	[M+Cl]-
819.5321045	819.5336	0.0015	1.8303	TG O-48:12;O	C51H76O6	[M+Cl]-
821.4613647	821.461	0.0003	0.3652	PI O-35:8	C44H71O12P	[M-H]-
821.4613647	821.461	0.0003	0.3652	PG 37:8	C43H69O10P	[M+Formate]-
821.4613647	821.461	0.0003	0.3652	PG O-37:9;O	C43H69O10P	[M+Formate]-
821.4613647	821.461	0.0003	0.3652	PG 36:8	C42H67O10P	[M+OAc]-
821.4613647	821.461	0.0003	0.3652	PG O-36:9;O	C42H67O10P	[M+OAc]-
823.527422	823.5283	0.0009	1.0929	PA 46:10	C49H77O8P	[M-H]-
823.527422	823.5283	0.0009	1.0929	PA O-46:11;O	C49H77O8P	[M-H]-
823.527422	823.5285	0.0011	1.3357	DG 47:11;O2	C50H76O7	[M+Cl]-
823.527422	823.5285	0.0011	1.3357	TG 47:10;O	C50H76O7	[M+Cl]-
823.527422	823.5285	0.0011	1.3357	TG O-47:11;O2	C50H76O7	[M+Cl]-
823.527422	823.5261	0.0013	1.5786	PG 37:2	C43H81O10P	[M+Cl]-
823.527422	823.5261	0.0013	1.5786	PG O-37:3;O	C43H81O10P	[M+Cl]-
825.5065398	825.5076	0.0011	1.3325	PA 45:10;O	C48H75O9P	[M-H]-
825.5065398	825.5076	0.0011	1.3325	PG O-42:11	C48H75O9P	[M-H]-
825.5065398	825.5054	0.0011	1.3325	LPI O-33:3	C42H79O11P	[M+Cl]-
825.5065398	825.5054	0.0011	1.3325	PG 36:2;O	C42H79O11P	[M+Cl]-
825.5065398	825.5078	0.0012	1.4537	TG 46:10;O2	C49H74O8	[M+Cl]-
825.5065398	825.5078	0.0012	1.4537	TG O-46:11;O3	C49H74O8	[M+Cl]-
825.5065398	825.5076	0.0011	1.3325	PA O-44:11	C47H73O7P	[M+Formate]-
825.5065398	825.5076	0.0011	1.3325	PA O-43:11	C46H71O7P	[M+OAc]-

826.5747681	826.5756	0.0009	1.0888	PE O-44:9	C49H82NO7P	[M-H]-
826.5747681	826.5734	0.0013	1.5728	CerP 43:1;O5	C43H86NO9P	[M+Cl]-
826.5747681	826.5734	0.0013	1.5728	PC 35:0;O	C43H86NO9P	[M+Cl]-
826.5747681	826.5734	0.0013	1.5728	PE 38:0;O	C43H86NO9P	[M+Cl]-
826.5747681	826.5734	0.0013	1.5728	PS O-37:0	C43H86NO9P	[M+Cl]-
826.5747681	826.5756	0.0009	1.0888	PC O-42:9	C50H84NO7P	[M-CH3]-
827.1637833	827.1619	0.0019	2.2970	PIP2 20:5	C29H47O19P3	[M+Cl]-
827.5221959	827.5233	0.0011	1.3293	PA 45:9;O	C48H77O9P	[M-H]-
827.5221959	827.5233	0.0011	1.3293	PG O-42:10	C48H77O9P	[M-H]-
827.5221959	827.5211	0.0011	1.3293	LPI O-33:2	C42H81O11P	[M+Cl]-
827.5221959	827.5211	0.0011	1.3293	PG 36:1;O	C42H81O11P	[M+Cl]-
827.5221959	827.5234	0.0012	1.4501	TG 46:9;O2	C49H76O8	[M+Cl]-
827.5221959	827.5234	0.0012	1.4501	TG O-46:10;O3	C49H76O8	[M+Cl]-
827.5221959	827.5233	0.0011	1.3293	PA O-44:10	C47H75O7P	[M+Formate]-
827.5221959	827.5233	0.0011	1.3293	PA O-43:10	C46H73O7P	[M+OAc]-
830.5952675	830.5917	0.0036	4.3343	CerP 45:3;O6	C45H86NO10P	[M-H]-
830.5952675	830.5917	0.0036	4.3343	PS 39:1	C45H86NO10P	[M-H]-
830.5952675	830.5917	0.0036	4.3343	PS O-39:2;O	C45H86NO10P	[M-H]-
830.5952675	830.5918	0.0034	4.0935	HexCer 40:3;O3	C46H85NO9	[M+Cl]-
830.5952675	830.5917	0.0036	4.3343	CerP 44:3;O4	C44H84NO8P	[M+Formate]-
830.5952675	830.5917	0.0036	4.3343	PC 36:2	C44H84NO8P	[M+Formate]-
830.5952675	830.5917	0.0036	4.3343	PC O-36:3;O	C44H84NO8P	[M+Formate]-
830.5952675	830.5917	0.0036	4.3343	PE 39:2	C44H84NO8P	[M+Formate]-
830.5952675	830.5917	0.0036	4.3343	PE O-39:3;O	C44H84NO8P	[M+Formate]-
830.5952675	830.5917	0.0036	4.3343	CerP 43:3;O4	C43H82NO8P	[M+OAc]-
830.5952675	830.5917	0.0036	4.3343	PC 35:2	C43H82NO8P	[M+OAc]-
830.5952675	830.5917	0.0036	4.3343	PC O-35:3;O	C43H82NO8P	[M+OAc]-
830.5952675	830.5917	0.0036	4.3343	PE 38:2	C43H82NO8P	[M+OAc]-
830.5952675	830.5917	0.0036	4.3343	PE O-38:3;O	C43H82NO8P	[M+OAc]-
831.5025059	831.5029	0.0004	0.4811	LPI 34:4;O	C43H77O13P	[M-H]-
831.5025059	831.5029	0.0004	0.4811	PI 34:3	C43H77O13P	[M-H]-
831.5025059	831.5029	0.0004	0.4811	PI O-34:4;O	C43H77O13P	[M-H]-
831.5025059	831.5029	0.0004	0.4811	LPI O-33:5	C42H75O11P	[M+Formate]-
831.5025059	831.5029	0.0004	0.4811	PG 36:4;O	C42H75O11P	[M+Formate]-
831.5025059	831.5029	0.0004	0.4811	LPI O-32:5	C41H73O11P	[M+OAc]-
831.5025059	831.5029	0.0004	0.4811	PG 35:4;O	C41H73O11P	[M+OAc]-
833.2101287	833.2088	0.0013	1.5602	PIP2 20:2	C29H53O19P3	[M+Cl]-
833.5202337	833.5217	0.0015	1.7996	EPC 41:5;O5	C43H79N2O9P	[M+Cl]-

833.5202337	833.5217	0.0015	1.7996	SM 38:5;O5	C43H79N2O9P	[M+Cl]-
833.5202337	833.5209	0.0007	0.8398	TG 46:11;O2	C49H72O8	[M+Formate]-
833.5202337	833.5209	0.0007	0.8398	TG O-46:12;O3	C49H72O8	[M+Formate]-
833.5202337	833.5209	0.0007	0.8398	TG 45:11;O2	C48H70O8	[M+OAc]-
833.5202337	833.5209	0.0007	0.8398	TG O-45:12;O3	C48H70O8	[M+OAc]-
833.5202337	833.5217	0.0015	1.7996	EPC 41:5;O5	C43H79N2O9P	[M+Cl]-
833.5202337	833.5217	0.0015	1.7996	SM 38:5;O5	C43H79N2O9P	[M+Cl]-
833.5202337	833.5209	0.0007	0.8398	TG 46:11;O2	C49H72O8	[M+Formate]-
833.5202337	833.5209	0.0007	0.8398	TG O-46:12;O3	C49H72O8	[M+Formate]-
833.5202337	833.5209	0.0007	0.8398	TG 45:11;O2	C48H70O8	[M+OAc]-
833.5202337	833.5209	0.0007	0.8398	TG O-45:12;O3	C48H70O8	[M+OAc]-
834.4285889	834.4315	0.0029	3.4754	SHexCer 34:5;O6	C40H69NO15S	[M-H]-
834.4285889	834.4315	0.0029	3.4754	SHexCer 33:5;O4	C39H67NO13S	[M+Formate]-
834.4285889	834.4315	0.0029	3.4754	SHexCer 32:5;O4	C38H65NO13S	[M+OAc]-
834.525377	834.5221	0.0033	3.9544	Hex2Cer 30:2;O4	C42H77NO15	[M-H]-
834.525377	834.5291	0.0037	4.4336	PS 40:6	C46H78NO10P	[M-H]-
834.525377	834.5291	0.0037	4.4336	PS O-40:7;O	C46H78NO10P	[M-H]-
834.525377	834.5221	0.0033	3.9544	Hex2Cer 29:2;O2	C41H75NO13	[M+Formate]-
834.525377	834.5291	0.0037	4.4336	PC 37:7	C45H76NO8P	[M+Formate]-
834.525377	834.5291	0.0037	4.4336	PC O-37:8;O	C45H76NO8P	[M+Formate]-
834.525377	834.5291	0.0037	4.4336	PE 40:7	C45H76NO8P	[M+Formate]-
834.525377	834.5291	0.0037	4.4336	PE O-40:8;O	C45H76NO8P	[M+Formate]-
834.525377	834.5221	0.0033	3.9544	Hex2Cer 28:2;O2	C40H73NO13	[M+OAc]-
834.525377	834.5291	0.0037	4.4336	PC 36:7	C44H74NO8P	[M+OAc]-
834.525377	834.5291	0.0037	4.4336	PC O-36:8;O	C44H74NO8P	[M+OAc]-
834.525377	834.5291	0.0037	4.4336	PE 39:7	C44H74NO8P	[M+OAc]-
834.525377	834.5291	0.0037	4.4336	PE O-39:8;O	C44H74NO8P	[M+OAc]-
835.5370483	835.5374	0.0003	0.3591	EPC 41:4;O5	C43H81N2O9P	[M+Cl]-
835.5370483	835.5374	0.0003	0.3591	SM 38:4;O5	C43H81N2O9P	[M+Cl]-
835.5370483	835.5366	0.0005	0.5984	TG 46:10;O2	C49H74O8	[M+Formate]-
835.5370483	835.5366	0.0005	0.5984	TG O-46:11;O3	C49H74O8	[M+Formate]-
835.5370483	835.5366	0.0005	0.5984	TG 45:10;O2	C48H72O8	[M+OAc]-
835.5370483	835.5366	0.0005	0.5984	TG O-45:11;O3	C48H72O8	[M+OAc]-
835.5394897	835.5414	0.0019	2.2740	PA O-45:8	C48H81O7P	[M+Cl]-
835.5394897	835.5374	0.0021	2.5134	EPC 41:4;O5	C43H81N2O9P	[M+Cl]-
835.5394897	835.5374	0.0021	2.5134	SM 38:4;O5	C43H81N2O9P	[M+Cl]-
836.5391192	836.5377	0.0014	1.6736	Hex2Cer 30:1;O4	C42H79NO15	[M-H]-
836.5391192	836.5377	0.0014	1.6736	Hex2Cer 29:1;O2	C41H77NO13	[M+Formate]-

836.5391192	836.5377	0.0014	1.6736	Hex2Cer 28:1;O2	C40H75NO13	[M+OAc]-
836.5412607	836.5447	0.0035	4.1839	PS 40:5	C46H80NO10P	[M-H]-
836.5412607	836.5447	0.0035	4.1839	PS O-40:6;O	C46H80NO10P	[M-H]-
836.5412607	836.5377	0.0036	4.3035	Hex2Cer 30:1;O4	C42H79NO15	[M-H]-
836.5412607	836.5447	0.0035	4.1839	PC 37:6	C45H78NO8P	[M+Formate]-
836.5412607	836.5447	0.0035	4.1839	PC O-37:7;O	C45H78NO8P	[M+Formate]-
836.5412607	836.5447	0.0035	4.1839	PE 40:6	C45H78NO8P	[M+Formate]-
836.5412607	836.5447	0.0035	4.1839	PE O-40:7;O	C45H78NO8P	[M+Formate]-
836.5412607	836.5377	0.0036	4.3035	Hex2Cer 29:1;O2	C41H77NO13	[M+Formate]-
836.5412607	836.5447	0.0035	4.1839	PC 36:6	C44H76NO8P	[M+OAc]-
836.5412607	836.5447	0.0035	4.1839	PC O-36:7;O	C44H76NO8P	[M+OAc]-
836.5412607	836.5447	0.0035	4.1839	PE 39:6	C44H76NO8P	[M+OAc]-
836.5412607	836.5447	0.0035	4.1839	PE O-39:7;O	C44H76NO8P	[M+OAc]-
836.5412607	836.5377	0.0036	4.3035	Hex2Cer 28:1;O2	C40H75NO13	[M+OAc]-
837.5414653	837.5418	0.0003	0.3582	PG 38:2	C44H83O10P	[M+Cl]-
837.5414653	837.5418	0.0003	0.3582	PG O-38:3;O	C44H83O10P	[M+Cl]-
837.5416962	837.5418	0.0001	0.1194	PG 38:2	C44H83O10P	[M+Cl]-
837.5416962	837.5418	0.0001	0.1194	PG O-38:3;O	C44H83O10P	[M+Cl]-
838.545559	838.5451	0.0004	0.4770	IPC 36:1;O4	C42H82NO13P	[M-H]-
838.545559	838.5453	0.0003	0.3578	HexCer 37:2;O6	C43H81NO12	[M+Cl]-
838.545559	838.5451	0.0004	0.4770	IPC 35:1;O2	C41H80NO11P	[M+Formate]-
838.545559	838.5451	0.0004	0.4770	PS 35:0;O	C41H80NO11P	[M+Formate]-
838.545559	838.5451	0.0004	0.4770	IPC 34:1;O2	C40H78NO11P	[M+OAc]-
838.545559	838.5451	0.0004	0.4770	PS 34:0;O	C40H78NO11P	[M+OAc]-
838.545559	838.5451	0.0004	0.4770	IPC 37:1;O4	C43H84NO13P	[M-CH3]-
842.5683598	842.5683	0	0.0000	CerP 43:1;O6	C43H86NO10P	[M+Cl]-
842.5683598	842.5683	0	0.0000	PS O-37:0;O	C43H86NO10P	[M+Cl]-
842.5710677	842.5705	0.0005	0.5934	PE 44:8	C49H82NO8P	[M-H]-
842.5710677	842.5705	0.0005	0.5934	PE O-44:9;O	C49H82NO8P	[M-H]-
842.5710677	842.5705	0.0005	0.5934	PC 42:8	C50H84NO8P	[M-CH3]-
842.5710677	842.5705	0.0005	0.5934	PC O-42:9;O	C50H84NO8P	[M-CH3]-
846.5856323	846.5866	0.0009	1.0631	IPC 39:2;O2	C45H86NO11P	[M-H]-
846.5856323	846.5866	0.0009	1.0631	PS 39:1;O	C45H86NO11P	[M-H]-
846.5856323	846.5868	0.0011	1.2993	HexCer 40:3;O4	C46H85NO10	[M+Cl]-
846.5856323	846.5866	0.0009	1.0631	CerP 44:3;O5	C44H84NO9P	[M+Formate]-
846.5856323	846.5866	0.0009	1.0631	PC 36:2;O	C44H84NO9P	[M+Formate]-
846.5856323	846.5866	0.0009	1.0631	PE 39:2;O	C44H84NO9P	[M+Formate]-
846.5856323	846.5866	0.0009	1.0631	PS O-38:2	C44H84NO9P	[M+Formate]-

846.5856323	846.5866	0.0009	1.0631	CerP 43:3;O5	C43H82NO9P	[M+OAc]-
846.5856323	846.5866	0.0009	1.0631	PC 35:2;O	C43H82NO9P	[M+OAc]-
846.5856323	846.5866	0.0009	1.0631	PE 38:2;O	C43H82NO9P	[M+OAc]-
846.5856323	846.5866	0.0009	1.0631	PS O-37:2	C43H82NO9P	[M+OAc]-
846.5856323	846.5866	0.0009	1.0631	IPC 40:2;O2	C46H88NO11P	[M-CH3]-
847.5367752	847.5374	0.0006	0.7079	EPC 42:5;O5	C44H81N2O9P	[M+Cl]-
847.5367752	847.5374	0.0006	0.7079	SM 39:5;O5	C44H81N2O9P	[M+Cl]-
847.5367752	847.5366	0.0002	0.2360	TG 47:11;O2	C50H74O8	[M+Formate]-
847.5367752	847.5366	0.0002	0.2360	TG O-47:12;O3	C50H74O8	[M+Formate]-
847.5367752	847.5366	0.0002	0.2360	TG 46:11;O2	C49H72O8	[M+OAc]-
847.5367752	847.5366	0.0002	0.2360	TG O-46:12;O3	C49H72O8	[M+OAc]-
849.5499878	849.5499	0.0001	0.1177	PI 35:1	C44H83O13P	[M-H]-
849.5499878	849.5499	0.0001	0.1177	PI O-35:2;O	C44H83O13P	[M-H]-
849.5499878	849.5499	0.0001	0.1177	LPI O-34:3	C43H81O11P	[M+Formate]-
849.5499878	849.5499	0.0001	0.1177	PG 37:2;O	C43H81O11P	[M+Formate]-
849.5499878	849.5499	0.0001	0.1177	LPI O-33:3	C42H79O11P	[M+OAc]-
849.5499878	849.5499	0.0001	0.1177	PG 36:2;O	C42H79O11P	[M+OAc]-
849.5515363	849.553	0.0015	1.7656	EPC 42:4;O5	C44H83N2O9P	[M+Cl]-
849.5515363	849.553	0.0015	1.7656	SM 39:4;O5	C44H83N2O9P	[M+Cl]-
849.5515363	849.5522	0.0007	0.8240	TG 47:10;O2	C50H76O8	[M+Formate]-
849.5515363	849.5522	0.0007	0.8240	TG O-47:11;O3	C50H76O8	[M+Formate]-
849.5515363	849.5522	0.0007	0.8240	TG 46:10;O2	C49H74O8	[M+OAc]-
849.5515363	849.5522	0.0007	0.8240	TG O-46:11;O3	C49H74O8	[M+OAc]-
850.5582004	850.5604	0.0022	2.5865	PS 41:5	C47H82NO10P	[M-H]-
850.5582004	850.5604	0.0022	2.5865	PS O-41:6;O	C47H82NO10P	[M-H]-
850.5582004	850.5604	0.0022	2.5865	PC 38:6	C46H80NO8P	[M+Formate]-
850.5582004	850.5604	0.0022	2.5865	PC O-38:7;O	C46H80NO8P	[M+Formate]-
850.5582004	850.5604	0.0022	2.5865	PE 41:6	C46H80NO8P	[M+Formate]-
850.5582004	850.5604	0.0022	2.5865	PE O-41:7;O	C46H80NO8P	[M+Formate]-
850.5582004	850.5604	0.0022	2.5865	PC 37:6	C45H78NO8P	[M+OAc]-
850.5582004	850.5604	0.0022	2.5865	PC O-37:7;O	C45H78NO8P	[M+OAc]-
850.5582004	850.5604	0.0022	2.5865	PE 40:6	C45H78NO8P	[M+OAc]-
850.5582004	850.5604	0.0022	2.5865	PE O-40:7;O	C45H78NO8P	[M+OAc]-
850.5629524	850.5604	0.0026	3.0568	PS 41:5	C47H82NO10P	[M-H]-
850.5629524	850.5604	0.0026	3.0568	PS O-41:6;O	C47H82NO10P	[M-H]-
850.5629524	850.5604	0.0026	3.0568	PC 38:6	C46H80NO8P	[M+Formate]-
850.5629524	850.5604	0.0026	3.0568	PC O-38:7;O	C46H80NO8P	[M+Formate]-
850.5629524	850.5604	0.0026	3.0568	PE 41:6	C46H80NO8P	[M+Formate]-

850.5629524	850.5604	0.0026	3.0568	PE O-41:7;O	C46H80NO8P	[M+Formate]-
850.5629524	850.5604	0.0026	3.0568	PC 37:6	C45H78NO8P	[M+OAc]-
850.5629524	850.5604	0.0026	3.0568	PC O-37:7;O	C45H78NO8P	[M+OAc]-
850.5629524	850.5604	0.0026	3.0568	PE 40:6	C45H78NO8P	[M+OAc]-
850.5629524	850.5604	0.0026	3.0568	PE O-40:7;O	C45H78NO8P	[M+OAc]-
851.5567597	851.5574	0.0007	0.8220	PG 39:2	C45H85O10P	[M+Cl]-
851.5567597	851.5574	0.0007	0.8220	PG O-39:3;O	C45H85O10P	[M+Cl]-
851.5655126	851.5655	0	0.0000	PI 35:0	C44H85O13P	[M-H]-
851.5655126	851.5655	0	0.0000	PI O-35:1;O	C44H85O13P	[M-H]-
851.5655126	851.5655	0	0.0000	LPI O-34:2	C43H83O11P	[M+Formate]-
851.5655126	851.5655	0	0.0000	PG 37:1;O	C43H83O11P	[M+Formate]-
851.5655126	851.5655	0	0.0000	LPI O-33:2	C42H81O11P	[M+OAc]-
851.5655126	851.5655	0	0.0000	PG 36:1;O	C42H81O11P	[M+OAc]-
853.5370802	853.5367	0.0004	0.4686	PG 38:2;O	C44H83O11P	[M+Cl]-
854.5395211	854.54	0.0005	0.5851	IPC 36:1;O5	C42H82NO14P	[M-H]-
854.5395211	854.5402	0.0007	0.8192	Hex2Cer 31:1;O2	C43H81NO13	[M+Cl]-
854.5395211	854.54	0.0005	0.5851	IPC 35:1;O3	C41H80NO12P	[M+Formate]-
854.5395211	854.54	0.0005	0.5851	IPC 34:1;O3	C40H78NO12P	[M+OAc]-
854.5395211	854.54	0.0005	0.5851	IPC 37:1;O5	C43H84NO14P	[M-CH3]-
855.5536434	855.5546	0.0009	1.0519	PA 47:9;O	C50H81O9P	[M-H]-
855.5536434	855.5546	0.0009	1.0519	PG O-44:10	C50H81O9P	[M-H]-
855.5536434	855.5547	0.0011	1.2857	TG 48:9;O2	C51H80O8	[M+Cl]-
855.5536434	855.5547	0.0011	1.2857	TG O-48:10;O3	C51H80O8	[M+Cl]-
855.5536434	855.5524	0.0013	1.5195	PG 38:1;O	C44H85O11P	[M+Cl]-
855.5536434	855.5546	0.0009	1.0519	PA O-46:10	C49H79O7P	[M+Formate]-
855.5536434	855.5546	0.0009	1.0519	PA O-45:10	C48H77O7P	[M+OAc]-
856.5373151	856.5381	0.0008	0.9340	SHexCer 37:1;O2	C43H83NO11S	[M+Cl]-
857.5208326	857.5209	0.0001	0.1166	TG 48:13;O2	C51H72O8	[M+Formate]-
857.5208326	857.5209	0.0001	0.1166	TG 47:13;O2	C50H70O8	[M+OAc]-
857.5209324	857.5209	0	0.0000	TG 48:13;O2	C51H72O8	[M+Formate]-
857.5209324	857.5209	0	0.0000	TG 47:13;O2	C50H70O8	[M+OAc]-
857.5430908	857.5469	0.0038	4.4312	PA 44:5;O	C47H83O9P	[M+Cl]-
857.5430908	857.5469	0.0038	4.4312	PG O-41:6	C47H83O9P	[M+Cl]-
857.5430908	857.5397	0.0034	3.9648	LPI 32:0;O	C41H81O13P	[M+Formate]-
857.5430908	857.5397	0.0034	3.9648	PI O-32:0;O	C41H81O13P	[M+Formate]-
857.5430908	857.5397	0.0034	3.9648	LPI 31:0;O	C40H79O13P	[M+OAc]-
857.5430908	857.5397	0.0034	3.9648	PI O-31:0;O	C40H79O13P	[M+OAc]-
857.5554469	857.5549	0.0005	0.5831	PI O-37:4	C46H83O12P	[M-H]-

857.5554469	857.5549	0.0005	0.5831	PG 39:4	C45H81O10P	[M+Formate]-
857.5554469	857.5549	0.0005	0.5831	PG O-39:5;O	C45H81O10P	[M+Formate]-
857.5554469	857.5549	0.0005	0.5831	PG 38:4	C44H79O10P	[M+OAc]-
857.5554469	857.5549	0.0005	0.5831	PG O-38:5;O	C44H79O10P	[M+OAc]-
858.5243291	858.5254	0.0011	1.2813	SHexCer 35:0;O6	C41H81NO15S	[M-H]-
858.5243291	858.5254	0.0011	1.2813	SHexCer 34:0;O4	C40H79NO13S	[M+Formate]-
858.5243291	858.5254	0.0011	1.2813	SHexCer 33:0;O4	C39H77NO13S	[M+OAc]-
858.550368	858.5502	0.0002	0.2330	IPC 39:4;O3	C45H82NO12P	[M-H]-
858.550368	858.5504	0	0.0000	HexCer 40:5;O5	C46H81NO11	[M+Cl]-
858.550368	858.5502	0.0002	0.2330	CerP 44:5;O6	C44H80NO10P	[M+Formate]-
858.550368	858.5502	0.0002	0.2330	PS 38:3	C44H80NO10P	[M+Formate]-
858.550368	858.5502	0.0002	0.2330	PS O-38:4;O	C44H80NO10P	[M+Formate]-
858.550368	858.5502	0.0002	0.2330	CerP 43:5;O6	C43H78NO10P	[M+OAc]-
858.550368	858.5502	0.0002	0.2330	PS 37:3	C43H78NO10P	[M+OAc]-
858.550368	858.5502	0.0002	0.2330	PS O-37:4;O	C43H78NO10P	[M+OAc]-
858.550368	858.5502	0.0002	0.2330	IPC 40:4;O3	C46H84NO12P	[M-CH3]-
858.5514328	858.5502	0.0012	1.3977	IPC 39:4;O3	C45H82NO12P	[M-H]-
858.5514328	858.5504	0.0011	1.2812	HexCer 40:5;O5	C46H81NO11	[M+Cl]-
858.5514328	858.5502	0.0012	1.3977	CerP 44:5;O6	C44H80NO10P	[M+Formate]-
858.5514328	858.5502	0.0012	1.3977	PS 38:3	C44H80NO10P	[M+Formate]-
858.5514328	858.5502	0.0012	1.3977	PS O-38:4;O	C44H80NO10P	[M+Formate]-
858.5514328	858.5502	0.0012	1.3977	CerP 43:5;O6	C43H78NO10P	[M+OAc]-
858.5514328	858.5502	0.0012	1.3977	PS 37:3	C43H78NO10P	[M+OAc]-
858.5514328	858.5502	0.0012	1.3977	PS O-37:4;O	C43H78NO10P	[M+OAc]-
858.5514328	858.5502	0.0012	1.3977	IPC 40:4;O3	C46H84NO12P	[M-CH3]-
858.5620728	858.5633	0.0012	1.3977	IPC 37:0;O2	C43H86NO11P	[M+Cl]-
859.5365875	859.5374	0.0008	0.9307	EPC 43:6;O5	C45H81N2O9P	[M+Cl]-
859.5365875	859.5374	0.0008	0.9307	SM 40:6;O5	C45H81N2O9P	[M+Cl]-
859.5365875	859.5366	0	0.0000	TG 48:12;O2	C51H74O8	[M+Formate]-
859.5365875	859.5366	0	0.0000	TG O-48:13;O3	C51H74O8	[M+Formate]-
859.5365875	859.5366	0	0.0000	TG 47:12;O2	C50H72O8	[M+OAc]-
859.5365875	859.5366	0	0.0000	TG O-47:13;O3	C50H72O8	[M+OAc]-
859.5691528	859.5706	0.0014	1.6287	PI O-37:3	C46H85O12P	[M-H]-
859.5691528	859.5706	0.0014	1.6287	PG 39:3	C45H83O10P	[M+Formate]-
859.5691528	859.5706	0.0014	1.6287	PG O-39:4;O	C45H83O10P	[M+Formate]-
859.5691528	859.5706	0.0014	1.6287	PG 38:3	C44H81O10P	[M+OAc]-
859.5691528	859.5706	0.0014	1.6287	PG O-38:4;O	C44H81O10P	[M+OAc]-
860.5399524	860.5377	0.0022	2.5565	Hex2Cer 32:3;O4	C44H79NO15	[M-H]-

860.5399524	860.5425	0.0026	3.0213	IPC 36:0;O3	C42H84NO12P	[M+Cl]-
860.5399524	860.5377	0.0022	2.5565	Hex2Cer 31:3;O2	C43H77NO13	[M+Formate]-
860.5399524	860.5377	0.0022	2.5565	Hex2Cer 30:3;O2	C42H75NO13	[M+OAc]-
860.5401385	860.5377	0.0024	2.7890	Hex2Cer 32:3;O4	C44H79NO15	[M-H]-
860.5401385	860.5425	0.0024	2.7889	IPC 36:0;O3	C42H84NO12P	[M+Cl]-
860.5401385	860.5377	0.0024	2.7890	Hex2Cer 31:3;O2	C43H77NO13	[M+Formate]-
860.5401385	860.5377	0.0024	2.7890	Hex2Cer 30:3;O2	C42H75NO13	[M+OAc]-
861.4537354	861.4536	0.0001	0.1161	PIP 30:0	C39H76O16P2	[M-H]-
861.5518176	861.553	0.0012	1.3928	EPC 43:5;O5	C45H83N2O9P	[M+Cl]-
861.5518176	861.553	0.0012	1.3928	SM 40:5;O5	C45H83N2O9P	[M+Cl]-
861.5518176	861.5522	0.0004	0.4643	TG 48:11;O2	C51H76O8	[M+Formate]-
861.5518176	861.5522	0.0004	0.4643	TG O-48:12;O3	C51H76O8	[M+Formate]-
861.5518176	861.5522	0.0004	0.4643	TG 47:11;O2	C50H74O8	[M+OAc]-
861.5518176	861.5522	0.0004	0.4643	TG O-47:12;O3	C50H74O8	[M+OAc]-
861.5521234	861.553	0.0009	1.0446	EPC 43:5;O5	C45H83N2O9P	[M+Cl]-
861.5521234	861.553	0.0009	1.0446	SM 40:5;O5	C45H83N2O9P	[M+Cl]-
861.5521234	861.5522	0.0001	0.1161	TG 48:11;O2	C51H76O8	[M+Formate]-
861.5521234	861.5522	0.0001	0.1161	TG O-48:12;O3	C51H76O8	[M+Formate]-
861.5521234	861.5522	0.0001	0.1161	TG 47:11;O2	C50H74O8	[M+OAc]-
861.5521234	861.5522	0.0001	0.1161	TG O-47:12;O3	C50H74O8	[M+OAc]-
862.4985962	862.4992	0.0006	0.6957	SHexCer 37:4;O5	C43H77NO14S	[M-H]-
862.4985962	862.4992	0.0006	0.6957	SHexCer 36:4;O3	C42H75NO12S	[M+Formate]-
862.4985962	862.4992	0.0006	0.6957	SHexCer 35:4;O3	C41H73NO12S	[M+OAc]-
862.5556134	862.5534	0.0023	2.6665	Hex2Cer 32:2;O4	C44H81NO15	[M-H]-
862.5556134	862.5534	0.0023	2.6665	Hex2Cer 31:2;O2	C43H79NO13	[M+Formate]-
862.5556134	862.5534	0.0023	2.6665	Hex2Cer 30:2;O2	C42H77NO13	[M+OAc]-
863.5082063	863.508	0.0002	0.2316	PI O-38:8	C47H77O12P	[M-H]-
863.5082063	863.508	0.0002	0.2316	PG 40:8	C46H75O10P	[M+Formate]-
863.5082063	863.508	0.0002	0.2316	PG O-40:9;O	C46H75O10P	[M+Formate]-
863.5082063	863.508	0.0002	0.2316	PG 39:8	C45H73O10P	[M+OAc]-
863.5082063	863.508	0.0002	0.2316	PG O-39:9;O	C45H73O10P	[M+OAc]-
863.5670722	863.5655	0.0016	1.8528	PI 36:1	C45H85O13P	[M-H]-
863.5670722	863.5655	0.0016	1.8528	PI O-36:2;O	C45H85O13P	[M-H]-
863.5670722	863.5687	0.0016	1.8528	EPC 43:4;O5	C45H85N2O9P	[M+Cl]-
863.5670722	863.5687	0.0016	1.8528	SM 40:4;O5	C45H85N2O9P	[M+Cl]-
863.5670722	863.5679	0.0008	0.9264	TG 48:10;O2	C51H78O8	[M+Formate]-
863.5670722	863.5679	0.0008	0.9264	TG O-48:11;O3	C51H78O8	[M+Formate]-
863.5670722	863.5655	0.0016	1.8528	PG 38:2;O	C44H83O11P	[M+Formate]-

863.5670722	863.5679	0.0008	0.9264	TG 47:10;O2	C50H76O8	[M+OAc]-
863.5670722	863.5679	0.0008	0.9264	TG O-47:11;O3	C50H76O8	[M+OAc]-
863.5670722	863.5655	0.0016	1.8528	LPI O-34:3	C43H81O11P	[M+OAc]-
863.5670722	863.5655	0.0016	1.8528	PG 37:2;O	C43H81O11P	[M+OAc]-
863.568287	863.5687	0.0004	0.4632	EPC 43:4;O5	C45H85N2O9P	[M+Cl]-
863.568287	863.5687	0.0004	0.4632	SM 40:4;O5	C45H85N2O9P	[M+Cl]-
863.568287	863.5679	0.0004	0.4632	TG 48:10;O2	C51H78O8	[M+Formate]-
863.568287	863.5679	0.0004	0.4632	TG O-48:11;O3	C51H78O8	[M+Formate]-
863.568287	863.5679	0.0004	0.4632	TG 47:10;O2	C50H76O8	[M+OAc]-
863.568287	863.5679	0.0004	0.4632	TG O-47:11;O3	C50H76O8	[M+OAc]-
864.5708818	864.569	0.0019	2.1976	Hex2Cer 32:1;O4	C44H83NO15	[M-H]-
864.5708818	864.569	0.0019	2.1976	Hex2Cer 31:1;O2	C43H81NO13	[M+Formate]-
864.5708818	864.569	0.0019	2.1976	Hex2Cer 30:1;O2	C42H79NO13	[M+OAc]-
864.5712833	864.569	0.0023	2.6603	Hex2Cer 32:1;O4	C44H83NO15	[M-H]-
864.5712833	864.569	0.0023	2.6603	Hex2Cer 31:1;O2	C43H81NO13	[M+Formate]-
864.5712833	864.569	0.0023	2.6603	Hex2Cer 30:1;O2	C42H79NO13	[M+OAc]-
865.5740234	865.5753	0.0013	1.5019	PA 49:10	C52H83O8P	[M-H]-
865.5740234	865.5753	0.0013	1.5019	PA O-49:11;O	C52H83O8P	[M-H]-
865.5740234	865.5731	0.0009	1.0398	PG 40:2	C46H87O10P	[M+Cl]-
865.5740234	865.5731	0.0009	1.0398	PG O-40:3;O	C46H87O10P	[M+Cl]-
865.5740234	865.5755	0.0014	1.6174	DG 50:11;O2	C53H82O7	[M+Cl]-
865.5740234	865.5755	0.0014	1.6174	TG 50:10;O	C53H82O7	[M+Cl]-
865.5740234	865.5755	0.0014	1.6174	TG O-50:11;O2	C53H82O7	[M+Cl]-
866.5761108	866.5764	0.0003	0.3462	IPC 38:1;O4	C44H86NO13P	[M-H]-
866.5761108	866.5766	0.0005	0.5770	HexCer 39:2;O6	C45H85NO12	[M+Cl]-
866.5761108	866.5764	0.0003	0.3462	IPC 37:1;O2	C43H84NO11P	[M+Formate]-
866.5761108	866.5764	0.0003	0.3462	PS 37:0;O	C43H84NO11P	[M+Formate]-
866.5761108	866.5764	0.0003	0.3462	IPC 36:1;O2	C42H82NO11P	[M+OAc]-
866.5761108	866.5764	0.0003	0.3462	PS 36:0;O	C42H82NO11P	[M+OAc]-
866.5761108	866.5764	0.0003	0.3462	IPC 39:1;O4	C45H88NO13P	[M-CH3]-
871.5598419	871.5625	0.0027	3.0979	PA 45:5;O	C48H85O9P	[M+Cl]-
871.5598419	871.5625	0.0027	3.0979	PG O-42:6	C48H85O9P	[M+Cl]-
871.5598419	871.5625	0.0027	3.0979	PA 45:5;O	C48H85O9P	[M+Cl]-
871.5598419	871.5625	0.0027	3.0979	PG O-42:6	C48H85O9P	[M+Cl]-
872.564685	872.5658	0.0012	1.3753	IPC 40:4;O3	C46H84NO12P	[M-H]-
872.564685	872.566	0.0013	1.4899	HexCer 41:5;O5	C47H83NO11	[M+Cl]-
872.564685	872.5658	0.0012	1.3753	CerP 45:5;O6	C45H82NO10P	[M+Formate]-
872.564685	872.5658	0.0012	1.3753	PS 39:3	C45H82NO10P	[M+Formate]-

872.564685	872.5658	0.0012	1.3753	PS O-39:4;O	C45H82NO10P	[M+Formate]-
872.564685	872.5658	0.0012	1.3753	CerP 44:5;O6	C44H80NO10P	[M+OAc]-
872.564685	872.5658	0.0012	1.3753	PS 38:3	C44H80NO10P	[M+OAc]-
872.564685	872.5658	0.0012	1.3753	PS O-38:4;O	C44H80NO10P	[M+OAc]-
872.564685	872.5658	0.0012	1.3753	IPC 41:4;O3	C47H86NO12P	[M-CH3]-
873.2239478	873.227	0.0031	3.5501	PIP2 25:7;O	C34H53O20P3	[M-H]-
879.553827	879.5546	0.0007	0.7959	PA 49:11;O	C52H81O9P	[M-H]-
879.553827	879.5546	0.0007	0.7959	PG O-46:12	C52H81O9P	[M-H]-
879.553827	879.5547	0.0009	1.0232	TG 50:11;O2	C53H80O8	[M+Cl]-
879.553827	879.5547	0.0009	1.0232	TG O-50:12;O3	C53H80O8	[M+Cl]-
879.553827	879.5524	0.0015	1.7054	PG 40:3;O	C46H85O11P	[M+Cl]-
879.553827	879.5546	0.0007	0.7959	PA O-48:12	C51H79O7P	[M+Formate]-
879.553827	879.5546	0.0007	0.7959	PA O-47:12	C50H77O7P	[M+OAc]-
884.5675514	884.5658	0.0017	1.9218	IPC 41:5;O3	C47H84NO12P	[M-H]-
884.5675514	884.566	0.0015	1.6957	HexCer 42:6;O5	C48H83NO11	[M+Cl]-
884.5675514	884.5694	0.0018	2.0349	SHexCer 39:1;O2	C45H87NO11S	[M+Cl]-
884.5675514	884.5658	0.0017	1.9218	CerP 46:6;O6	C46H82NO10P	[M+Formate]-
884.5675514	884.5658	0.0017	1.9218	PS 40:4	C46H82NO10P	[M+Formate]-
884.5675514	884.5658	0.0017	1.9218	PS O-40:5;O	C46H82NO10P	[M+Formate]-
884.5675514	884.5658	0.0017	1.9218	CerP 45:6;O6	C45H80NO10P	[M+OAc]-
884.5675514	884.5658	0.0017	1.9218	PS 39:4	C45H80NO10P	[M+OAc]-
884.5675514	884.5658	0.0017	1.9218	PS O-39:5;O	C45H80NO10P	[M+OAc]-
884.5675514	884.5658	0.0017	1.9218	IPC 42:5;O3	C48H86NO12P	[M-CH3]-
884.570246	884.5694	0.0009	1.0174	SHexCer 39:1;O2	C45H87NO11S	[M+Cl]-
888.5154042	888.5149	0.0005	0.5627	SHexCer 39:5;O5	C45H79NO14S	[M-H]-
888.5154042	888.5163	0.0009	1.0129	IPC 40:6;O2	C46H80NO11P	[M+Cl]-
888.5154042	888.5163	0.0009	1.0129	PS 40:5;O	C46H80NO11P	[M+Cl]-
888.5154042	888.5149	0.0005	0.5627	SHexCer 38:5;O3	C44H77NO12S	[M+Formate]-
888.5154042	888.5149	0.0005	0.5627	SHexCer 37:5;O3	C43H75NO12S	[M+OAc]-
889.5830982	889.5843	0.0012	1.3489	EPC 45:5;O5	C47H87N2O9P	[M+Cl]-
889.5830982	889.5843	0.0012	1.3489	SM 42:5;O5	C47H87N2O9P	[M+Cl]-
889.5830982	889.5835	0.0004	0.4496	TG 50:11;O2	C53H80O8	[M+Formate]-
889.5830982	889.5835	0.0004	0.4496	TG O-50:12;O3	C53H80O8	[M+Formate]-
889.5830982	889.5835	0.0004	0.4496	TG 49:11;O2	C52H78O8	[M+OAc]-
889.5830982	889.5835	0.0004	0.4496	TG O-49:12;O3	C52H78O8	[M+OAc]-
890.5352427	890.532	0.0033	3.7057	IPC 40:5;O2	C46H82NO11P	[M+Cl]-
890.5352427	890.532	0.0033	3.7057	PS 40:4;O	C46H82NO11P	[M+Cl]-
890.5868026	890.5847	0.0021	2.3580	Hex2Cer 34:2;O4	C46H85NO15	[M-H]-

890.5868026	890.5847	0.0021	2.3580	Hex2Cer 33:2;O2	C45H83NO13	[M+Formate]-
890.5868026	890.5847	0.0021	2.3580	Hex2Cer 32:2;O2	C44H81NO13	[M+OAc]-
890.5877605	890.5847	0.0031	3.4809	Hex2Cer 34:2;O4	C46H85NO15	[M-H]-
890.5877605	890.5917	0.0039	4.3791	PS 44:6	C50H86NO10P	[M-H]-
890.5877605	890.5917	0.0039	4.3791	PS O-44:7;O	C50H86NO10P	[M-H]-
890.5877605	890.5847	0.0031	3.4809	Hex2Cer 33:2;O2	C45H83NO13	[M+Formate]-
890.5877605	890.5917	0.0039	4.3791	PC 41:7	C49H84NO8P	[M+Formate]-
890.5877605	890.5917	0.0039	4.3791	PC O-41:8;O	C49H84NO8P	[M+Formate]-
890.5877605	890.5917	0.0039	4.3791	PE 44:7	C49H84NO8P	[M+Formate]-
890.5877605	890.5917	0.0039	4.3791	PE O-44:8;O	C49H84NO8P	[M+Formate]-
890.5877605	890.5847	0.0031	3.4809	Hex2Cer 32:2;O2	C44H81NO13	[M+OAc]-
890.5877605	890.5917	0.0039	4.3791	PC 40:7	C48H82NO8P	[M+OAc]-
890.5877605	890.5917	0.0039	4.3791	PC O-40:8;O	C48H82NO8P	[M+OAc]-
890.5877605	890.5917	0.0039	4.3791	PE 43:7	C48H82NO8P	[M+OAc]-
890.5877605	890.5917	0.0039	4.3791	PE O-43:8;O	C48H82NO8P	[M+OAc]-
891.4930712	891.4948	0.0018	2.0191	PG 43:10	C49H77O10P	[M+Cl]-
891.4930712	891.4948	0.0018	2.0191	PG O-43:11;O	C49H77O10P	[M+Cl]-
891.5912703	891.5909	0.0003	0.3365	PA 51:11	C54H85O8P	[M-H]-
891.5912703	891.5909	0.0003	0.3365	PA O-51:12;O	C54H85O8P	[M-H]-
891.5912703	891.5911	0.0002	0.2243	DG 52:12;O2	C55H84O7	[M+Cl]-
891.5912703	891.5911	0.0002	0.2243	TG 52:11;O	C55H84O7	[M+Cl]-
891.5912703	891.5911	0.0002	0.2243	TG O-52:12;O2	C55H84O7	[M+Cl]-
893.4943831	893.4952	0.0009	1.0073	PI 36:4	C45H79O13P	[M+Cl]-
893.4943831	893.4952	0.0009	1.0073	PI O-36:5;O	C45H79O13P	[M+Cl]-
893.5067512	893.5105	0.0037	4.1410	PG 43:9	C49H79O10P	[M+Cl]-
893.5067512	893.5105	0.0037	4.1410	PG O-43:10;O	C49H79O10P	[M+Cl]-
893.5067512	893.5033	0.0034	3.8052	PI 34:3;O	C43H77O14P	[M+Formate]-
893.5067512	893.5033	0.0034	3.8052	PI 33:3;O	C42H75O14P	[M+OAc]-
893.5073853	893.5105	0.0031	3.4695	PG 43:9	C49H79O10P	[M+Cl]-
893.5073853	893.5105	0.0031	3.4695	PG O-43:10;O	C49H79O10P	[M+Cl]-
894.4976364	894.4986	0.0009	1.0062	MIPC 31:3;O2	C43H78NO16P	[M-H]-
894.4976364	894.4987	0.0011	1.2297	Hex2Cer 32:4;O4	C44H77NO15	[M+Cl]-
894.4976364	894.4986	0.0009	1.0062	IPC 36:4;O5	C42H76NO14P	[M+Formate]-
894.4976364	894.4986	0.0009	1.0062	IPC 35:4;O5	C41H74NO14P	[M+OAc]-
894.4976364	894.4986	0.0009	1.0062	MIPC 32:3;O2	C44H80NO16P	[M-CH3]-
894.505188	894.5057	0.0006	0.6708	PS 42:8	C48H78NO10P	[M+Cl]-
894.505188	894.5057	0.0006	0.6708	PS O-42:9;O	C48H78NO10P	[M+Cl]-
894.512249	894.5116	0.0006	0.6708	IPC 35:0;O6	C41H82NO15P	[M+Cl]-

895.2219449	895.2245	0.0025	2.7926	PIP2 25:6	C34H55O19P3	[M+Cl]-
897.2370496	897.2401	0.0031	3.4550	PIP2 25:5	C34H57O19P3	[M+Cl]-
897.2392559	897.2401	0.0009	1.0031	PIP2 25:5	C34H57O19P3	[M+Cl]-
897.5747354	897.5782	0.0034	3.7880	PA 47:6;O	C50H87O9P	[M+Cl]-
897.5747354	897.5782	0.0034	3.7880	PG O-44:7	C50H87O9P	[M+Cl]-
897.5747354	897.571	0.0037	4.1222	PI 35:0	C44H85O13P	[M+Formate]-
897.5747354	897.571	0.0037	4.1222	PI O-35:1;O	C44H85O13P	[M+Formate]-
897.5747354	897.571	0.0037	4.1222	LPI 34:1;O	C43H83O13P	[M+OAc]-
897.5747354	897.571	0.0037	4.1222	PI 34:0	C43H83O13P	[M+OAc]-
897.5747354	897.571	0.0037	4.1222	PI O-34:1;O	C43H83O13P	[M+OAc]-
903.5293857	903.5312	0.0018	1.9922	PA 48:10;O	C51H81O9P	[M+Cl]-
903.5293857	903.5312	0.0018	1.9922	PG O-45:11	C51H81O9P	[M+Cl]-
904.5111694	904.5112	0.0001	0.1106	IPC 40:6;O3	C46H80NO12P	[M+Cl]-
907.5237754	907.5261	0.0024	2.6446	PG 44:9	C50H81O10P	[M+Cl]-
907.5237754	907.5261	0.0024	2.6446	PG O-44:10;O	C50H81O10P	[M+Cl]-
908.5263208	908.5273	0.0009	0.9906	IPC 36:0;O6	C42H84NO15P	[M+Cl]-
909.4533955	909.4536	0.0002	0.2199	PIP 34:4	C43H76O16P2	[M-H]-
909.5346292	909.5346	0	0.0000	PI 35:2;O	C44H81O14P	[M+Formate]-
909.5346292	909.5346	0	0.0000	PI 34:2;O	C43H79O14P	[M+OAc]-
911.2160851	911.2194	0.0033	3.6215	PIP2 25:6;O	C34H55O20P3	[M+Cl]-
911.2368038	911.2403	0.0035	3.8409	PIP3 21:0;O	C30H60O23P4	[M-H]-
911.2411245	911.2403	0.0008	0.8779	PIP3 21:0;O	C30H60O23P4	[M-H]-
912.611659	912.6124	0.0007	0.7670	PE 48:9;O	C53H88NO9P	[M-H]-
912.611659	912.6124	0.0007	0.7670	PS O-47:9	C53H88NO9P	[M-H]-
912.611659	912.6102	0.0014	1.5341	IPC 41:1;O2	C47H92NO11P	[M+Cl]-
912.611659	912.6102	0.0014	1.5341	PS 41:0;O	C47H92NO11P	[M+Cl]-
912.611659	912.6124	0.0007	0.7670	PC O-44:10	C52H86NO7P	[M+Formate]-
912.611659	912.6124	0.0007	0.7670	PE O-47:10	C52H86NO7P	[M+Formate]-
912.611659	912.6124	0.0007	0.7670	PC O-43:10	C51H84NO7P	[M+OAc]-
912.611659	912.6124	0.0007	0.7670	PE O-46:10	C51H84NO7P	[M+OAc]-
912.611659	912.6124	0.0007	0.7670	PC 46:9;O	C54H90NO9P	[M-CH3]-
913.232275	913.235	0.0027	2.9565	PIP2 25:5;O	C34H57O20P3	[M+Cl]-
915.2504423	915.2507	0.0002	0.2185	PIP2 25:4;O	C34H59O20P3	[M+Cl]-
915.5031683	915.5029	0.0003	0.3277	PI 41:10	C50H77O13P	[M-H]-
915.5031683	915.5029	0.0003	0.3277	PI O-41:11;O	C50H77O13P	[M-H]-
915.5031683	915.5029	0.0003	0.3277	PG 43:11;O	C49H75O11P	[M+Formate]-
915.5031683	915.5029	0.0003	0.3277	PG 42:11;O	C48H73O11P	[M+OAc]-
917.5087039	917.5105	0.0018	1.9618	PG 45:11	C51H79O10P	[M+Cl]-

917.5087039	917.5105	0.0018	1.9618	PG O-45:12;O	C51H79O10P	[M+Cl]-
917.5093415	917.5105	0.0012	1.3079	PG 45:11	C51H79O10P	[M+Cl]-
917.5093415	917.5105	0.0012	1.3079	PG O-45:12;O	C51H79O10P	[M+Cl]-
918.5132153	918.5138	0.0006	0.6532	PS 42:9;O	C48H76NO11P	[M+Formate]-
918.5132153	918.5138	0.0006	0.6532	PS 41:9;O	C47H74NO11P	[M+OAc]-
919.5160776	919.519	0.0029	3.1538	PI 36:4;O	C45H79O14P	[M+Formate]-
919.5160776	919.519	0.0029	3.1538	PI 35:4;O	C44H77O14P	[M+OAc]-
919.5234222	919.5261	0.0027	2.9363	PG 45:10	C51H81O10P	[M+Cl]-
919.5234222	919.5261	0.0027	2.9363	PG O-45:11;O	C51H81O10P	[M+Cl]-
920.5261055	920.5273	0.0012	1.3036	IPC 37:1;O6	C43H84NO15P	[M+Cl]-
920.527626	920.5273	0.0004	0.4345	IPC 37:1;O6	C43H84NO15P	[M+Cl]-
921.5287476	921.5265	0.0022	2.3873	PI 38:4	C47H83O13P	[M+Cl]-
921.5287476	921.5265	0.0022	2.3873	PI O-38:5;O	C47H83O13P	[M+Cl]-
921.5371767	921.5346	0.0026	2.8214	PI 36:3;O	C45H81O14P	[M+Formate]-
921.5371767	921.5346	0.0026	2.8214	PI 35:3;O	C44H79O14P	[M+OAc]-
922.5355225	922.537	0.0015	1.6260	PS 44:8	C50H82NO10P	[M+Cl]-
922.5355225	922.537	0.0015	1.6260	PS O-44:9;O	C50H82NO10P	[M+Cl]-
922.5355225	922.5334	0.0021	2.2763	SHexCer 37:0;O6	C43H85NO15S	[M+Cl]-
923.5245693	923.5211	0.0035	3.7898	PG 44:9;O	C50H81O11P	[M+Cl]-
927.2329539	927.2352	0.0023	2.4805	PIP3 20:0	C29H58O22P4	[M+Formate]-
929.5411987	929.5397	0.0015	1.6137	PI 38:5	C47H81O13P	[M+Formate]-
929.5411987	929.5397	0.0015	1.6137	PI O-38:6;O	C47H81O13P	[M+Formate]-
929.5411987	929.5397	0.0015	1.6137	PI 37:5	C46H79O13P	[M+OAc]-
929.5411987	929.5397	0.0015	1.6137	PI O-37:6;O	C46H79O13P	[M+OAc]-
931.5586094	931.5625	0.0039	4.1865	PA 50:10;O	C53H85O9P	[M+Cl]-
931.5586094	931.5625	0.0039	4.1865	PG O-47:11	C53H85O9P	[M+Cl]-
931.5586094	931.5553	0.0033	3.5425	PI 38:4	C47H83O13P	[M+Formate]-
931.5586094	931.5553	0.0033	3.5425	PI O-38:5;O	C47H83O13P	[M+Formate]-
931.5586094	931.5553	0.0033	3.5425	PI 37:4	C46H81O13P	[M+OAc]-
931.5586094	931.5553	0.0033	3.5425	PI O-37:5;O	C46H81O13P	[M+OAc]-
931.5600136	931.5625	0.0025	2.6837	PA 50:10;O	C53H85O9P	[M+Cl]-
931.5600136	931.5625	0.0025	2.6837	PG O-47:11	C53H85O9P	[M+Cl]-
932.5440875	932.5425	0.0016	1.7157	IPC 42:6;O3	C48H84NO12P	[M+Cl]-
932.5555842	932.5541	0.0014	1.5013	SHexCer 39:1;O5	C45H87NO14S	[M+Cl]-
932.5555842	932.5578	0.0022	2.3591	PC 44:10;O	C52H84NO9P	[M+Cl]-
932.5555842	932.5578	0.0022	2.3591	PE 47:10;O	C52H84NO9P	[M+Cl]-
932.5555842	932.5578	0.0022	2.3591	PS O-46:10	C52H84NO9P	[M+Cl]-
937.4831543	937.4849	0.0017	1.8134	PIP 36:4	C45H80O16P2	[M-H]-

937.6135745	937.6118	0.0017	1.8131	TG 57:15	C60H86O6	[M+Cl]-
937.6135745	937.6118	0.0017	1.8131	TG O-57:16;O	C60H86O6	[M+Cl]-
938.615515	938.6163	0.0008	0.8523	SHexCer 43:2;O2	C49H93NO11S	[M+Cl]-
938.6267514	938.6281	0.0013	1.3850	PE 50:10;O	C55H90NO9P	[M-H]-
938.6267514	938.6281	0.0013	1.3850	PS O-49:10	C55H90NO9P	[M-H]-
938.6267514	938.6259	0.0009	0.9588	IPC 43:2;O2	C49H94NO11P	[M+Cl]-
938.6267514	938.6259	0.0009	0.9588	PS 43:1;O	C49H94NO11P	[M+Cl]-
938.6267514	938.6281	0.0013	1.3850	PC O-46:11	C54H88NO7P	[M+Formate]-
938.6267514	938.6281	0.0013	1.3850	PE O-49:11	C54H88NO7P	[M+Formate]-
938.6267514	938.6281	0.0013	1.3850	PC O-45:11	C53H86NO7P	[M+OAc]-
938.6267514	938.6281	0.0013	1.3850	PE O-48:11	C53H86NO7P	[M+OAc]-
938.6267514	938.6281	0.0013	1.3850	PC 48:10;O	C56H92NO9P	[M-CH3]-
939.6204975	939.6179	0.0026	2.7671	PI 38:0	C47H91O13P	[M+Formate]-
939.6204975	939.6179	0.0026	2.7671	PI O-38:1;O	C47H91O13P	[M+Formate]-
939.6204975	939.6179	0.0026	2.7671	PI 37:0	C46H89O13P	[M+OAc]-
939.6204975	939.6179	0.0026	2.7671	PI O-37:1;O	C46H89O13P	[M+OAc]-
939.6327515	939.6332	0.0004	0.4257	PI O-43:5	C52H93O12P	[M-H]-
939.6327515	939.6332	0.0004	0.4257	PG 45:5	C51H91O10P	[M+Formate]-
939.6327515	939.6332	0.0004	0.4257	PG O-45:6;O	C51H91O10P	[M+Formate]-
939.6327515	939.6332	0.0004	0.4257	PG 44:5	C50H89O10P	[M+OAc]-
939.6327515	939.6332	0.0004	0.4257	PG O-44:6;O	C50H89O10P	[M+OAc]-
940.4943926	940.496	0.0016	1.7012	IPC 39:5;O6	C45H80NO15P	[M+Cl]-
941.6012202	941.6044	0.0032	3.3985	PG 46:6	C52H91O10P	[M+Cl]-
941.6012202	941.6044	0.0032	3.3985	PG O-46:7;O	C52H91O10P	[M+Cl]-
941.6012202	941.5972	0.004	4.2481	PI 37:0;O	C46H89O14P	[M+Formate]-
941.6012202	941.5972	0.004	4.2481	PI 36:0;O	C45H87O14P	[M+OAc]-
941.6354705	941.6336	0.0019	2.0178	PI O-38:0;O	C47H93O13P	[M+Formate]-
941.6354705	941.6336	0.0019	2.0178	PI O-37:0;O	C46H91O13P	[M+OAc]-
942.5643999	942.5643	0.0001	0.1061	Hex2Cer 32:1;O6	C44H83NO17	[M+Formate]-
942.5643999	942.5643	0.0001	0.1061	Hex2Cer 31:1;O6	C43H81NO17	[M+OAc]-
942.605658	942.6077	0.0021	2.2279	IPC 44:5;O4	C50H90NO13P	[M-H]-
942.605658	942.6079	0.0022	2.3340	HexCer 45:6;O6	C51H89NO12	[M+Cl]-
942.605658	942.6077	0.0021	2.2279	IPC 43:5;O2	C49H88NO11P	[M+Formate]-
942.605658	942.6077	0.0021	2.2279	PS 43:4;O	C49H88NO11P	[M+Formate]-
942.605658	942.6077	0.0021	2.2279	IPC 42:5;O2	C48H86NO11P	[M+OAc]-
942.605658	942.6077	0.0021	2.2279	PS 42:4;O	C48H86NO11P	[M+OAc]-
942.605658	942.6077	0.0021	2.2279	IPC 45:5;O4	C51H92NO13P	[M-CH3]-
943.5335249	943.5342	0.0007	0.7419	PI 43:10	C52H81O13P	[M-H]-

943.5335249	943.5342	0.0007	0.7419	PI O-43:11;O	C52H81O13P	[M-H]-
943.5335249	943.5342	0.0007	0.7419	PG 45:11;O	C51H79O11P	[M+Formate]-
943.5335249	943.5342	0.0007	0.7419	PG 44:11;O	C50H77O11P	[M+OAc]-
944.2413735	944.2437	0.0023	2.4358	CoA 12:2	C33H54N7O17P3S	[M-H]-
944.5796283	944.5789	0.0007	0.7411	IPC 44:6;O2	C50H88NO11P	[M+Cl]-
944.5796283	944.5789	0.0007	0.7411	PS 44:5;O	C50H88NO11P	[M+Cl]-
944.5796283	944.58	0.0003	0.3176	Hex2Cer 32:0;O6	C44H85NO17	[M+Formate]-
944.5796283	944.58	0.0003	0.3176	Hex2Cer 31:0;O6	C43H83NO17	[M+OAc]-
944.5800682	944.58	0.0001	0.1059	Hex2Cer 32:0;O6	C44H85NO17	[M+Formate]-
944.5800682	944.58	0.0001	0.1059	Hex2Cer 31:0;O6	C43H83NO17	[M+OAc]-
945.5384424	945.5418	0.0034	3.5958	PG 47:11	C53H83O10P	[M+Cl]-
945.5384424	945.5418	0.0034	3.5958	PG O-47:12;O	C53H83O10P	[M+Cl]-
945.5384424	945.5346	0.0038	4.0189	PI 38:5;O	C47H81O14P	[M+Formate]-
945.5384424	945.5346	0.0038	4.0189	PI 37:5;O	C46H79O14P	[M+OAc]-
945.5388343	945.5418	0.003	3.1728	PG 47:11	C53H83O10P	[M+Cl]-
945.5388343	945.5418	0.003	3.1728	PG O-47:12;O	C53H83O10P	[M+Cl]-
945.5769971	945.5782	0.0012	1.2691	PA 51:10;O	C54H87O9P	[M+Cl]-
945.5769971	945.5782	0.0012	1.2691	PG O-48:11	C54H87O9P	[M+Cl]-
945.5831238	945.5841	0.0009	0.9518	PI 38:0;O	C47H91O14P	[M+Cl]-
946.5358959	946.537	0.0011	1.1621	PS 46:10	C52H82NO10P	[M+Cl]-
946.5358959	946.537	0.0011	1.1621	PS O-46:11;O	C52H82NO10P	[M+Cl]-
946.5434583	946.5429	0.0005	0.5282	IPC 39:2;O6	C45H86NO15P	[M+Cl]-
947.5901578	947.5866	0.0035	3.6936	PI O-38:4;O	C47H85O13P	[M+OAc]-
947.5911648	947.5938	0.0027	2.8493	PA 51:9;O	C54H89O9P	[M+Cl]-
947.5911648	947.5938	0.0027	2.8493	PG O-48:10	C54H89O9P	[M+Cl]-
948.5938049	948.595	0.0012	1.2650	IPC 40:0;O5	C46H92NO14P	[M+Cl]-
949.5586279	949.5578	0.0008	0.8425	PI 40:4	C49H87O13P	[M+Cl]-
949.5586279	949.5578	0.0008	0.8425	PI O-40:5;O	C49H87O13P	[M+Cl]-
949.6055624	949.6095	0.0039	4.1070	PA 51:8;O	C54H91O9P	[M+Cl]-
949.6055624	949.6095	0.0039	4.1070	PG O-48:9	C54H91O9P	[M+Cl]-
949.6055624	949.6023	0.0033	3.4751	PI 39:2	C48H89O13P	[M+Formate]-
949.6055624	949.6023	0.0033	3.4751	PI O-39:3;O	C48H89O13P	[M+Formate]-
949.6055624	949.6023	0.0033	3.4751	PI 38:2	C47H87O13P	[M+OAc]-
949.6055624	949.6023	0.0033	3.4751	PI O-38:3;O	C47H87O13P	[M+OAc]-
950.2141896	950.2179	0.0037	3.8938	CoA 10:1;O2	C31H52N7O19P3S	[M-H]-
951.621933	951.6251	0.0032	3.3627	PA 51:7;O	C54H93O9P	[M+Cl]-
951.621933	951.6251	0.0032	3.3627	PG O-48:8	C54H93O9P	[M+Cl]-
951.621933	951.6179	0.004	4.2034	PI 39:1	C48H91O13P	[M+Formate]-

951.621933	951.6179	0.004	4.2034	PI O-39:2;O	C48H91O13P	[M+Formate]-
951.621933	951.6179	0.004	4.2034	PI 38:1	C47H89O13P	[M+OAc]-
951.621933	951.6179	0.004	4.2034	PI O-38:2;O	C47H89O13P	[M+OAc]-
951.6220498	951.6251	0.0031	3.2576	PA 51:7;O	C54H93O9P	[M+Cl]-
951.6220498	951.6251	0.0031	3.2576	PG O-48:8	C54H93O9P	[M+Cl]-
953.6380168	953.6408	0.0028	2.9361	PA 51:6;O	C54H95O9P	[M+Cl]-
953.6380168	953.6408	0.0028	2.9361	PG O-48:7	C54H95O9P	[M+Cl]-
955.2215512	955.2221	0.0005	0.5234	PIP3 23:2	C32H60O22P4	[M+Cl]-
955.4995728	955.4978	0.0017	1.7792	PI 43:12;O	C52H77O14P	[M-H]-
957.2354159	957.2377	0.0023	2.4027	PIP3 23:1	C32H62O22P4	[M+Cl]-
957.574483	957.5782	0.0037	3.8639	PA 52:11;O	C55H87O9P	[M+Cl]-
957.574483	957.5782	0.0037	3.8639	PG O-49:12	C55H87O9P	[M+Cl]-
957.574483	957.571	0.0035	3.6551	PI 40:5	C49H85O13P	[M+Formate]-
957.574483	957.571	0.0035	3.6551	PI O-40:6;O	C49H85O13P	[M+Formate]-
957.574483	957.571	0.0035	3.6551	PI 39:5	C48H83O13P	[M+OAc]-
957.574483	957.571	0.0035	3.6551	PI O-39:6;O	C48H83O13P	[M+OAc]-
958.5585867	958.5567	0.0019	1.9821	SHexCer 43:6;O6	C49H85NO15S	[M-H]-
958.5585867	958.5567	0.0019	1.9821	SHexCer 42:6;O4	C48H83NO13S	[M+Formate]-
958.5585867	958.5567	0.0019	1.9821	SHexCer 41:6;O4	C47H81NO13S	[M+OAc]-
958.5599535	958.5567	0.0032	3.3384	SHexCer 43:6;O6	C49H85NO15S	[M-H]-
958.5599535	958.5567	0.0032	3.3384	SHexCer 42:6;O4	C48H83NO13S	[M+Formate]-
958.5599535	958.5567	0.0032	3.3384	SHexCer 41:6;O4	C47H81NO13S	[M+OAc]-
959.5549462	959.5574	0.0025	2.6054	PG 48:11	C54H85O10P	[M+Cl]-
959.5549462	959.5574	0.0025	2.6054	PG O-48:12;O	C54H85O10P	[M+Cl]-
959.5899685	959.5938	0.0039	4.0642	PA 52:10;O	C55H89O9P	[M+Cl]-
959.5899685	959.5938	0.0039	4.0642	PG O-49:11	C55H89O9P	[M+Cl]-
959.5899685	959.5866	0.0033	3.4390	PI 40:4	C49H87O13P	[M+Formate]-
959.5899685	959.5866	0.0033	3.4390	PI O-40:5;O	C49H87O13P	[M+Formate]-
959.5899685	959.5866	0.0033	3.4390	PI 39:4	C48H85O13P	[M+OAc]-
959.5899685	959.5866	0.0033	3.4390	PI O-39:5;O	C48H85O13P	[M+OAc]-
959.5903252	959.5938	0.0035	3.6474	PA 52:10;O	C55H89O9P	[M+Cl]-
959.5903252	959.5938	0.0035	3.6474	PG O-49:11	C55H89O9P	[M+Cl]-
959.5903252	959.5866	0.0037	3.8558	PI 40:4	C49H87O13P	[M+Formate]-
959.5903252	959.5866	0.0037	3.8558	PI O-40:5;O	C49H87O13P	[M+Formate]-
959.5903252	959.5866	0.0037	3.8558	PI 39:4	C48H85O13P	[M+OAc]-
959.5903252	959.5866	0.0037	3.8558	PI O-39:5;O	C48H85O13P	[M+OAc]-
960.5575551	960.5586	0.001	1.0411	IPC 40:2;O6	C46H88NO15P	[M+Cl]-
961.5699248	961.5731	0.0032	3.3279	PG 48:10	C54H87O10P	[M+Cl]-

961.5699248	961.5731	0.0032	3.3279	PG O-48:11;O	C54H87O10P	[M+Cl]-
961.5699248	961.5659	0.004	4.1599	PI 39:4;O	C48H85O14P	[M+Formate]-
961.5699248	961.5659	0.004	4.1599	PI 38:4;O	C47H83O14P	[M+OAc]-
961.5700657	961.5731	0.003	3.1199	PG 48:10	C54H87O10P	[M+Cl]-
961.5700657	961.5731	0.003	3.1199	PG O-48:11;O	C54H87O10P	[M+Cl]-
961.5700657	961.5659	0.0042	4.3679	PI 38:4;O	C47H83O14P	[M+OAc]-
962.5733726	962.5742	0.0008	0.8311	IPC 40:1;O6	C46H90NO15P	[M+Cl]-
963.5778352	963.5735	0.0043	4.4626	PI 41:4	C50H89O13P	[M+Cl]-
963.5778352	963.5735	0.0043	4.4626	PI O-41:5;O	C50H89O13P	[M+Cl]-
963.5778352	963.5816	0.0037	3.8398	PI 39:3;O	C48H87O14P	[M+Formate]-
963.5778352	963.5816	0.0037	3.8398	PI 38:3;O	C47H85O14P	[M+OAc]-
963.5822359	963.5816	0.0007	0.7265	PI 39:3;O	C48H87O14P	[M+Formate]-
963.5822359	963.5816	0.0007	0.7265	PI 38:3;O	C47H85O14P	[M+OAc]-
964.5483394	964.5476	0.0007	0.7257	PS 46:9;O	C52H84NO11P	[M+Cl]-
964.5483394	964.5487	0.0003	0.3110	Hex2Cer 34:4;O6	C46H81NO17	[M+Formate]-
964.5483394	964.5487	0.0003	0.3110	Hex2Cer 33:4;O6	C45H79NO17	[M+OAc]-
964.5823975	964.584	0.0016	1.6587	PS 47:8	C53H88NO10P	[M+Cl]-
964.5823975	964.584	0.0016	1.6587	PS O-47:9;O	C53H88NO10P	[M+Cl]-
964.5823975	964.5804	0.002	2.0734	SHexCer 40:0;O6	C46H91NO15S	[M+Cl]-
965.3842216	965.3835	0.0007	0.7251	PIP2 31:3;O	C40H73O20P3	[M-H]-
966.5619295	966.5633	0.0013	1.3450	PS 46:8;O	C52H86NO11P	[M+Cl]-
967.6158005	967.62	0.0042	4.3405	PG 48:7	C54H93O10P	[M+Cl]-
967.6158005	967.62	0.0042	4.3405	PG O-48:8;O	C54H93O10P	[M+Cl]-
968.5819431	968.58	0.002	2.0649	Hex2Cer 34:2;O6	C46H85NO17	[M+Formate]-
968.5819431	968.58	0.002	2.0649	Hex2Cer 33:2;O6	C45H83NO17	[M+OAc]-
969.2626885	969.2611	0.0016	1.6507	PIP3 27:5	C36H62O22P4	[M-H]-
969.5353099	969.5346	0.0007	0.7220	PI 40:7;O	C49H81O14P	[M+Formate]-
969.5353099	969.5346	0.0007	0.7220	PI 39:7;O	C48H79O14P	[M+OAc]-
969.547102	969.5475	0.0004	0.4126	PIP 38:2	C47H88O16P2	[M-H]-
970.5947603	970.5946	0.0002	0.2061	PS 46:6;O	C52H90NO11P	[M+Cl]-
970.5947603	970.5956	0.0009	0.9273	Hex2Cer 34:1;O6	C46H87NO17	[M+Formate]-
970.5947603	970.5956	0.0009	0.9273	Hex2Cer 33:1;O6	C45H85NO17	[M+OAc]-
971.5545653	971.5574	0.0029	2.9849	PG 49:12	C55H85O10P	[M+Cl]-
971.5594482	971.5574	0.002	2.0586	PG 49:12	C55H85O10P	[M+Cl]-
971.5994764	971.5997	0.0002	0.2058	PI 40:1;O	C49H93O14P	[M+Cl]-
972.5587868	972.5586	0.0002	0.2056	IPC 41:3;O6	C47H88NO15P	[M+Cl]-
972.5605565	972.5608	0.0002	0.2056	PS 46:10;O	C52H82NO11P	[M+Formate]-
972.5605565	972.5608	0.0002	0.2056	PS 45:10;O	C51H80NO11P	[M+OAc]-

973.5693081	973.5731	0.0038	3.9031	PG 49:11	C55H87O10P	[M+Cl]-
973.5693081	973.5731	0.0038	3.9031	PG O-49:12;O	C55H87O10P	[M+Cl]-
973.5693081	973.5659	0.0034	3.4923	PI 40:5;O	C49H85O14P	[M+Formate]-
973.5693081	973.5659	0.0034	3.4923	PI 39:5;O	C48H83O14P	[M+OAc]-
973.5693775	973.5731	0.0037	3.8004	PG 49:11	C55H87O10P	[M+Cl]-
973.5693775	973.5731	0.0037	3.8004	PG O-49:12;O	C55H87O10P	[M+Cl]-
973.5693775	973.5659	0.0035	3.5950	PI 40:5;O	C49H85O14P	[M+Formate]-
973.5693775	973.5659	0.0035	3.5950	PI 39:5;O	C48H83O14P	[M+OAc]-
974.5724093	974.5742	0.0018	1.8470	IPC 41:2;O6	C47H90NO15P	[M+Cl]-
974.5731103	974.5742	0.0011	1.1287	IPC 41:2;O6	C47H90NO15P	[M+Cl]-
975.5810419	975.5816	0.0005	0.5125	PI 40:4;O	C49H87O14P	[M+Formate]-
975.5810419	975.5816	0.0005	0.5125	PI 39:4;O	C48H85O14P	[M+OAc]-
975.5840511	975.5816	0.0025	2.5626	PI 40:4;O	C49H87O14P	[M+Formate]-
975.5840511	975.5816	0.0025	2.5626	PI 39:4;O	C48H85O14P	[M+OAc]-
976.5885458	976.5899	0.0013	1.3312	IPC 41:1;O6	C47H92NO15P	[M+Cl]-
976.5892564	976.5899	0.0006	0.6144	IPC 41:1;O6	C47H92NO15P	[M+Cl]-
977.5908813	977.5891	0.0017	1.7390	PI 42:4	C51H91O13P	[M+Cl]-
977.5908813	977.5891	0.0017	1.7390	PI O-42:5;O	C51H91O13P	[M+Cl]-
977.5946322	977.5972	0.0026	2.6596	PI 40:3;O	C49H89O14P	[M+Formate]-
977.5946322	977.5972	0.0026	2.6596	PI 39:3;O	C48H87O14P	[M+OAc]-
978.5971662	978.596	0.0012	1.2262	SHexCer 41:0;O6	C47H93NO15S	[M+Cl]-
979.5982092	979.5941	0.0041	4.1854	TG 56:16;O3	C59H82O9	[M+Formate]-
979.6021014	979.6048	0.0027	2.7562	PI 42:3	C51H93O13P	[M+Cl]-
979.6021014	979.6048	0.0027	2.7562	PI O-42:4;O	C51H93O13P	[M+Cl]-
981.5126666	981.5111	0.0016	1.6301	PIP 38:4;O	C47H84O17P2	[M-H]-
981.5722991	981.571	0.0013	1.3244	PI 42:7	C51H85O13P	[M+Formate]-
981.5722991	981.571	0.0013	1.3244	PI O-42:8;O	C51H85O13P	[M+Formate]-
981.5722991	981.571	0.0013	1.3244	PI 41:7	C50H83O13P	[M+OAc]-
981.5722991	981.571	0.0013	1.3244	PI O-41:8;O	C50H83O13P	[M+OAc]-
983.5906068	983.5938	0.0032	3.2534	PA 54:12;O	C57H89O9P	[M+Cl]-
983.5906068	983.5866	0.004	4.0667	PI 42:6	C51H87O13P	[M+Formate]-
983.5906068	983.5866	0.004	4.0667	PI O-42:7;O	C51H87O13P	[M+Formate]-
983.5906068	983.5866	0.004	4.0667	PI 41:6	C50H85O13P	[M+OAc]-
983.5906068	983.5866	0.004	4.0667	PI O-41:7;O	C50H85O13P	[M+OAc]-
984.5715332	984.5666	0.0049	4.9768	MIPC 35:2;O4	C47H88NO18P	[M-H]-
984.5715332	984.5668	0.0047	4.7737	Hex2Cer 36:3;O6	C48H87NO17	[M+Cl]-
984.5715332	984.5666	0.0049	4.9768	MIPC 34:2;O2	C46H86NO16P	[M+Formate]-
984.5715332	984.5666	0.0049	4.9768	MIPC 33:2;O2	C45H84NO16P	[M+OAc]-

984.5715332	984.5666	0.0049	4.9768	MIPC 36:2;O4	C48H90NO18P	[M-CH3]-
985.572104	985.5731	0.001	1.0146	PG 50:12	C56H87O10P	[M+Cl]-
987.5854103	987.5887	0.0033	3.3415	PG 50:11	C56H89O10P	[M+Cl]-
987.5854103	987.5887	0.0033	3.3415	PG O-50:12;O	C56H89O10P	[M+Cl]-
987.5854103	987.5816	0.0039	3.9490	PI 41:5;O	C50H87O14P	[M+Formate]-
987.5854103	987.5816	0.0039	3.9490	PI 40:5;O	C49H85O14P	[M+OAc]-
988.5886262	988.5899	0.0012	1.2139	IPC 42:2;O6	C48H92NO15P	[M+Cl]-
988.5894864	988.5899	0.0004	0.4046	IPC 42:2;O6	C48H92NO15P	[M+Cl]-
988.5894864	988.5921	0.0026	2.6300	PS 47:9;O	C53H86NO11P	[M+Formate]-
988.5894864	988.5921	0.0026	2.6300	PS 46:9;O	C52H84NO11P	[M+OAc]-
989.5647034	989.568	0.0033	3.3348	PG 49:11;O	C55H87O11P	[M+Cl]-
989.6006532	989.6044	0.0037	3.7389	PG 50:10	C56H91O10P	[M+Cl]-
989.6006532	989.6044	0.0037	3.7389	PG O-50:11;O	C56H91O10P	[M+Cl]-
989.6006532	989.5972	0.0034	3.4357	PI 41:4;O	C50H89O14P	[M+Formate]-
989.6006532	989.5972	0.0034	3.4357	PI 40:4;O	C49H87O14P	[M+OAc]-
989.6012798	989.6044	0.0031	3.1326	PG 50:10	C56H91O10P	[M+Cl]-
989.6012798	989.6044	0.0031	3.1326	PG O-50:11;O	C56H91O10P	[M+Cl]-
990.5658206	990.5643	0.0015	1.5143	Hex2Cer 36:5;O6	C48H83NO17	[M+Formate]-
990.5658206	990.5643	0.0015	1.5143	Hex2Cer 35:5;O6	C47H81NO17	[M+OAc]-
990.5707936	990.5691	0.0017	1.7162	MIPC 35:1;O2	C47H90NO16P	[M+Cl]-
990.5982835	990.5996	0.0014	1.4133	PS 49:9	C55H90NO10P	[M+Cl]-
990.5982835	990.5996	0.0014	1.4133	PS O-49:10;O	C55H90NO10P	[M+Cl]-
990.5982835	990.596	0.0023	2.3218	SHexCer 42:1;O6	C48H93NO15S	[M+Cl]-
990.6045576	990.6055	0.001	1.0095	IPC 42:1;O6	C48H94NO15P	[M+Cl]-
991.5797543	991.5837	0.0039	3.9331	PG 49:10;O	C55H89O11P	[M+Cl]-
991.58013	991.5837	0.0035	3.5297	PG 49:10;O	C55H89O11P	[M+Cl]-
993.5426905	993.5475	0.0048	4.8312	PIP 40:4	C49H88O16P2	[M-H]-
994.5835928	994.5815	0.0021	2.1114	PS 49:12	C55H84NO10P	[M+Formate]-
994.5835928	994.5815	0.0021	2.1114	PS 48:12	C54H82NO10P	[M+OAc]-
995.5530108	995.5503	0.0028	2.8125	PI 42:8;O	C51H83O14P	[M+Formate]-
995.5530108	995.5503	0.0028	2.8125	PI 41:8;O	C50H81O14P	[M+OAc]-
997.5694631	997.5659	0.0036	3.6088	PI 42:7;O	C51H85O14P	[M+Formate]-
997.5694631	997.5659	0.0036	3.6088	PI 41:7;O	C50H83O14P	[M+OAc]-
998.5750814	998.5742	0.0009	0.9013	IPC 43:4;O6	C49H90NO15P	[M+Cl]-
998.5750814	998.5764	0.0013	1.3019	PS 48:11;O	C54H84NO11P	[M+Formate]-
998.5750814	998.5764	0.0013	1.3019	PS 47:11;O	C53H82NO11P	[M+OAc]-
998.5791632	998.5823	0.0031	3.1044	MIPC 36:2;O4	C48H90NO18P	[M-H]-
998.5791632	998.5825	0.0033	3.3047	Hex2Cer 37:3;O6	C49H89NO17	[M+Cl]-

998.5791632	998.5764	0.0028	2.8040	PS 48:11;O	C54H84NO11P	[M+Formate]-
998.5791632	998.5823	0.0031	3.1044	MIPC 35:2;O2	C47H88NO16P	[M+Formate]-
998.5791632	998.5764	0.0028	2.8040	PS 47:11;O	C53H82NO11P	[M+OAc]-
998.5791632	998.5823	0.0031	3.1044	MIPC 34:2;O2	C46H86NO16P	[M+OAc]-
998.5791632	998.5823	0.0031	3.1044	MIPC 37:2;O4	C49H92NO18P	[M-CH3]-
999.5852016	999.5887	0.0035	3.5014	PG 51:12	C57H89O10P	[M+Cl]-
999.5852016	999.5816	0.0036	3.6015	PI 42:6;O	C51H87O14P	[M+Formate]-
999.5852016	999.5816	0.0036	3.6015	PI 41:6;O	C50H85O14P	[M+OAc]-
999.5872787	999.5887	0.0015	1.5006	PG 51:12	C57H89O10P	[M+Cl]-

Supplementary Table 7: LipidMatch settings

Settings	Value
Retention time window	±0.15
Window for matching experimental and in-silico fragments	±5 ppm
Mass accuracy window for matching experimental and in-silico precursors for full scan	±0.005 Da
MS/MS isolation Window (for determining MS/MS scans for each feature)	1 Da
Threshold for determining what the minimum signal intensity cut off for a given MS/MS ion should be (used for confirmations)	1000
Minimum number of scans required for the result to be a confirmation (MS/MS)	1

Supplementary Table 8: Annotations for control samples of BUVEC in positive-ion mode

Input Mass	Matched Mass	Delta	ppm	Name	Formula	Adduct
361.2737885	361.2737	0.0001	0.2768	MG(20:4)	C23H37O3	[M+H-H2O]+
363.2889906	363.2894	0.0004	1.1011	MG(20:3)	C23H39O3	[M+H-H2O]+
385.2737679	385.2737	0.0001	0.2596	MG(22:6)	C25H37O3	[M+H-H2O]+
387.2892692	387.2894	0.0001	0.2582	MG(22:5)	C25H39O3	[M+H-H2O]+
566.3198033	566.3217	0.0019	3.3550	LPC(20:4)	C28H50NO7PNa	[M+Na]+
566.3206856	566.3217	0.0010	1.7658	LPC(20:4)	C28H50NO7PNa	[M+Na]+
584.3092298	584.3113	0.0021	3.5940	LPC(20:3)	C28H52NO7PK	[M+K]+
584.3108605	584.3113	0.0004	0.6846	LPC(20:3)	C28H52NO7PK	[M+K]+
611.5388808	611.5398	0.0009	1.4717	DG(O-38:5)	C41H71O3	[M+H-H2O]+
611.5388808	611.5398	0.0009	1.4717	DG(P-38:4)	C41H71O3	[M+H-H2O]+
644.5009491	644.5013	0.0004	0.6206	CerP(d36:2)	C36H71NO6P	[M+H]+
644.5009491	644.5013	0.0004	0.6206	CerP(t36:1)	C36H71NO6P	[M+H-H2O]+
644.5009491	644.5013	0.0004	0.6206	LPC(28:1)	C36H71NO6P	[M+H-H2O]+

644.5009491	644.5013	0.0004	0.6206	PC(P-28:0)	C36H71NO6P	[M+H-H2O]+
644.5009491	644.5013	0.0004	0.6206	PE(O-31:1)	C36H71NO6P	[M+H-H2O]+
644.5009491	644.5013	0.0004	0.6206	PE(P-31:0)	C36H71NO6P	[M+H-H2O]+
678.3393849	678.3379	0.0015	2.2113	PS(24:0(OH))	C30H58NO11PK	[M+K]+
678.3403438	678.3379	0.0024	3.5381	PS(24:0(OH))	C30H58NO11PK	[M+K]+
704.3557465	704.3536	0.0022	3.1234	PI-Cer(d26:2)	C32H60NO11PK	[M+K]+
704.3557465	704.3536	0.0022	3.1234	PS(26:1(OH))	C32H60NO11PK	[M+K]+
706.3714581	706.3692	0.0023	3.2561	PI-Cer(d26:1)	C32H62NO11PK	[M+K]+
706.3714581	706.3692	0.0023	3.2561	PS(26:0(OH))	C32H62NO11PK	[M+K]+
707.374831	707.3742	0.0006	0.8482	PI(23:0)	C32H61O13PNa	[M+Na]+
787.5937226	787.596	0.0023	2.9203	PC(35:3)	C43H84N2O8P	[M+NH4]+
787.5937226	787.596	0.0023	2.9203	PE(38:3)	C43H84N2O8P	[M+NH4]+
787.5937226	787.596	0.0023	2.9203	PE(O-38:4(OH))	C43H84N2O8P	[M+NH4]+
787.5937226	787.596	0.0023	2.9203	PE(P-38:3(OH))	C43H84N2O8P	[M+NH4]+
858.6094119	858.609	0.0004	0.4659	MGDG(41:8)	C50H84NO10	[M+NH4]+
858.6113279	858.6123	0.0010	1.1647	SHexCer(d41:2)	C47H88NO10S	[M+H-H2O]+
859.6141394	859.6171	0.0030	3.4899	PS(40:3)	C46H88N2O10P	[M+NH4]+
859.6141394	859.6171	0.0030	3.4899	PS(O-40:4(OH))	C46H88N2O10P	[M+NH4]+
859.6141394	859.6171	0.0030	3.4899	PS(P-40:3(OH))	C46H88N2O10P	[M+NH4]+
865.6051994	865.6069	0.0017	1.9639	SQDG(37:0)	C46H89O12S	[M+H]+
865.6051994	865.6065	0.0013	1.5018	PC(40:7(OH))	C48H86N2O9P	[M+NH4]+
865.6051994	865.6065	0.0013	1.5018	PE(43:7(OH))	C48H86N2O9P	[M+NH4]+
865.6051994	865.6065	0.0013	1.5018	PS(P-42:6)	C48H86N2O9P	[M+NH4]+
866.6086251	866.6117	0.0031	3.5771	PI-Cer(t39:1)	C45H89NO12P	[M+H]+
866.6086251	866.6058	0.0028	3.2310	PC(44:9)	C52H85NO7P	[M+H-H2O]+
866.6086251	866.6117	0.0031	3.5771	PI(O-36:2)	C45H89NO12P	[M+NH4]+
866.6086251	866.6117	0.0031	3.5771	PI(P-36:1)	C45H89NO12P	[M+NH4]+
902.5885121	902.5882	0.0004	0.4432	PS(43:5)	C49H86NO10PNa	[M+Na]+
902.5885121	902.5883	0.0002	0.2216	PI-Cer(d40:1)	C46H90NO11PK	[M+K]+
902.5885121	902.5883	0.0002	0.2216	PS(40:0(OH))	C46H90NO11PK	[M+K]+
902.5904	902.5906	0.0002	0.2216	PS(45:8)	C51H85NO10P	[M+H]+
902.5904	902.5906	0.0002	0.2216	PS(45:7(OH))	C51H85NO10P	[M+H-H2O]+
903.592499	903.5933	0.0008	0.8854	PI(37:0)	C46H89O13PNa	[M+Na]+
903.592499	903.5933	0.0008	0.8854	PI(O-37:1(OH))	C46H89O13PNa	[M+Na]+
903.592499	903.5933	0.0008	0.8854	PI(P-37:0(OH))	C46H89O13PNa	[M+Na]+
903.592499	903.5917	0.0008	0.8854	MIPC(m31:0)	C43H88N2O15P	[M+NH4]+
907.5337917	907.5331	0.0007	0.7713	PI(40:8)	C49H80O13P	[M+H]+
907.5337917	907.5331	0.0007	0.7713	PI(40:7(OH))	C49H80O13P	[M+H-H2O]+

922.6713775	922.6743	0.0029	3.1430	PI-Cer(t43:1)	C49H97NO12P	[M+H]+
922.6713775	922.6684	0.0030	3.2514	PC(48:9)	C56H93NO7P	[M+H-H2O]+
922.6713775	922.6684	0.0030	3.2514	PE(51:9)	C56H93NO7P	[M+H-H2O]+
922.6713775	922.6743	0.0029	3.1430	PI(O-40:2)	C49H97NO12P	[M+NH4]+
922.6713775	922.6743	0.0029	3.1430	PI(P-40:1)	C49H97NO12P	[M+NH4]+
928.6046923	928.6062	0.0015	1.6153	PS(47:8(OH))	C53H87NO10P	[M+H-H2O]+
928.6046923	928.6038	0.0009	0.9692	PS(45:6)	C51H88NO10PNa	[M+Na]+
928.6046923	928.604	0.0007	0.7538	PI-Cer(d42:2)	C48H92NO11PK	[M+K]+
928.6046923	928.604	0.0007	0.7538	PS(42:1(OH))	C48H92NO11PK	[M+K]+
929.6082157	929.6089	0.0007	0.7530	PI(39:1)	C48H91O13PNa	[M+Na]+
929.6082157	929.6073	0.0009	0.9682	MIPC(m33:1)	C45H90N2O15P	[M+NH4]+
930.6197399	930.6195	0.0003	0.3224	PS(45:5)	C51H90NO10PNa	[M+Na]+
930.6197399	930.6196	0.0001	0.1075	PI-Cer(d42:1)	C48H94NO11PK	[M+K]+
930.6197399	930.6196	0.0001	0.1075	PS(42:0(OH))	C48H94NO11PK	[M+K]+
930.6206399	930.6219	0.0012	1.2895	PS(47:8)	C53H89NO10P	[M+H]+
930.6206399	930.6219	0.0012	1.2895	PS(47:7(OH))	C53H89NO10P	[M+H-H2O]+
930.6206399	930.6195	0.0012	1.2895	PS(45:5)	C51H90NO10PNa	[M+Na]+
930.6206399	930.6196	0.0010	1.0746	PI-Cer(d42:1)	C48H94NO11PK	[M+K]+
930.6206399	930.6196	0.0010	1.0746	PS(42:0(OH))	C48H94NO11PK	[M+K]+
931.6238551	931.6246	0.0007	0.7514	PI(39:0)	C48H93O13PNa	[M+Na]+
931.6238551	931.6246	0.0007	0.7514	PI(P-39:0(OH))	C48H93O13PNa	[M+Na]+
931.6238551	931.623	0.0009	0.9661	MIPC(m33:0)	C45H92N2O15P	[M+NH4]+
931.6880925	931.6903	0.0022	2.3613	SQDG(43:0)	C52H99O11S	[M+H-H2O]+
931.6880925	931.6899	0.0018	1.9320	PC(46:8)	C54H96N2O8P	[M+NH4]+
931.6880925	931.6899	0.0018	1.9320	PE(49:8)	C54H96N2O8P	[M+NH4]+
932.6252655	932.6257	0.0005	0.5361	SHexCer(d42:0)	C48H95NO11SK	[M+K]+
934.6424793	934.6403	0.0022	2.3538	MGDG(47:12)	C56H88NO10	[M+NH4]+
977.6672613	977.6689	0.0016	1.6365	PI(43:2(OH))	C52H98O14P	[M+H]+
1040.621805	1040.6199	0.0019	1.8258	PIP(42:4)	C51H96NO16P2	[M+NH4]+
1040.621805	1040.6199	0.0019	1.8258	PIP(O-42:5(OH))	C51H96NO16P2	[M+NH4]+
1040.621805	1040.6199	0.0019	1.8258	PIP(P-42:4(OH))	C51H96NO16P2	[M+NH4]+
1064.622211	1064.6199	0.0023	2.1604	PIP(44:6)	C53H96NO16P2	[M+NH4]+
1064.622211	1064.6199	0.0023	2.1604	PIP(P-44:6(OH))	C53H96NO16P2	[M+NH4]+
1066.638117	1066.6355	0.0026	2.4376	PIP(44:5)	C53H98NO16P2	[M+NH4]+
1066.638117	1066.6355	0.0026	2.4376	PIP(O-44:6(OH))	C53H98NO16P2	[M+NH4]+
1066.638117	1066.6355	0.0026	2.4376	PIP(P-44:5(OH))	C53H98NO16P2	[M+NH4]+
1068.654043	1068.6512	0.0029	2.7137	PIP(44:4)	C53H100NO16P2	[M+NH4]+
1068.654043	1068.6512	0.0029	2.7137	PIP(O-44:5(OH))	C53H100NO16P2	[M+NH4]+

1068.654043	1068.6512	0.0029	2.7137	PIP(P-44:4(OH))	C53H100NO16P2	[M+NH4]+

Supplementary Table 9: Annotations for control samples of BUVEC in negative-ion mode

Input Mass	Matched Mass	Delta	ppm	Name	Formula	Adduct
342.0787511	342.0784	0.0004	1.1693	NAT 10:2;O2	C12H21NO6S	[M+Cl]-
343.1071639	343.1083	0.0011	3.2060	LPA O-10:2	C13H25O6P	[M+Cl]-
344.094394	344.094	0.0004	1.1625	NAT 10:1;O2	C12H23NO6S	[M+Cl]-
347.1148163	347.1136	0.0012	3.4571	ST 18:5;O7	C18H20O7	[M-H]-
356.0945322	356.094	0.0005	1.4041	NAT 11:2;O2	C13H23NO6S	[M+Cl]-
357.0868134	357.0876	0.0007	1.9603	LPA 10:2	C13H23O7P	[M+Cl]-
368.0945765	368.094	0.0006	1.6300	NAT 12:3;O2	C14H23NO6S	[M+Cl]-
389.1152147	389.1138	0.0014	3.5979	LPA 11:1;O	C14H27O8P	[M+Cl]-
402.0983924	402.0995	0.0011	2.7356	NAT 12:2;O4	C14H25NO8S	[M+Cl]-
403.0987985	403.0988	0	0.0000	ST 18:3;O3;S	C18H24O6S	[M+Cl]-
405.1147775	405.1144	0.0004	0.9874	ST 18:2;O3;S	C18H26O6S	[M+Cl]-
413.0835273	413.0831	0.0004	0.9683	ST 19:5;O3;S	C19H22O6S	[M+Cl]-
417.1143864	417.1144	0	0.0000	ST 19:3;O3;S	C19H26O6S	[M+Cl]-
417.1213344	417.1225	0.0012	2.8769	ST 18:2;O6;S	C18H26O9S	[M-H]-
419.0940071	419.0937	0.0003	0.7158	ST 18:3;O4;S	C18H24O7S	[M+Cl]-
420.1333747	420.1334	0	0.0000	NAT 14:4;O3	C16H25NO7S	[M+Formate]-
429.1146626	429.1144	0.0002	0.4661	ST 20:4;O3;S	C20H26O6S	[M+Cl]-
433.1156861	433.1174	0.0017	3.9250	ST 18:2;O7;S	C18H26O10S	[M-H]-
441.1144929	441.1144	0.0001	0.2267	ST 21:5;O3;S	C21H26O6S	[M+Cl]-
443.1253033	443.1243	0.001	2.2567	LPG 11:3	C17H29O9P	[M+Cl]-
445.1050028	445.1036	0.0014	3.1453	BMP 10:2	C16H27O10P	[M+Cl]-
457.1043667	457.1036	0.0008	1.7502	BMP 11:3	C17H27O10P	[M+Cl]-
461.098438	461.0985	0.0001	0.2169	BMP 10:2;O	C16H27O11P	[M+Cl]-
463.1259018	463.128	0.0021	4.5344	ST 19:2;O8;S	C19H28O11S	[M-H]-
463.1259018	463.128	0.0021	4.5344	ST 18:2;O6;S	C18H26O9S	[M+Formate]-
473.0982597	473.0985	0.0003	0.6341	BMP 11:3;O	C17H27O11P	[M+Cl]-
485.1354237	485.1349	0.0005	1.0306	BMP 13:3	C19H31O10P	[M+Cl]-
493.1371121	493.1385	0.0014	2.8390	ST 19:2;O7;S	C19H28O10S	[M+Formate]-
493.1371121	493.1385	0.0014	2.8390	ST 18:2;O7;S	C18H26O10S	[M+OAc]-
514.1495565	514.1519	0.0024	4.6679	ST 18:1;O8;T	C20H33NO10S	[M+Cl]-
525.1533667	525.1533	0.0001	0.1904	ST 19:5;O4;GlcA	C25H30O10	[M+Cl]-
525.1680191	525.1662	0.0018	3.4275	BMP 16:4	C22H35O10P	[M+Cl]-
527.1317181	527.1326	0.0009	1.7074	ST 18:5;O5;GlcA	C24H28O11	[M+Cl]-

529.1468633	529.1482	0.0014	2.6458	ST 18:4;O5;GlcA	C24H30O11	[M+Cl]-
529.1468633	529.1482	0.0014	2.6458	ST 18:5;O6;Hex	C24H30O11	[M+Cl]-
531.1635507	531.1639	0.0003	0.5648	ST 18:3;O5;GlcA	C24H32O11	[M+Cl]-
531.1635507	531.1639	0.0003	0.5648	ST 18:4;O6;Hex	C24H32O11	[M+Cl]-
537.141238	537.1436	0.0024	4.4681	ST 25:7;O8;S	C25H30O11S	[M-H]-
539.1574317	539.1593	0.0018	3.3385	ST 25:6;O8;S	C25H32O11S	[M-H]-
539.1574317	539.1593	0.0018	3.3385	ST 24:6;O6;S	C24H30O9S	[M+Formate]-
539.1574317	539.1593	0.0018	3.3385	ST 23:6;O6;S	C23H28O9S	[M+OAc]-
540.1529791	540.1545	0.0015	2.7770	ST 21:6;O7;T	C23H29NO9S	[M+Formate]-
541.1476953	541.1482	0.0005	0.9240	ST 19:5;O5;GlcA	C25H30O11	[M+Cl]-
543.127428	543.1275	0.0001	0.1841	ST 18:5;O6;GlcA	C24H28O12	[M+Cl]-
543.1783397	543.1768	0.0016	2.9456	BMP 16:3;O	C22H37O11P	[M+Cl]-
551.157368	551.1593	0.0019	3.4473	ST 26:7;O8;S	C26H32O11S	[M-H]-
551.157368	551.1593	0.0019	3.4473	ST 25:7;O6;S	C25H30O9S	[M+Formate]-
555.1781258	555.1768	0.0014	2.5217	BMP 17:4;O	C23H37O11P	[M+Cl]-
559.1739009	559.1717	0.0022	3.9344	LPI 13:3	C22H37O12P	[M+Cl]-
567.1770476	567.1768	0.0003	0.5289	BMP 18:5;O	C24H37O11P	[M+Cl]-
575.1687765	575.1666	0.0022	3.8250	LPI 13:3;O	C22H37O13P	[M+Cl]-
583.1483173	583.1491	0.0008	1.3719	ST 25:7;O8;S	C25H30O11S	[M+Formate]-
585.1877398	585.1873	0.0004	0.6835	LPI 15:4	C24H39O12P	[M+Cl]-
778.5162159	778.5159	0.0003	0.3853	CerP 41:3;O4	C41H78NO8P	[M+Cl]-
778.5162159	778.5159	0.0003	0.3853	LPC 33:3;O	C41H78NO8P	[M+Cl]-
778.5162159	778.5159	0.0003	0.3853	PC 33:2	C41H78NO8P	[M+Cl]-
778.5162159	778.5159	0.0003	0.3853	PC O-33:3;O	C41H78NO8P	[M+Cl]-
778.5162159	778.5159	0.0003	0.3853	PE 36:2	C41H78NO8P	[M+Cl]-
778.5162159	778.5159	0.0003	0.3853	PE O-36:3;O	C41H78NO8P	[M+Cl]-
835.527128	835.5283	0.0012	1.4362	PA 47:11	C50H77O8P	[M-H]-
835.527128	835.5283	0.0012	1.4362	PA O-47:12;O	C50H77O8P	[M-H]-
835.527128	835.5261	0.001	1.1969	PG 38:3	C44H81O10P	[M+Cl]-
835.527128	835.5261	0.001	1.1969	PG O-38:4;O	C44H81O10P	[M+Cl]-
835.527128	835.5285	0.0014	1.6756	DG 48:12;O2	C51H76O7	[M+Cl]-
835.527128	835.5285	0.0014	1.6756	TG 48:11;O	C51H76O7	[M+Cl]-
835.527128	835.5285	0.0014	1.6756	TG O-48:12;O2	C51H76O7	[M+Cl]-
863.5578143	863.5574	0.0004	0.4632	PG 40:3	C46H85O10P	[M+Cl]-
863.5578143	863.5574	0.0004	0.4632	PG O-40:4;O	C46H85O10P	[M+Cl]-

Supplementary Table 10: Identified markers for infection in negative-ion mode

MALDI				Lipid Maps		MS/MS
Input Mass	Matched Mass	Delta	ppm	Annotation	Adduct	Lipid Match
569.2748070	569.2732	0.0016	2.8106	LPI 16:1	[M-H]-	LPI(16:1) as M-H
571.2901028	571.2889	0.0012	2.1005	LPI 16:0	[M-H]-	LPI(16:0) as M-H
619.4361133	619.4344	0.0017	2.7444	PA 30:0	[M-H]-	PA(14:0_16:0) as M-H
645.4517773	645.4501	0.0017	2.6338	PA 32:1	[M-H]-	PA(14:0_18:1) & PA(16:0_16:1) as M-H
671.4677303	671.4657	0.002	2.9786	PA 34:2	[M-H]-	PA(16:1_18:1) & PA(16:0_18:2) as M-H
690.5091403	690.5079	0.0012	1.7379	PE 32:0	[M-H]-	PE(16:0_16:0) as M-H
725.5146533	725.5127	0.002	2.7567	PA 38:3	[M-H]-	PA(18:1_20:2) & PA(18:0_20:3) & PA(18:2_20:1) as M-H
750.5316162	750.5291	0.0025	3.331	PC 30:0	[M+Formate]-	PC(14:0_16:0)+HCO2
775.5513417	775.5495	0.0019	2.4499	PG 36:1	[M-H]-	PG(18:0_18:1) & PG(16:1_20:0) & PG(16:0_20:1) as M-H
781.4886410	781.4873	0.0014	1.7915	PI 30:0	[M-H]-	PI(14:0_16:0) as M-H
807.5057632	807.5029	0.0029	3.5913	PI 32:1	[M-H]-	PI(16:0_16:1) & PI(14:0_18:1) & PI(14:1_18:0) as M-H
808.5112476	808.5134	0.0022	2.721	PS 38:5	[M-H]-	PS(18:0_20:5) & PS(18:1_20:4) & PS(16:0_22:5) as M-H
831.5025059	831.5029	0.0004	0.4811	PI 34:3	[M-H]-	PI(16:0_18:3) & PI(16:1_18:2) & PI(14:0_20:3) as M-H
833.5202337	833.5186	0.0017	2.0395	PI 34:2	[M-H]-	PI(16:1_18:1) & PI(16:0_18:2) & PI(14:0_20:2) as M-H
834.5253770	834.5291	0.0037	4.4336	PS 40:6	[M-H]-	PS(18:0_22:6) & PS(18:1_22:5) as M-H
835.5370483	835.5342	0.0028	3.3511	PI 34:1	[M-H]-	PI(16:0_18:1) & PI(16:1_18:0) as M-H
836.5412607	836.5447	0.0035	4.1839	PS 40:5	[M-H]-	PC(17:1_20:5) as M+HCO2 & PS(18:0_22:5) as M-H
836.5412607	836.5447	0.0035	4.1839	PC 37:6	[M+Formate]-	PC(17:1_20:5) as M+HCO2 & PS(18:0_22:5) as M-H
846.5856323	846.5866	0.0009	1.0631	PC 36:2;O	[M+Formate]-	OxPC(18:1_18:1(OH)) & OxPC(18:0_18:2(OH)) & OxPC(18:0_18:1(Ke)) & OxPC(18:1_18:1(1O)) & OxPC(18:1_18:0(1O(1Cyc))) & OxPC(18:0_18:1(1O(1Cyc))) & OxPC(18:0_18:2(1O)) as M+HCO2
847.5367752	847.5342	0.0026	3.0677	PI 35:2	[M-H]-	PI(15:0_20:2) & PI(15:1_20:1) & PI(17:1_18:1) & PI(17:0_18:2) & PI(17:2_18:0) as M-H

849.5499878	849.5499	0.0001	0.1177	PI 35:1	[M-H]-	PI(15:0_20:1) & PI(17:0_18:1) & PI(17:1_18:0) & PI(16:1_19:0) as M-H
850.5582004	850.5604	0.0022	2.5865	PC 38:6	[M+Formate]-	PC(18:1_20:5) & PC(18:2_20:4) & PC(16:1_22:5) & PC(16:0_22:6) as M+HCO2
851.5655126	851.5655	0	0	PI 35:0	[M-H]-	PI(17:0_18:0) & PI(16:0_19:0) as M-H
857.5208326	857.5186	0.0023	2.6822	PI 36:4	[M-H]-	PI(16:0_20:4) & PI(16:1_20:3) & PI(18:0_18:4) & PI(18:1_18:3) & PI(18:2_18:2) as M-H
859.5365875	859.5342	0.0024	2.7922	PI 36:3	[M-H]-	PI(16:0_20:3) & PI(18:1_18:2) & PI(18:0_18:3) & PI(16:1_20:2) as M-H
861.5518176	861.5499	0.002	2.3214	PI 36:2	[M-H]-	PI(18:0_18:2) & PI(18:1_18:1) & PI(16:0_20:2) & PI(16:1_20:1) as M-H
863.5670722	863.5655	0.0016	1.8528	PI 36:1	[M-H]-	PI(18:0_18:1) & PI(16:1_20:0) & PI(16:0_20:1) as M-H
889.5830982	889.5812	0.0019	2.1358	PI 38:2	[M-H]-	PI(18:0_20:2) & PI(18:1_20:1) & PI(16:0_22:2) & PI(16:1_22:1) & PI(18:2_20:0) as M-H
890.5877605	890.5917	0.0039	4.3791	PS 44:6	[M-H]-	PS(22:0_22:6) as M-H

Supplementary Table 11: Identified markers for infection in positive-ion mode

MALDI				Lipid Maps		MS/MS	
Input Mass	Matched Mass	Delta	ppm	Annotation	Formula	Adduct	Lipid Match
468.3086543	468.3085	0.0002	0.4271	LPC(14:0)	C22H46NO7P	[M+H]+	LPC(14:0) as M+H
612.5554329	612.5561	0.0007	1.1428	DG(34:1)	C37H70O5	[M+NH4]+	DG(16:0_18:1) & DG(16:1_18:0) & DG(14:0_20:1) as M+NH4
664.4911038	664.4912	0.0001	0.1505	PE(30:0)	C35H70NO8P	[M+H]+	PE(14:0_16:0) & 3_PE(30:0) as M+H*
678.5067599	678.5068	0.0001	0.1474	PC(28:0)	C36H72NO8P	[M+H]+	PC(14:0_14:0) & PC(12:0_16:0) &

							PC(13:0_15:0) as M+H
692.5224818	692.5225	0	0	PC(29:0)	C37H74NO8P	[M+H]+	PC(14:0_15:0) as M+H
704.5224864	704.5225	0	0	PC(30:1)	C38H74NO8P	[M+H]+	PC(14:0_16:1) & PC(12:0_18:1) as M+H
706.5380984	706.5381	0	0	PC(30:0)	C38H76NO8P	[M+H]+	PC(14:0_16:0) as M+H
728.5199054	728.5201	0.0002	0.2745	PC(30:0)	C38H76NO8PNa	[M+Na]+	PC(14:0_16:0) as M+Na
754.5358589	754.5357	0.0001	0.1325	PC(32:1)	C40H78NO8PNa	[M+Na]+	PC(14:0_18:1) & PC(16:0_16:1) as M+Na
786.6008673	786.6007	0.0001	0.1271	PC(36:2)	C44H84NO8P	[M+H]+	PC(18:1_18:1) as M+H
836.5410680	836.5436	0.0025	2.9885	PS(40:6)	C46H78NO10P	[M+H]+	PS(18:0_22:6) as M+H
846.5239137	846.5256	0.0016	1.8901	PS(39:5)	C45H78NO10PNa	[M+Na]+	PS(39:5) as M+Na*

Supplementary Table 12: Identified markers for control cells in positive-ion mode

MALDI				Lipid Maps			MS/MS
Input Mass	Matched Mass	Delta	ppm	Annotation	Formula	Adduct	Lipid Match
566.3206856	566.3217	0.001	1.7658	LPC(20:4)	C28H50NO7PNa	[M+Na]+	LPC(20:4) as M+Na*
932.6252655	932.6223	0.003	3.2167	PI(40:4)	C49H91NO13P	[M+NH4]+	PI(40:4) as M+NH4*
934.6424793	934.6379	0.0046	4.9217	PI(40:3)	C49H93NO13P	[M+NH4]+	PI(40:3) as M+NH4

Supplementary Table 13: Comparison of identified infection markers with literature[30]

MALDI	Lipid Maps			MS/MS	Literature	
Input Mass	Matched Mass	ppm	Annotation	Lipid Match	Species	MS/MS Lipid Match
754.5358589	754.5357	0.1325	PC(32:1)	PC(14:0_18:1) & PC(16:0_16:1) as M+Na	T. gondii	PC(14:0_18:1) as M+Na
775.5513417	775.5495	2.4499	PG 36:1	PG(18:0_18:1) & PG(16:1_20:0) & PG(16:0_20:1) as M-H	T. gondii	PG(18:0_18:1) as M-H
781.4886410	781.4873	1.7915	PI 30:0	PI(14:0_16:0) as M-H	B. besnoiti	PI(14:0_16:0) as M-H
807.5057632	807.5029	3.5913	PI 32:1	PI(16:0_16:1) & PI(14:0_18:1) & PI(14:1_18:0) as M-H	B. besnoiti	PI(14:0_18:1) & PI(16:0_16:1) as M-H
833.5202337	833.5186	2.0395	PI 34:2	PI(16:1_18:1) & PI(16:0_18:2) & PI(14:0_20:2) as M-H	T. gondii, B. besnoiti	PI(16:0_18:2) & PI(16:1_18:1) as M-H

* Only identified in a single measurement

**835.5370483**	**835.5342**	**3.3511**	**PI 34:1**	**PI(16:0_18:1) & PI(16:1_18:0) as M-H**	**B. besnoiti**	**PI(16:1_18:0) & PI(16:0_18:1) as M-H**
**857.5208326**	**857.5186**	**2.6822**	**PI 36:4**	**PI(16:0_20:4) & PI(16:1_20:3) & PI(18:0_18:4) & PI(18:1_18:3) & PI(18:2_18:2) as M-H**	**B. besnoiti**	**PI(16:0_20:4) as M-H**
**859.5365875**	**859.5342**	**2.7922**	**PI 36:3**	**PI(16:0_20:3) & PI(18:1_18:2) & PI(18:0_18:3) & PI(16:1_20:2) as M-H**	**B. besnoiti**	**PI(16:1_20:2) & PI(18:0_18:3) & PI(18:1_18:2) as M-H**
**861.5518176**	**861.5499**	**2.3214**	**PI 36:2**	**PI(18:0_18:2) & PI(18:1_18:1) & PI(16:0_20:2) & PI(16:1_20:1) as M-H**	**T. gondii**	**PI(16:0_20:2) & PI(16:1_20:1) & PI(18:1_18:1) & PI(18:0_18:2) & PI(18:1_18:1) as M-H**
**863.5670722**	**863.5655**	**1.8528**	**PI 36:1**	**PI(18:0_18:1) & PI(16:1_20:0) & PI(16:0_20:1) as M-H**	**T. gondii, B. besnoiti**	**PI(16:0_20:1) & PI(18:0_18:1) as M-H**
